# Torsional and Electronic Factors Control the C−H⋅⋅⋅O Interaction

**DOI:** 10.1002/chem.201602905

**Published:** 2016-10-06

**Authors:** Russell W. Driver, Timothy D. W. Claridge, Steve Scheiner, Martin D. Smith

**Affiliations:** ^1^ Chemistry Research LaboratoryUniversity of Oxford12 Mansfield RoadOxfordOX1 3TAUK; ^2^ Department of Chemistry and BiochemistryUtah State UniversityLoganUtah84322-0300USA

**Keywords:** crystallography, density functional calculations, foldamers, hydrogen bonds, NMR spectroscopy

## Abstract

The precise role of non‐conventional hydrogen bonds such as the C−H⋅⋅⋅O interaction in influencing the conformation of small molecules remains unresolved. Here we survey a series of β‐turn mimetics using X‐ray crystallography and NMR spectroscopy in conjunction with quantum calculation, and conclude that favourable torsional and electronic effects are important for the population of states with conformationally influential C−H⋅⋅⋅O interactions. Our results also highlight the challenge in attempting to deconvolute a myriad of interdependent noncovalent interactions in order to focus on the contribution of a single one. Within a small molecule that is designed to resemble the complexity of the environment within peptides and proteins, the interplay of different steric burdens, hydrogen‐acceptor/‐donor properties and rotational profiles illustrate why unambiguous conclusions based solely on NMR chemical shift data are extremely challenging to rationalize.

## Introduction

**Figure 1:**
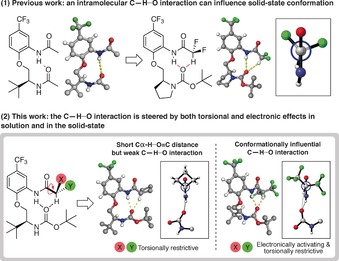
Strategy to probe torsional and electronic control of the C−H⋅⋅⋅O interaction.

A complex ensemble of noncovalent interactions, including hydrogen bonds, drives the folding of biopolymers into functional conformations. Relatively weak interactions such as the C−H⋅⋅⋅O hydrogen bond^1^ make important contributions to the folding process but their potential for directly influencing conformation is unclear.^2^ For instance, the C−H⋅⋅⋅O interaction likely contributes to the stability of the A⋅T base pair in the Hoogsteen geometry,^3^, ^4^ and is also a key component governing certain catalytic enantioselective processes.^5^ Increasingly, these nonconventional hydrogen bonds are considered to play a significant role in molecular recognition involved in the binding of small molecules to large ones.^6^ We have previously investigated whether an intramolecular C−H⋅⋅⋅O interaction can influence the conformation of small molecules in the solid‐state through a combined crystallographic and computational study.^7^ This approach demonstrated that for certain α,α‐difluoroamides the C−H⋅⋅⋅O interaction could be a determinant of conformation, with bond enthalpies of up to 3.5 kcal mol^−1^ through increased polarization of the Cα−H bond. However, distinguishing between an attractive C−H⋅⋅⋅O hydrogen bond and a coincidental close contact proved challenging and we subsequently began to investigate the factors that determine whether a weak interaction is conformationally influential.^8^ The study of noncovalent interactions is most relevant when examined in a dynamic environment and hence we decided to extend our work to encompass the solution‐state. We recognized that a thorough study would require: 1) a conformationally well‐defined platform that would allow us to study the C−H⋅⋅⋅O interaction; 2) the ability to vary both acceptor and donor groups around this scaffold; 3) suitable methods to gauge the influence of the C−H⋅⋅⋅O interaction on global conformation. Consequently, we decided to utilize an established parallel β‐turn scaffold which would allow us to systematically probe the influence that of a C−H⋅⋅⋅O donor on the rotation of a single bond within the confines of a conformationally well‐defined system (Figure 1). We and others have previously extensively investigated the conformational properties of this peptidomimetic in the solid and solution state and have shown that β‐turn like conformations stabilized by intramolecular hydrogen bonding are well‐populated for most derivatives.^9^ Our motivation in choosing a small and dynamic β‐turn mimetic was to perform our investigation in a construct resembling a natural protein and peptide environment rather than a simple chemical model. Our system has a multitude of rotatable bonds and many interdependent noncovalent interactions, which significantly complicate analysis but which are necessary to probe the factors that make the C−H⋅⋅⋅O interaction influential within small molecule models, synthetic foldamers and ultimately biomacromolecules.

## Results and Discussion

### Solid state studies

**Figure 2:**
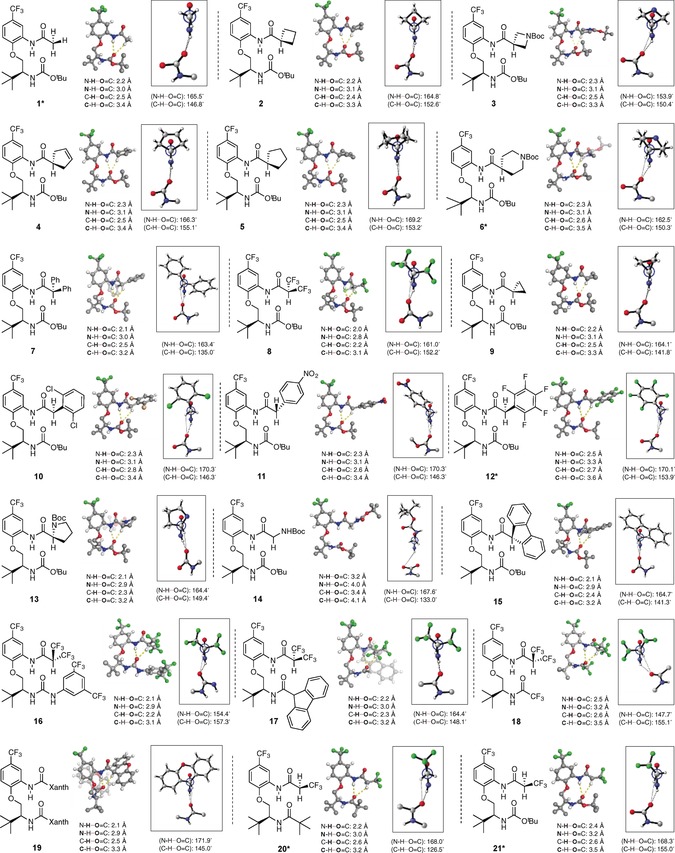
Solid‐state conformations of β‐turn mimic bearing different C−H⋅⋅⋅O donors with relevant intramolecular distances and angles (some atoms omitted for clarity; distances [Å] are indicated between atoms in bold).^10^ Positions of hydrogen atoms are calculated. *: Asymmetric unit contains two conformationally similar but crystallographically unique molecules; distances and hydrogen‐bond angles are given for only one molecule. Xanth=9‐xanthene.

We rationalized that to probe the influence of the C−H⋅⋅⋅O interaction would require the synthesis of a series of α,α‐disubstituted derivatives possessing donor and acceptor moieties of different sizes (and hence different torsional profiles) and varying electronic properties. The conformation of these materials was initially examined in the solid‐state using single crystal X‐ray crystallography (Figure 2).^10^ The substrates we examined are sterically and electronically diverse, but the vast majority were found to populate β‐turn like structures with intranuclear distances consistent with a N−**H**⋅⋅⋅**O**=C hydrogen bond between the aromatic amide proton donor and the carbonyl oxygen acceptor, as expected. These distances varied significantly between 2.0 Å for powerful α‐electron withdrawing groups such as the bistrifluoromethyl substrate **8**, and 2.5 Å for other groups such as pentafluorophenylbenzyl derivative **12**. Less polarized substituents such as alkyl groups (typified by **2**–**6**) generally possess longer N−**H**⋅⋅⋅**O**=C bond lengths.^11^ We recognized that many substrates contain aromatic rings and were therefore capable of engaging in additional inter‐ and intramolecular interactions that could potentially affect the global conformation. In our search for these additional interactions (particularly C−H⋅⋅⋅π contacts) we were guided by distance criteria established during surveys of protein X‐ray structures and theoretical studies.^12^

We were initially interested in whether changing the size of α‐carbonyl substituents through disubstitution and cyclization without explicitly increasing Cα−H acidity, would affect rotation around the HCCO torsion. Compounds **1**–**6** explore this question through a series of simple alkyl derivatives. Although there are no obvious additional interactions in the crystals of these materials (**1**–**6**) that appear to be responsible for influencing the rotational states of the α,α‐disubstituted amide moiety, incidental intermolecular hydrogen bonds between adjacent molecules can be observed within the crystal lattice of these and all other compounds.^10^ Close examination of the X‐ray crystal structure of acyl derivative **1** reveals a β‐turn‐like motif featuring an N−**H**⋅⋅⋅**O**=C distance of 2.2 Å, consistent with this hydrogen bond being a stabilizing element. The C−**H**⋅⋅⋅**O**=C distance (2.5 Å) and angle *θ* (147°, C−H⋅⋅⋅O=C) in **1** is also consistent with the presence of a C−H⋅⋅⋅O interaction, but we have previously commented that the shallow rotational energy profile for related materials bearing simple acyl groups is inconsequential to the observed conformation.^7^ Therefore we also compared the C−**H**⋅⋅⋅**O**=C distances in **2**–**6** to determine whether disubstitution and cyclization could be influencing the putative C−H⋅⋅⋅O interaction. These internuclear distances are between 2.4 and 2.6 Å and all are consistent with the presence of a C−H⋅⋅⋅O hydrogen bond. Similarly, examination of the angle *θ*(C−H⋅⋅⋅O=C) across structures **1**–**6** reveals a relatively narrow range of values between 147° and 155°, all of which lie within the conventionally accepted angle ranges for identification of a C−H⋅⋅⋅O interaction.^13^ For compounds **2**–**6** the measured angles and distances are largely similar to those observed for **1**.

We next decided to examine compounds **7**–**14**, which bear substituents designed to probe the effect of changing the Cα−H acidity relative to acyl control compound **1**. All appear to populate turn‐like conformations in the solid‐state, but a closer examination of BocGly derivative **14** shows an intramolecular distance (3.2 Å) much larger than normal; this is consistent with the global conformation in this case being dominated by intermolecular hydrogen bonding interactions in the crystal lattice.^14^ In contrast, compounds **7**–**13** show no additional important intermolecular interactions and have relatively short N−**H**⋅⋅⋅**O**=C distances (between 2.0 and 2.5 Å) consistent with hydrogen bonding. C−**H**⋅⋅⋅**O**=C distances vary between 2.2 Å (for bistrifluoromethylamide derivative **8**) and 2.7 Å (for pentafluorophenylbenzyl derivative **12**). Bistrifluoroamide **8** has a significantly shorter C−**H**⋅⋅⋅**O**=C distance than any other member in this series, which appears to be consistent with its powerful electron withdrawing ability. Compound **7**, which has a benzylic C−H proton, and would consequently be expected to be more acidic, does not have a significantly shorter C−**H**⋅⋅⋅**O**=C distance than **1**. A similar observation can be made for cyclopropane **9**, which is somewhat more acidic than an acyclic alkane by virtue of its greater s‐orbital character.^15^ Substituted benzyl derivatives **10**, **11** and **12** have relatively long C−**H**⋅⋅⋅**O**=C distances (2.8, 2.6 and 2.7 Å, respectively) when compared to **1**, which appears to result from a balance between the proximity of the sterically demanding arene and Boc groups and stabilization by the C−H⋅⋅⋅O interaction. There is no indication that pentafluorophenyl derivative **12** participates in significant C−H⋅⋅⋅π interactions. BocPro derivative **13** possesses a relatively short C−**H**⋅⋅⋅**O**=C distance of 2.3 Å; this is shorter than geometrically similar cyclopentane **5** and electronically similar BocGly derivative **14** and likely results from unique torsional preferences within the turn scaffold.

Bistrifluoromethyl derivative **8** has the shortest C−**H**⋅⋅⋅**O**=C distance and was therefore used as a baseline to evaluate other compounds with various hydrogen‐bond acceptor groups (**16**–**18**). As ureas are somewhat better hydrogen‐bond acceptors than carbamates,^16^ we first synthesized urea derivative **16**. This compound has a short (2.2 Å) C−**H**⋅⋅⋅**O**=C distance, which again (see **10**–**12** above) likely results from a balance between the steric demand of the bistrifluoromethylaryl group and the increased hydrogen‐bond acceptor ability of the urea. We also synthesized amides **17** and **18** with the expectation that the trifluoromethyl amide **18** would be a poorer hydrogen‐bond acceptor than **17**. This is borne out by the observation that the **H**⋅⋅⋅**O**=C distances are 2.3 and 2.6 Å (for **17** and **18**, respectively), although the steric demands of these two amides are obviously different. We also examined the X‐ray structures of **15**, **16** and **17** for evidence of interactions involving the aromatic group (particularly H⋅⋅⋅π contacts) but as in **10**–**12** no interactions relevant to the global conformation were found.

**Figure 3:**
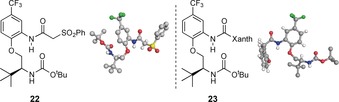
Solid‐state conformations of constructs that do not populate β‐turn like conformations. Positions of hydrogen atoms are calculated.

These bistrifluoromethylamides can also be compared with monotrifluoromethylamides **20** and **21**, which possess different hydrogen‐bond acceptor groups. It was expected that a monotrifluoromethylamide group would be a poorer H⋅⋅⋅O donor than a bistrifluoromethylamide group, and this is in fact reflected in the longer C−**H**⋅⋅⋅**O**=C distance in **21** (2.6 Å) versus **8** (2.2 Å). It is relevant to note that several of the compounds we examined (specifically **22** and **23**) did not populate turn‐like conformations in the solid‐state (Figure 3).The global conformation of these materials is dominated by intermolecular hydrogen bonds and crystal packing forces that preclude observation of the intramolecular interactions of interest; these compounds are thus included here for completeness but their solid‐state structures are a departure from our usual observations.

### Solution state study: hydrogen‐bond donors

**Figure 4:**
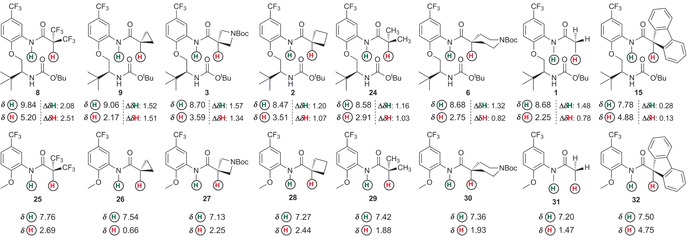
^1^H NMR chemical shift data for selected compounds bearing different C−H⋅⋅⋅O bond donors versus controls (C_6_D_6_, 298 K, 50 μm). Compounds are in decreasing Cα−H Δ*δ* **H**.

In general, our solid‐state survey of C−H⋅⋅⋅O donors and acceptors shows that many compounds possess internuclear distances and angles consistent with the presence of multiple noncovalent interactions. However, in isolation these observations do not constitute a demonstration that a specific potential interaction is necessarily important or influential in the overall folding process, as solid‐state studies of crystals provide a wealth of information about small‐molecule geometry but betray little about the energetic or dynamic aspects. Consequently we decided to expand our study and examine the ^1^H NMR spectra of a cross‐section of these compounds and relate observable parameters such as ^1^H chemical shift to the potential strength of these interactions through quantum calculation (Figure 4).^17^ We chose a structurally diverse series of derivatives to investigate the effects of both torsional and electronic factors on the C−H⋅⋅⋅O interaction, and also prepared a corresponding series of control compounds that do not possess the carbamate intramolecular hydrogen‐bond acceptor. These are imperfect control compounds as their conformational preferences are necessarily different from compounds bearing an intramolecular hydrogen‐bond acceptor group, but they nonetheless provide a valuable benchmark for comparison. Dilution experiments ruled out intermolecular aggregation at concentrations below 50 mm, and a suite of 2D experiments permitted assignment of all spin systems and confirmed that all compounds examined populate a turn‐like conformation in solution, similar to that observed in the solid‐state (Figure 4). Hydrogen bonding is manifested in ^1^H NMR spectroscopy through a reduction in diamagnetic shielding and hence we examined the chemical shifts of amide N−**H** (shown in green in all Figures) and Cα−**H** (shown in red in all Figures) protons.^18^ ^1^H NMR chemical shift data appear consistent with the involvement of amide N−**H** and Cα−**H** protons in hydrogen bonds for all compounds (with the possible exception of **15**), as both are deshielded relative to their controls. Fluorenyl derivative **15** has only small chemical shift differences relative to its control **32**, potentially consistent with relatively weak and conformationally inconsequential solution‐state interactions. This is a significant departure from the solid‐state structure of **15**, in which close C−H⋅⋅⋅O and N−H⋅⋅⋅O contacts were observed. This discrepancy may derive from differences in the solution and solid‐state conformations of **15**, but is more likely a demonstration of the limitations intrinsic to our control compounds. In general the amide N−**H** and Cα−**H** proton chemical shift differences vary significantly within the series, particularly for the Cα−**H** protons.^19^ For our nominal control compound **1**, the change in chemical shift (Δ*δ*C**H**) versus control compound **31** is 0.78 ppm.^20^ The Cα−**H** donor in **1** is only very moderately polarized by the adjacent carbonyl; we have previously demonstrated that similar compounds have an almost flat rotational profile, consistent with a very weak C−H⋅⋅⋅O interaction.^7^ This suggests that a significant contributor to the deshielding observed in **1** is an effect other than hydrogen bonding. It is known that proton chemical shifts are sensitive to magnetic anisotropic effects from carbonyl groups proximal to the Cα−**H**, and also to steric and electric field effects; these are likely responsible for the observed Δ*δ*C**H** in **1** versus **31**.^21^ A related study estimated that the deshielding of a proton involved in C−H⋅⋅⋅O hydrogen bonding in bindone analogues was mostly due to these other effects, with only 0.6 ppm (of a 1.8 ppm shift) ascribed to the influence of hydrogen bonding.^22^ Examination of **24** 23 versus **29**, and **2** versus **28** demonstrated that the chemical shift differences are significantly higher (Δ*δ*C**H**=1.03 and 1.07 ppm, respectively) than the difference between **1** and **31**; this is counterintuitive as compounds bearing α,α‐dialkyl groups such as **24** or **2** are not significantly better hydrogen‐bond donors than **1**.^24^ This shift difference is instead consistent with a larger population of conformers that place the Cα−**H** in proximity to the carbonyl group, possibly because non‐hydrogen bonded conformers are disfavoured through steric or torsional effects, thereby increasing the significance of the C−H⋅⋅⋅O interaction across the time‐averaged ensemble. Although this mechanism is well known in peptides containing α,α‐dialkyl amino acids,^25^ it is difficult to prove here conclusively. Azetidine 3‐carboxylic acid derivative **3** and its control **27** demonstrate a larger shift difference than cyclobutanes **2** and **28** (Δ*δ*C**H**=1.34 ppm vs. Δ*δ*C**H**=1.07 ppm, respectively); this appears to be at odds with solid‐state data, where C−**H**⋅⋅⋅**O**=C distances for **2** and **3** are 2.4 and 2.5 Å, respectively. This is likely a consequence of the azetidine Boc group functioning as a hydrogen‐bond donor in the solid‐state, which obscures the increased hydrogen bond acidity of the azetidine **3** Cα−**H** versus cyclobutane **2**. An even larger chemical shift difference is apparent in the cyclopropanes **9** and **26** (Δ*δ*C**H**=1.51 ppm),^26^ which is consistent with favourable electronic and torsional effects working in concert to enhance cyclopropane Cα−**H** hydrogen‐bond donor ability through increased s‐orbital character and restriction around the *ϕ*(OCCH) torsion by virtue of α,α‐disubstitution. Bistrifluoromethyl substituted example **8** exhibits the largest chemical shift difference versus **25** (Δ*δ*C**H**=2.51 ppm).^27^ We attribute this predominantly to the electron‐withdrawing effect of the trifluoromethyl groups, enhancing the hydrogen‐bond donor ability of the Cα−**H**, but also to their increased size versus the alkyl groups (compounds **2**–**6**) described earlier.^28^, ^29^ Thus, if **8** is compared to **24**, there is a clear effect due to the increased size of CF_3_ relative to CH_3_, which leads to torsional restriction about the OCCH bond and favours conformations that place Cα−**H** in close proximity to the carbonyl oxygen (as in **9**). However, the largest contributor to chemical shift is likely the electron‐withdrawing effect of substitution with electronegative fluorine atoms, which polarize the Cα−**H** bond and result in a more significant C−H⋅⋅⋅O interaction. The combination of these favourable torsional and electronic factors leads to the large Δ*δ*C**H** observed for 4.

In order to probe further whether these chemical‐shift changes are consistent with the presence of hydrogen bonds, we examined the temperature dependence of four key compounds (Table 1).^30^ The amide temperature coefficients are almost identical across this series, consistent with the thermally mediated change in environment being similar. We interpret this as being a reflection of conserved N−H⋅⋅⋅O hydrogen bond strength, as the length (2.0–2.2 Å) is almost invariant across **1**, **2**, **8** and **24**. In contrast, examination of the temperature coefficients for the Cα−**H** protons shows that: 1) **8** has the largest negative coefficient; 2) **2** and **24** have very similar and relatively large negative coefficients; and 3) the temperature coefficient for **1** has a significantly smaller value than **2**, **8**, or **24** (Table 1). We interpret these larger Cα−**H** temperature coefficients as reflecting more drastic environmental changes upon thermal perturbation that ultimately lead to an increase in the average internuclear Cα−**H** to **O**=C distance. With increasing temperature in an aprotic solvent, the population‐weighted average will reflect: 1) a greater contribution from non‐hydrogen‐bonded conformations leading to reduced deshielding of protons involved in hydrogen bonds, and hence larger negative temperature coefficients, 2) a smaller contribution of the magnetic anisotropic effect as proximity to the carbonyl group is reduced. The larger temperature coefficient of Cα−**H** in **24** and **2** versus **1** could thus be a consequence of a “stronger” C−H⋅⋅⋅O interaction. However, although torsional restriction could certainly potentiate the C−H⋅⋅⋅O interaction in **24** by disfavouring conformations with *ϕ*(OCCH) significantly different from 180°, we feel that the contribution of this effect to the temperature coefficient is likely small compared to that of magnetic anisotropy. Compound **8** has a significantly larger Cα−**H** temperature coefficient than **2**, **24** or **1** and we attribute this to a combination of significant torsional restriction (placing the C−H group closer to the carbonyl oxygen) and considerable Cα−**H** polarization (increasing the hydrogen‐bond donor ability). In the case of **8** we are confident that the large positive chemical ^1^H shift difference versus control (2.51 ppm) and large negative temperature coefficient (4.1 ppb K^−1^) indicate the presence of a C−H⋅⋅⋅O interaction that influences global conformation in the solution‐state.

**Table 1:** Temperature coefficients for protons potentially involved in H‐bonding interactions.^[a]^

	Cmpd.	R^1^/R^2^	Cα−**H** (red)	N−**H** (green)
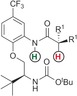	**8**	CF_3_/CF_3_	−4.1	−5.1
	**2**	cyclobutyl	−3.0	−5.5
	**24**	Me/Me	−3.0	−5.2
	**1**	H/H	−1.7	−5.2

[a] Spectra recorded at 500 MHz, [D_8_]toluene, 50.0 μm, Δ*δ* in ppb K^−1^.

### Solution state study: hydrogen‐bond acceptors

**Figure 5:**
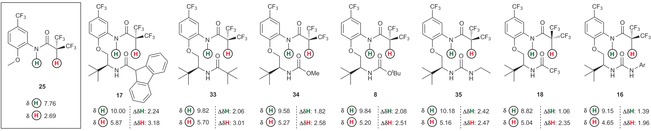
^1^H NMR chemical shift data for selected compounds bearing different hydrogen‐bond acceptors versus control **25** (C_6_D_6_, 298 K, 50 μm). Compounds are ordered in decreasing Cα−H Δ*δ* **H**. Ar=3,5‐(CF_3_)_2_C_6_H_3_.

We next surveyed the effects of changing the hydrogen‐bond acceptor group in **8** by comparing the ^1^H NMR chemical shifts of amide N−**H** and Cα−**H** protons to those of control molecule **25** (Figure 5). We chose to examine compounds possessing a bistrifluoromethyl group as the putative C−H⋅⋅⋅O donor because both solid‐state and solution‐state data for **8** were consistent with the presence of a conformationally influential C−H⋅⋅⋅O interaction in this molecule. As discussed earlier, it is challenging to deconvolute the factors contributing to a chemical shift and observe directly the effect of hydrogen bonding within an individual compound, but using ^1^H NMR spectroscopy data from a short series of amides, carbamates and ureas, all of which are proficient hydrogen‐bond acceptors, we sought to delineate general trends. When analysing compounds such as carbamate **8**, where the hydrogen‐bond acceptor oxygen has two lone pairs, we must also consider how one hydrogen bond could affect the strength of the other, as it has been shown that participation in an intramolecular hydrogen bond reduces the propensity of an atom to accept additional intermolecular hydrogen bonds.^31^ The largest Δ*δ*C**H** shifts are observed for amides **17** and **33** (Δ*δ*C**H** 3.18 and 3.01 ppm, respectively), followed by carbamates 34 and **8**, whilst ureas **35** and **16** demonstrate smaller shifts. By some measures, ureas may be considered to be more powerful hydrogen‐bond acceptors than amides which makes this observation difficult to directly explain.^16^ While ethyl urea **35** has a Δ*δ*C**H** value similar to that observed in **34** and **8** it does have a significantly higher Δ*δ*N**H** value and therefore potentially a stronger N−H⋅⋅⋅O bond than the other compounds in this series; the Δ*δ*C**H** value may reflect only electronic factors, as the steric profile of the ethyl substituent is small. Although it is possible that the unexpectedly low Δ*δ*C**H** value for **16** results from shielding of the Cα−**H** by the bistrifluoromethylphenyl substituent, there is no indication of this in the X‐ray structure, and it may that the solution and solid‐state conformational preferences differ substantially (as in fluorenyl derivative **15**). In contrast, amides **17** and **33** show relatively large Δ*δ*C**H** values, which is consistent with their steric relatively bulk, favouring hydrogen‐bonded conformations. Trifluoromethyl substituted amide **18**, which would be expected to be a poorer hydrogen‐bond acceptor, does present a smaller Δ*δ*C**H** than the other amides. There are only relatively small differences between carbamates **34** and **8**, consistent with an increase in bulk (for **8**) having relatively little effect when distant from hydrogen bonding sites. This series demonstrates the challenges in attempting to deconvolute a myriad of interdependent noncovalent interactions within a construct that resembles the complex environment of peptides and proteins. In particular, the interconnectedness of the N−H⋅⋅⋅O and C−H⋅⋅⋅O hydrogen bonds coupled with the interplay of different steric burdens, hydrogen‐acceptor/‐donor properties and rotational profiles make clear and unambiguous trends based solely on NMR chemical shift data difficult to rationalize.^32^

### Quantum chemical calculations

**Figure 6:**
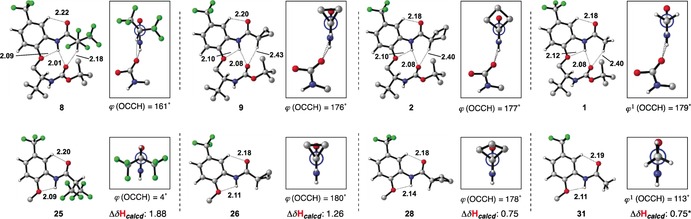
Optimized geometries of minima in the surfaces of **8**, **9**, **2** and **1** (and their respective controls **25**, **26**, **28** and **31**) with close contacts indicated (distances in Å). Chemical shift changes (Δ*δ*H_calcd_, ppm) were estimated by first calculating the chemical shifts for **8**, **9**, **2** and **1** in their energy minimized conformations and subtracting the chemical shifts calculated for optimized conformations of **25**, **26**, **28** and **31**, respectively. *: For **31** only one of two energetically equivalent minima are pictured, and Δ*δ* **H**_calcd_ is the mean of these two conformers (for **31**: *ϕ*(OCCH)=113°; Δ*δ*H_calcd_=0.45 ppm. *ϕ*(OCCH)=178°; Δ*δ*H_calcd_=1.06 ppm).

Our solution‐state and solid‐state analyses suggest a correlation between the steric and electronic properties of different Cα−**H** substituents and the propensity of those substituents to function as donors in C−H⋅⋅⋅O interactions. To further evaluate whether these observations—the close contacts observed in crystal structures and chemical shift changes seen by ^1^H NMR spectroscopy—are a consequence of an attractive C−H⋅⋅⋅O interaction we applied quantum calculation using the Gaussian 09 package at the M06‐2X/6‐31+G** level of theory.^33^ The geometry of compounds **8**, **9**, **2** and **1** and their respective controls **25**, **26**, **28** and **31** were probed by rotating the amide group about torsion angle *ϕ*(OCCH); fully optimized geometries of minima are presented above (Figure 6).^28^ These calculated geometries are in excellent agreement to those observed by X‐ray crystallography and exhibit only minor differences. For example: in α,α‐bistrifluoromethylamide **8**, the optimized conformation is remarkably similar to that seen in the crystal structure, with a short C−H⋅⋅⋅O distance of 2.18 Å and a *ϕ*(OCCH) torsion angle of 161° (Figure 2). This conformation is significantly different from that of its control **25**, which possesses a *ϕ*(OCCH) torsion angle of 4°.^34^ The minima illustrated for **9** and **2**, and their controls **26** and **28** have *ϕ*(OCCH) torsion angles close to 180°; only **28** has an additional minimum with *ϕ*(OCCH) close to 0° within 3 kcal mol^−1^. For **1** and **31**, rotation about the OCCH torsion from the illustrated minimum is effectively free, with other minima within 0.1 kcal mol^−1^. This appears to be consistent with a weak and inconsequential C−H⋅⋅⋅O interaction. In order to estimate the strength of these intramolecular interactions, we examined the second‐order perturbation energy *E*(2) for **8**, **9**, **2**, and **1** through natural bond orbital (NBO) analysis.^35^ This provides an estimation of the donor–acceptor interaction energy between the oxygen lone‐pair acceptor and the σ* orbital of the Cα−H donor. *E*(2) is equal to 5.08, 1.66, 2.00, and 1.61 kcal mol^−1^ for molecules **8**, **9**, **2**, and **1**, respectively, consistent with values normally observed for C−H⋅⋅⋅O hydrogen bonds.^36^ A second metric to estimate the strength of a C−H⋅⋅⋅O interaction is the ^1^H NMR deshielding of the bridging Cα−**H**.^17^ The calculated deshielding (Δ*δ*C**H**_calcd_) in **8**, **9**, **2**, and **1** (relative to controls **25**, **26**, **28** and **31**, respectively) was 1.88, 1.26, 0.75, and 0.45 ppm. These values are smaller than, but consistent with the order observed experimentally in **8**, **9**, **2**, and **1** but refer to a single, static conformation without thermal averaging. To give an indication of how much of this deshielding can be attributed to the C−H⋅⋅⋅O hydrogen bond, we compared the Cα−**H** chemical shift when in the optimized geometry (Figure 6) to that after rotating about the OCNH torsion by 180°. The deshielding of the Cα−**H** calculated in this manner is: 1.42, 0.67, 0.50, and 0.38 ppm, for **8**, **9**, **2**, and **1**, respectively. It is notable that all of the metrics outlined above suggest that the C−H⋅⋅⋅O interaction is strongest for **8** and least significant for **1**.^37^

**Figure 7:**
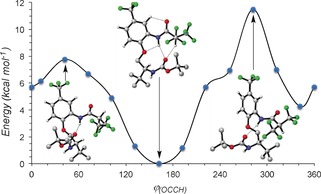
Computed rotational profiles of **8** as a function of dihedral angle *ϕ*(OCCH). Optimized structures are illustrated for important geometries.

Compound **8** appears to provide the clearest evidence for a conformationally influential solution and solid‐state C−H⋅⋅⋅O interaction. To further explore the relationship between energy and *ϕ*(OCCH) torsion, the terminal CH(CF_3_)_2_ group was rotated (in ca. 30° increments) around the *ϕ*(OCCH) torsion, generating a series of structures that were fully optimized, tracing out a potential energy curve as a function of dihedral angle (Figure 7). The profile shows a clear minimum at *ϕ*(OCCH)=161° and two maxima at *ϕ*(OCCH)=42° and *ϕ*(OCCH)=282°. In both of the maxima, the amide C=O is effectively *trans*‐planar to one of the CF_3_ groups, with the N−H⋅⋅⋅O hydrogen bond intact. In contrast, the minimum at *ϕ*(OCCH)=161° corresponds to the optimum geometry of the C−H⋅⋅⋅O hydrogen bond.

We subsequently quantified the strength of this interaction using an approach that has previously been applied to protein β‐sheets.^38^ Taking **8** and removing all atoms except those directly related to the C−H⋅⋅⋅O hydrogen bond left two fragments, which were frozen in their precise relative orientations. The energy of this complex was then compared with the sum of the energies of each isolated monomer, leading to a value of 3.6 kcal mol^−1^. This is a relatively strong interaction, consistent with the acidifying trifluoromethyl group, which, in combination with the torsional effects of geminal Cα‐substitution, led to the observed rotational minimum.

## Conclusions

Through a combined crystallographic, spectroscopic and quantum computational study we have probed the presence and influence of the C−H⋅⋅⋅O interaction in a series of β‐turn mimetics and demonstrated that electronic and torsional effects act in concert to modulate the potential influence of the C−H⋅⋅⋅O hydrogen bond. Considerably polarized Cα−**H** donor atoms and conformational restriction appear to be necessary for the C−H⋅⋅⋅O interaction to play a significant role in conformation. Our results also highlight the challenges in attempting to deconvolute a myriad of interdependent noncovalent interactions in order to focus on the contribution of a single one. Within a biomimetic construct designed to resemble the complexity of the environment within peptides and proteins, the interconnectedness of different hydrogen bonds coupled with the subtle interplay of steric burdens, hydrogen acceptor/donor properties and rotational profiles illustrate why unambiguous conclusions based solely on NMR chemical shift data are extremely challenging to rationalize, even for carefully calibrated model systems. These conclusions are directly relevant to foldamer and protein conformational preferences, especially β‐sheets,^13^ where α‐electronegative atoms can enhance Cα−H acidity and work in concert with conformational and torsional restriction provided by an extensive network of strong hydrogen bonds to potentiate weaker C−H⋅⋅⋅O interactions. In the context of designing and developing new folded systems and hydrogen‐bonding organocatalysts that exploit the C−H⋅⋅⋅O hydrogen bond as a structural element, it is clear that a combination of favourable electronic and torsional effects is a prerequisite for such interactions to be conformationally influential.^39^

## Experimental Section

Full synthetic procedures and complete spectroscopic data (including ^1^H and ^13^C NMR spectra) for all compounds are available in Supporting Information.

## Supporting information

As a service to our authors and readers, this journal provides supporting information supplied by the authors. Such materials are peer reviewed and may be re‐organized for online delivery, but are not copy‐edited or typeset. Technical support issues arising from supporting information (other than missing files) should be addressed to the authors.

Supplementary
