# Supplementary material for: Torsional and Electronic Factors Control the C−H⋅⋅⋅O Interaction
Source: Chemistry. 2016 Oct 6;22(46):16513–21. doi: 10.1002/chem.201602905 (PMC5113693; doi:10.1002/chem.201602905)
Supplement: Supplementary file 1 — Supplementary [file CHEM-22-16513-s001.pdf]

CHEMISTRY
A European Journal

Supporting Information

Torsional and Electronic Factors Control the C–H···O Interaction

Russell W. Driver,[a] Timothy D. W. Claridge,[a] Steve Scheiner,*[b] and Martin D. Smith*[a]

chem_201602905_sm_miscellaneous_information.pdf

Supporting information:

Torsional and Electronic Factors Control the C–H···O Interaction

Russell W. Driver,[a] T. D. W. Claridge,[a] Steve Scheiner,[b] & Martin D. Smith[a]*

[a] *Chemistry Research Laboratory, University of Oxford, 12 Mansfield Road, Oxford, UK.*
[b] *Department of Chemistry and Biochemistry, Utah State University, Logan, UT, USA.*

1. **General Synthetic Experimental Procedures.**

2. **Quantum Calculation**

3. **Experimental procedures, ^{1}H, ^{13}C and ^{19}F NMR spectra and characterization data**

1. General synthetic experimental procedures.

1.1 Solvents and Reagents

THF was distilled under an atmosphere of dry nitrogen from lithium aluminium hydride and calcium hydride in the presence of triphenylmethane; dichloromethane was distilled from calcium hydride; triethylamine was distilled from calcium hydride and stored over potassium hydroxide. pH 7 buffer was prepared by dissolving KH_2PO_4 (85 g) and NaOH (14.5 g) in distilled water (950 mL). Petrol refers to the fraction of petroleum ether boiling between 40 and 60 °C. Aldehydes were purified by distillation or recrystallization. All other reagents and solvents were used as supplied, without prior purification.

1.2 Chromatography

Thin layer chromatography (TLC) was performed on glass or aluminium plates coated with Merck 60 F254 silica and visualization was achieved by UV light or by staining with ninhydrin solution or potassium permanganate. Flash column chromatography was carried out using Merck Kieselgel (230-400 mesh).

1.3 Nuclear Magnetic Resonance Spectroscopy

NMR spectra were recorded on a Bruker Avance Cryo 500 (1H: 500 MHz and 13C: 125 MHz), a Bruker AVB 500 (1H: 500 MHz and 13C: 125 MHz) spectrometer. Chemical shifts are quoted in ppm and are referenced to the residual non-deuterated solvent peak, and are reported (based on appearance rather than interpretation) as follows: chemical shift δ/ppm (number of protons, multiplicity, coupling constant *J*/Hz, assignment) [br, broad; s, singlet; d, doublet; t, triplet; q, quartet; qui, quintet; sept, septet; m, multiplet].

1.4 Mass Spectrometry

Accurate mass measurements were performed on a Bruker microTOF (ES+) at the University of Oxford.

1.5 Polarimetry

Optical rotations were recorded on a Perkin-Elmer 241 polarimeter with a path length of 1 dm.

1.6 IR Spectroscopy

All measurements were obtained from neat materials. IR spectra were recorded on a Bruker Tensor 27 FTIR spectrometer equipped with an attenuated total reflectance attachment with internal calibration. Absorption maxima (ν_{max}) are quoted in wavenumbers (cm^{-1}).

1.7 Xray Crystallography

Crystals were mounted using the oil technique, in perfluoropolyether oil at 150(2) K (Cu and Mo) or 100(2) K (Synchrotron) with a Cryostream N_2 open-flow cooling device.

1.7.1 Molybdenum Radiation

Single crystal X-ray diffraction data were collected using graphite monochromated Mo-Ka radiation (0.71073 Å) using a Nonius KappaCCD diffractometer. Series of μ-scans were generally performed to provide sufficient data in each case to a maximum resolution of 0.77 Å. Data collection and cell refinement were carried out using DENZO-SMN Intensity data were processed and corrected for absorption effects by the multi-scan method, based on multiple scans of identical and Laue equivalent reflections using SCALEPACK (within DENZO-SMN). Structure solution was carried out with direct methods using the programs SIR9219 within the CRYSTALS software suite. Refinement was carried out using full-matrix least-squares within the CRYSTALS suite on F2. In general, all non-hydrogen atoms were refined with anisotropic displacement parameters. Hydrogen atoms were generally visible in the difference map and their positions and displacement parameters were refined using restraints prior to inclusion into the model using riding constraints.

1.7.2 Copper Radiation

Single crystal X-ray diffraction data were collected using graphite monochromated Cu K radiation (λ= 1.54184 Å) on an Oxford Diffraction SuperNova diffractometer. Series of ω-scans were performed in such a way as to collect all unique reflections to a maximum of 0.77 Å. Cell parameters and intensity data (including inter-frame scaling) were processed using CrysAlis Pro. The structures were solved by charge-flipping methods using SUPERFLIP and refined using full-matrix least-squares on F2 within the CRYSTALS suite. All non-hydrogen atoms were refined with anisotropic displacement parameters. Hydrogen atoms were generally visible in the difference map and their positions and displacement parameters were refined using restraints prior to inclusion into the model using riding constraints.

1.7.3 Synchrotron Radiation

Single crystal X-ray diffraction data were collected using silicon double crystal monochromated synchrotron radiation (λ = 0.68890 Å) at Diamond Light Source beamline I19 using a custom-built Rigaku diffractometer equipped with a Cryostream N_2 open-flow cooling device. The data were collected via a series of ω-scans that were performed in such a way as to cover a full-sphere of data to a maximum resolution of 0.77 Å. Cell parameters and intensity data (including inter-frame scaling) were processed using CrysAlis Pro.8. The structures were solved by charge-flipping methods using SUPERFLIP and refined using full-matrix least- squares on F2 within the CRYSTALS

suite. All non-hydrogen atoms were refined with anisotropic displacement parameters. Hydrogen atoms were generally visible in the difference map and their positions and displacement parameters were refined using restraints prior to inclusion into the model using riding constraints.

2.0 Quantum calculations

Calculations were carried out via the GAUSSIAN03 code.[1] The 6-31+G** double-ζ quality basis set contains polarization functions on all atoms, augmented by diffuse functions on heavy atoms; the larger and more flexible aug-cc-pVDZ basis set was also used. The B3LYP variant of density functional theory (DFT)[2] was used as it has had good success in the past with related molecules.[3] Complementary calculations were carried out with M06,[4] a recently developed functional with a focus on non-covalent interactions such as H-bonds. Second order Møller-Plesset (MP2) was used as a more complete means of including electron correlation. The self-consistent reaction field (SCRF) approach[5] was applied to study the effects of a surrounding polarizable medium, with a dielectric constant ε. The polarizable continuum method (PCM)[6] embeds the solute in a cavity that reproduces the shape of the molecule by a series of overlapping spheres. The particular variant of this method used here is the conductor polarized continuum model (CPCM)[7] wherein the apparent charges distributed on the cavity surface are such that the total electrostatic potential cancels on the surface. Recent calculations[8] have shown that the CPCM variant provides results that are in good agreement with other approaches, notably PCM and SCIPCM, in treating the C-H···O interaction as well as conventional H-bonds.

---

[1] Frisch, M. J.; Trucks, G. W.; Schlegel, H. B.; Scuseria, G. E.; Robb, M. A.; Cheeseman, J. R.; Zakrzewski, V. G.; Montgomery, J. J. A.; Stratmann, R. E.; Burant, J. C.; Dapprich, S.; Millam, J. M.; Daniels, A. D.; Kudin, K. N.; Strain, M. C.; Farkas, O.; Tomasi, J.; Barone, V.; Cossi, M.; Cammi, R.; Mennucci, B.; Pomelli, C.; Adamo, C.; Clifford, S.; Ochterski, J.; Petersson, G. A.; Ayala, P. Y.; Cui, Q.; Morokuma, K.; Malick, D. K.; Rabuck, A. D.; Raghavachari, K.; Foresman, J. B.; Cioslowski, J.; Ortiz, J. V.; Baboul, A. G.; Stefanov, B. B.; Liu, G.; Liashenko, A.; Piskorz, P.; Komaromi, I.; Gomperts, R.; Martin, R. L.; Fox, D. J.; Keith, T.; Al-Laham, M. A.; Peng, C. Y.; Nanayakkara, A.; Gonzalez, C.; Challacombe, M.; Gill, P. M. W.; Johnson, B.; Chen, W.; Wong, M. W.; Andres, J. L.; Gonzalez, C.; Head-Gordon, M.; Replogle, E. S.; Pople, J. A. Gaussian03; D.01 ed.; Gaussian, Inc.: Pittsburgh PA, **2003**.

[2] Becke, A. D. *J. Chem. Phys.* **1993**, *98*, 5648-5652; Lee, C.; Yang, W.; Parr, R. G. *Phys. Rev. B* **1988**, *37*, 785-789

[3] Rablen, P. R.; Lockman, J. W.; Jorgensen, W. L. *J. Phys. Chem. A* **1998**, *102*, 3782-3797; Rao, L.; Ke, H.; Fu, G.; Xu, X.; Yan, Y. *J. Chem. Theory Comput.* **2009**, *5*, 86-96; Esrafili, M. D.; Hadipour, N. L. *Mol. Phys.* **2011**, *109*, 2451-2460; Zvereva, E. E.; Shagidullin, A. R.; Katsyuba, S. A. *J. Phys. Chem. A* **2011**, *115*, 63-69; Plumley, J. A.; Dannenberg, J. J. *J. Comput. Chem.* **2011**, *32*, 1519-1527; Bühl, M.; Kilian, P.; Woollins, J. D. *ChemPhysChem.* **2011**, *12*, 2405-2408.

[4] Zhao, Y.; Truhlar, D. G. *Theor. Chem. Acc.* **2008**, *120*, 215-241.

[5] Onsager, L. *J. Am. Chem. Soc.* **1936**, *58*, 1486-1493; Wong, M. W.; Frisch, M. J.; Wiberg, K. B. *J. Am. Chem. Soc.* **1991**, *113*, 4776-4782; Wong, M. W.; Wiberg, K. B.; Frisch, M. *J. Chem. Phys.* **1991**, *95*, 8991-8998.

[6] Miertus, S.; Scrocco, E.; Tomasi, J. *Chem. Phys.* **1981**, *55*, 117-129; Miertus, S.; Tomasi, J. *J. Chem. Phys.* **1982**, *65*, 239-245; Mennucci, B.; Tomasi, J. *J. Chem. Phys*. **1997**, *106*, 5151-5198.

[7] Barone, V.; Cossi, M. *J. Phys. Chem. A* **1998**, *102*, 1995-2001.

[8] Scheiner, S.; Kar, T. *J. Phys. Chem. B* **2005**, *109*, 3681-3689.

3. Experimental procedures, ^{1}H and ^{13}C, ^{19}F NMR spectra and characterization data for all compounds.

3.1 Representative General Procedure: (amide bond formation)

Pyridine (0.2 mL) and N,N-dimethylaminopyridine (2 mg, 0.01 mmol) were added to stirring solution of *carboxylic acid* (71 mg, 0.6 mmol) in DCM (10 mL). The solution was cooled to 0˚C, EDCI·HCl (114 mg, 0.6 mmol) was added in one portion and the reaction stirred for 20 min. *Amine* (0.075 g, 0.2 mmol) was added in one portion. The reaction was allowed to stir overnight, slowly warming to room temperature before addition of water (5 mL). The mixture was concentrated *in vacuo* and the reaction diluted with ethyl acetate (100 mL), washed with aqueous saturated ammonium chloride solution (2 X 50 mL), aqueous sodium carbonate solution (1 X 50 mL), then saturated brine solution (1 X 50 mL). The organic phase was dried with $MgSO_4$ and filtered to yield a white solid. The compound was purified by flash column chromatography (15:1 PET:EtOAc) and subsequently recrystallized.

(S)-tert-butyl (3,3-dimethyl-1-(2-nitro-4-(trifluoromethyl)phenoxy)butan-2-yl)carbamate [S1][9]

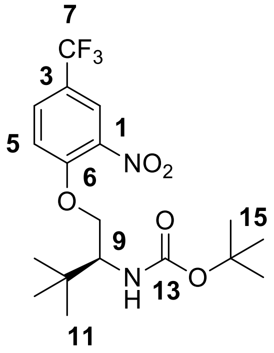

Potassium bis(trimethylsilyl)amide (0.5 M in toluene, 32 mL, 15.4 mmol) was added dropwise to a stirring solution of 4-fluoro-3-nitro-trifluoromethylbenzene (2.93 mL, 20.78 mmol) in THF (150 mL) at 0˚C. (S)-tert-butyl (1-hydroxy-3,3-dimethylbutan-2-yl)carbamate (1.75g, 8.3 mmol) in THF (30 mL) was added dropwise over 1 hour. The reaction was stirred at 0˚C for 30 min, then quenched with water (20 mL). The organic layer was concentrated *in vacuo* before being diluted with ethyl acetate (400 mL), then washed with aqueous saturated ammonium chloride solution (1 X 200mL), aqueous sodium carbonate solution (1 X 200mL), then saturated brine solution (1 X 100 mL). The organic phase was dried with $MgSO_4$ and filtered to yield a bright yellow oil. Compound purified by FC 8:1 PET:EtOAc. The resulting yellow solid was dissolved in pentane/ethyl acetate (10:1) and colorless needle crystals grown for X-ray studies (1.52 g, 49%).

δH (500 MHz, $CDCl_3$, 23˚C): 8.15 (1H, d, *J* 1.5 Hz, H2), 7.80 (1H, dd, J_1 9 Hz, J_2 2 Hz, H4), 7.21 (1H, d, *J* 8.7 Hz, H5), 5.04 (1H, d, *J* 9.6 Hz, H12), 4.33 (1H, dd, J_1 9 Hz, J_2 3 Hz, H8), 4.23 (1H, dd, J_1 9 Hz, J_2 4 Hz, H8), 3.83 (1H, m, H9), 1.43 (9H, s, H15), 1.01 (9H, s, H11).

δC (125 MHz, $CDCl_3$, 23˚C): 156.02 (C13), 154.67 (C6), 139.39 (C1), 131.43 (q, $^{3}J_{13C\text{-}19F}$ 4 Hz, C4), 123.75 (q, $^{3}J_{13C\text{-}19F}$ 4 Hz, C2), 123.31 (q, $^{2}J_{13C\text{-}19F}$ 32 Hz, C3), 123.19 (q, $^{1}J_{13C\text{-}19F}$ 271 Hz, C7), 114.88 (C5), 79.85 (C14), 69.82 (C8), 57.02 (C9), 34.61 (C10), 28.53 (C15), 27.31 (C11).

δF (282 MHz, $CDCl_3$, 23˚C): -62.47

HRMS: (ES+): found 429.1601; Formula $C_{18}H_{25}F_3N_2NaO_5$, [M + H+] requires 429.1608.

ν_{max} (neat, cm^{-1}): 3335.41, 2964.66, 2871.71, 2163.61, 1980.68, 1677.60, 1649.87, 1628.30, 1586.31, 1535.23, 1479.11, 1468.22, 1392.00, 1367.51, 1348.13, 1324.38, 1293.23, 1278.06, 1248.20, 1173.10, 1159.40, 1126.97, 1092.88, 1063.02, 1035.04, 1011.74, 999.08, 939.69, 923.96, 902.97, 895.78, 863.43, 836.27, 785.06, 758.84, 741.92, 721.26, 684.68, 633.23.

MP: 134˚C.

$[\alpha]_D^{25.0}$ +77.0 (c = 0.001, $CDCl_3$).

[9] [9] C. R. Jones, G. D. Pantos, A. J. Morrison & M. D Smith, *Angew. Chem. Int. Ed.,* **2009**, *48*, 7391-7394.

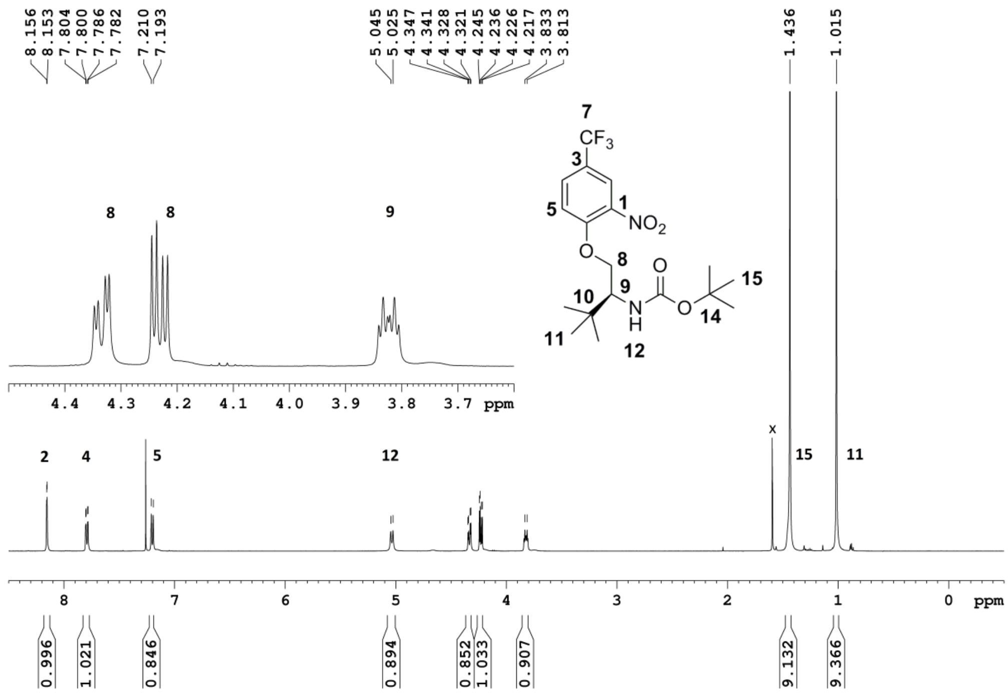

^{1}H NMR spectrum (23˚C, $CDCl_3$, 500 MHz).

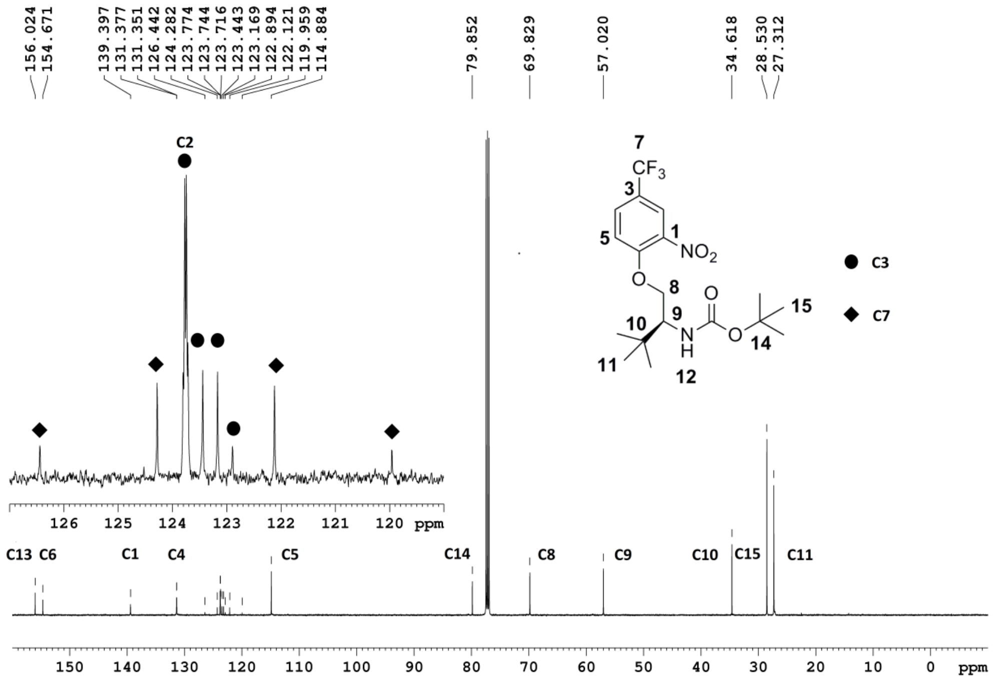

^{13}C NMR spectrum (23˚C in $CDCl_3$, 125 MHz).

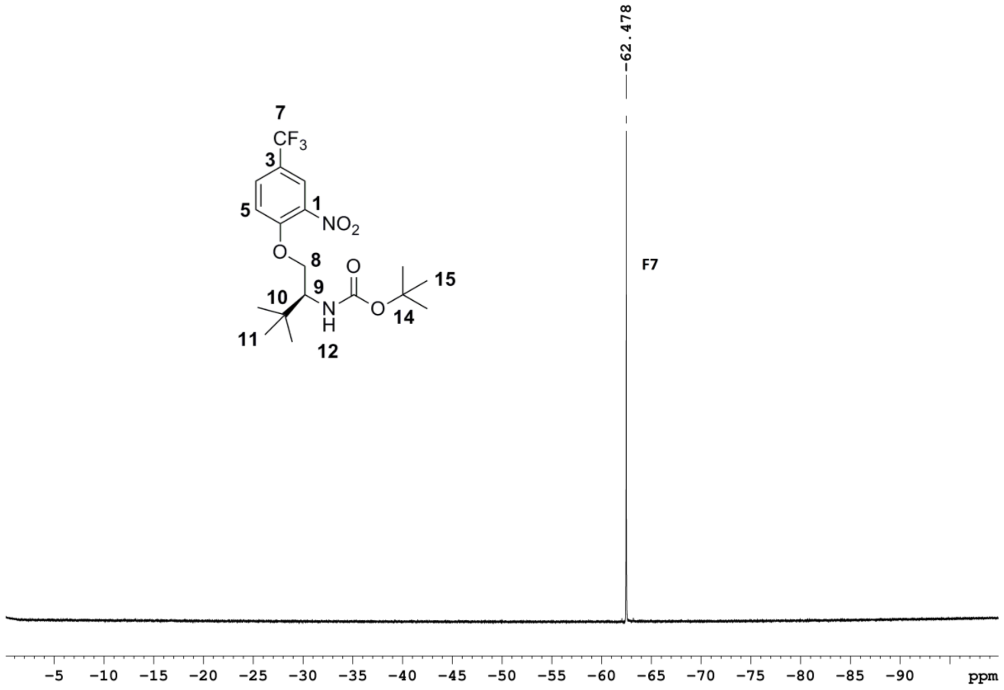

^{19}F NMR spectrum (23˚C, $CDCl_3$, 282 MHz).

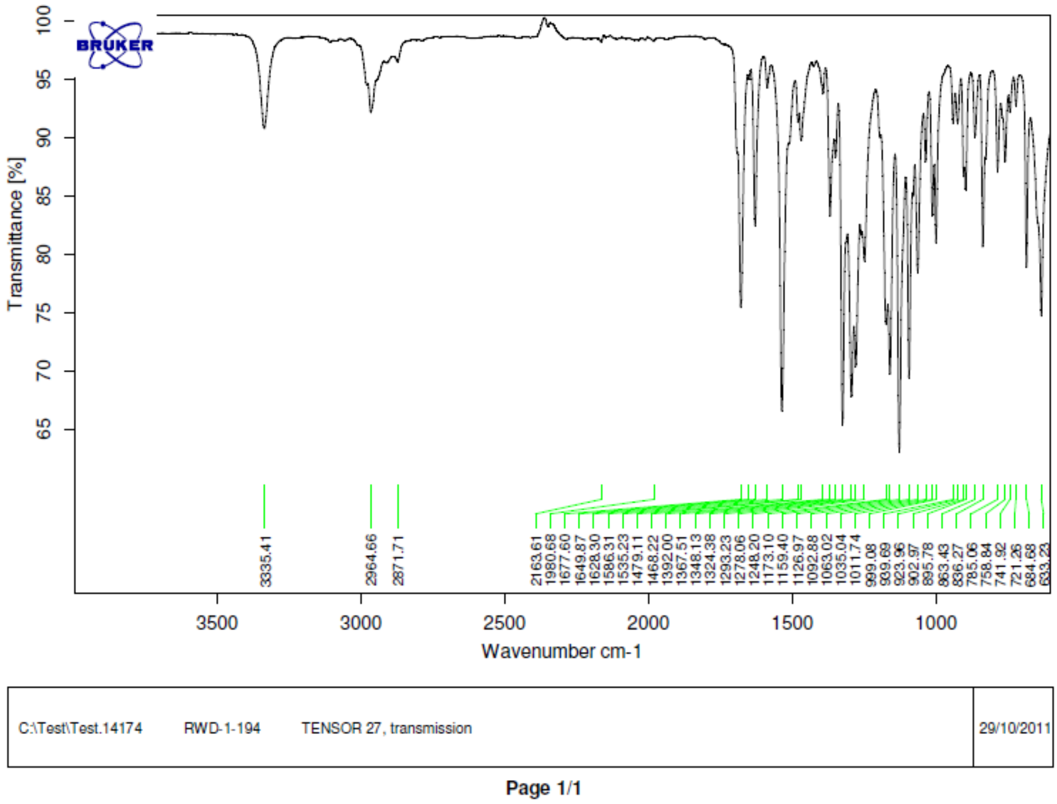

FT-IR of diffraction quality crystals.

Mass Spectrum SmartFormula Report

**Analysis Info**			
Analysis Name	\\Utof\Data\Oct 11\ESI30893_17_01_34086.d	Acquisition Date	31/10/2011 08:12:52
Method	2.5min_cal_sample_pos_Naf_11-10-10.m	Operator	Mass Spec
Sample Name	ESI30893	Instrument / Ser#	micrOTOF 92
Comment			

**Acquisition Parameter**					
Source Type	ESI	Ion Polarity	Positive	Set Nebulizer	2.0 Bar
Focus	Not active			Set Dry Heater	180 °C
Scan Begin	100 m/z	Set Capillary	4500 V	Set Dry Gas	10.0 l/min
Scan End	1500 m/z	Set End Plate Offset	-500 V	Set Divert Valve	Source

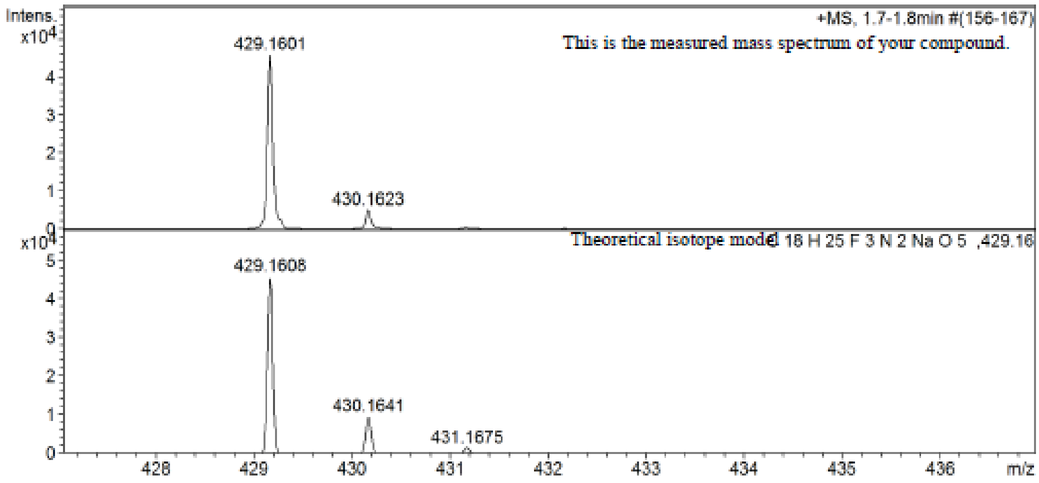

Meas. m/z	#	Formula	m/z	err [ppm]	Mean err [ppm]	rdb	e⁻ Conf	mSigma
429.1601								
	1	C 18 H 25 F 3 N 2 Na O 5	429.1608	1.5	1.8	5.5	even	55.69

High resolution mass spectrum of **S1**.

(S)-tert-butyl (1-(2-amino-4-(trifluoromethyl)phenoxy)-3,3-dimethylbutan-2-yl)carbamate [S3][10]

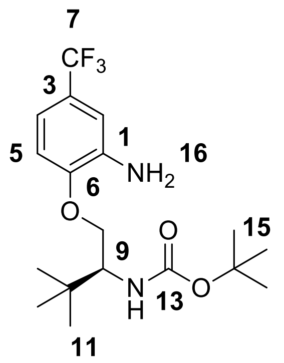

Ammonium formate (750 mg, 11.9 mmol) was added to a stirring suspension of (S)-tert-butyl (3,3-dimethyl-1-(2-nitro-4-(trifluoromethyl)phenoxy)butan-2-yl)carbamate (0.5 g, 1.2 mmol) and 10% Pd/C (75 mg) in methanol (20 mL) and ethyl acetate (3 mL). The yellow solution became clear almost instantly. The reaction was stirred for 20 minutes, then the palladium was removed by filtration (Celite: eluent methanol). The solvent was removed in vacuo and the resulting white solid dissolved in 200mL ethyl acetate. The organic phase was washed with sat. aqueous ammonium formate (50 mL X 2), sat. sodium carbonate (50mL X 2) and brine (50mL X 2). The organic phase was dried with $MgSO_4$, then concentrated to yield 450 mg (1.2 mmol, 100%) of the target molecule.

δH (500 MHz, $CDCl_3$, 23˚C): 6.93 (1H, d, *J* 9 Hz, H4), 6.89 (1H, s, H2), 6.74 (1H, *J* 8 Hz, H5), 4.65 (1H, d, *J* 9 Hz, H12), 4.19 (1H, m, H8), 4.03 (2H, bs, H16), 3.88 (2H, m, H8, H9), 1.44 (9H, H15), 1.02 (9H, H11).

δC (125 MHz, $CDCl_3$, 23˚C): 156.50 (C13), 148.74 (C6), 137.09 (C1), 124.88 (C7, q, $^{1}J_{13C\text{-}19F}$ 270.64), 123.71 (C3, q, $^{2}J_{13C\text{-}19F}$ 32 Hz), 115.21 (C4), 110.72 (C2), 110.51 (C5), 79.64 (C14), 68.99 (C8), 58.25 (C9), 33.79 (C15), 28.60 (C15), 27.16 (C11).

δF (470 MHz, $CDCl_3$, 23˚C): -62.12 (F7).

ν_{max} (neat, cm^{-1}): 3424.69, 3400.12, 3236.58, 3057.10, 2964.35, 2918.60, 2849.65, 1740.25, 1654.57, 1618.51, 1587.49, 1538.65, 1517.91, 1493.39, 1476.11, 1453.43, 1437.06, 1368.37, 1341.49, 1329.67, 1292.70, 1244.49, 1220.67, 1189.21, 116.89, 1138.52, 1098.14, 1075.65, 1042.71, 1026.40, 975.88, 943.99, 927.93, 899.51, 874.56, 849.15, 820.26, 802.16, 755.59, 736.85, 722.05, 699.34, 652.17, 636.77, 628.50, 606.88.

MP: 118-120˚C.

HRMS: (ES+): found (399.1857); $C_{18}H_{27}F_3N_2NaO_3$, [M + H+] requires 399.1866.

$[\alpha]_D^{25.0}$ +15° (c = 0.01, $CHCl_3$).

---

[10] [10] C. R. Jones, G. D. Pantos, A. J. Morrison & M. D Smith, *Angew. Chem. Int. Ed.,* **2009**, *48*, 7391-7394.

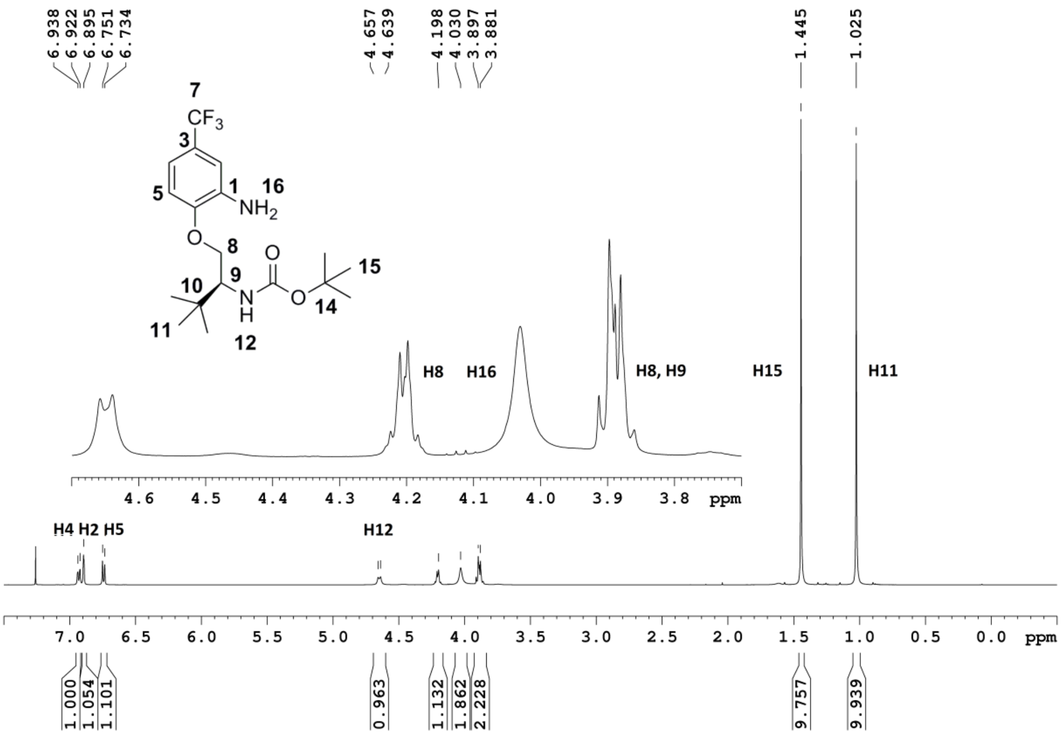

^{1}H NMR spectrum (23˚C, $CDCl_3$, 500 MHz).

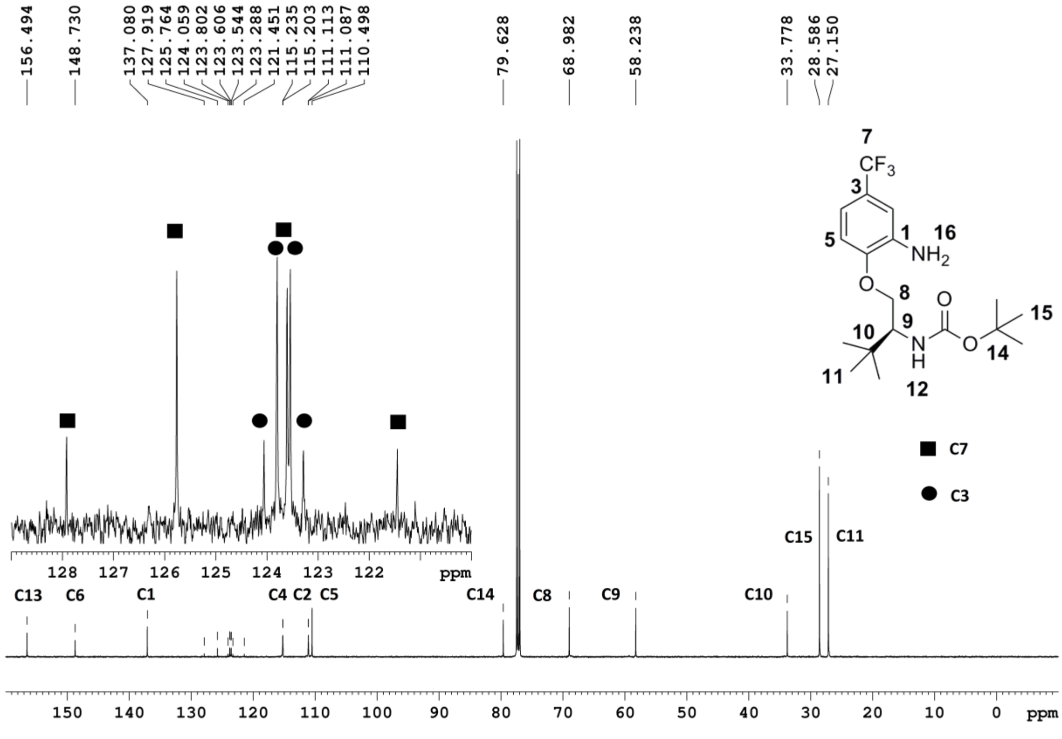

^{13}C NMR spectrum ($CDCl_3$, 23˚C, 125 MHz).

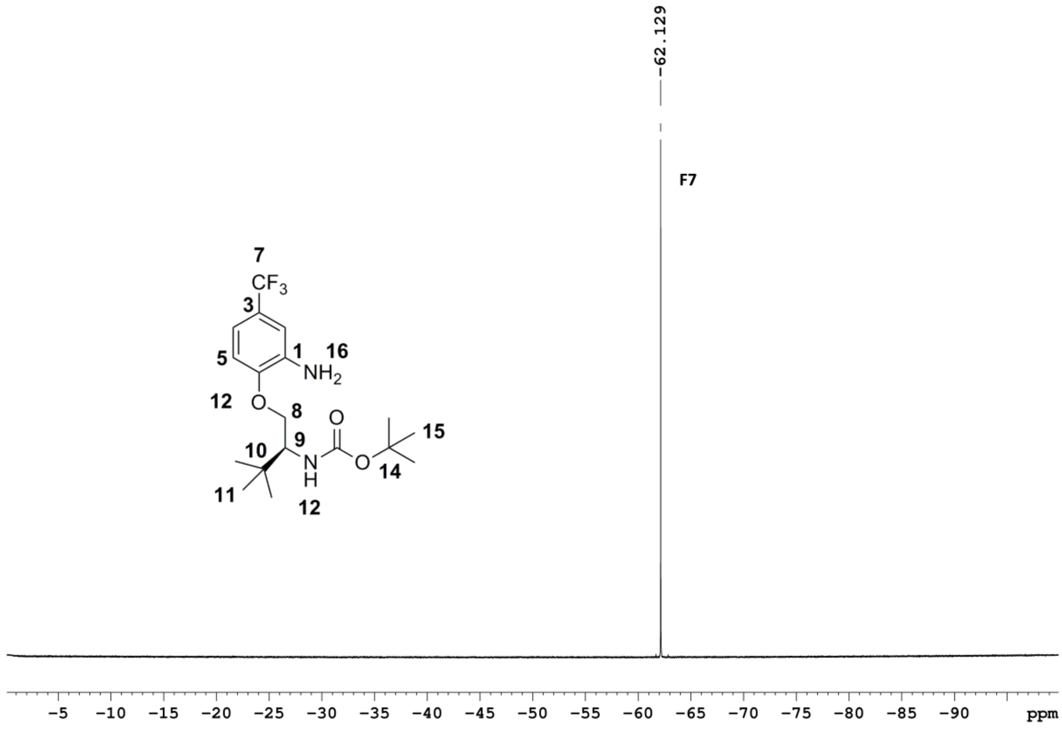

^{19}F NMR spectrum (cDCl$_3$, 23˚C, 470 MHz).

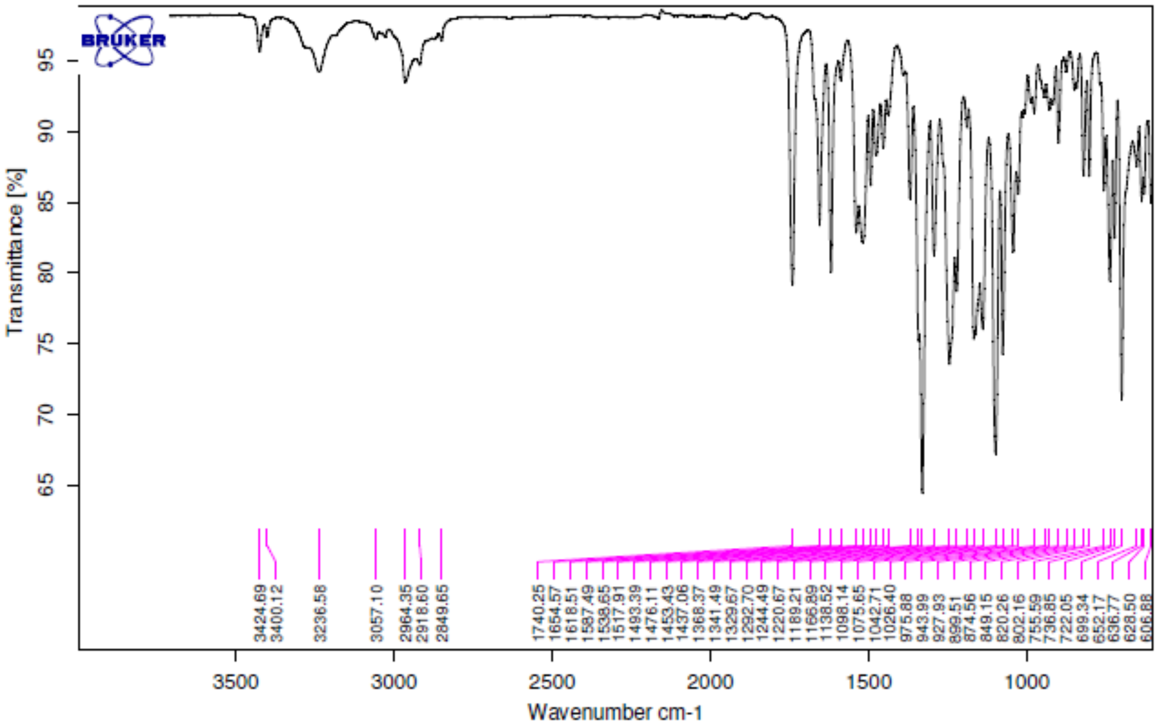

Solid state FT-IR of diffraction quality crystals.

Mass Spectrum SmartFormula Report

Analysis Info

Analysis Name	\\Utof\Data\Nov 11\ESI31042_13_01_34394.d	Acquisition Date	09/11/2011 07:58:59
Method	2.5min_cal_sample_pos_Naf_11-10-10.m	Operator	Mass Spec
Sample Name	ESI31042	Instrument / Ser#	micrOTOF 92
Comment			

Acquisition Parameter

Source Type	ESI	Ion Polarity	Positive	Set Nebulizer	2.0 Bar
Focus	Not active			Set Dry Heater	180 °C
Scan Begin	100 m/z	Set Capillary	4500 V	Set Dry Gas	10.0 l/min
Scan End	1500 m/z	Set End Plate Offset	-500 V	Set Divert Valve	Source

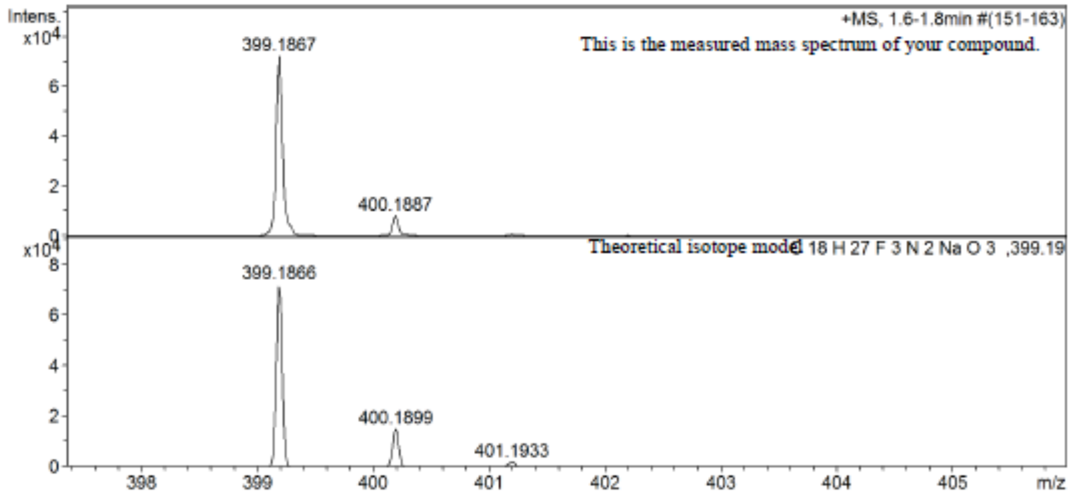

Meas. m/z	#	Formula	m/z	err [ppm]	Mean err [ppm]	rdb	e⁻ Conf	mSigma
399.1867								
	1	C 18 H 27 F 3 N 2 Na O 3	399.1866	-0.3	0.1	4.5	even	53.62

High resolution mass spectrum.

tert-butyl (1-(2-acetamido-4-(trifluoromethyl)phenoxy)-3,3-dimethylbutan-2-yl)carbamate [1][11]

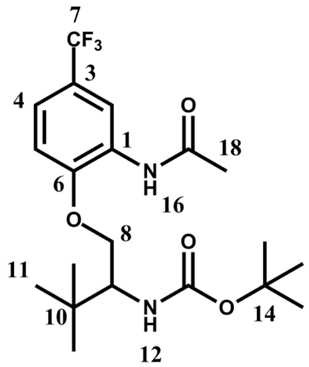

Prepared according to the representative procedure from racemic tert-butyl (1-(2-amino-4-(trifluoromethyl)phenoxy)-3,3-dimethylbutan-2-yl)carbamate on a 0.1 mmol scale. Chromatography (silica gel, petroleum ether : ethyl acetate 6:1), 31mg, 78% as a white solid.

δH (200 MHz, C_6D_6): 9.55 (s, H2), 8.68 (H16), 7.27 (obscured by solvent, H4), 6.14 (1H, d, *J* 7 Hz, H5), 4.34 (1H, d, *J* 10 Hz, H12), 3.81 (1H, t, *J* 8 Hz, H9), 3.60 (1H, dt, J_1 7 Hz J_2 2 Hz, H8), 3.16 (1H, apparent triplet, *J* 9 Hz, H8), 2.23 (3H, s, H18), 1.36 (9H, s, H15), 0.58 (9H, s, H11).

δ_H (400 MHz, $CDCl_3$): 8.77 (1H, d, *J* 1.4, Ar-H^3), 8.58 (1H, br s, C^4NH), 7.24 (1H, dd, *J* 8.4, 1.4, Ar-H^7), 6.81 (1H, d, *J* 8.4, Ar-H^6), 4.66 (1H, d, *J* 8.6, N*H*Boc), 4.03 (1H, dd, *J* 14.3, 8.0, CH_2OAr), 3.93-3.86 (2H, m, CH_2OAr & C*H*NHBoc), 2.28 (3H, s, $COCH_3$), 1.44 (9H, s, $CO_2C(CH_3)_3$), 1.05 (9H, s, $C(CH_3)_3$).

δ_C (125 MHz, $CDCl_3$): 169.3 (C=O amide), 157.0 (C=O carbamate), 149.3 (C^5), 128.9 (C^4), 124.2 (q, J_{C-F} 269.9, C^1), 123.4 (q, J_{C-F} 32.4, C^2), 120.2 (q, J_{C-F} 4.0, C^7), 116.5 (q, J_{C-F} 3.4, C^3), 109.8 (C^6), 79.7 ($CO_2\mathit{C}(CH_3)_3$), 70.2 ($\mathit{C}H_2$OAr), 58.8 (*C*HNHBoc), 32.7 ($\mathit{C}(CH_3)_3$), 28.4 ($CO_2C(\mathit{C}H_3)_3$), 26.9 ($C(\mathit{C}H_3)_3$), 24.6 ($CO\mathit{C}H_3$);

ν_{max} (neat, cm^{-1}): 3325 (br) (N-H), 2969 (m) (C-H), 1681 (s) (C=O), 1604 (m) (N-H), 1542 (s) & 1492 (m) (Ar C=C), 1272 (s) & 1118 (s) (C-O).

HRMS calcd for $C_{20}H_{29}F_3N_2O_4$ $[M+H]^+$: 419.2152; found: 419.2155.

MP: 126-127 ˚C.

[11] C. R. Jones, G. D. Pantos, A. J. Morrison & M. D Smith, *Angew. Chem. Int. Ed.,* **2009**, *48*, 7391-7394.

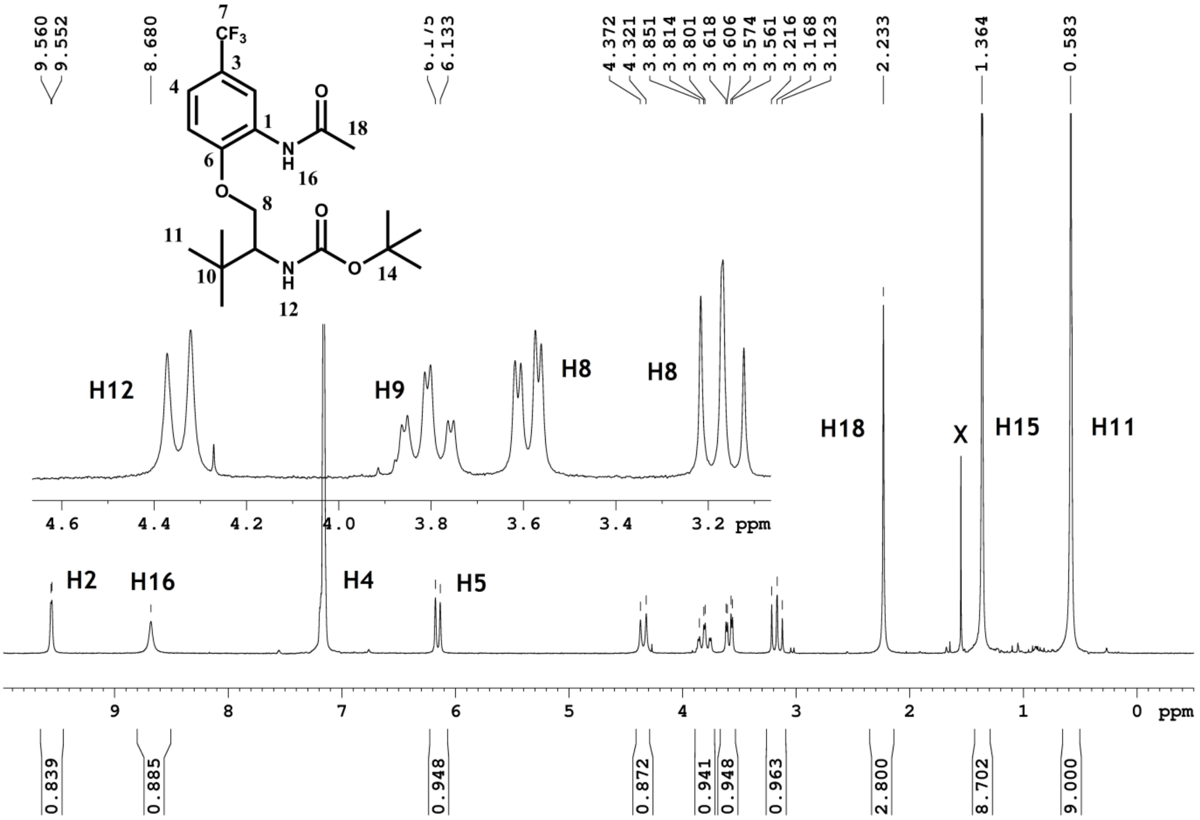

^{1}H NMR spectrum of **1** (23˚C, C_6D_6, 200 MHz).

(rac)-tert-butyl (1-(2-(cyclobutanecarboxamido)-4-(trifluoromethyl)phenoxy)-3,3-dimethylbutan-2-yl)carbamate [2]

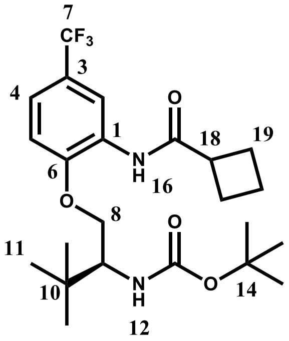

Prepared according to the representative procedure from racemic tert-butyl (1-(2-amino-4-(trifluoromethyl)phenoxy)-3,3-dimethylbutan-2-yl)carbamate on a 0.20 mmol scale. Chromatography (4:1 PET:EtOAc) to yield a white solid (10 mg, 11%) for X-ray crystallographic and NMR studies.

δH (500 MHz, C_6D_6, 23˚C): 9.63 (1H, s, H2), 8.47 (1H, s, H16), 7.17 (1H, dd, J_1 8 Hz, J_2 1 Hz, H4), 6.17 (1H, d, *J* 8 Hz, H5), 4.31 (1H, d, *J* 10 Hz, H12), 3.81 (1H, t, *J* 8 Hz, H9), 3.58 (1H, d, *J* 8 Hz, H8), 3.51 (1H, pentet, *J* 8 Hz, H18), 3.18 (1H, m, H8), 2.69 (1H, m, H19), 2.50 (1H, m, H19), 2.21 (2H, m, H19), 1.85 (2H, m, H20), 1.40 (9H, s, H15), 0.59 (9H, s, H11).

δC (125 MHz, C_6D_6, 23˚C): 174.07 (C17), 157.21 (C13), 149.94 (C6), 130.35 (C1), 125.74 (q, $^1J_{13C\text{-}19F}$ 272 Hz, C7), 124.28 (q, $^2J_{13C\text{-}19F}$ 32 Hz, C3), 120.26 (q, $^3J_{13C\text{-}19F}$ 4 Hz, C4), 117.45 (q, $^3J_{13C\text{-}19F}$ 3 Hz, C2), 110.56 (C5), 79.72 (C14), 70.15 (C8), 59.04 (C9), 41.66 (C18), 32.97 (C10), 28.73 (C11), 26.91 (C15), 26.52 (C19), 25.60 (C19), 19.03 (C20).

δF (370 MHz, C_6D_6, 23˚C): -61.14.

HRMS: (ES-): found 481.2288; Formula $C_{23}H_{33}F_3N_2O_4Na$, [M + Na] requires 481.2285.

ν_{max} (neat, cm^{-1}): 3334.58, 2967.34, 1685.27, 1603.36, 1538.71, 1490.06, 1439.48, 1367.01, 1338.05, 1270.40, 1214.96, 1164.85, 1120.86, 1063.93, 1012.50, 919.17, 812.39, 639.29.

MP: 133-134˚C.

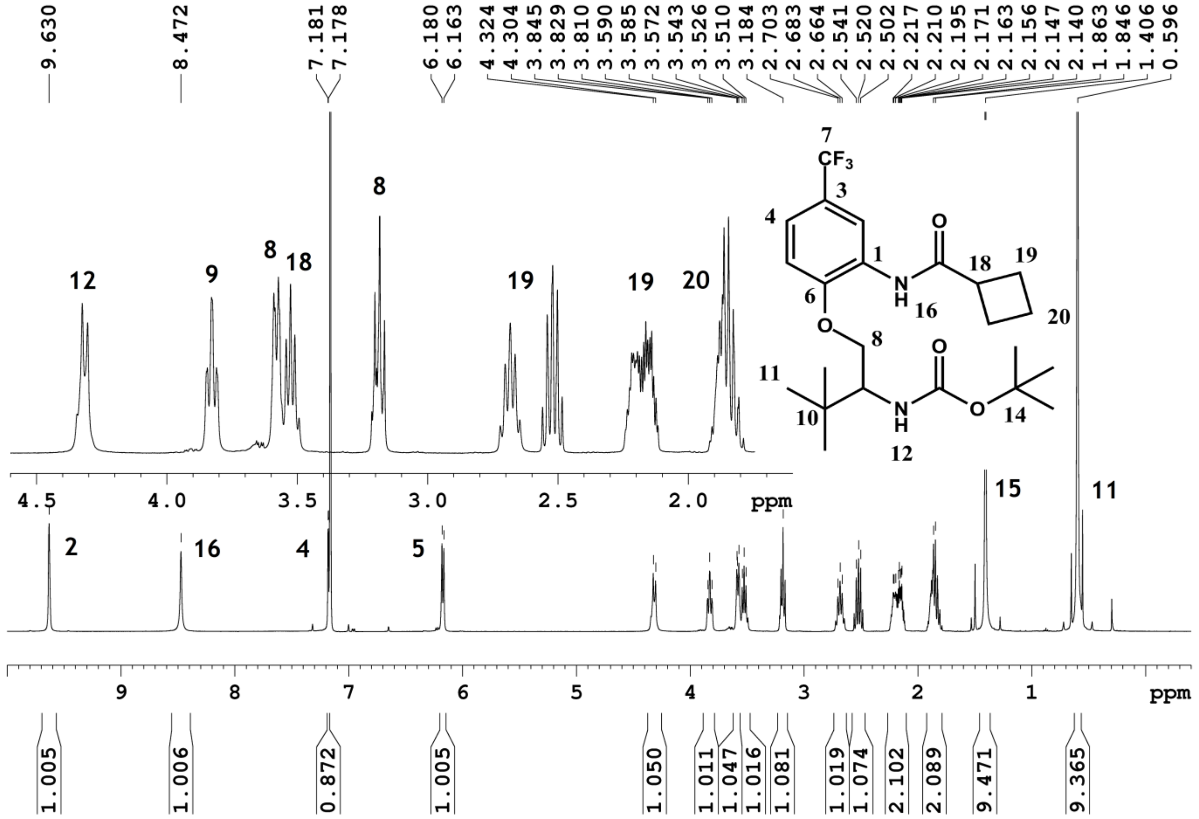

^{1}H NMR spectrum of **2** (23˚C, C_6D_6, 500 MHz).

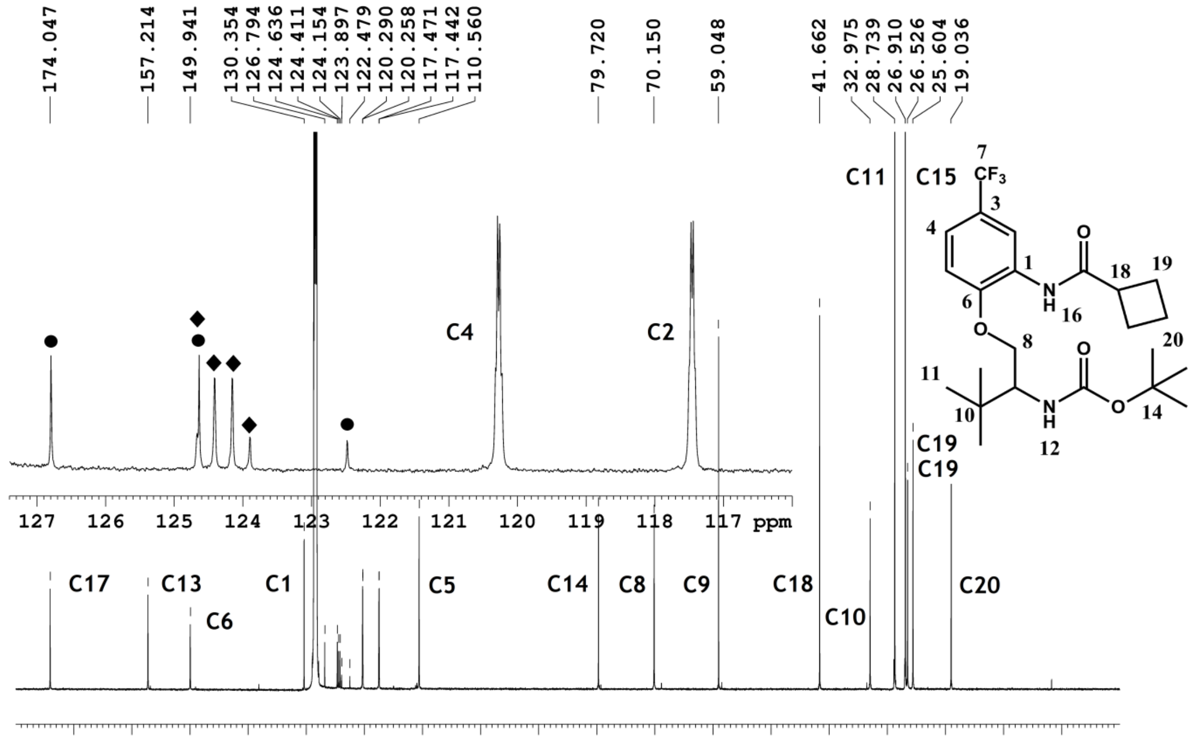

^{13}C NMR spectrum of **2** (23˚C in C_6D_6, 125MHz).

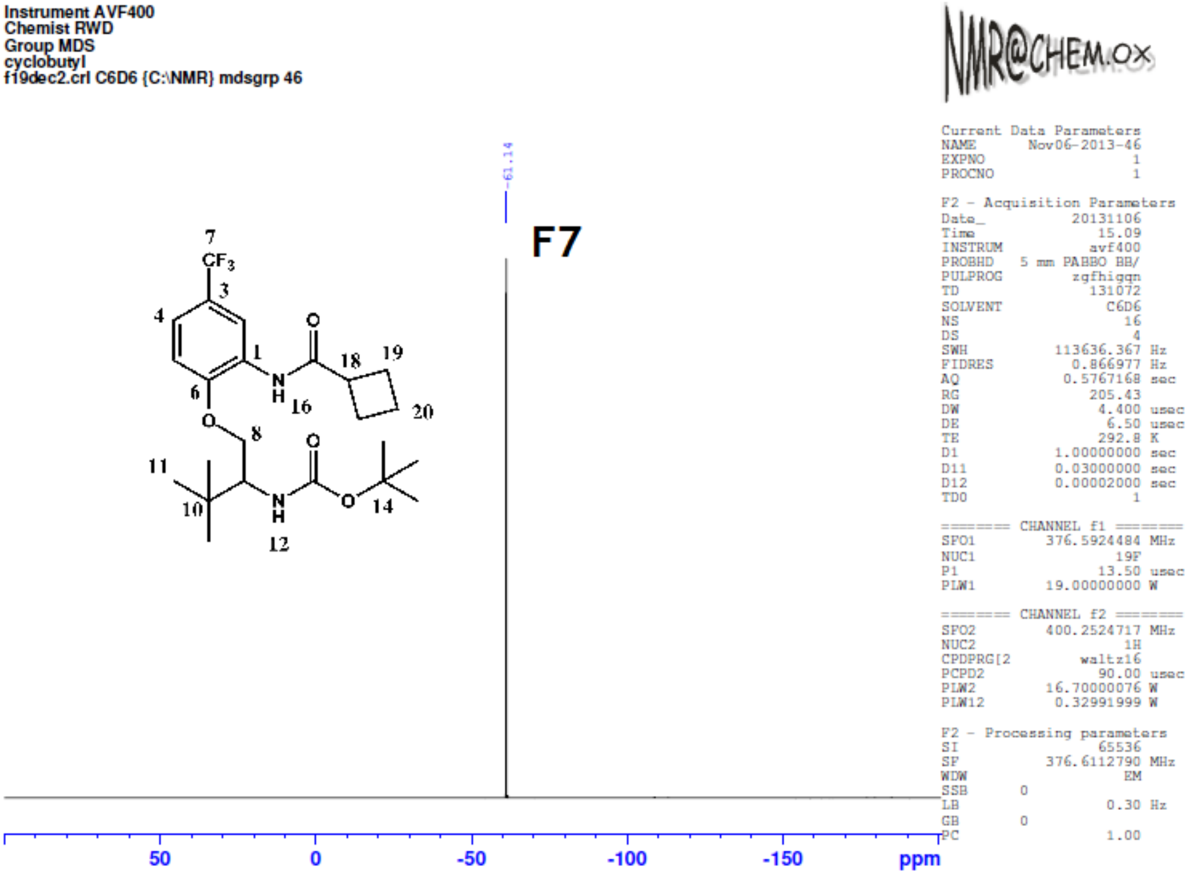

^{19}F NMR spectrum of **2** (23˚C in C_6D_6, 376 MHz).

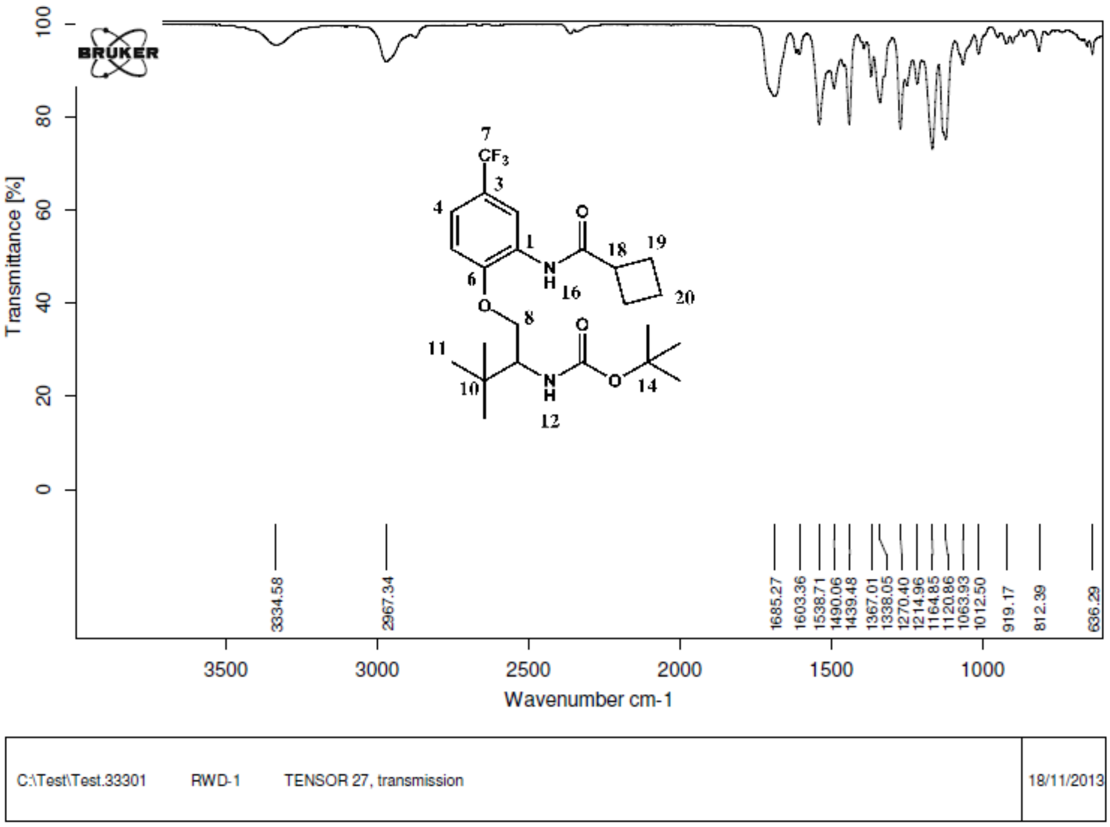

FT-IR of **2**.

Mass Spectrum SmartFormula Report

**Analysis Info**			
Analysis Name	\\Utof\data\Nov 13\ESI43501_6_01_14155.d	Acquisition Date	14/11/2013 08:02:33
Method	2.5min_cal_sample_pos_naf_05-08-13.m	Operator	Mass Spec
Sample Name	ESI43501	Instrument / Ser#	micrOTOF 92
Comment			

**Acquisition Parameter**					
Source Type	ESI	Ion Polarity	Positive	Set Nebulizer	2.0 Bar
Focus	Not active			Set Dry Heater	180 °C
Scan Begin	100 m/z	Set Capillary	4500 V	Set Dry Gas	10.0 l/min
Scan End	1000 m/z	Set End Plate Offset	-500 V	Set Divert Valve	Source

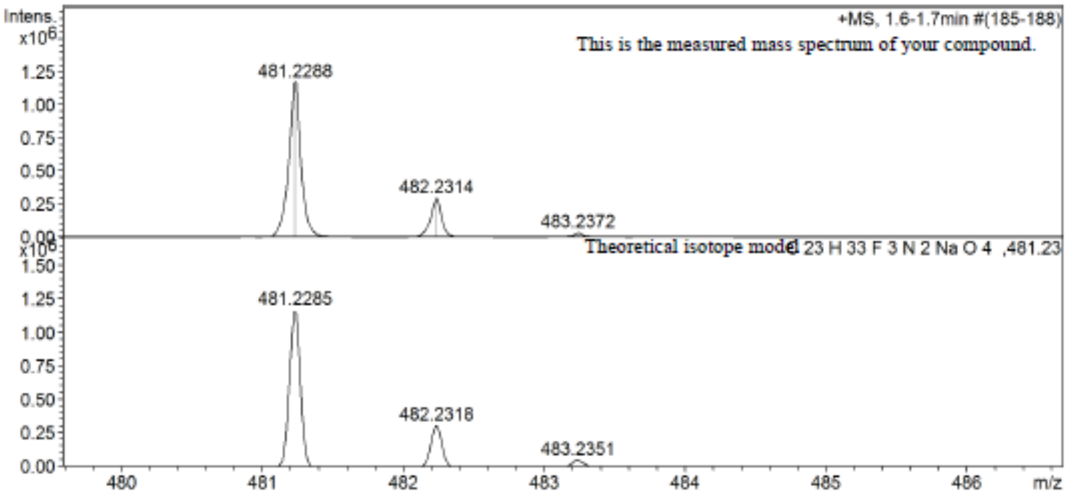

Meas. m/z	#	Formula	m/z	err [ppm]	Mean err [ppm]	rdb	e⁻ Conf	mSigma
481.2288								
	1	C 23 H 33 F 3 N 2 Na O 4	481.2285	-0.6	-0.4	6.5	even	6.16

High resolution mass spectrum of **2**.

(rac)-tert-butyl 3-((2-(2-((tert-butoxycarbonyl)amino)-3,3-dimethylbutoxy)-5-(trifluoromethyl)phenyl)carbamoyl)azetidine-1-carboxylate [3]

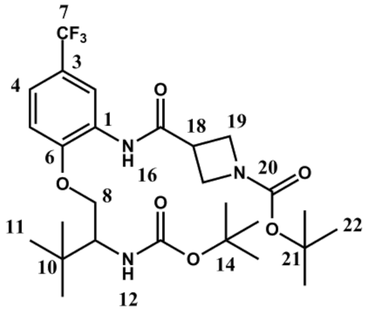

Prepared according to the representative procedure from racemic tert-butyl (1-(2-amino-4-(trifluoromethyl)phenoxy)-3,3-dimethylbutan-2-yl)carbamate on a 0.2 mmol scale. Chromatography (silica gel, petroleum ether : ethyl acetate 3:1), 32mg, 28% as a white solid.

δH (500 MHz, C_6D_6, 23˚C): 9.50 (1H, s, H2), 8.70 (1H, s, H16), 7.18 (1H, dd, J_1 8 Hz, J_2 1 Hz, H4), 6.16 (1H, d, J 8 Hz, H5), 4.53 (1H, m, H19), 4.40 (2H, m, H19, H12), 4.15 (1H, m, H19), 4.04 (1H, m, H19), 3.74 (1H, t, J 8 Hz, H9), 3.59 (2H, m, H8, H18), 3.19 (1H, m, H8), 1.54 (9H, s, H15), 1.30 (9H, s, H22), 0.58 (9H, s, H11).

δC (125 MHz, C_6D_6, 23˚C): 171.34 (C17), 157.53 (C13), 156.80 (C20), 150.11 (C6), 129.87 (C1), 125.59 (q, $^1J_{13C\text{-}19F}$ 271 Hz, C7), 124.30 (dq, $^2J_{13C\text{-}19F}$ 32 Hz, J 4 Hz, C3), 120.88 (C4), 117.69 (C2), 110.80 (C5), 79.88 (C21), 79.57 (C14), 70.56 (C8), 59.31 (C9), 52.67 (C19), 35.01 (C18), 32.68 (C10), 28.81 (C15), 28.62 (C22), 26.90 (C11).

δF (370 MHz, C_6D_6, 23˚C): -61.21.

HRMS: (ES+): found 582.2766; Formula $C_{27}H_{40}F_3N_3O_6Na$, [M + H+] requires 582.2761.

ν_{max} (neat, cm^{-1}): 3321.94, 2970.80, 1683.75, 1615.11, 1543.45, 1493.28, 1440.75, 1414.70, 1366.87, 1340.34, 1271.64, 1218.13, 1163.88, 1121.77, 1064.30, 1012.08, 922.36, 900.46, 858.18, 813.55, 775.52, 636.42.

MP: 195˚C.

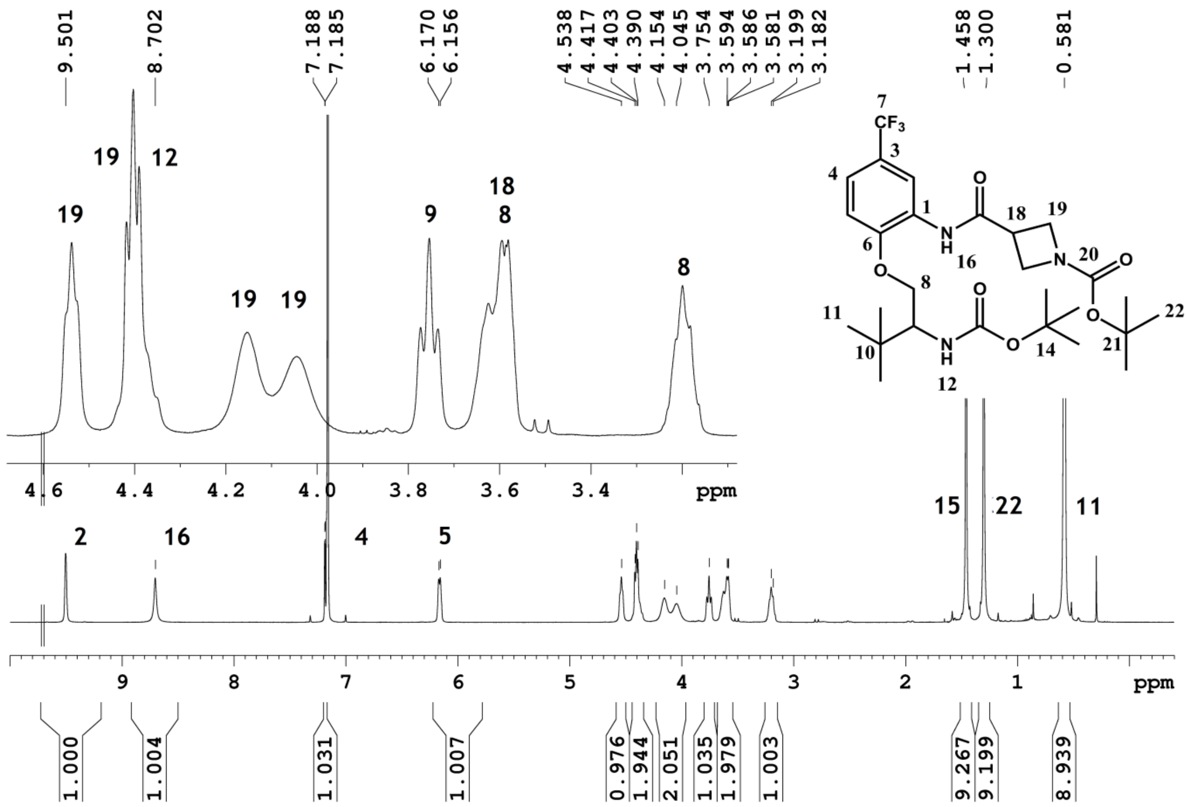

^{1}H NMR spectrum of **3** (23˚C in C_6D_6, 500MHz).

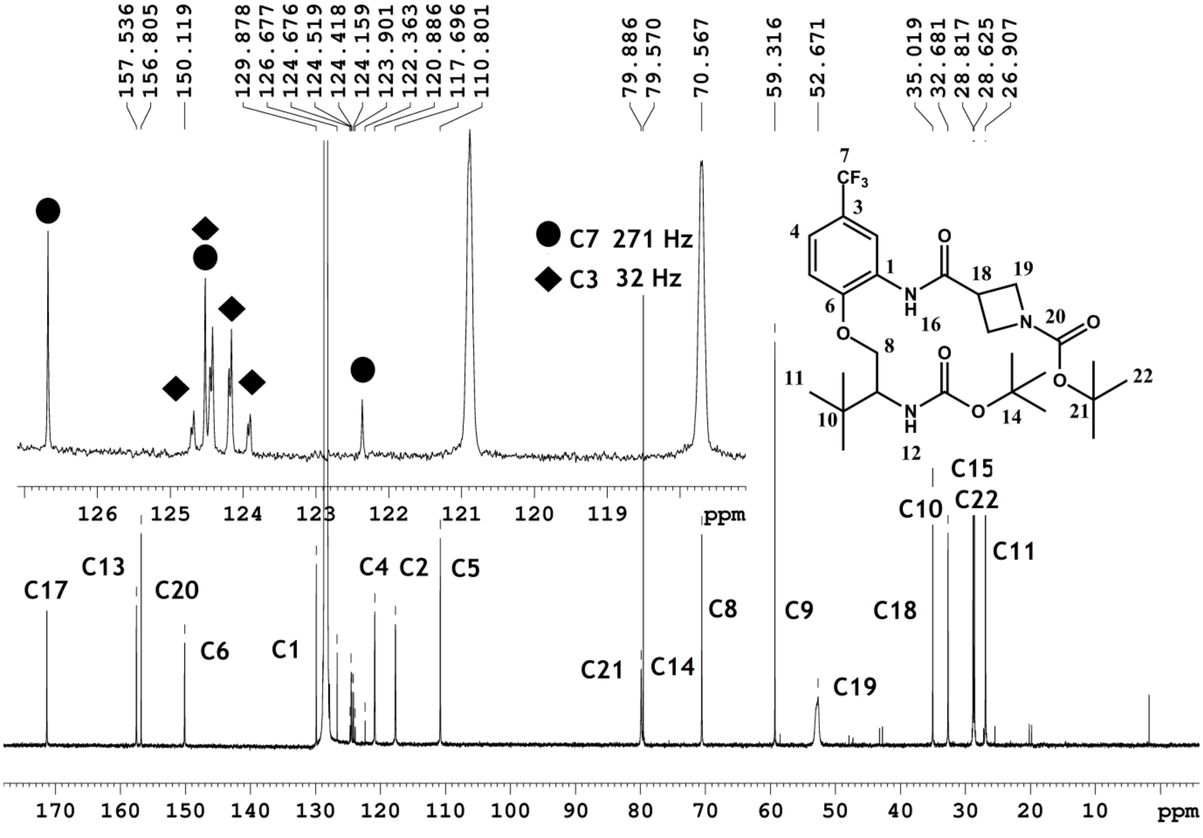

^{13}C NMR spectrum of **3** (23˚C, C_6D_6, 125MHz).

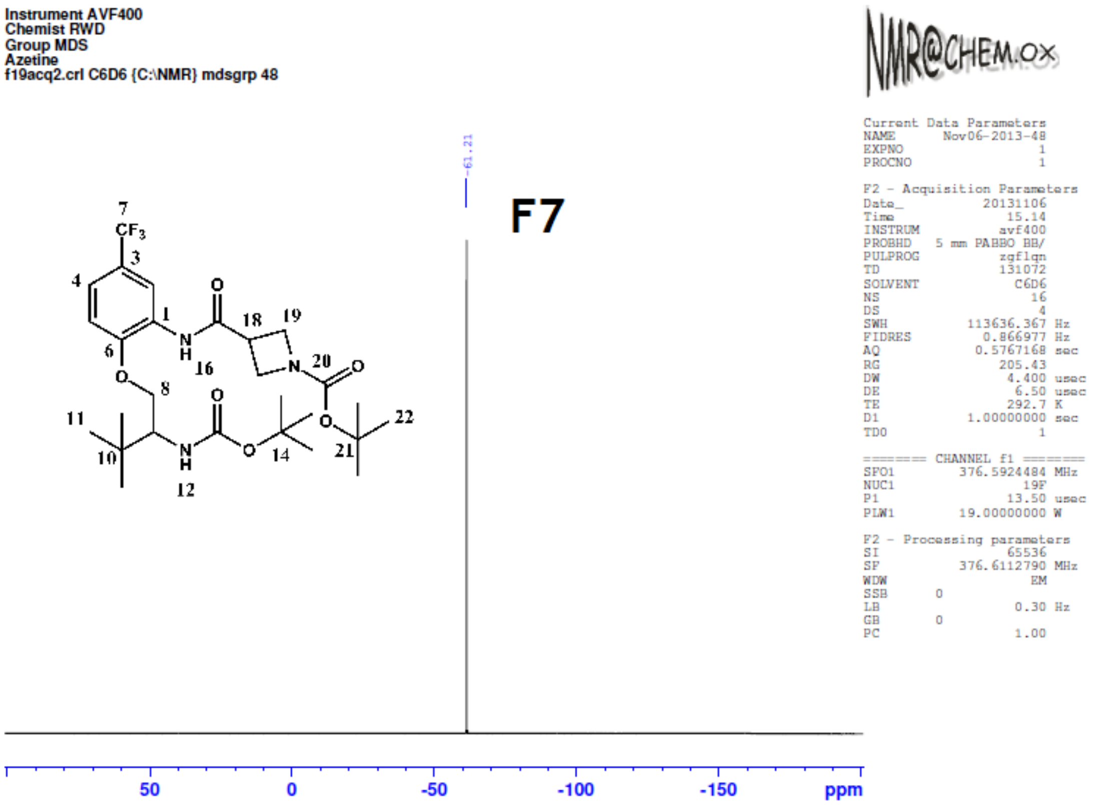

^{19}F NMR spectrum of **3** (23˚C, C_6D_6, 376 MHz).

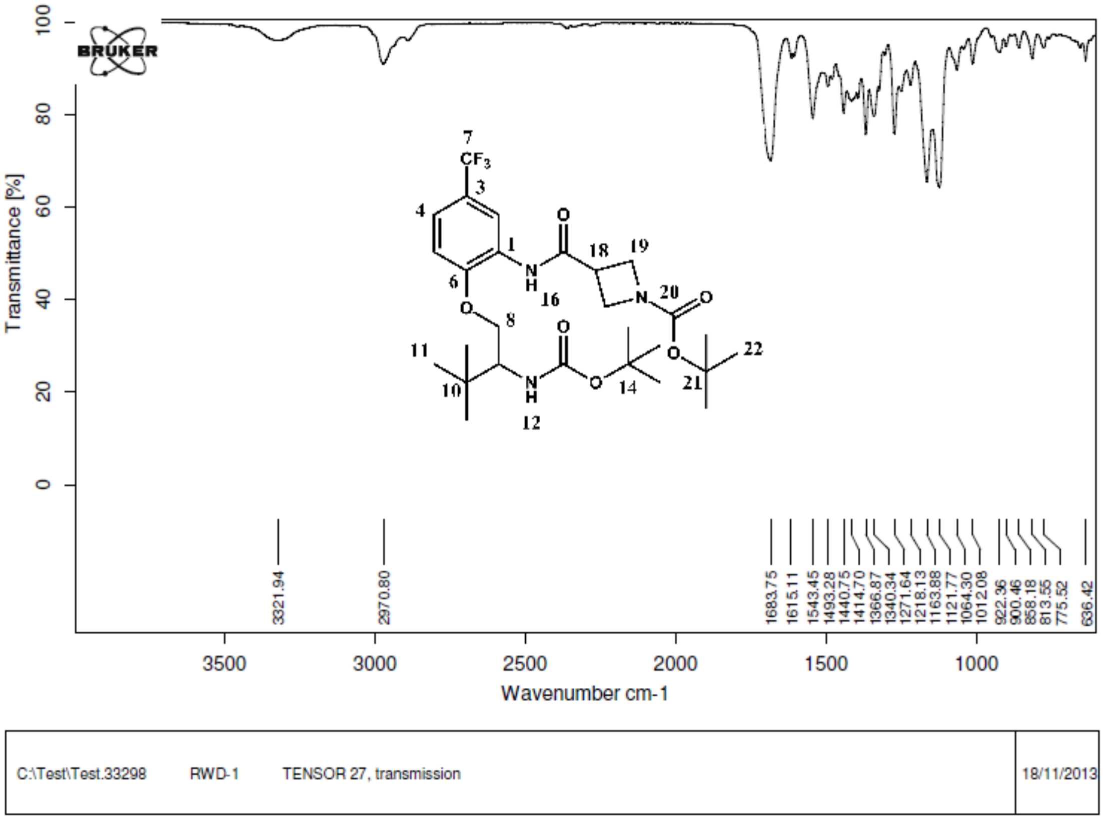

FT-IR of **3**.

Mass Spectrum SmartFormula Report

Analysis Info

Analysis Name	\\Utof\data\Nov 13\ESI43505_10_01_14159.d	Acquisition Date	14/11/2013 08:17:54
Method	2.5min_cal_sample_pos_naf_05-08-13.m	Operator	Mass Spec
Sample Name	ESI43505	Instrument / Ser#	micrOTOF 92
Comment			

Acquisition Parameter

Source Type	ESI	Ion Polarity	Positive	Set Nebulizer	2.0 Bar
Focus	Not active			Set Dry Heater	180 °C
Scan Begin	100 m/z	Set Capillary	4500 V	Set Dry Gas	10.0 l/min
Scan End	1000 m/z	Set End Plate Offset	-500 V	Set Divert Valve	Source

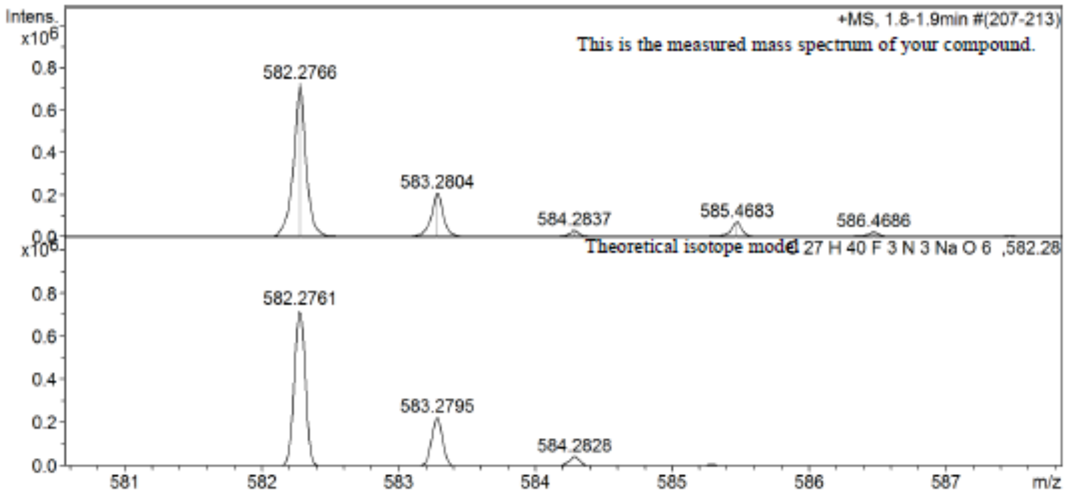

Meas. m/z	#	Formula	m/z	err [ppm]	Mean err [ppm]	rdb	e¯ Conf	mSigma
582.2766								
	1	C 27 H 40 F 3 N 3 Na O 6	582.2761	-0.8	-1.0	7.5	even	12.56

High-Resolution mass spectrum of **3**.

(rac)-tert-butyl (1-(2-(cyclopent-3-enecarboxamido)-4-(trifluoromethyl)phenoxy)-3,3-dimethylbutan-2-yl)carbamate [4]

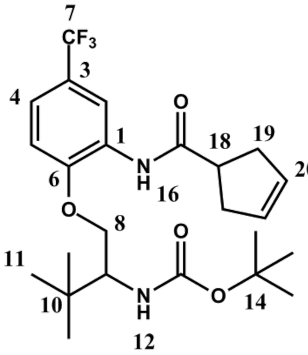

Prepared according to the representative procedure from racemic tert-butyl (1-(2-amino-4-(trifluoromethyl)phenoxy)-3,3-dimethylbutan-2-yl)carbamate on a 0.20 mmol scale. Chromatography (10:1 PET:EtOAc) to yield clear, pale, colourless needles (45 mg, 55%) for X-ray crystallographic and NMR studies.

δH (500 MHz, C_6D_6, 23˚C): 9.59 (1H, s, H2), 8.62 (1H, s, H16), 7.18 (1H, dd, J_1 8 Hz, J_2 2 Hz, H4), 6.17 (1H, d, J 8 Hz, H5), 5.63 (1H, m, H20), 5.55 (1H, m, H20), 4.31 (1H, d, J 10 Hz, H12), 3.82 (1H, t, J 9 Hz, H9), 3.57 (1H, dd, J_1 9 Hz, J_2 2 Hz, H8), 3.53 (1H, m, H18), 3.20 (1H, t, J 9 Hz, H8), 3.12 (1H, m, H19), 2.82 (1H, m, H19), 2.66 (1H, m, H19), 2.02 (1H, m, H19), 1.94 (1H, m, H19), 1.38 (9H, s, H15), 0.59 (9H, s, H11).

δC (125 MHz, C_6D_6, 23˚C): 174.29 (C17), 159.44 (C13), 149.21 (C6), 129.68 (C1), 129.15 (C20), 128.88 (C20), 125.02 (q, $^1J_{13C-19F}$ 271 Hz, C7), 123.62 (q, $^2J_{13C-19F}$ 32 Hz, C3), 119.65 (q, $^3J_{13C-19F}$ 4 Hz, C4), 116.86 (q, $^3J_{13C-19F}$ 4 Hz, C2), 109.92 (C5), 79.07 (C14), 69.44 (C8), 58.33 (C9), 44.56 (C18), 37.78 (C10), 36.66 (C19), 32.33 (C19), 28.05 (C15), 26.12 (C11).

δF (376 MHz, C_6D_6, 23˚C): -61.18 (F7).

HRMS: (ES-): 493.2283; Formula $C_{23}H_{33}F_3N_2O_4Na$, [M + Na] requires 493.2285.

ν_{max} (neat, cm^{-1}): 3329.77, 2966.89, 1682.57, 1615.16, 1538.69, 1490.02, 1439.21, 1367.02, 1339.35, 1270.04, 1214.67, 1162.41, 1120.86, 1063.79, 1012.42, 920.78, 812.86, 636.02.

MP: 107-108˚C

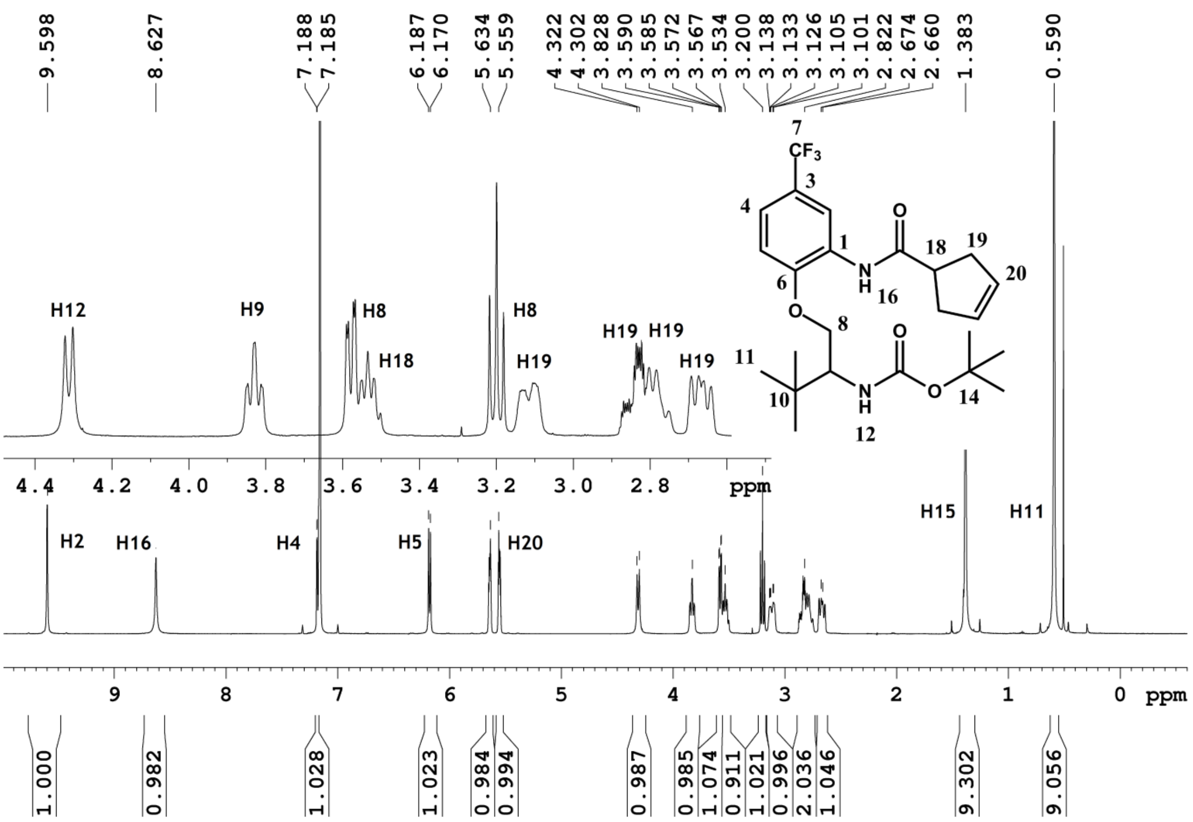

^{1}H NMR spectrum of **4** (23˚C, C_6D_6, 500 MHz).

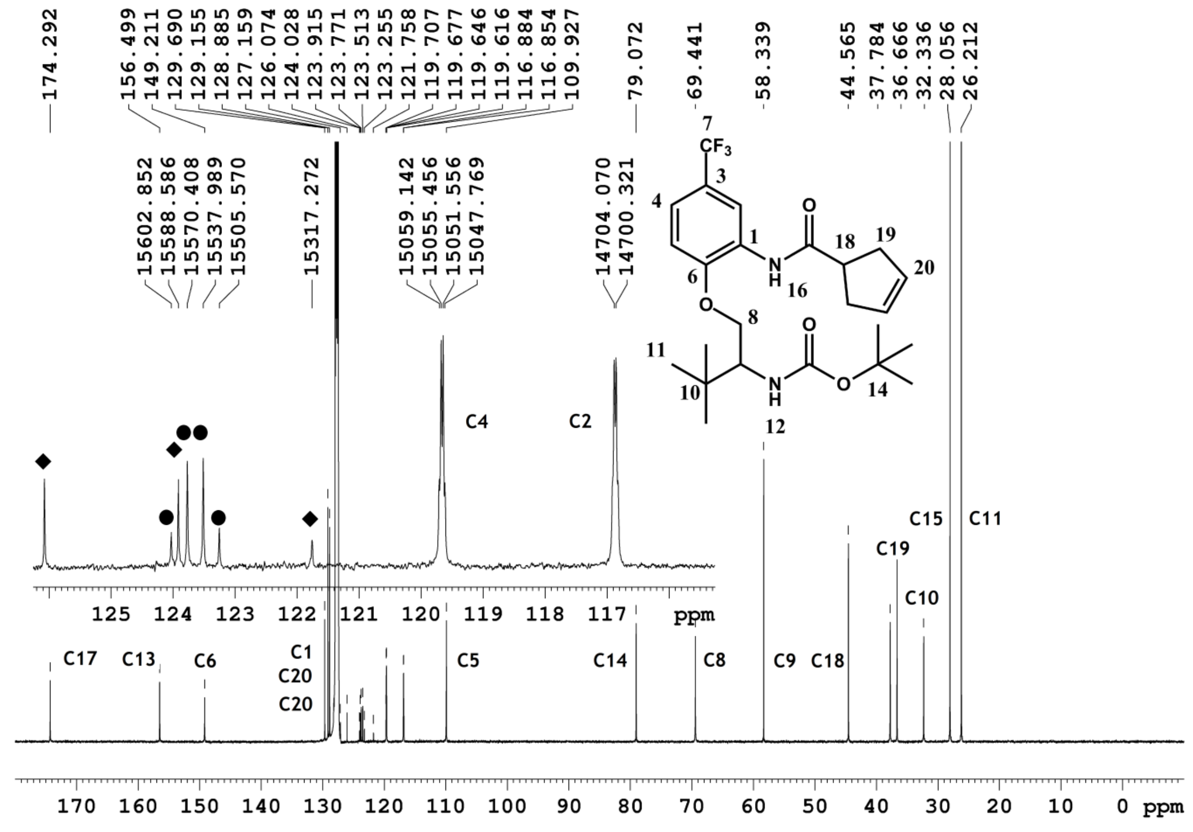

^{13}C NMR spectrum of **4** (23˚C, C_6D_6, 125MHz).

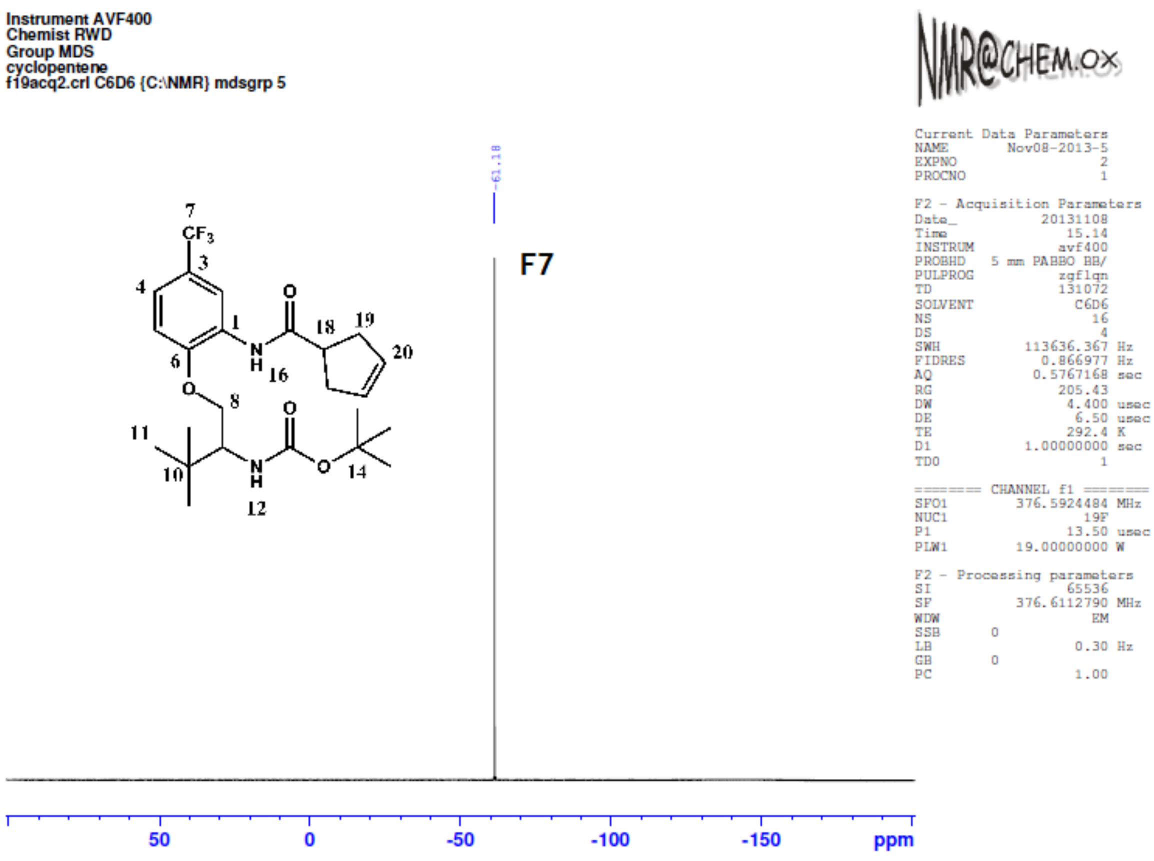

^{19}F NMR spectrum of **4** (23˚C, C_6D_6, 376 MHz).

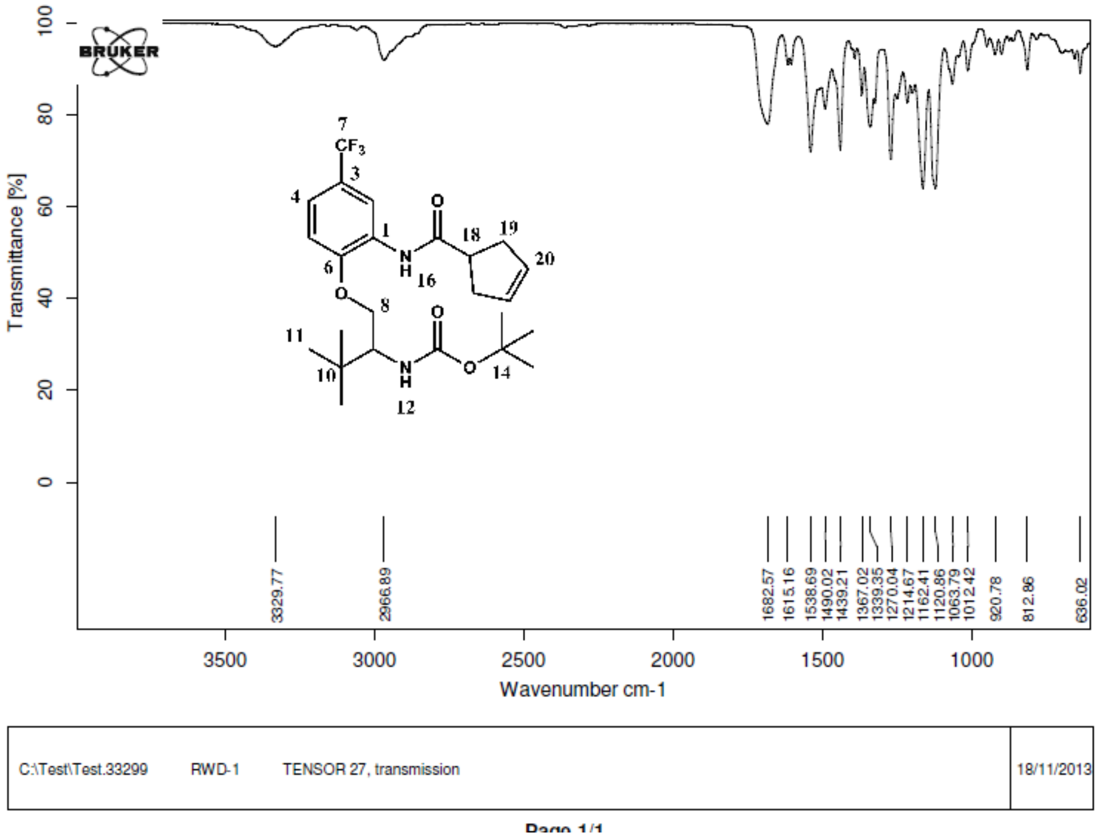

FT-IR of **4**.

Mass Spectrum SmartFormula Report

Analysis Info

Analysis Name	\\Utof\data\Nov 13\ESI43504_9_01_14158.d	Acquisition Date	14/11/2013 08:14:05
Method	2.5min_cal_sample_pos_naf_05-08-13.m	Operator	Mass Spec
Sample Name	ESI43504	Instrument / Ser#	micrOTOF 92
Comment			

Acquisition Parameter

Source Type	ESI	Ion Polarity	Positive	Set Nebulizer	2.0 Bar
Focus	Not active			Set Dry Heater	180 °C
Scan Begin	100 m/z	Set Capillary	4500 V	Set Dry Gas	10.0 l/min
Scan End	1000 m/z	Set End Plate Offset	-500 V	Set Divert Valve	Source

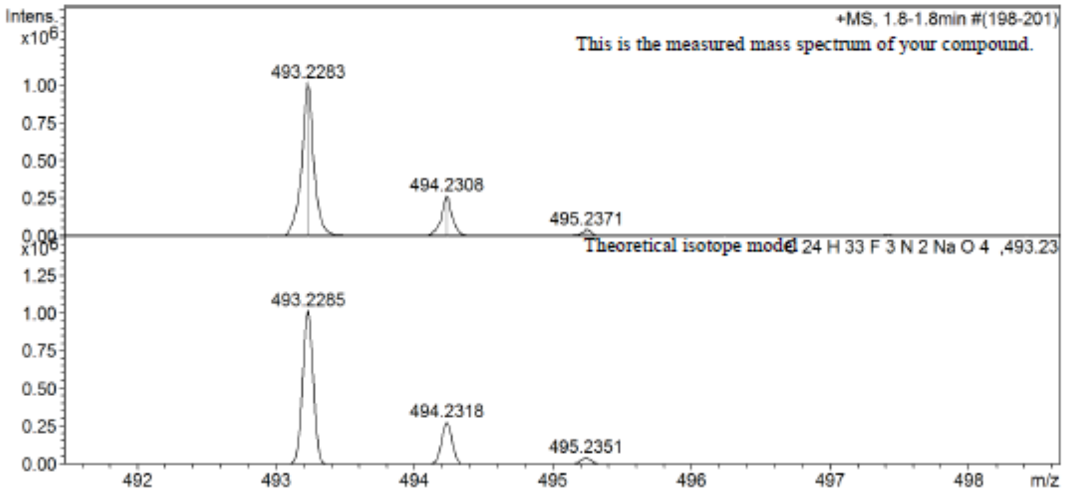

Meas. m/z	#	Formula	m/z	err [ppm]	Mean err [ppm]	rdb	e¯ Conf	mSigma
493.2283								
	1	C 24 H 33 F 3 N 2 Na O 4	493.2285	0.3	0.5	7.5	even	4.84

High resolution mass spectrum of **4**.

(rac)-tert-butyl (1-(2-(cyclopentanecarboxamido)-4-(trifluoromethyl)phenoxy)-3,3-dimethylbutan-2-yl)carbamate [5]

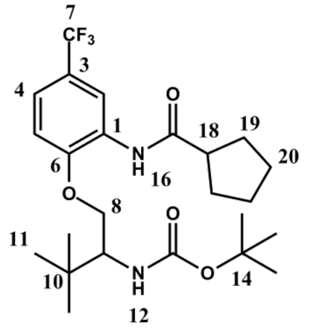

Prepared according to the representative procedure from racemic tert-butyl (1-(2-amino-4-(trifluoromethyl)phenoxy)-3,3-dimethylbutan-2-yl)carbamate on a 0.2 mmol scale. Chromatography (15:1 PET:EtOAc) and subsequent recrystallization in *n*-pentanes at 0 C to yield clear, pale, colourless blocks (26 mg, 27%) for X-ray crystallographic and NMR studies.

δH (500 MHz, C_6D_6, 23˚C): 9.59 (1H, s, H2), 8.67 (1H, s, H16), 7.17 (1H, dd, J_1 8 Hz, J_2 2 Hz, H4), 6.20 (1H, d, J 8 Hz, H5), 4.40 (1H, d, J 10 Hz, H12), 3.83 (1H, dt, J_1 10 Hz, J_2 2 Hz, H9), 3.61 (1H, dd, J_1 8 Hz, J_2 2 Hz, H8), 3.23 (1H, t, J 9 Hz, H8), 3.13 (1H, quintet, J 8 Hz, H18), 2.22 (1H, m, H19), 2.02 (1H, m, H19), 1.94 (1H, m, H19), 1.78 (2H, m, H20), 1.50 (2H, m, H20), 1.40 (9H, s, H15), 0.61 (9H, s, H11).

δC (125 MHz, C_6D_6, 23˚C): 175.72 (C17), 157.27 (C13), 149.98 (C6), 130.45 (C1), 125.74 (q, $^{1}J_{13C\text{-}19F}$ 271 Hz, C7), 124.22 (q, $^{2}J_{13C\text{-}19F}$ 32 Hz, C3), 120.27 (q, $^{3}J_{13C\text{-}19F}$ 4 Hz, C4), 117.47 (q, $^{3}J_{13C\text{-}19F}$ 4 Hz, C2), 110.57 (C5), 79.68 (C14), 70.22 (C8), 59.13 (C9), 47.37 (C18), 33.00 (C10), 32.09 (C19), 31.10 (C19), 28.78 (C15), 26.85 (C11), 26.85 (C20), 26.84 (C20).

δF (370 MHz, C_6D_6, 23˚C): -61.12 (F7).

HRMS: (ES-): 495.2443 ; Formula $C_{24}H_{35}F_3N_2O_4Na$, [M + Na] requires 495.2441.

ν_{max} (neat, cm^{-1}): 3331.84, 2963.73, 1680.30, 1603.01, 1537.56, 1489.14, 1438.98, 1366.84, 1340.59, 1269.23, 1214.33, 1163.34, 1119.66, 1063.96, 1012.34, 898.83, 812.21, 636.08.

MP: 122-123˚C.

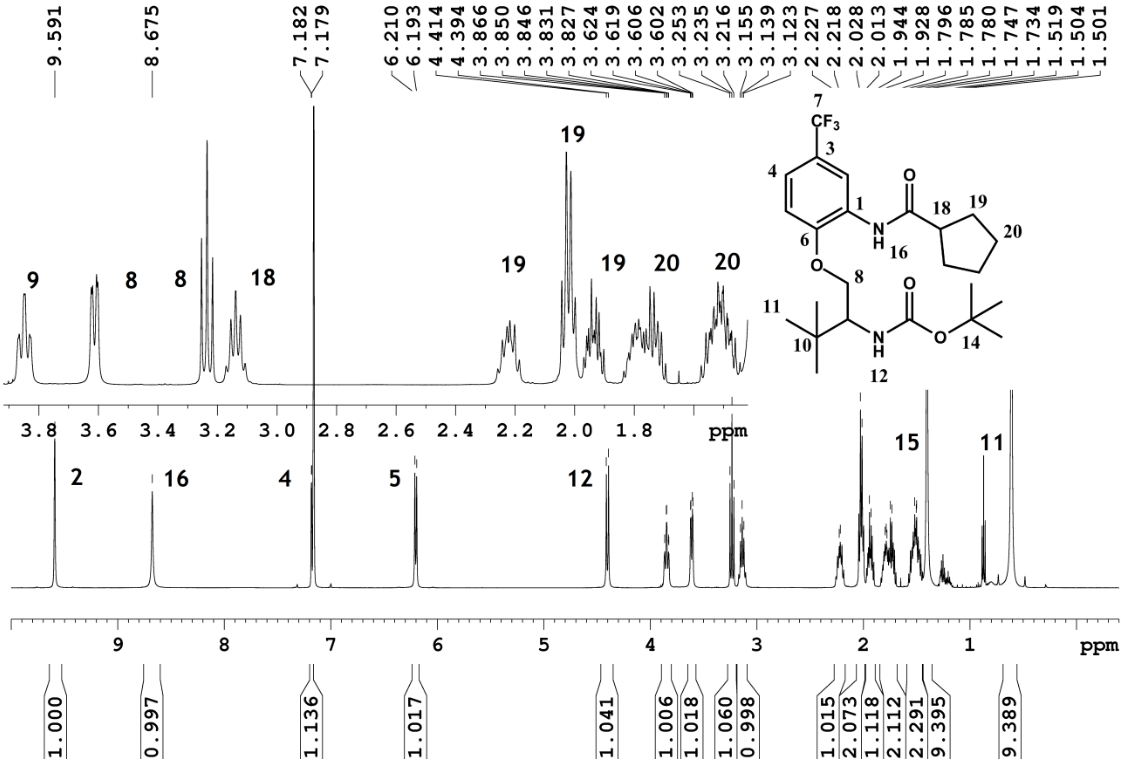

^{1}H NMR spectrum of **5** (23˚C, C_6D_6, 500 MHz).

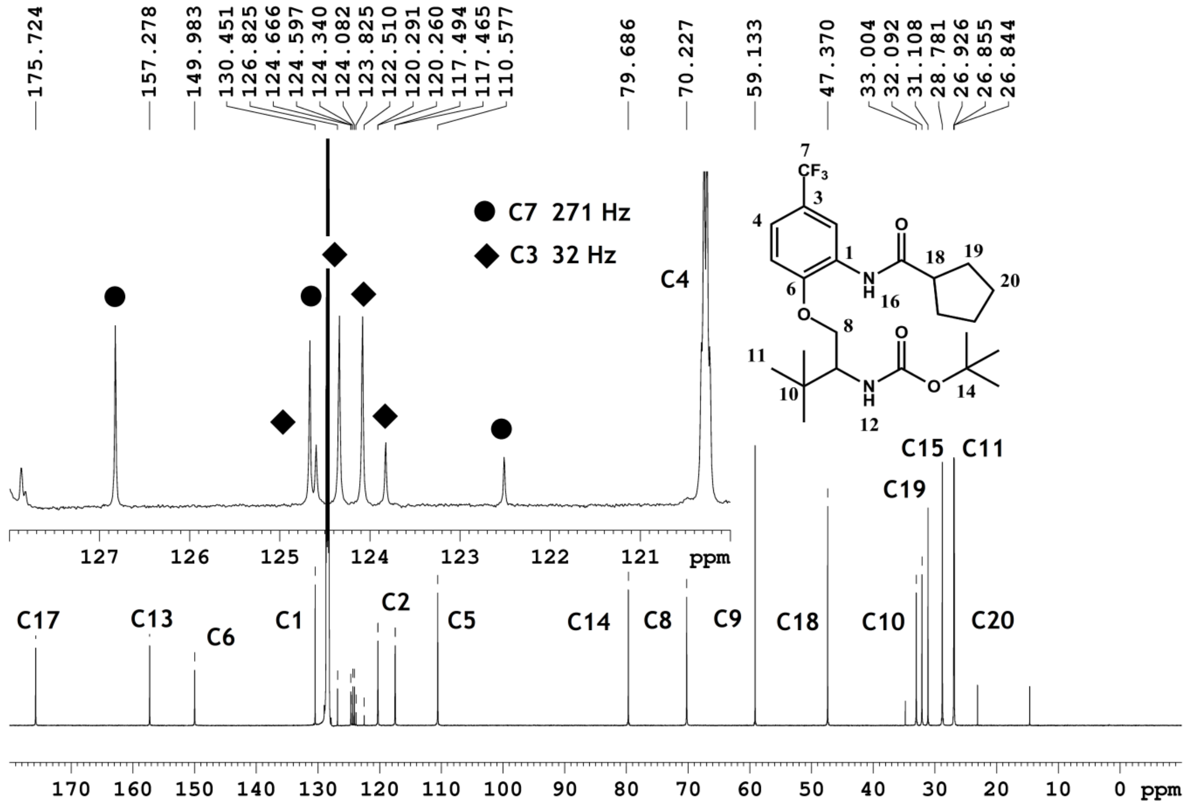

^{13}C NMR spectrum of **5** (23˚C, C_6D_6, 125 MHz).

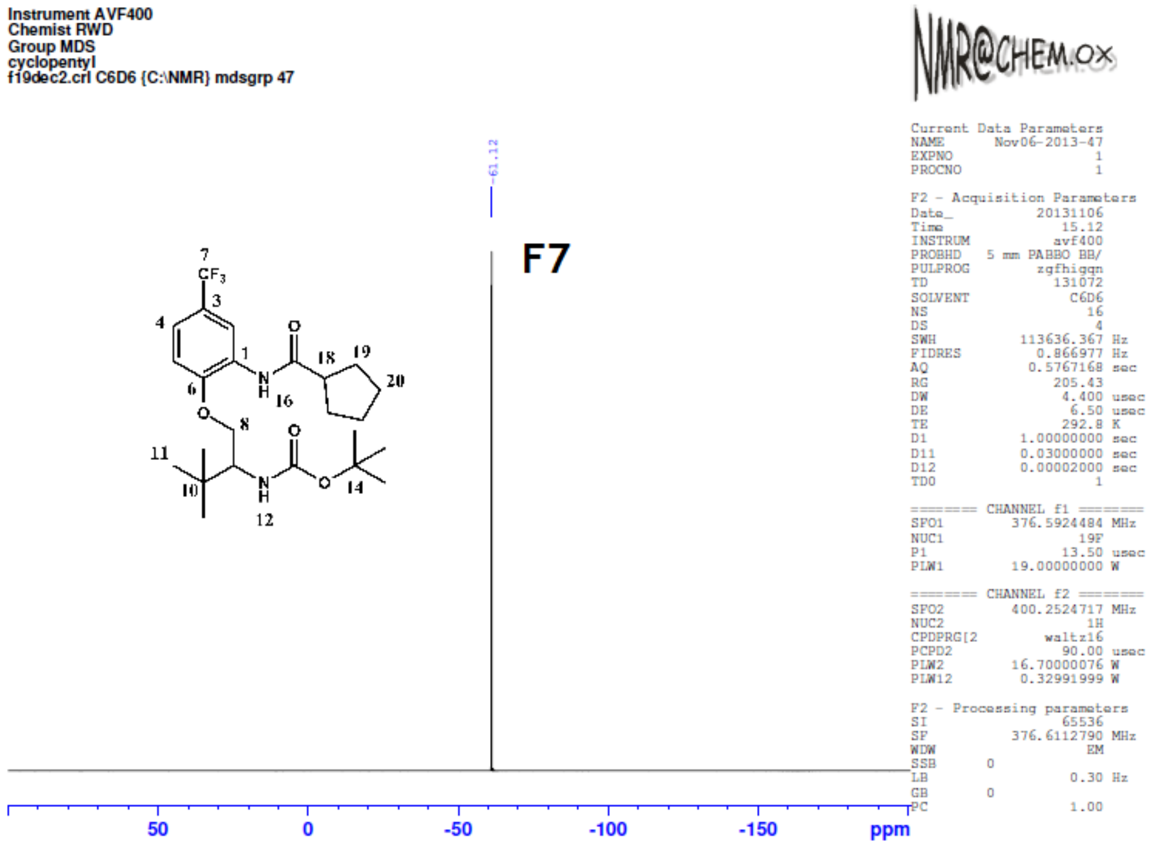

^{19}F NMR spectrum of **5** (23˚C in C_6D_6 (376 MHz).

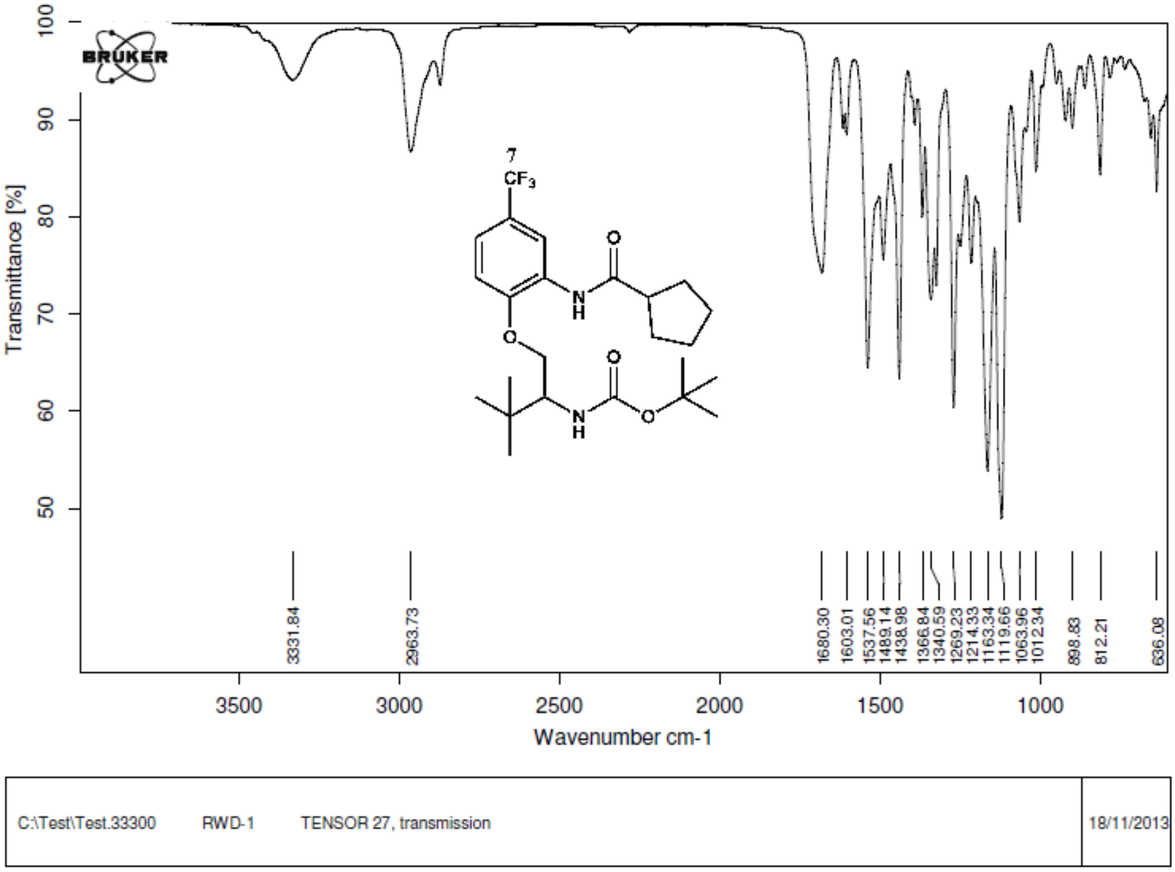

FT-IR of **5**.

Mass Spectrum SmartFormula Report

Analysis Info

Analysis Name	\\Utof\data\Nov 13\ESI43502_7_01_14156.d	Acquisition Date	14/11/2013 08:06:23
Method	2.5min_cal_sample_pos_naf_05-08-13.m	Operator	Mass Spec
Sample Name	ESI43502	Instrument / Ser#	micrOTOF 92
Comment			

Acquisition Parameter

Source Type	ESI	Ion Polarity	Positive	Set Nebulizer	2.0 Bar
Focus	Not active			Set Dry Heater	180 °C
Scan Begin	100 m/z	Set Capillary	4500 V	Set Dry Gas	10.0 l/min
Scan End	1000 m/z	Set End Plate Offset	-500 V	Set Divert Valve	Source

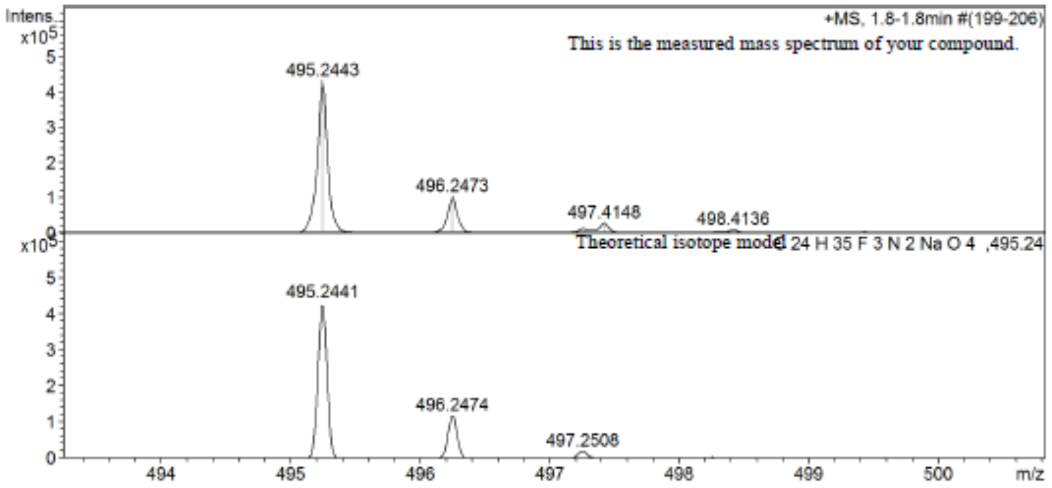

Meas. m/z	#	Formula	m/z	err [ppm]	Mean err [ppm]	rdb	e⁻ Conf	mSigma
495.2443								
	1	C 24 H 35 F 3 N 2 Na O 4	495.2441	-0.3	-1.0	6.5	even	18.52

High resolution mass spectrum of **5**.

***rac*-tert-butyl 4-((2-(2-((tert-butoxycarbonyl)amino)-3,3-dimethylbutoxy)-5-(trifluoromethyl)phenyl)carbamoyl)piperidine-1-carboxylate [6]**

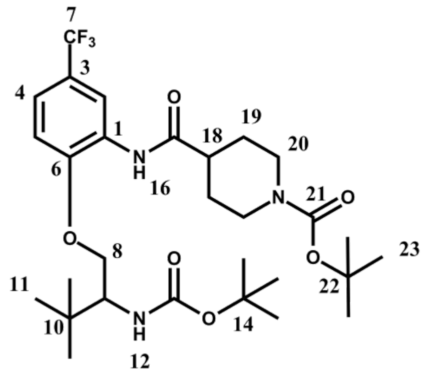

Prepared according to the representative procedure from racemic tert-butyl (1-(2-amino-4-(trifluoromethyl)phenoxy)-3,3-dimethylbutan-2-yl)carbamate on a 0.20 mmol scale. Chromatography (3:1 PET:EtOAc) to yield clear, pale, colourless needles (15 mg, 12%) for X-ray crystallographic and NMR studies.

δH (700 MHz, C_6D_6, 23˚C): 9.54 (1H, s, H2), 8.68 (1H, s, H16), 7.18 (1H, dd, J_1 8 Hz, J_2 2 Hz, H4), 6.17 (1H, d, *J* 7 Hz, H5), 4.50 (1H, bm, H20), 4.34 (1H, m, H12), 4.14 (1H, m, H20), 3.80 (1H, t, *J* 9 Hz, H9), 3.58 (1H, m, H8), 3.21 (1H, m, H18), 2.75 (3H, m, H20, H20, H18), 1.06 (1H, m, H19), 1.84 (3H, m, H19), 1.46 (9H, s, H23), 1.35 (9H, s, H15), 0.56 (9H, s, H11).

δC (125 MHz, C_6D_6, 23˚C): 173.82 (C17), 157.33 (C21), 155.04 (C13), 150.03 (C6), 130.20 (C1), 125.65 (q, $^1J_{13C\text{-}19F}$ 271 Hz, C7), 124.32 (q, $^2J_{13C\text{-}19F}$ 32 Hz, C3), 120.48 (q, $^3J_{13C\text{-}19F}$ 4 Hz, C4), 117.59 (q, $^3J_{13C\text{-}19F}$ 4 Hz, C2), 110.61 (C5), 79.74 (C22), 79.43 (C14), 70.44 (C8), 59.25 (C9), 44.63 (C18), 44.26 (bs, C20), 43.73 (bs, C20), 32.83 (C10), 29.92 (C19), 29.02 (C19), 28.92 (C14), 28.82 (C22), 26.86 (C11).

δF (376 MHz, C_6D_6, 23˚C): -61.20 (F7).

HRMS: (ES-): 610.3074; Formula $C_{29}H_{44}F_3N_3O_6Na$, [M + Na] requires 610.3083.

ν_{max} (neat, cm^{-1}): 2971.06, 1691.74, 1540.17, 1439.98, 1365.59, 1332.38, 1271.03, 1243.10, 1213.25, 1164.08, 1121.59, 1063.78, 1012.23, 954.85, 812.86, 636.35.

MP: 89°C.

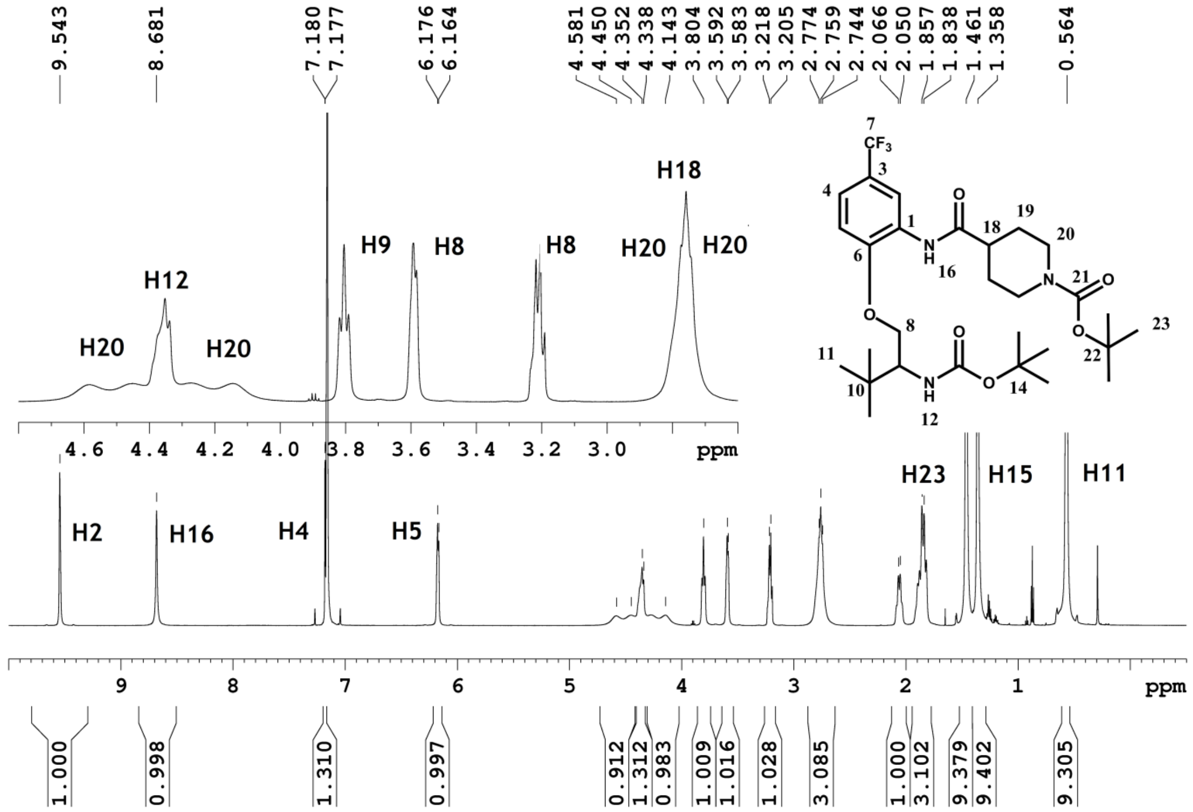

^{1}H NMR spectrum of **6** (23˚C, C_6D_6, 700 MHz).

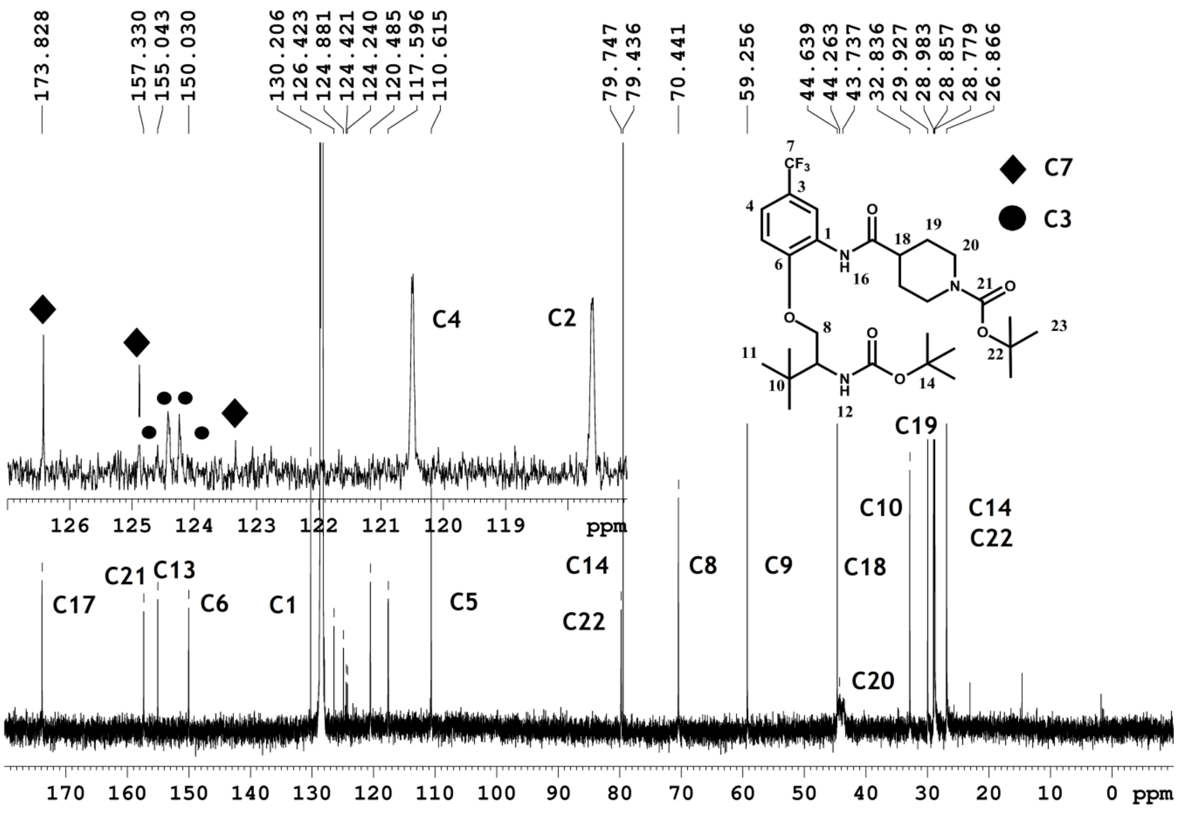

^{13}C NMR spectrum of **6** (23˚C, C_6D_6, 175 MHz).

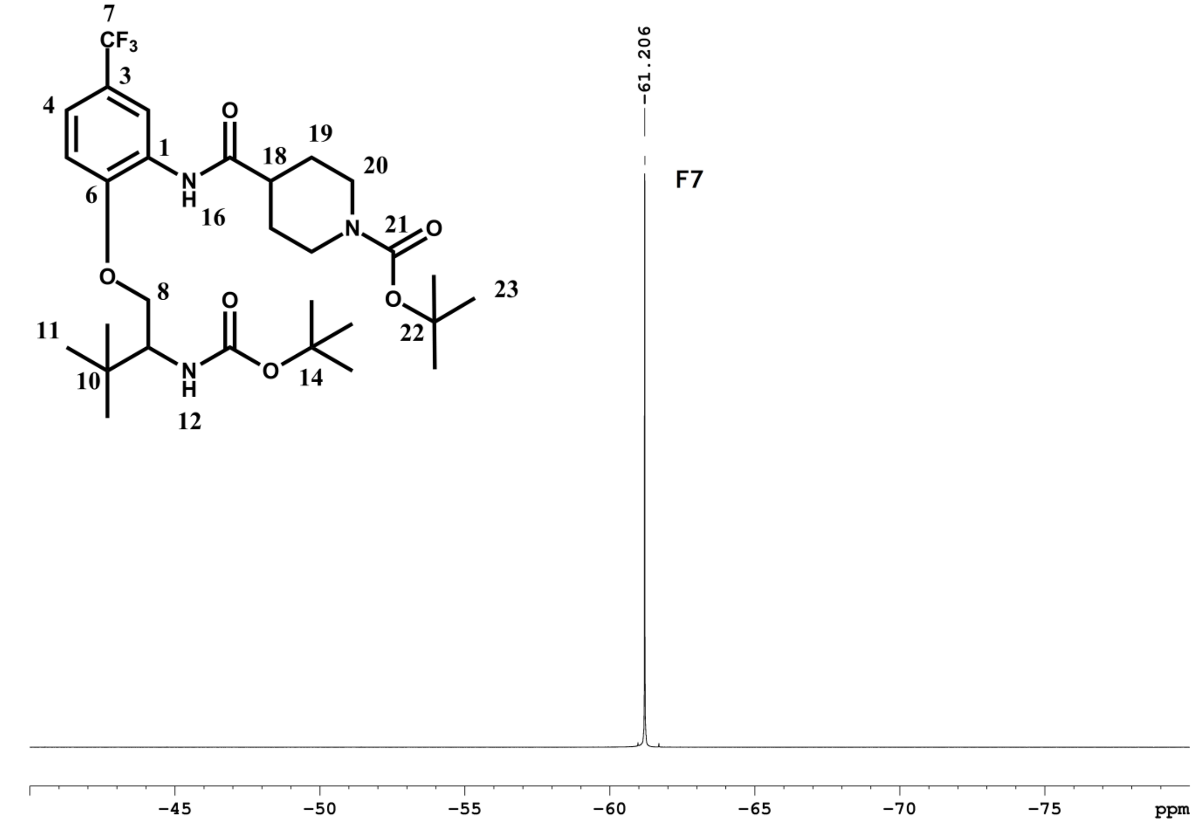

^{19}F NMR spectrum of **6** (23˚C, C_6D_6, 658 MHz).

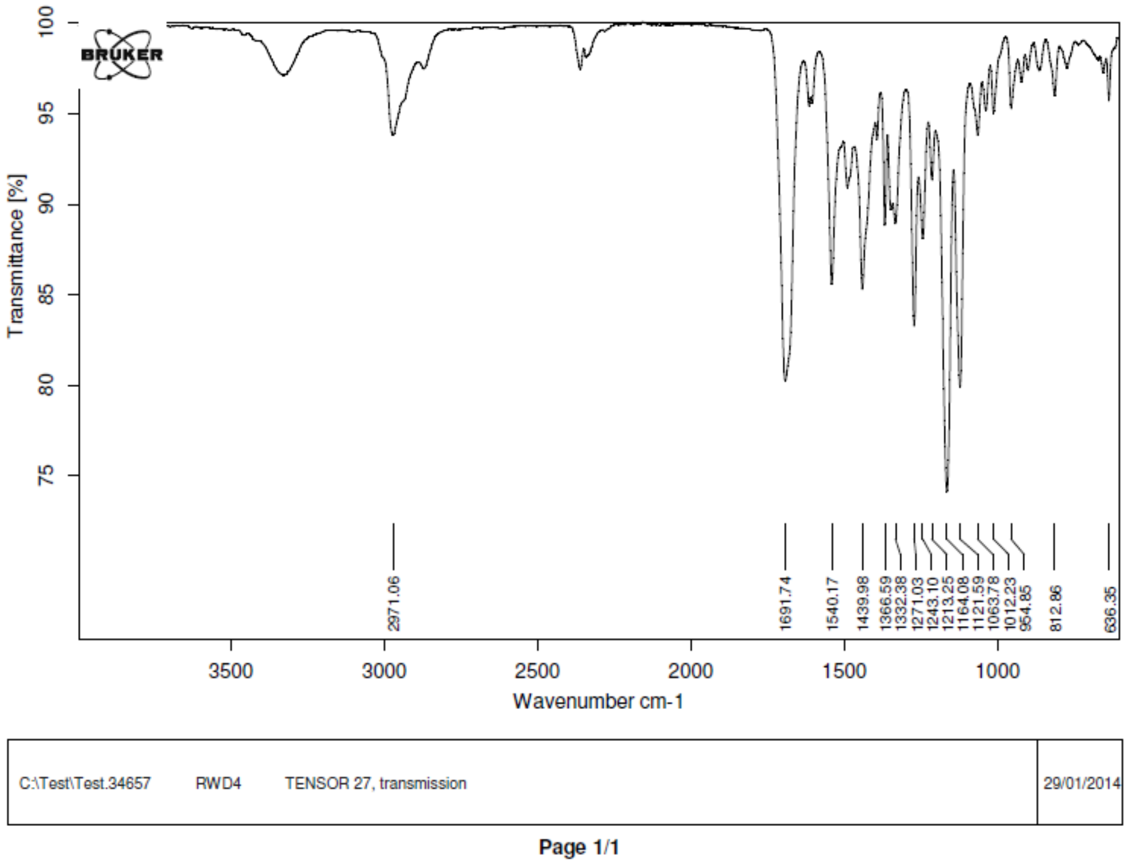

FT-IR of **6**.

Mass Spectrum SmartFormula Report

Analysis Info

Analysis Name	\\Utof\data\Jan 14\ESI44170_8_01_15470.d	Acquisition Date	14/01/2014 08:16:24
Method	2.5min_cal_sample_pos_naf_05-08-13.m	Operator	Mass Spec
Sample Name	ESI44170	Instrument / Ser#	micrOTOF 92
Comment			

Acquisition Parameter

Source Type	ESI	Ion Polarity	Positive	Set Nebulizer	2.0 Bar
Focus	Not active			Set Dry Heater	180 °C
Scan Begin	100 m/z	Set Capillary	4500 V	Set Dry Gas	10.0 l/min
Scan End	1000 m/z	Set End Plate Offset	-500 V	Set Divert Valve	Source

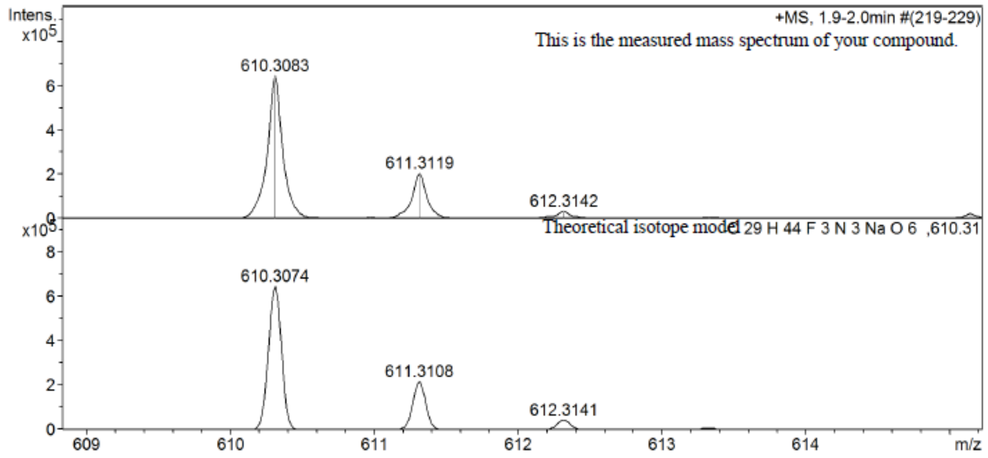

Meas. m/z	#	Formula	m/z	err [ppm]	Mean err [ppm]	rdb	e⁻ Conf	mSigma
610.3083								
	1	C 29 H 44 F 3 N 3 Na O 6	610.3074	-1.3	-1.5	7.5	even	9.04

High resolution mass spectrum of **6**.

(S)-tert-butyl (1-(2-(2,2-diphenylacetamido)-4-(trifluoromethyl)phenoxy)-3,3-dimethylbutan-2-yl)carbamate [7]

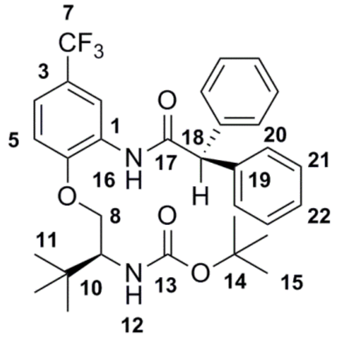

Prepared according to the representative procedure from (S)-tert-butyl (1-(2-amino-4-(trifluoromethyl)phenoxy)-3,3-dimethylbutan-2-yl)carbamate on 0.2 mmol scale. Chromatography (silica gel, petroleum ether : ethyl acetate 8:1), 2 separations, followed by recrystallization in EtOAc/n-pentane (1:10), 22 mg, 20%.

δH (500 MHz, $CDCl_3$, 23˚C): 8.87 (1H, d, *J* 2 Hz, H2), 8.86 (1H, s, H16), 7.46 (2H, d, *J* 8 Hz, H21), 7.40 (2H, d, *J* 7 Hz, H21), 7.35-7.30 (4H, M, H20), 7.27-7.23 (3H, m, H4, H22), 6.81 (1H, d, *J* 8 Hz, H5), 5.31 (1H, s, H18), 4.61 (1H, d, *J* 9 Hz, H12), 4.09 (1H, m, H8), 4.94 (1H, t, *J* 8 Hz, H8), 3.80 (1H, m, H9), 1.37 (9H, s, H15), 0.96 (9H, s, H11).

δC (125 MHz, $CDCl_3$, 23˚C): 171.04 (C17), 156.76 (C13), 149.82 (C6), 139.75 (C19), 139.58 (C19), 129.22 (C21), 129.05 (C21), 128.86 (C20), 128.65 (C20), 127.44 (C22), 127.20 (C22), 124.38 (C7, q, $^1J_{13C\text{-}19F}$ = 271 Hz), 123.62 (C3, q, $^2J_{13C\text{-}19F}$ = 33 Hz), 120.87 (C4, d, $^3J_{C\text{-}F}$ = 4 Hz), 117.14 (C2, d, $^3J_{C\text{-}F}$ = 4 Hz), 110.57 (C5), 79.79 (C14), 70.38 (C8), 59.24 (C18), 58.55 (C9), 33.21 (C10), 28.50 (C15), 27.06 (C11). Resonance for C1 is obscured.

δF (235 MHz, $CDCl_3$, 23˚C): -61.23 (F7).

HRMS: (ES+): found (593.2609); $C_{32}H_{37}F_3N_2O_4Na$, [M + H+] requires 593.2598.

ν_{max} (neat, cm^{-1}): 3367.18, 3063.99, 3030.05, 2968.15, 2361.02, 2250.54, 1683.90, 1614.03, 1602.62, 1538.41, 1493.01, 1439.33, 1391.58, 1366.71, 1341.05, 1270.16, 1248.71, 1214.22, 1160.01, 1119.42, 1064.61, 1042.51, 1033.27, 1010.67.

MP: 136-138˚C.

$[\alpha]_D^{25.0}$ +47.30° (c = 0.15, $CHCl_3$).

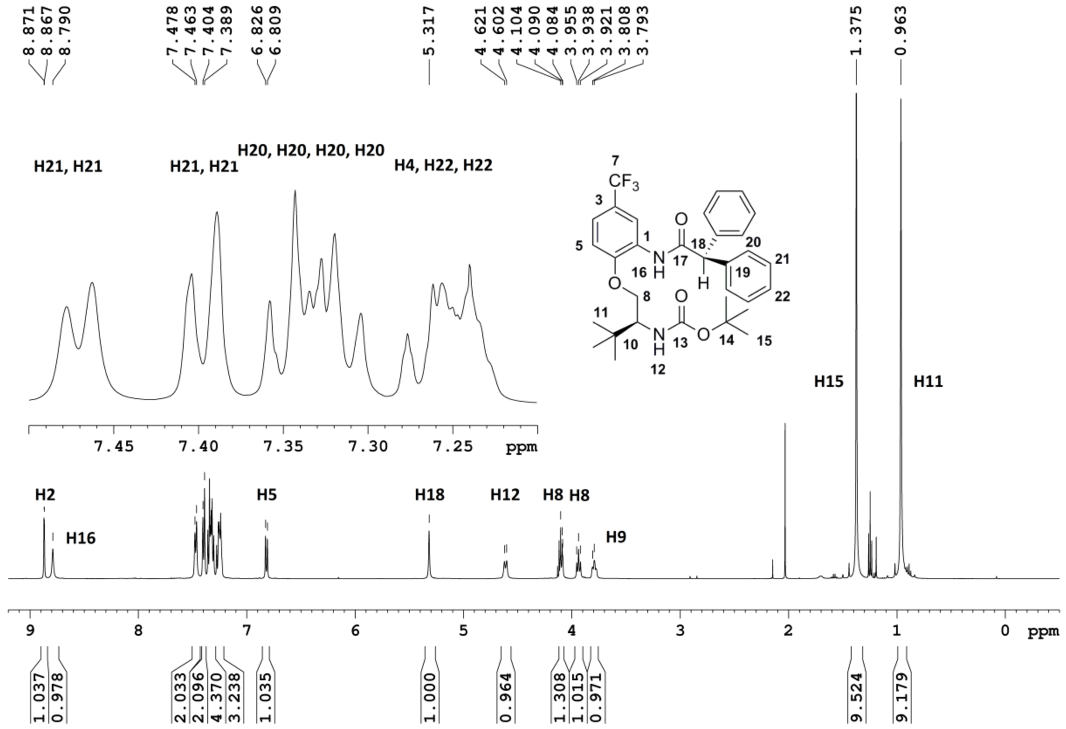

^{1}H NMR spectrum of **7** (23˚C, $CDCl_3$, 500 MHz).

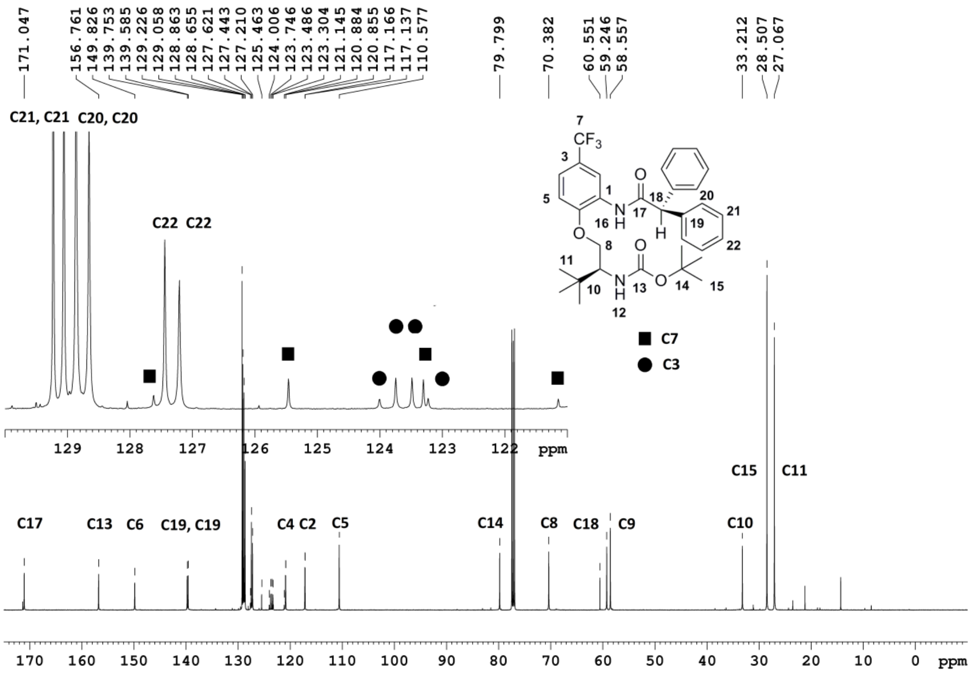

^{13}C NMR spectrum of **7** ($CDCl_3$, 23˚C, 125MHz).

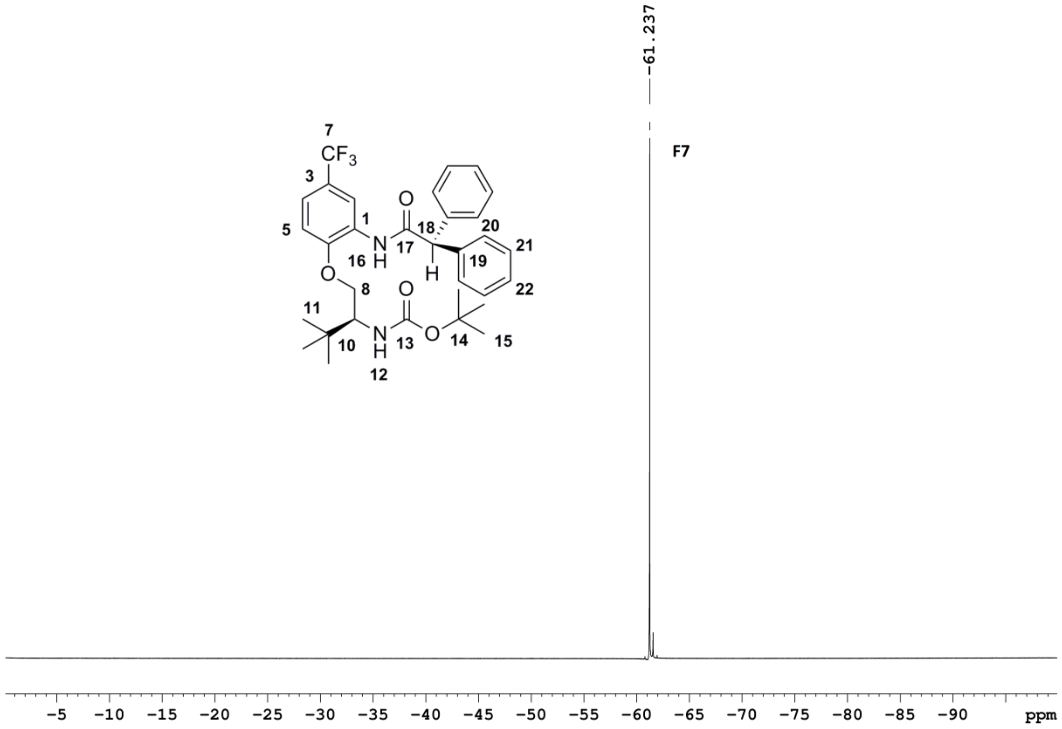

^{19}F NMR spectrum of **7** ($CDCl_3$, 23˚C, 282 MHz).

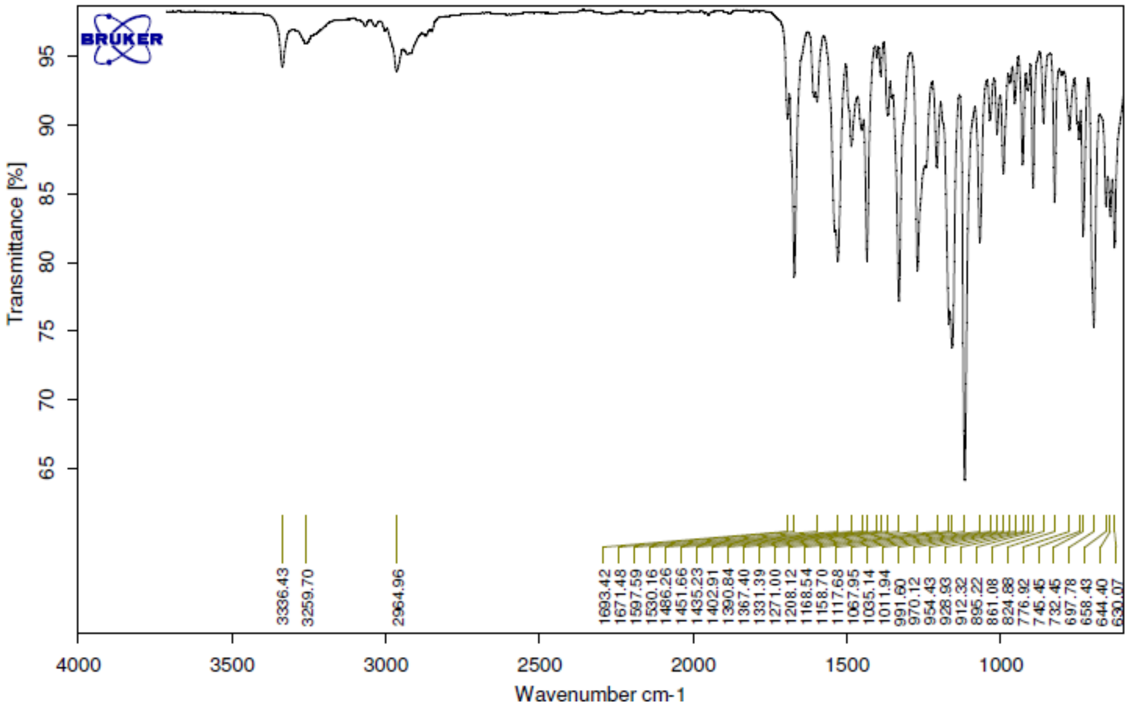

Solid State FT-IR of diffraction quality crystals of **7**.

Mass Spectrum SmartFormula Report

Analysis Info

Analysis Name	\\Utof\Data\Sep 11\ESI30234_25_01_32529.d	Acquisition Date	19/09/2011 08:25:29
Method	2.5min_cal_sample_pos_Naf_11-10-10.m	Operator	Mass Spec
Sample Name	ESI30234	Instrument / Ser#	micrOTOF 92
Comment			

Acquisition Parameter

Source Type	ESI	Ion Polarity	Positive	Set Nebulizer	2.0 Bar
Focus	Not active			Set Dry Heater	180 °C
Scan Begin	100 m/z	Set Capillary	4500 V	Set Dry Gas	10.0 l/min
Scan End	1500 m/z	Set End Plate Offset	-500 V	Set Divert Valve	Source

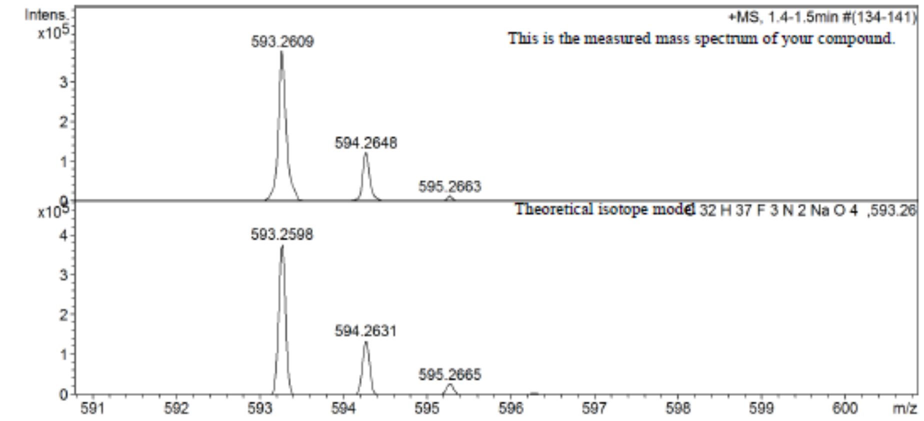

Meas. m/z	#	Formula	m/z	err [ppm]	Mean err [ppm]	rdb	e⁻ Conf	mSigma
593.2609	1	C 32 H 37 F 3 N 2 Na O 4	593.2598	-1.8	-2.0	13.5	even	22.64

High resolution mass spectrum of **7**.

(S)-2-((2-(2,2-diphenylacetamido)-4-(trifluoromethyl)phenyl)amino)-3,3-dimethylbutyl acetate

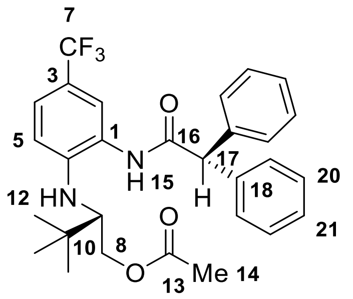

Prepared according to the general procedure at (0.37 mmols): EDCI.HCl (300 mg, 1.5 mmol), diphenylbenzoic acid (614 mg, 3.0 mmol), N,N-dimethylaminopyridine (7 mg, 0.05 mmol), pyridine (1 mL) and DCM (5 mL). Chromatography (silica gel, petroleum ether : ethyl acetate 5:1), 2 separations, followed by recrystallization in EtOAc/*n*-pentane (1:10), 20 mg, 12% for X-ray crystallographic studies.

δH (500 MHz, $CDCl_3$, 23 C): 7.37 (1H, m, H2), 7.37-7.33 (4H, m, H19), 7.33-7.31 (4H, m, H20), 7.30 (1H, m, H4), 7.26 (2H, m, H21), 6.75 (1H, d, *J* 7 Hz, H5), 5.12 (1H, s, H17), 4.14 (1H, dd, J_1 11 Hz, J_2 4 Hz, H8), 4.03 (1H, dd, J_1 11 Hz, J_2 8 Hz, H8), 3.89 (1H, d, *J* 9 Hz, H12), 3.41 (1H, m, C9), 1.78 (3H, s, H14), 0.83 (9H, s, 11).

δC (125 MHz, $CDCl_3$, 23 C): 171.80 (C13), 171.63 (C16), 146.21 (C6), 139.09 (C18), 139.06 (C18), 129.19 (C20), 129.17 (C20), 128.98 (C19), 128.96 (C19), 127.78 (C21), 125.13 (q, $^3J_{13C-19F}$ 4 Hz, C4), 124.62 (q, $^1J_{13C-19F}$ 271 Hz, C7), 124.31 (q, $^3J_{13C-19F}$ 3 Hz, C2), 121.87 (C1), 118.50 (q, $^2J_{13C-19F}$ 33 Hz, C3), 111.74 (C5), 64.81 (C8), 60.56 (C9), 59.42 (C17), 34.61 (C10), 26.80 (C11), 20.89 (C14).

δF (236 MHz, C_6D_6, 23 C): -60.69 (s, F7).

HRMS: found (512.2288); $C_{29}H_{31}F_3N_2O_3$, requires 512.2287.

ν_{max} (neat, cm^{-1}): 3421.87, 3234.37, 2964.67, 1737.33, 1659.72, 1617.85, 1535.59, 1494.39, 1328.92, 1235.71, 1152.79, 1109.90, 1074.88, 1042.74, 908.78, 819.91, 730.71, 698.14, 637.91.

MP: 70-72 C.

$[\alpha]_D^{25.0}$ +8.2 (c = 0.01, $CHCl_3$).

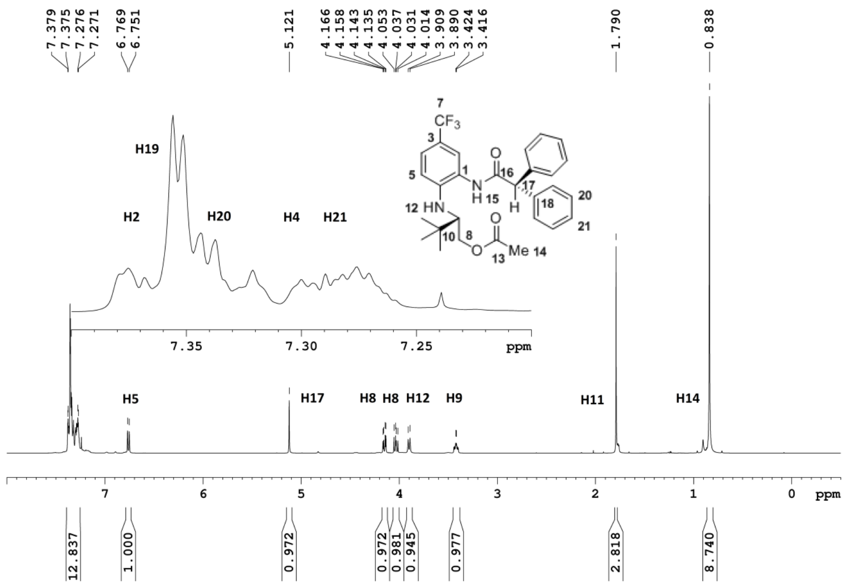

^{1}H NMR spectra in $CDCl_3$ at 23C on a 500 MHz instrument.

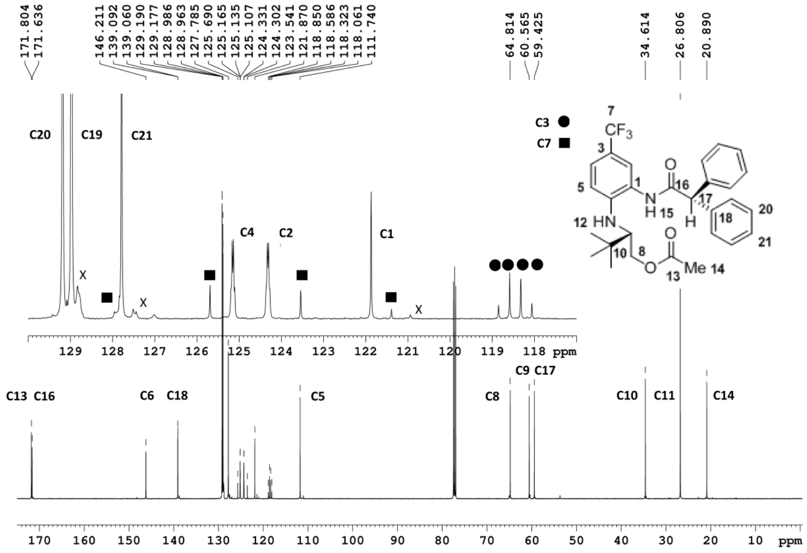

^{13}C NMR spectrum ($CDCl_3$, 23C, 125 MHz).

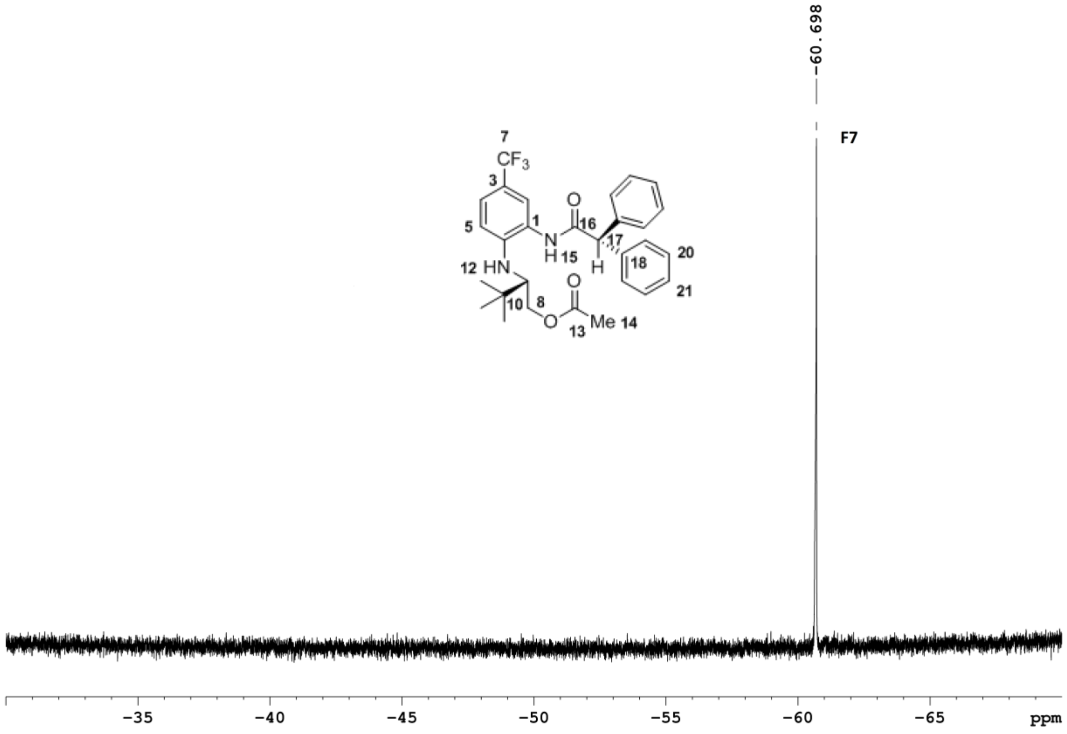

^{19}F NMR spectrum ($CDCl_3$, 23C, 236 MHz).

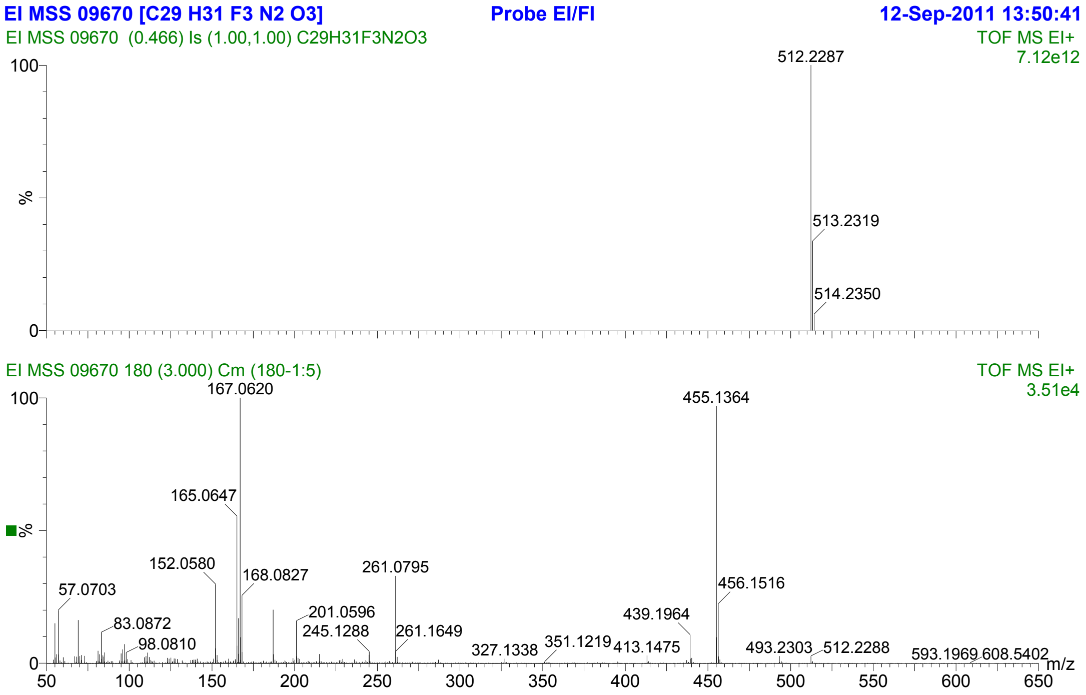

High Resolution mass spectrum.

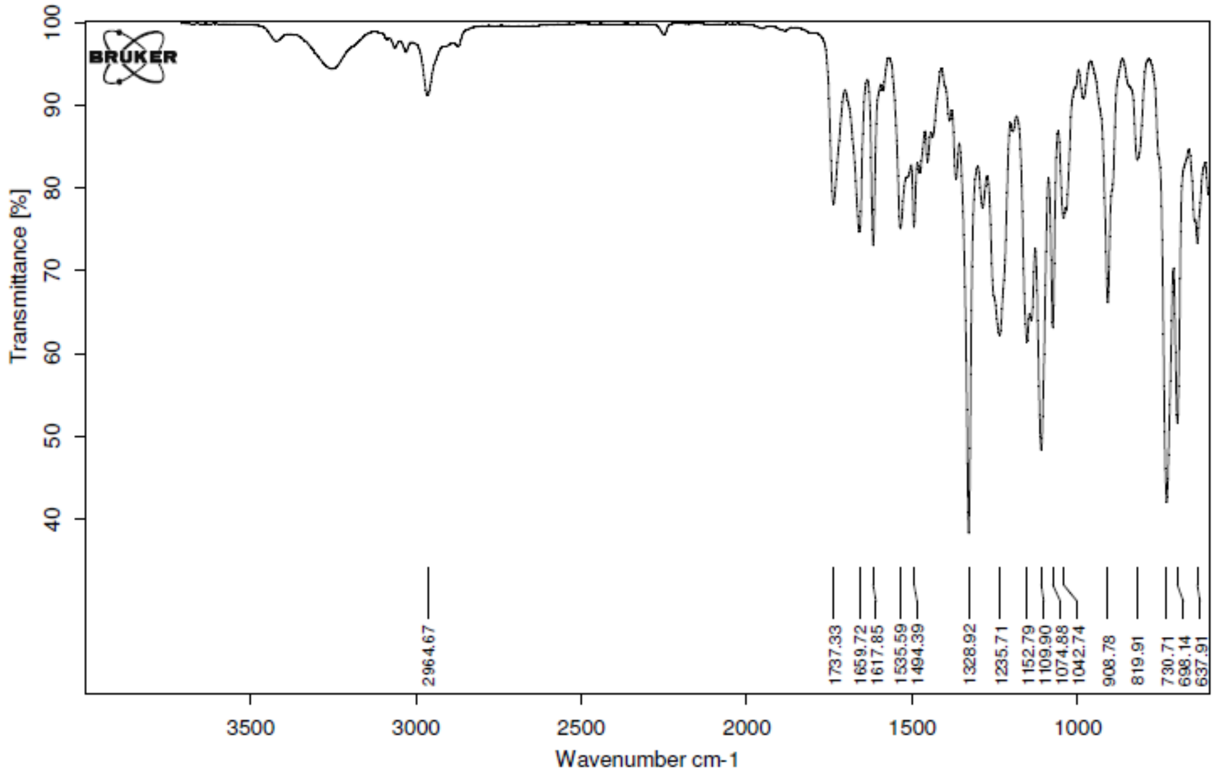

Solution State FT-IR spectrum.

(S)-tert-butyl (3,3-dimethyl-1-(2-(3,3,3-trifluoro-2-(trifluoromethyl)propanamido)-4-(trifluoromethyl)phenoxy)butan-2-yl)carbamate [8]

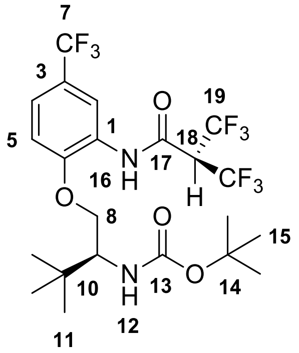

Prepared according to the representative procedure: (S)-tert-butyl (1-(2-amino-4-(trifluoromethyl)phenoxy)-3,3-dimethylbutan-2-yl)carbamate (130 mg, 0.34 mmol), EDCI.HCl (132 mg, 0.68 mmol), 2H perfluoro-2-methylpropanoic acid (133 mg, 0.68 mmol), N,N-dimethylaminopyridine (7 mg, 0.05 mmol), pyridine (1.1 mL) and DCM (20 mL). Chromatography (silica gel, petroleum ether : ethyl acetate 5:1), 165 mg, 86%. Diffraction quality crystals, colorless blocks, obtained from pure *n*-pentanes by cooling overnight.

δH (500 MHz, C_6D_6, 23˚C): 9.84 (1H, s, H16), 9.25 (1H, d, *J* 1.0 Hz, H2), 7.13 (1H, dd, *J*1 8 Hz, *J*2 1 Hz, H4), 6.13 (1H, d, *J* 8 Hz, H5), 5.20 (1H, m, $^3J_{1H-19F}$ 7.5 Hz, H18), 4.42 (1H, d, *J* 10 Hz, H12), 3.57 (1H, dt, J_1 10 Hz, J_2 2 Hz H8), 3.57 (1H, dd, J_1 6 Hz, J_2 2 Hz H9), 3.35 (1H, adventitious t, *J* 10 Hz, H8), 1.35 (9H, s, H15), 0.48 (9H, s, H11).

δC (125 MHz, C_6D_6, 23˚C): 158.36 (C13), 157.53 (m, C17, $^3J_{13C-19F}$ = 1 Hz), 150.73 (C6, q, $^3J_{13C-19F}$ = 1.5 Hz), 129.30 (C1), 125.29 (q, $^1J_{13C-19}$ 272 Hz, C7), 124.62 (q, $^2J_{13C-19F}$ 33 Hz, C3), 122.82 (two overlapping qq, $^1J_{13C-19F}$ 272 Hz, J_3 1.8 Hz, Δδ C19, C19′ = 0.09 ppm), 122.27 (q, $^3J_{13C-19F}$ 4 Hz, C4), 117.76 (q, $^3J_{13C-19F}$ 4 Hz, C2), 112.12 (C5), 80.61 (C14), 71.70 (C8), 60.07 (C9), 55.66 (hept, $^2J_{13C-19F}$ 29 Hz, C18), 32.30 (C10), 28.61 (C15), 26.69 (C11).

δF (470 MHz, C_6D_6, 23˚C): -61.34 (s, C7), -63.38 (q, $^3J_{1H-19F}$ 9.0 Hz, C19), -64.14 (q, $^3J_{1H-19F}$ 9.0 Hz, C19).

δH (500 MHz, $CDCl_3$, 23˚C): 9.60 (1H, s, H16), 8.73 (1H, s, H2), 7.32 (1H, d, *J* 9 Hz, H4), 6.85 (1H, d, *J* 9 Hz, H5), 4.74 (1H, d, H12), 4.72 (1H, hept, *J* 7 Hz, H18), 4.15 (1H, d, *J* 7 Hz, H8), 3.99 (1H, t, *J* 9 Hz, H9), 3.73 (1H, t, *J* 10 Hz, H8), 1.40 (9H, s, H15), 1.01 (9H, s, H11).

δH (500 MHz, CF_3CD_2OD, 23˚C): 9.33 (1H, s, H16), 8.30 (1H, s, H2), 7.28 (1H, d, *J* 8 Hz, H4), 6.85 (1H, d, *J* 9 Hz), 5.21 (1H, d, *J* 10 Hz, H12), 4.58 (1H, hept, *J* 7 Hz, H18), 4.10 (d, *J* 9 Hz, H8), 3.87 (1H, t, *J* 7 Hz, H9), 3.67 (1H, m, H8), 1.25 (9H, s, H15), 0.84 (9H, s, H11).

HRMS: (ES+): found (577.1719); $C_{26}H_{27}F_9N_2O_4$, [M + H+] requires 577.1725.

ν_{max} (neat, cm^{-1}): 3458.29, 3381.78, 2968.36, 1716.03, 1682.77, 1671.51, 1555.63, 1508.44, 1443.88, 1368.68, 1354.43, 1340.39, 1320.44, 1289.82, 1273.39, 1234.49, 1212.58, 1165.93, 1119.73, 1098.20, 1068.68, 1042.31, 1010.93, 949.72, 930.58, 918.98, 900.40, 867.34, 838.62,

817.17, 780.88, 759.14, 720.08, 657.88, 634.55, 624.70.

MP: 114-116˚C.

$[\alpha]_D^{25.0}$ +62.0 (c = 0.002, $CHCl_3$).

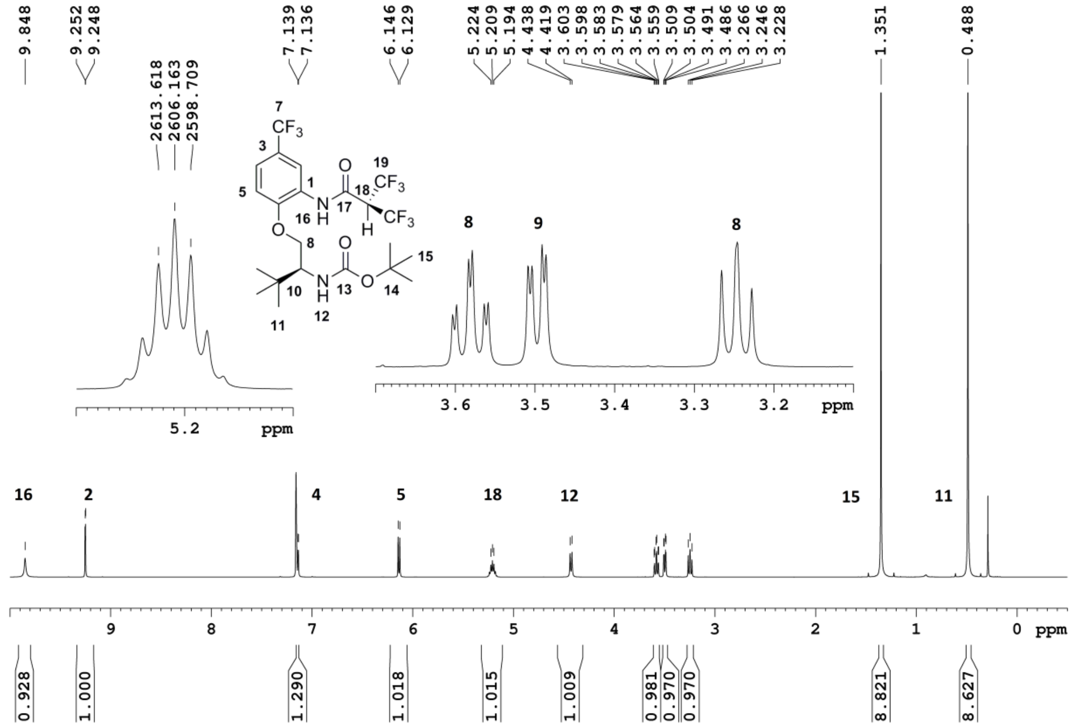

^{1}H NMR spectrum of **8** (23˚C, C_6D_6, 500 MHz).

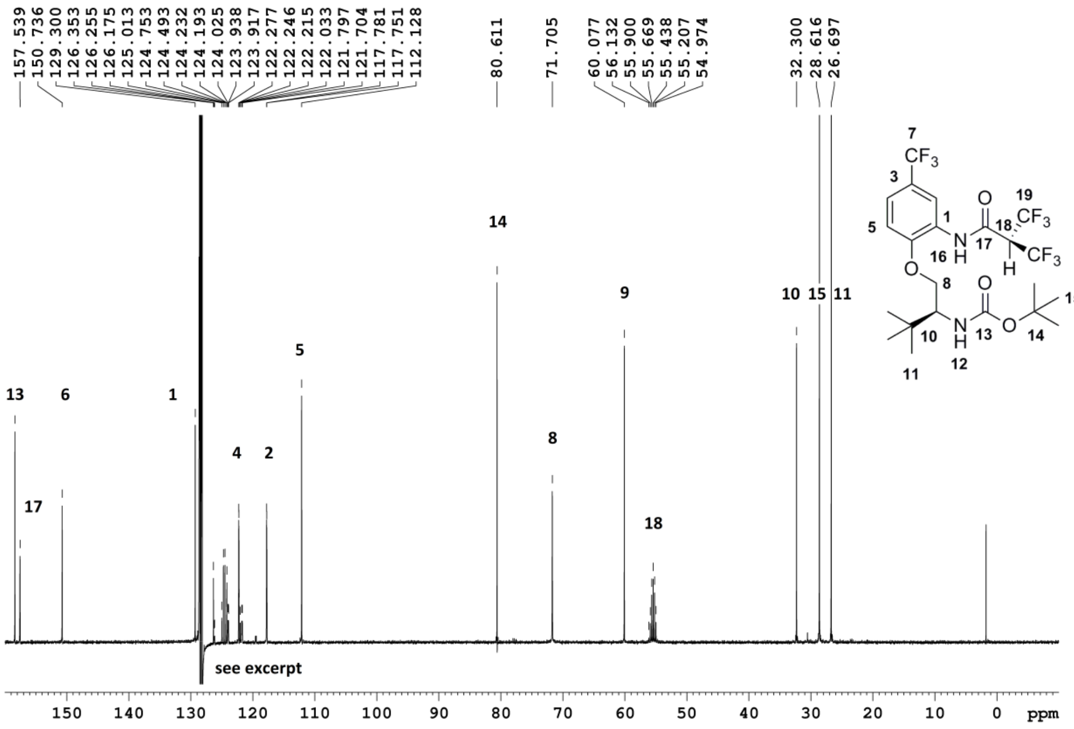

^{13}C NMR spectrum of **8** (C_6D_6, 23˚C, 125 MHz).

Mass Spectrum SmartFormula Report

Analysis Info

Analysis Name	\\Utof\Data\Sep 11\ESI29946_9_01_31872.d	Acquisition Date	01/09/2011 10:53:53
Method	2.5min_cal_sample_pos_Naf_11-10-10.m	Operator	Mass Spec
Sample Name	ESI29946	Instrument / Ser#	micrOTOF 92
Comment			

Acquisition Parameter

Source Type	ESI	Ion Polarity	Positive	Set Nebulizer	2.0 Bar
Focus	Not active			Set Dry Heater	180 °C
Scan Begin	100 m/z	Set Capillary	4500 V	Set Dry Gas	10.0 l/min
Scan End	1500 m/z	Set End Plate Offset	-500 V	Set Divert Valve	Source

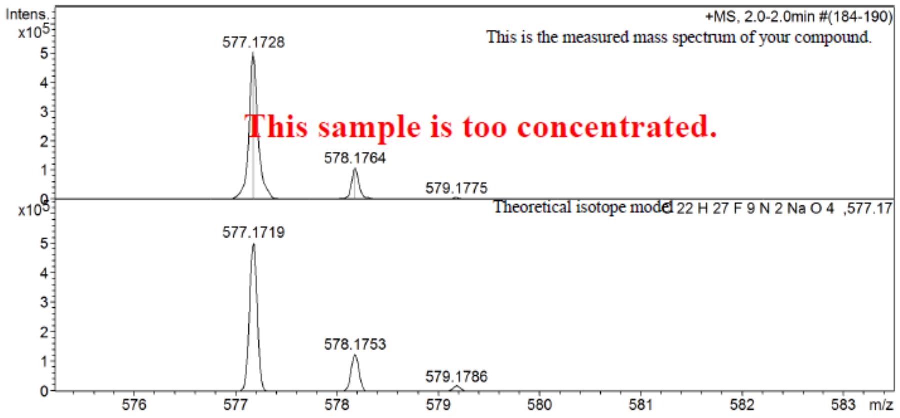

Meas. m/z	#	Formula	m/z	err [ppm]	Mean err [ppm]	rdb	e⁻ Conf	mSigma
577.1728								
	1	C 22 H 27 F 9 N 2 Na O 4	577.1719	-1.4	-1.5	5.5	even	20.07

High resolution mass spectrum of **8**.

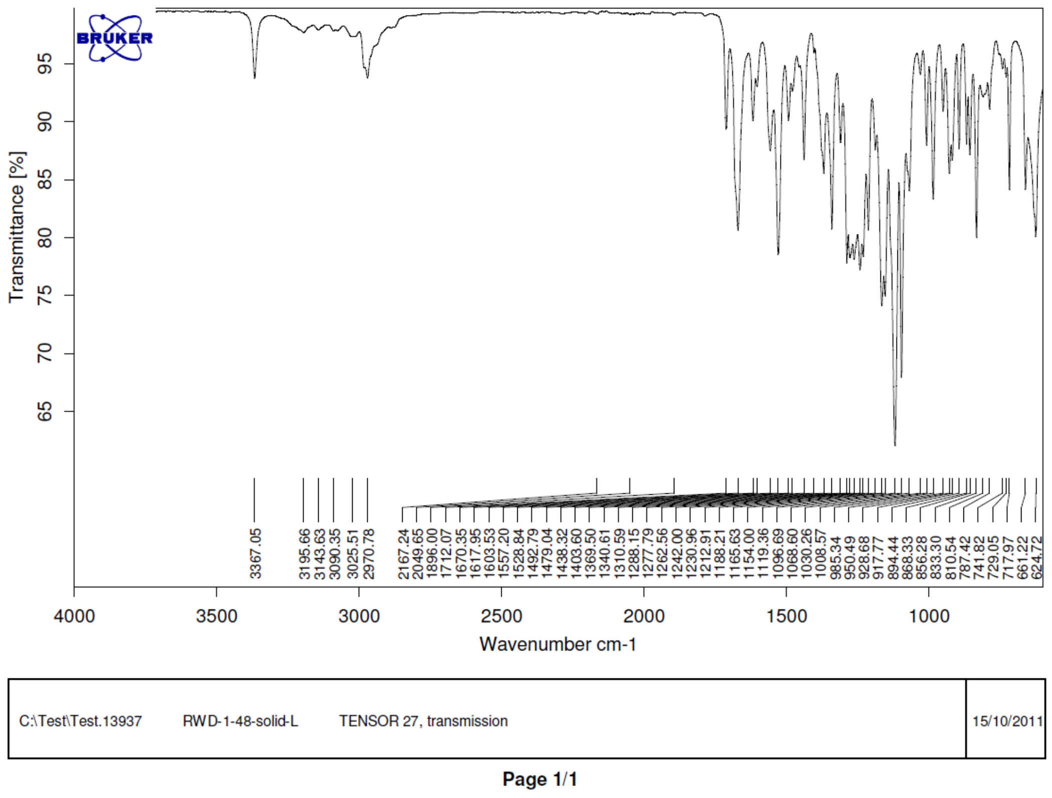

FT-IR of diffraction quality crystals of **8**.

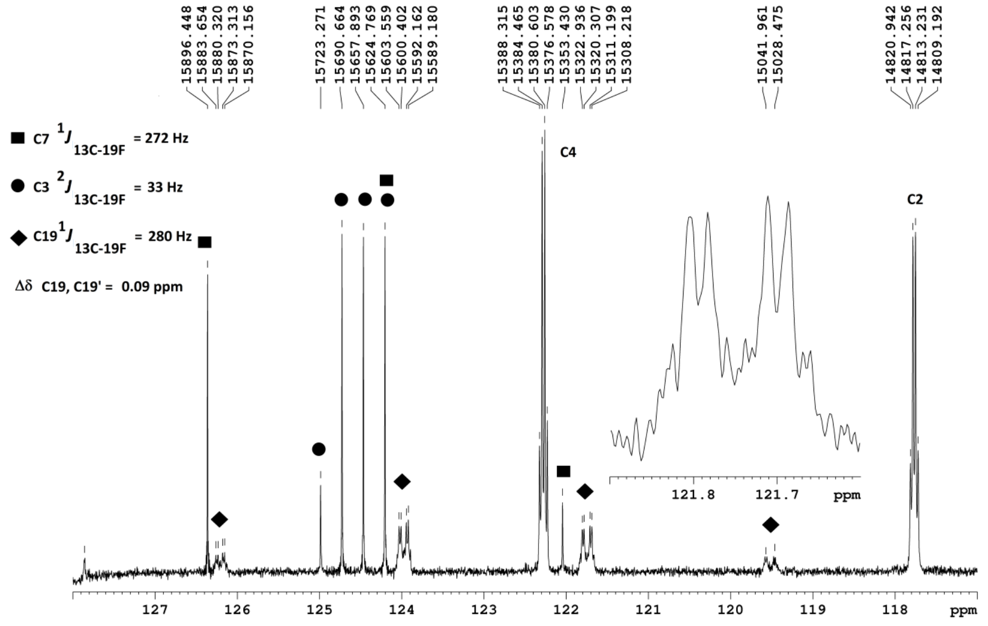

Excerpt from the ^{13}C NMR spectrum of **8** (C_6D_6, 23˚C, 125MHz). Diastereotopic carbons 19 and 19' are anisochronous and appear as two distinct partially overlapping quartets of quartets.

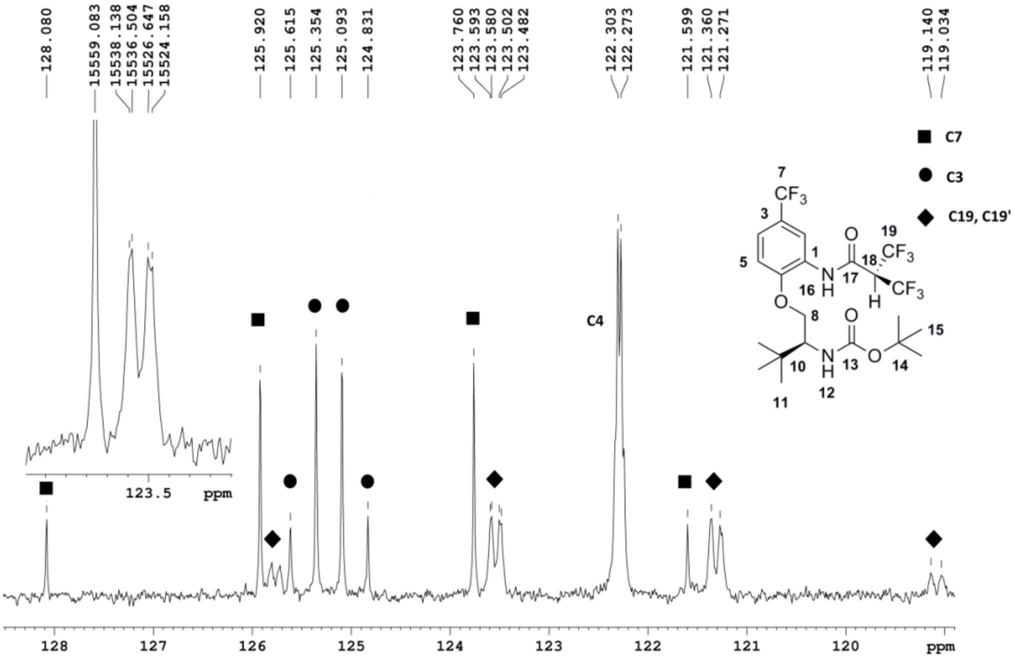

Excerpt from the ^{13}C NMR spectrum of **8** (CCl_4, 23˚C, 125MHz) with acetone-d_6 insert to facilitate locking. Diastereotopic carbons 19 and 19' are anisochronous and appear as two distinct partially overlapping quartets of quartets.

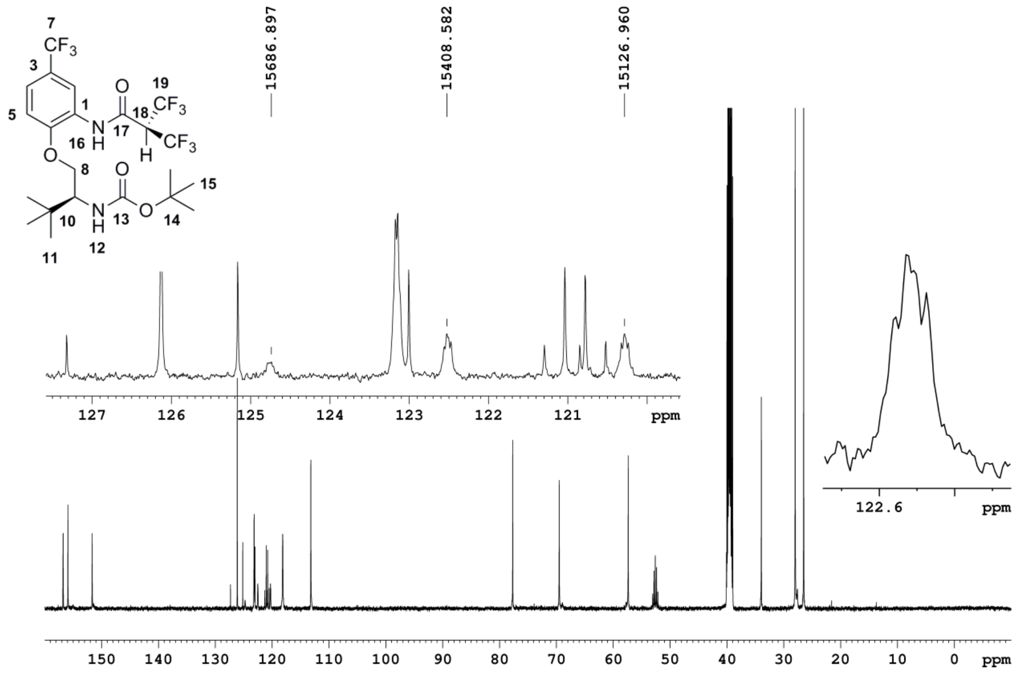

Excerpt from the ^{13}C NMR spectrum of **8** (DMSO-d_6, 23˚C, 125MHz). Diastereotopic carbons 19 and 19' are isochronous and appear as a single quartet of quartets where $^{1}J_{13C-19F}$ = 279 Hz.

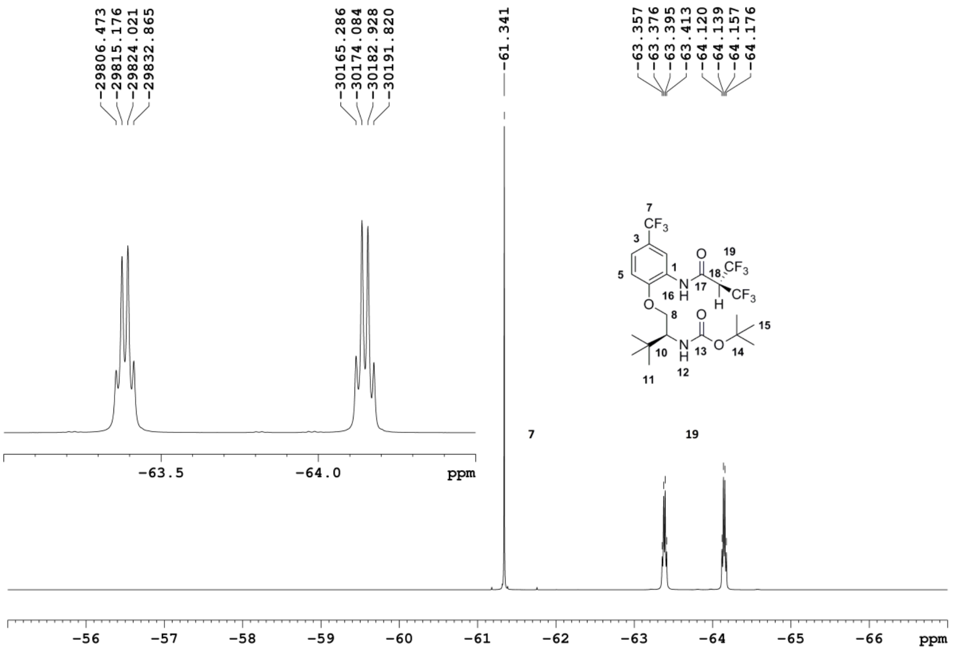

^{19}F (^1H decoupled) NMR spectrum of **8** (C_6D_6, 23˚C 470 MHz) showing two quartets resulting from diastereotopic sets of F19 fluorine atoms.

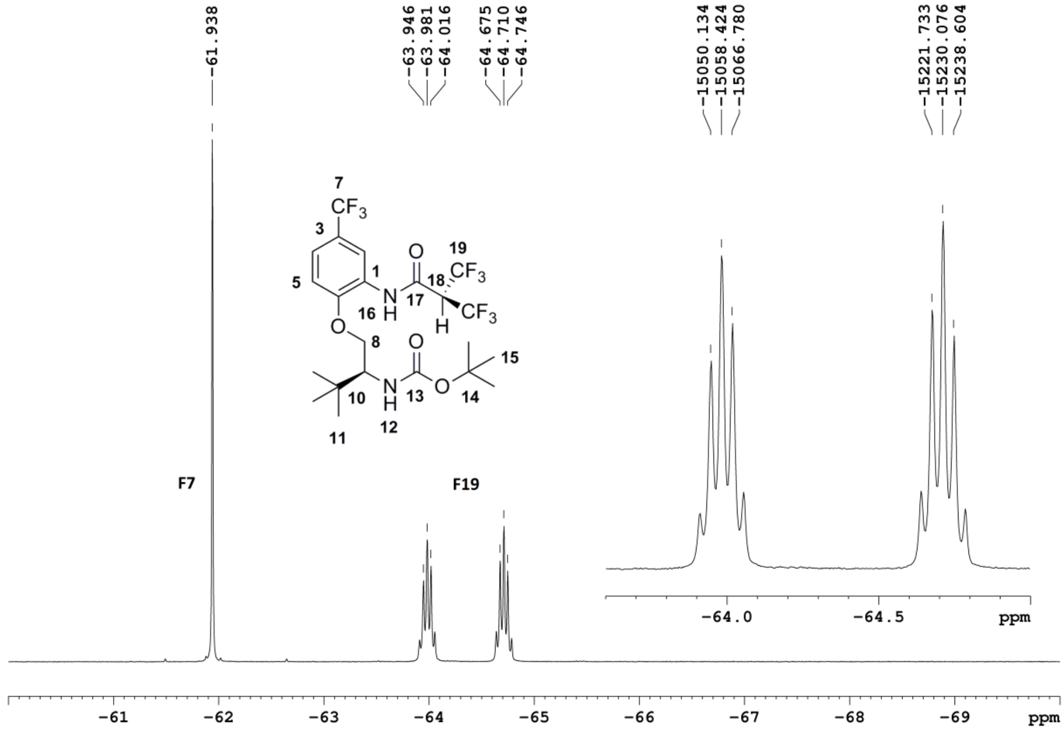

^{19}F (^1H coupled) NMR spectrum of **8** ($CDCl_3$, 23˚C, 235MHz) showing two pentets resulting from diastereotopic sets of ^{19}F fluorine atoms.

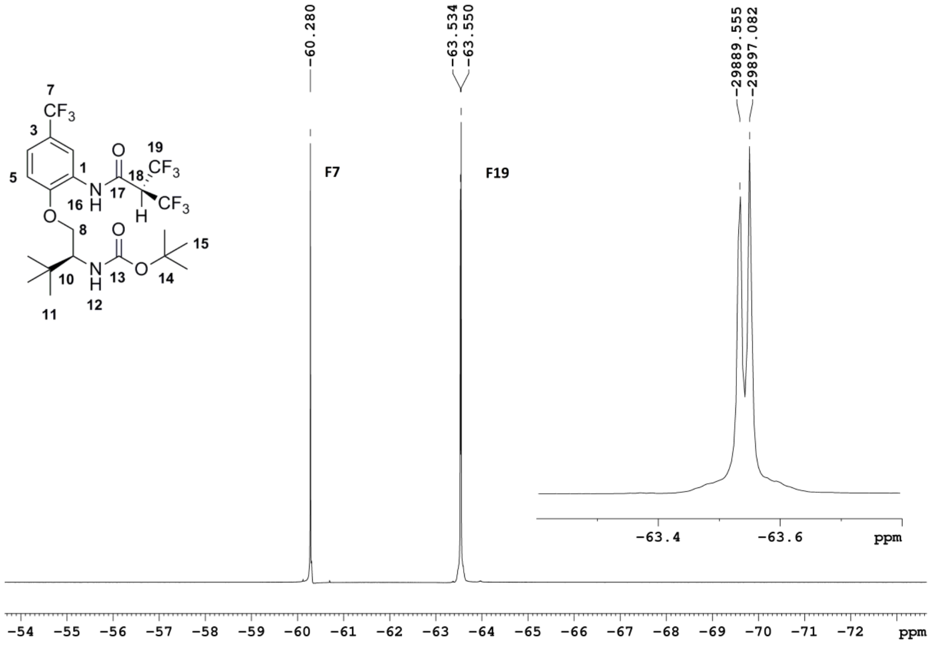

^{19}F (^{1}H coupled) NMR spectrum of **8** (DMSO-d_6, 23˚C, 470 MHz) showing a doublet resulting from homeotopic sets of ^{19}F fluorine atoms.

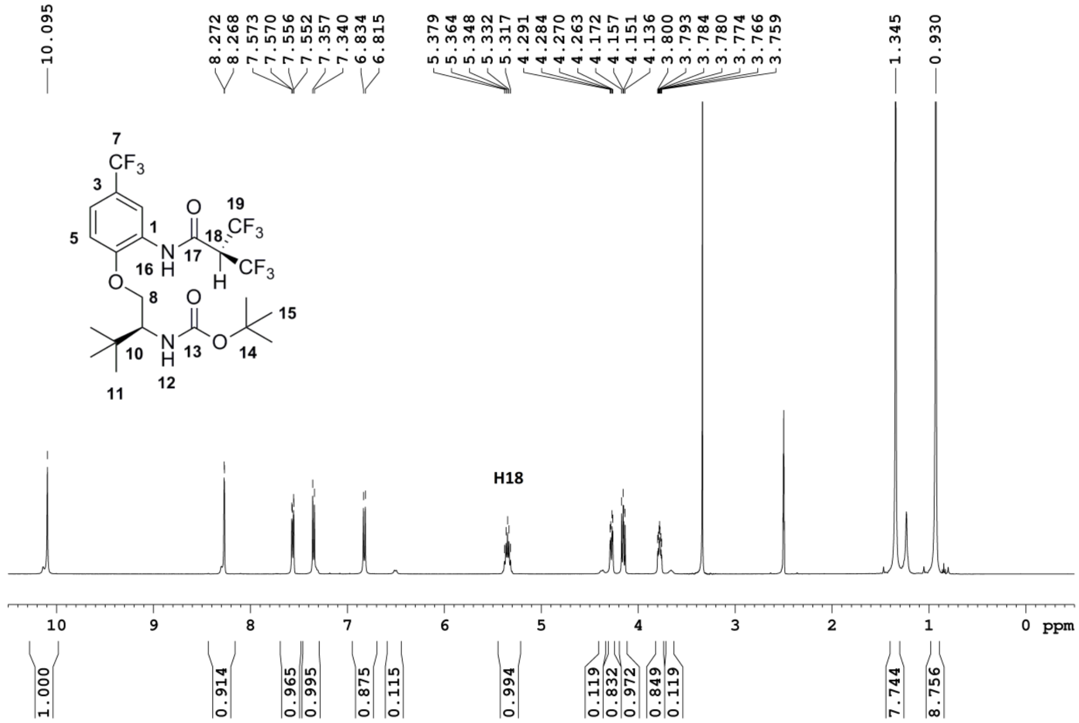

^{1}H NMR spectrum of **8** (DMSO-d_6, 23˚C, 500 MHz). Spectrum from recrystallized material: two conformers are visible.

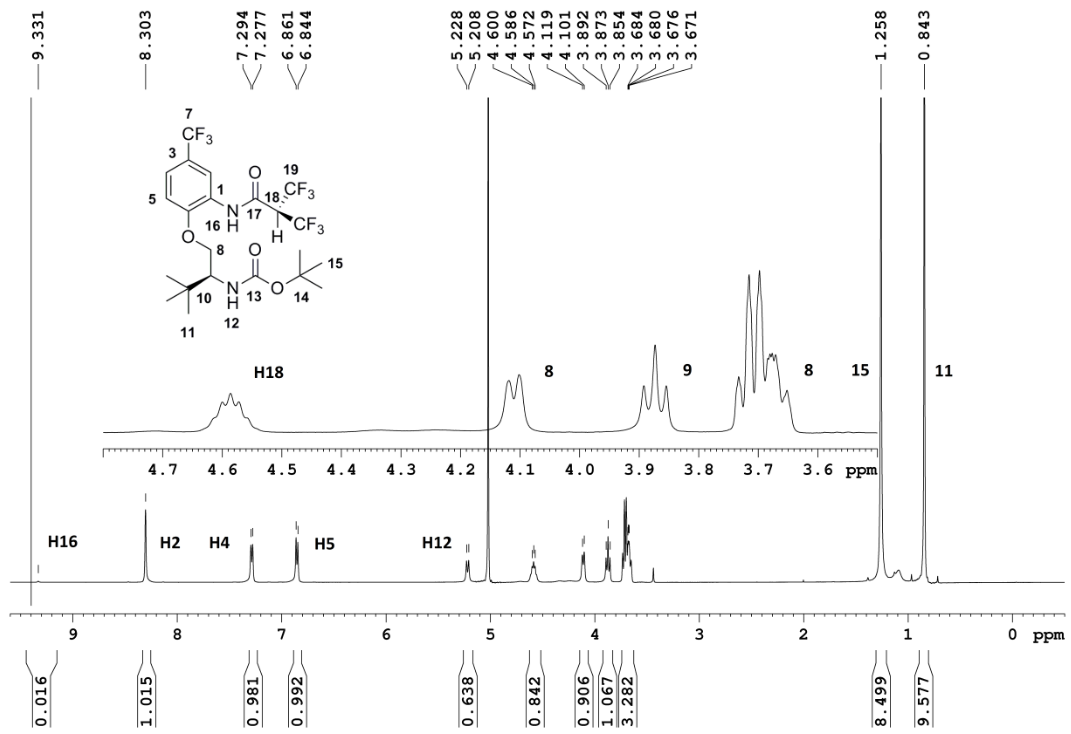

^{1}H NMR spectrum of **8** (CF_3CD_2OD, 23˚C, 500 MHz).

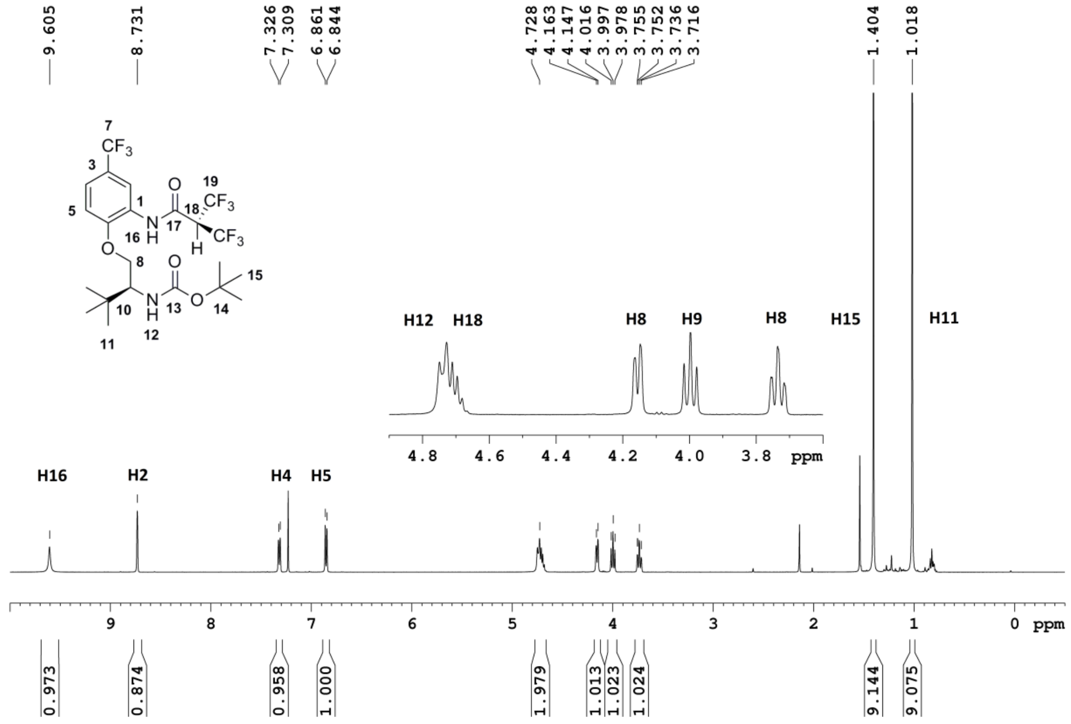

^{1}H NMR spectrum of **8** ($CDCl_3$, 23˚C, 500 MHz).

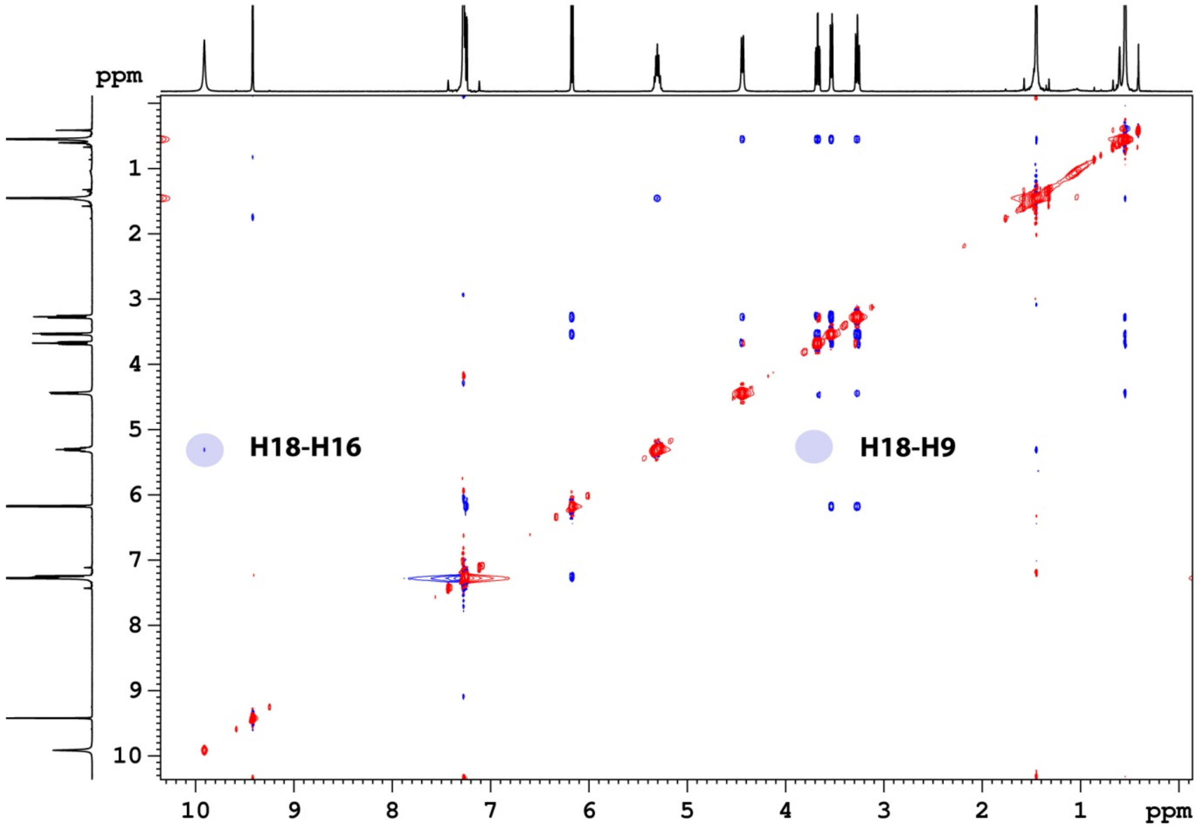

ROESY spectrum of **8** ($CDCl_3$, 298K, 500MHz) showing H18-H16 and H18-H9 crosspeaks.

Solvent effects on hydrogen bonding protons for **8**

	N-pentanes	C_6D_6	$CDCl_3$	CD_3OD	DMSO
δ NH (ppm)	10.20	9.84	9.60	-	10.09
δ CH (ppm)	5.41	5.20	4.72	5.02	5.34
Δ δ CH (CF_3, CF_3')	0.75	0.76	0.58	0	0

(rac)-tert-butyl (1-(2-(cyclopropanecarboxamido)-4-(trifluoromethyl)phenoxy)-3,3-dimethylbutan-2-yl)carbamate [9]

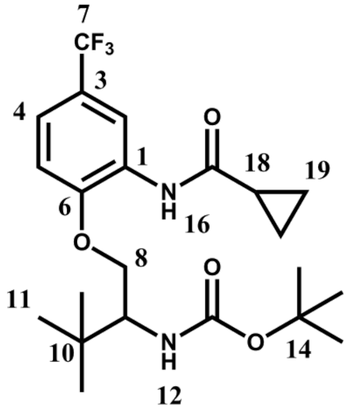

Prepared according to the representative procedure from racemic tert-butyl (1-(2-amino-4-(trifluoromethyl)phenoxy)-3,3-dimethylbutan-2-yl)carbamate on a 0.20 mmol scale. Chromatography (8:1 PET:EtOAc) to yield a white solid (42 mg, 47%) for X-ray crystallographic and NMR studies.

δH (500 MHz, C_6D_6, 23˚C): 9.52 (1H, s, H2), 9.06 (1H, s, H16), 7.16 (1H, dd, J_1 8 Hz, J_2 1 Hz, H4), 6.19 (1H, d, *J* 8 Hz, H5), 4.66 (1H, d, *J* 10 Hz, H12), 3.86 (1H, dt, J_1 10 Hz, J_2 2 Hz, H9), 3.63 (1H, dd, J_1 9 Hz, J_2 2 Hz, H8), 3.23 (1H, t, *J* 9 Hz, H8), 2.17 (1H, m, H18), 1.41 (9H, s, H15), 1.26 (2H, m, H19), 0.63 (9H, s, H11), 0.61 (2H, m, H19).

δC (125 MHz, C_6D_6, 23˚C): 172.88 (C17), 157.56 (C13), 130.54 (C1), 125.75 (q, $^1J_{13C\text{-}19F}$ 270 Hz, C7), 124.07 (q, $^2J_{13C\text{-}19F}$ 31 Hz, C3), 120.12 (q, $^3J_{13C\text{-}19F}$ 4 Hz, C4), 117.17 (q, $^3J_{13C\text{-}19F}$ 4 Hz, C2), 110.48 (C5), 79.77 (C14), 70.29 (C8), 59.28 (C9), 32.73 (C10), 28.77 (C15), 26.93 (C11), 16.23 (C18), 8.82 (C19), 8.62 (C19).

δF (376 MHz, C_6D_6, 23˚C): -61.08

HRMS: (ES-): found 467.2127; Formula $C_{22}H_{31}F_3N_2O_4Na$, [M + Na] requires 467.2128.

ν_{max} (neat, cm^{-1}): 3326.43, 2967.57, 1676.92, 1604.45, 1541.75, 1492.43, 1440.05, 1392.91, 1367.16, 1346.54, 1322.28, 1269.76, 1223.05, 1162.38, 1119.40, 1064.36, 1011.78, 957.26, 924.53, 898.28, 812.96, 636.39.

MP: 166˚C.

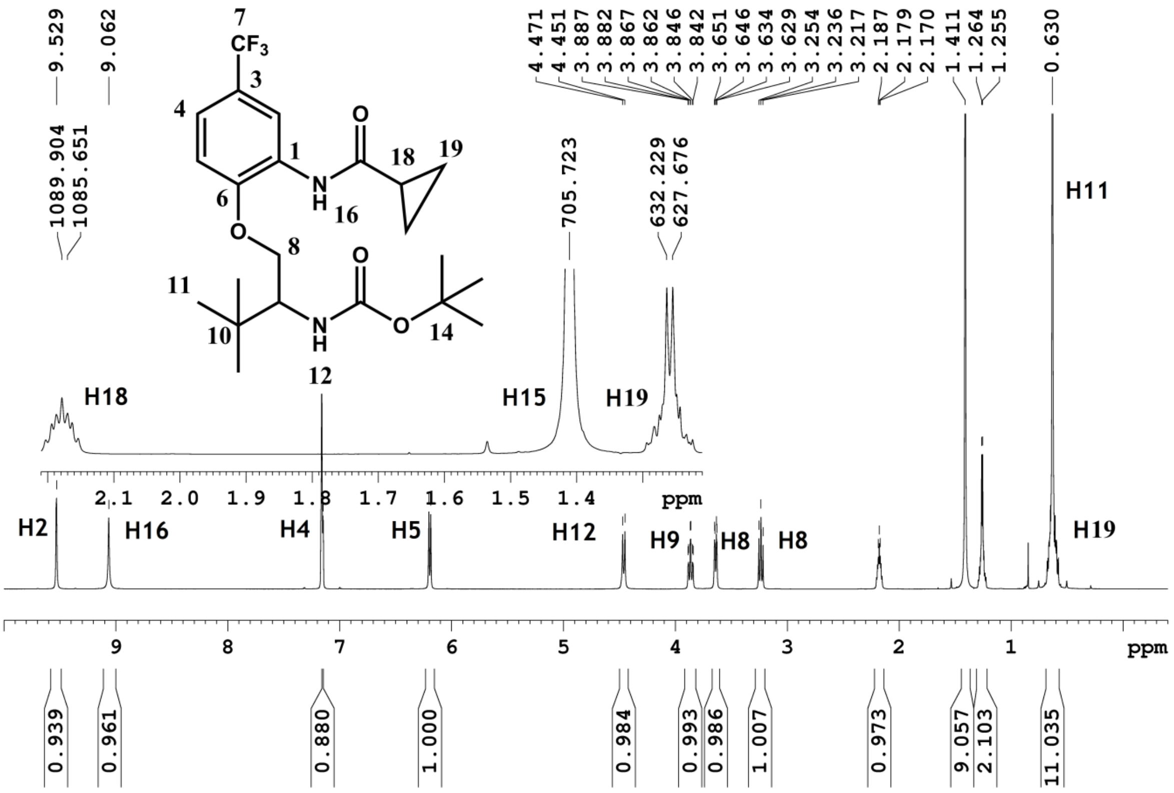

^1H NMR spectrum of **9** (C_6D_6, 23˚C, 500 MHz).

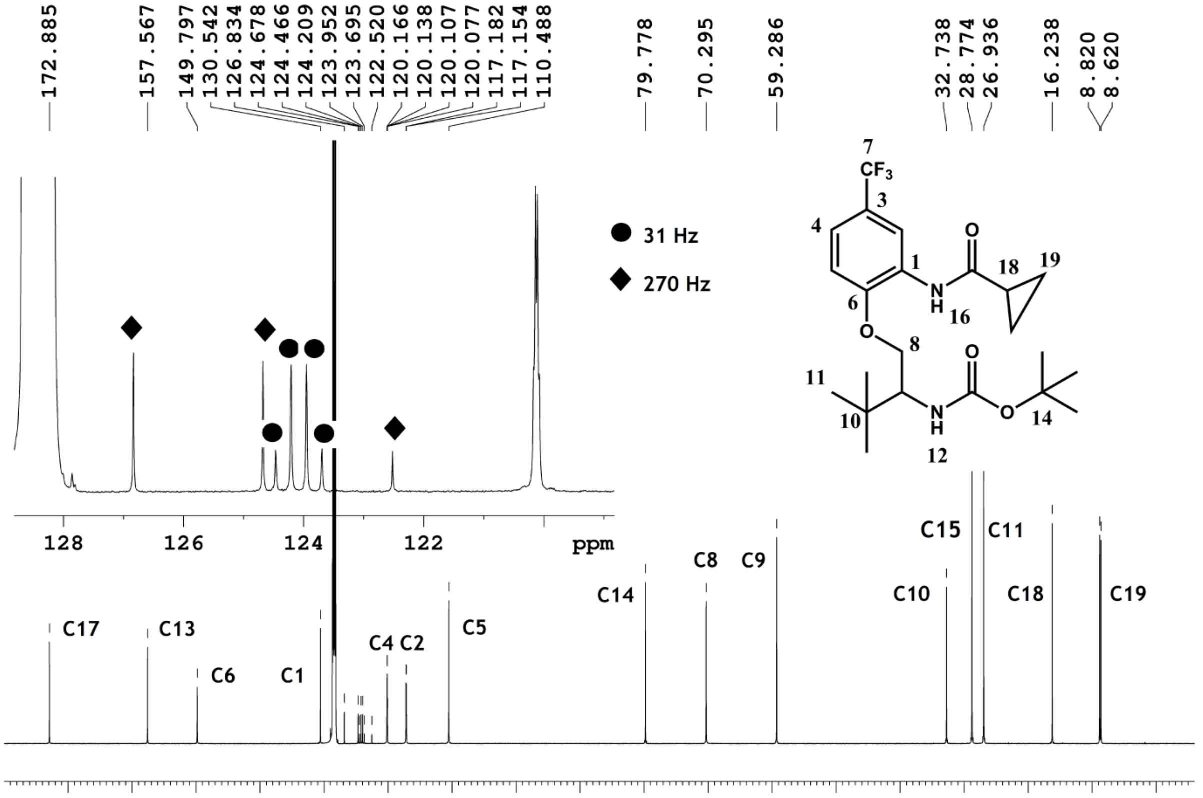

^{13}C NMR spectrum of **9** (C_6D_6, 23˚C, 125 MHz).

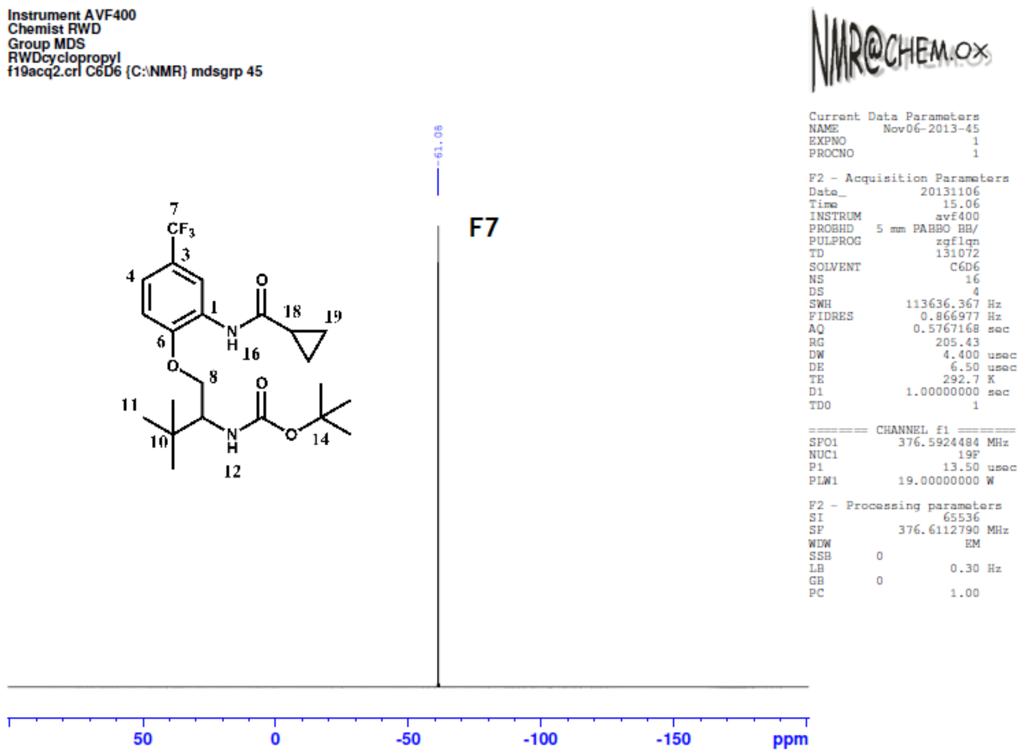

^{19}F NMR spectrum of **9** (23˚C, C_6D_6, 376 MHz).

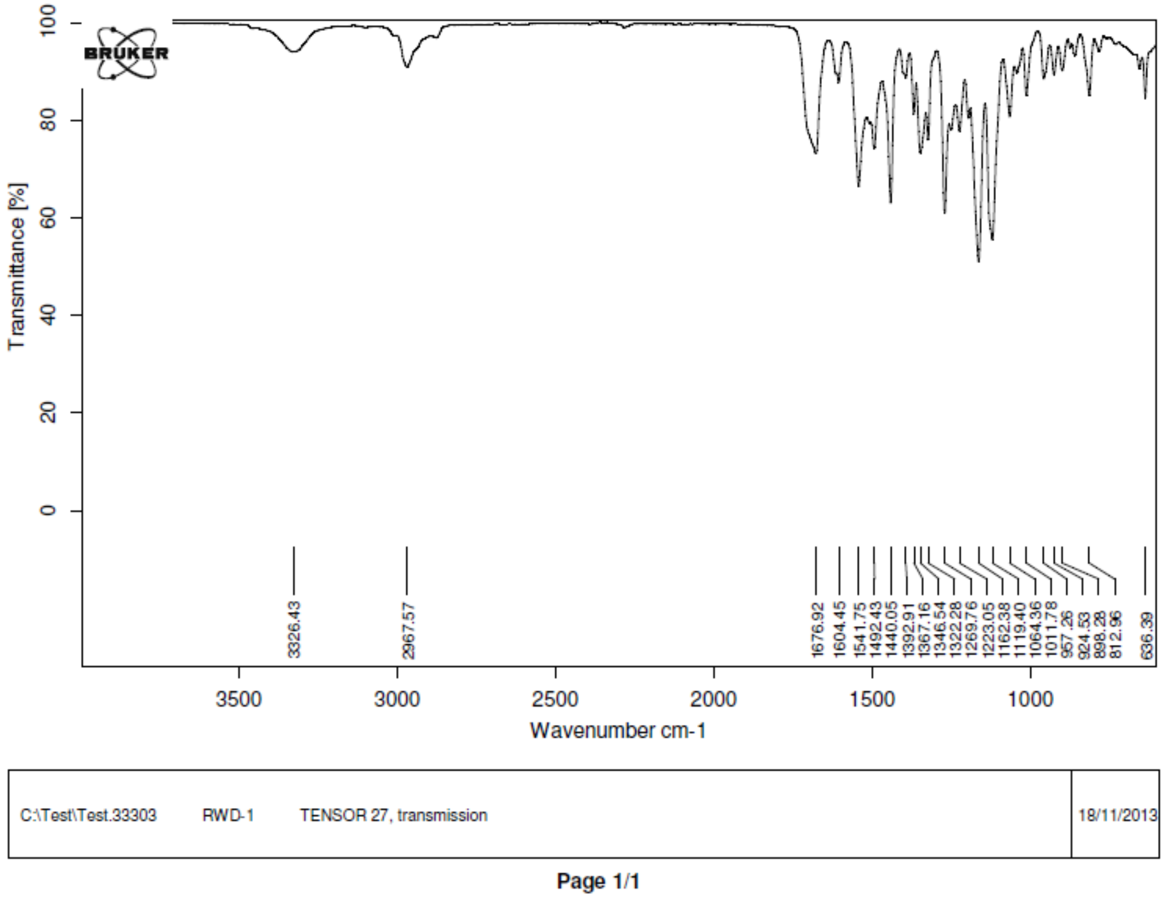

FT-IR of **9**.

Mass Spectrum SmartFormula Report

Analysis Info

Analysis Name	\\Utof\data\Nov 13\ESI43500_5_01_14154.d	Acquisition Date	14/11/2013 07:58:44
Method	2.5min_cal_sample_pos_naf_05-08-13.m	Operator	Mass Spec
Sample Name	ESI43500	Instrument / Ser#	micrOTOF 92
Comment			

Acquisition Parameter

Source Type	ESI	Ion Polarity	Positive	Set Nebulizer	2.0 Bar
Focus	Not active			Set Dry Heater	180 °C
Scan Begin	100 m/z	Set Capillary	4500 V	Set Dry Gas	10.0 l/min
Scan End	1000 m/z	Set End Plate Offset	-500 V	Set Divert Valve	Source

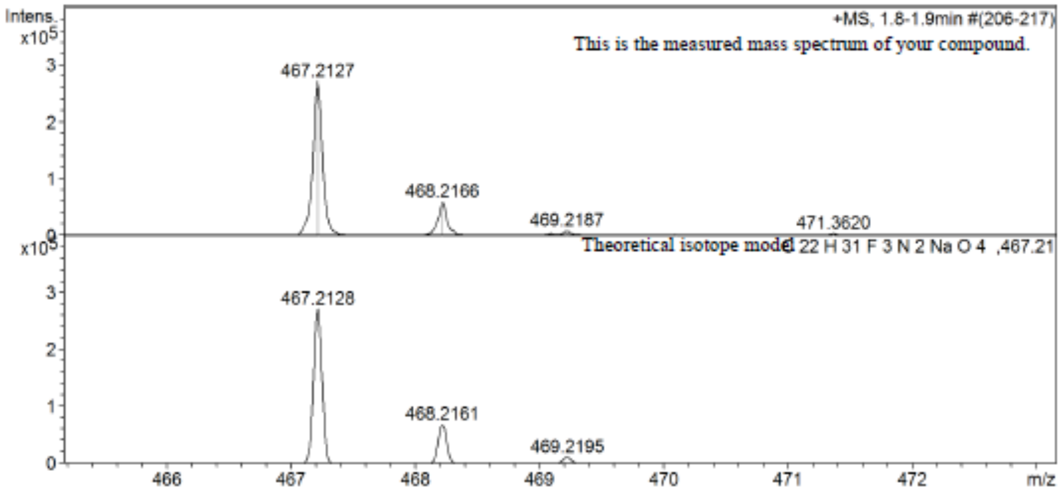

Meas. m/z	#	Formula	m/z	err [ppm]	Mean err [ppm]	rdb	e¯ Conf	mSigma
467.2127								
	1	C 22 H 31 F 3 N 2 Na O 4	467.2128	0.2	-0.0	6.5	even	18.04

High resolution mass spectrum of **9**.

(S)-tert-butyl (1-(2-(2-(2,6-dichlorophenyl)acetamido)-4-(trifluoromethyl)phenoxy)-3,3-dimethylbutan-2-yl)carbamate [10]

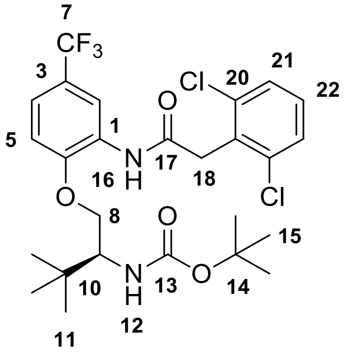

Prepared according to the representative procedure from tert-butyl (1-(2-amino-4-(trifluoromethyl)phenoxy)-3,3-dimethylbutan-2-yl)carbamate (38 mg, 0.098 mmol), 75 mg EDCI.HCl (0.38 mmol), 77 mg 2,6 dichlorobenzoic acid (0.38 mmol), 3 mg N,N-dimethylaminopyridine (0.02 mmol), 1mL pyridine and 5 mL DCM. Chromatography (silica gel, petroleum ether : ethyl acetate 5:1), 2 separations, followed by recrystallization in EtOAc/n-pentane (1:10), 20 mg, 36%.

δH (500 MHz, $CDCl_3$, 23˚C): 8.79 (1H, s, H16), 8.75 (1H, d, *J* 2 Hz, H2), 7.32 (2H, d, *J* 8 Hz, H21), 7.23 (1H, dd, *J*1 8 Hz *J*2 1 Hz, H4), 7.14 (1H,*t*, *J* 8.2 Hz, H22), 6.82 (1H, d, *J* 8 Hz, H5), 4.69 (1H, d, *J* 9 Hz, H12), 4.27 (2H, s, H18), 4.22 (1H, dd, *J*1 8 Hz *J*2 2 Hz, H8), 3.95 (1H, m, *J* 9 Hz, H9), 3.92 (1H, m, 9 Hz, H8), 1.46 (s, 9H, H15), 1.02 (s, 9H, H11).

δH (400 MHz, C_6D_6, 23˚C): 9.46 (1H, s, H16), 8.94 (1H, s, H2), 6.96 (2H, d, *J* 8 Hz, H21), 6.81 (1H, d, *J* 8 Hz, H4), 6.48 (1H, t, *J* 8 Hz, H22), 6.14 (1H, d, *J* 8 Hz, H5), 4.51, 4.48 (2H, apparent dd, J_1 14 Hz, J_2 14 Hz, H18), 4.29 (1H, d, *J* 10 Hz, H12), 3.82 (1H, t, *J* 9 Hz, H9), 3.52 (1H, dd, J_1 8 Hz, J_2 2Hz, H8), 3.19 (1H, d, *J* 10 Hz, H8), 1.46 (9H, s), 0.56 (9H, s).

δC (125 MHz, $CDCl_3$, 23˚C): 167.68 (C17), 157.11 (C13), 149.67 (C6), 136.59 (C20), 131.97 (C19), 129.09 (C1), 128.96 (C22), 128.29 (C21), 124.43 (q, $^1J_{13C\text{-}19F}$ 273 Hz, C7), 123.71 (q, $^2J_{13C\text{-}19F}$ 31 Hz, C3), 120.63 (q, $^3J_{13C\text{-}19F}$ 4 Hz, C4), 117.01 (q, $^3J_{13C\text{-}19F}$ 3Hz, C2), 110.45 (C5), 79.98 (C14), 70.60 (C8), 58.95 (C9), 39.47 (C18), 33.12 (C14), 28.72 (C15), 27.10 (C11).

δF (236 MHz, $CDCl_3$, 23˚C): 62.01 (C7).

HRMS: (ES+Na): found (585.1505); $C_{26}H_{31}F_3N_2O_4Cl_2$, [M + H+] requires 585.1511.

ν_{max} (neat, cm^{-1}): 3264.60, 2967.00, 1691.16, 1603.82, 1541.92, 1493.51, 1438.79, 1334.15, 1270.96, 1163.04, 1120.43, 1065.26, 1011.98, 918.99, 812.20, 766.97, 733.11, 635.49.

MP: 141-142˚C.

$[\alpha]_D^{25.0}$ +28.4 (c = 0.45, $CHCl_3$).

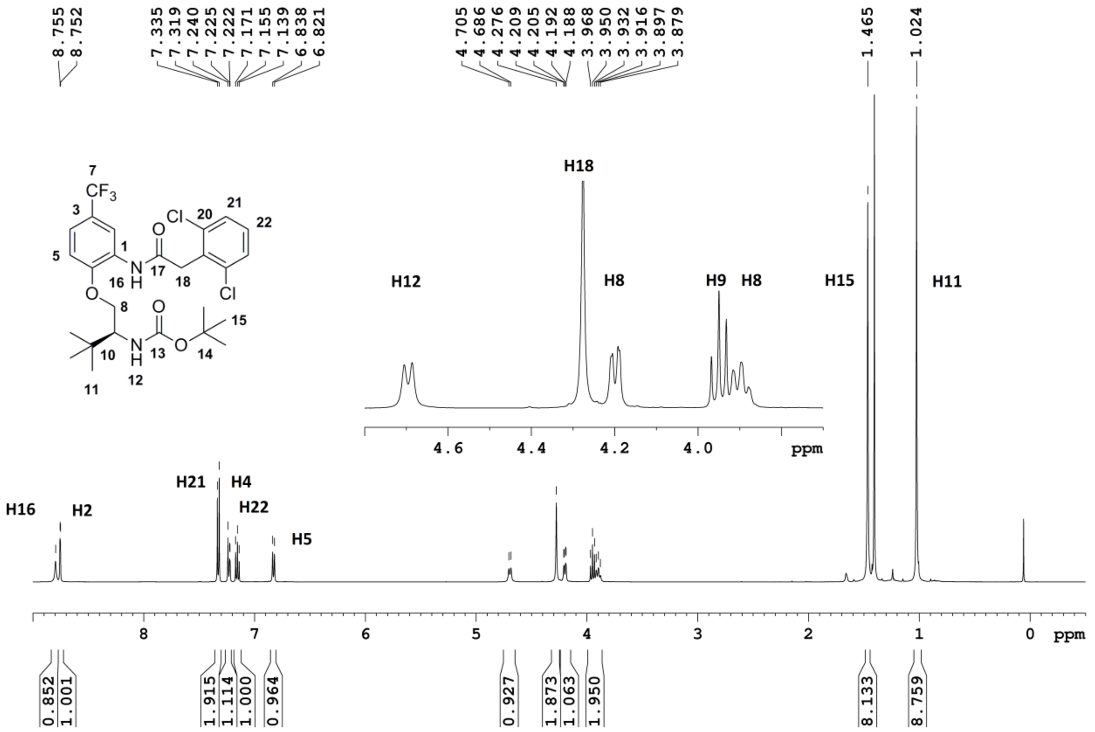

^{1}H NMR spectra of **10** (23˚C, $CDCl_3$, 500 MHz).

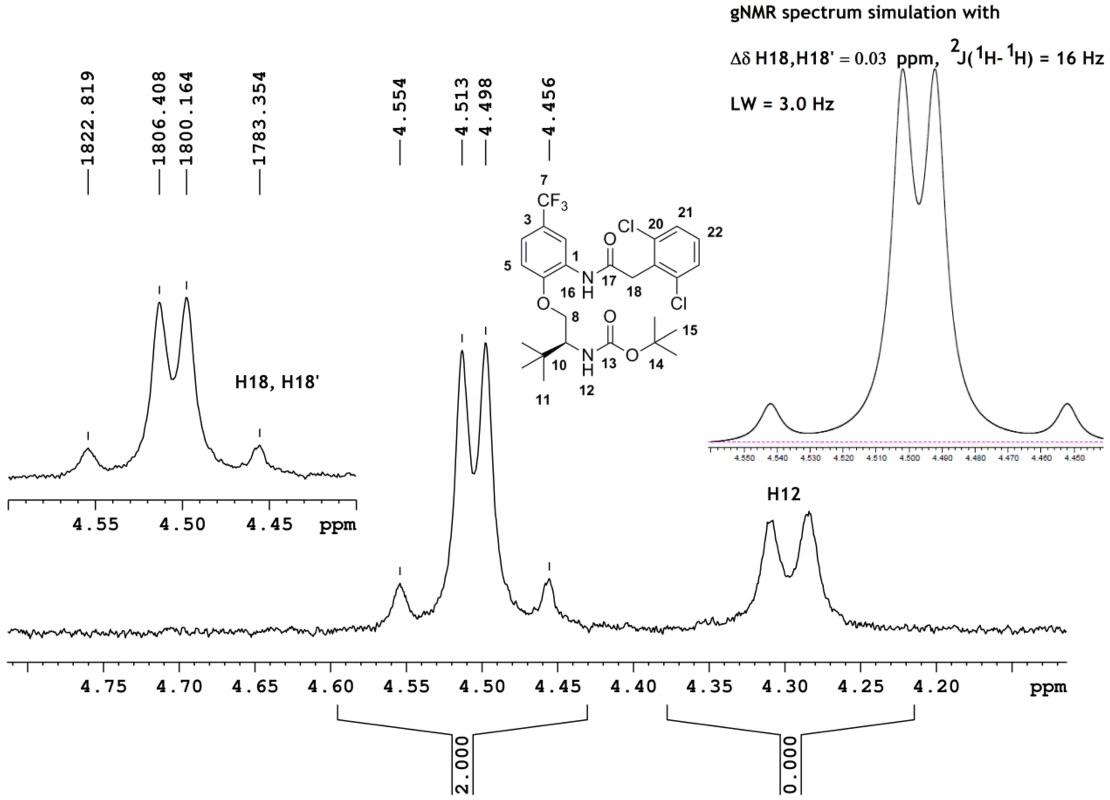

Excerpt from the ^{1}H NMR spectra of **10** (C_6D_6, 23˚C, 400 MHz) showing H18 and H18' to anisochronous; gNMR simulation reproduces the lineshape.

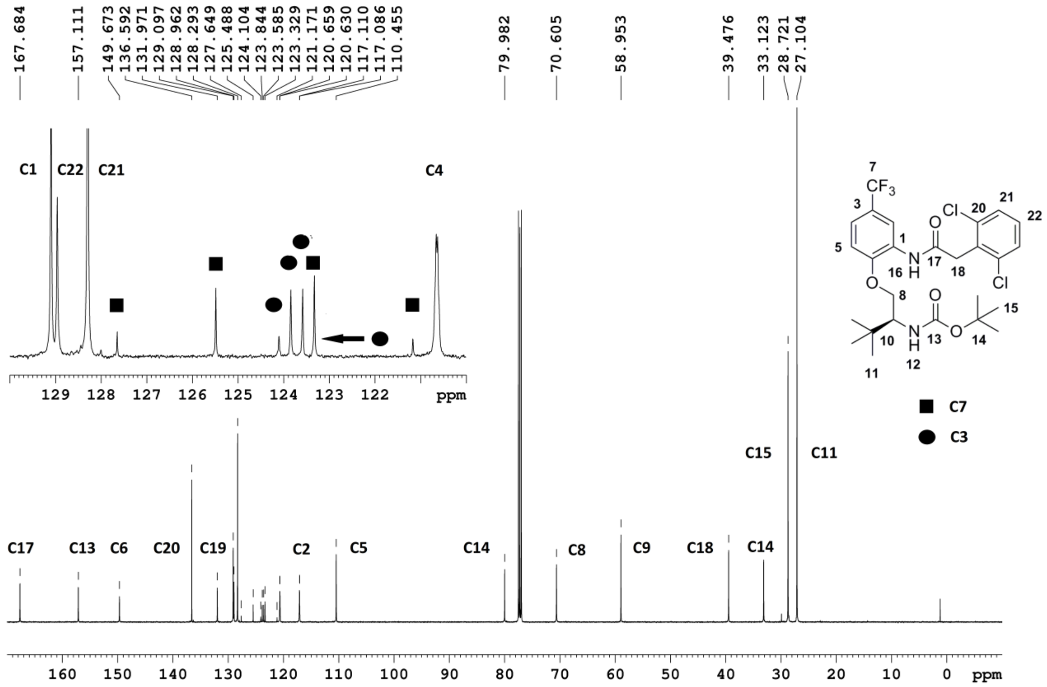

^{13}C NMR spectrum of **10** (23˚C, $CDCl_3$, 125 MHz).

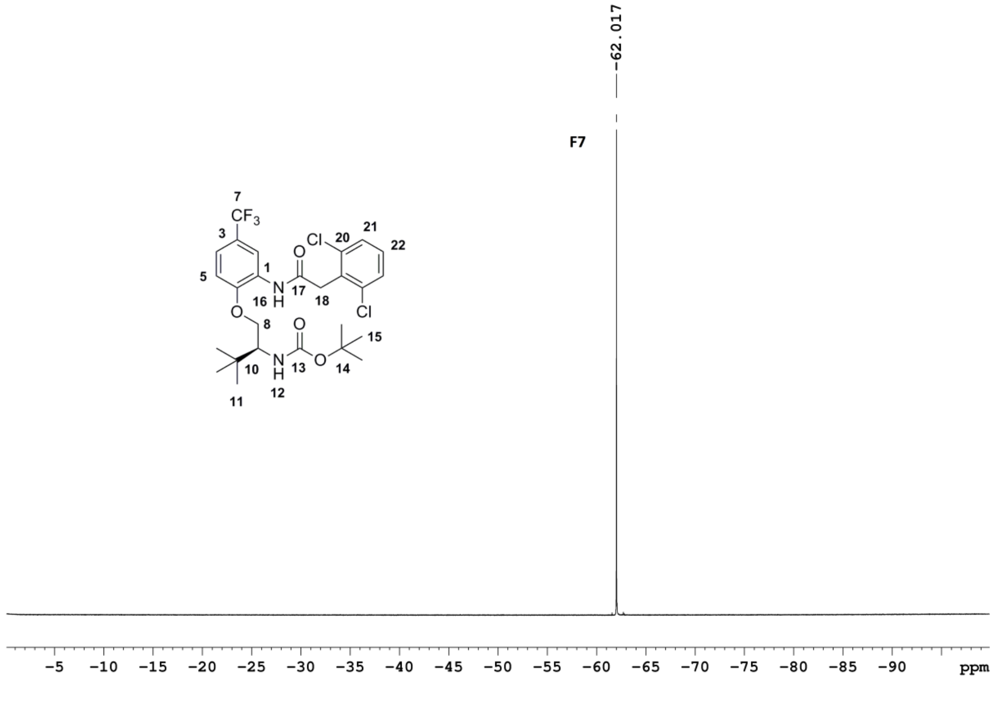

^{19}F NMR spectrum of **10** (23˚C, $CDCl_3$, 235 MHz).

Mass Spectrum SmartFormula Report

Analysis Info

Analysis Name	\\Utof\Data\Sep 11\ESI29944_7_01_31870.d	Acquisition Date	01/09/2011 10:46:57
Method	2.5min_cal_sample_pos_Naf_11-10-10.m	Operator	Mass Spec
Sample Name	ESI29944	Instrument / Ser#	micrOTOF 92
Comment			

Acquisition Parameter

Source Type	ESI	Ion Polarity	Positive	Set Nebulizer	2.0 Bar
Focus	Not active			Set Dry Heater	180 °C
Scan Begin	100 m/z	Set Capillary	4500 V	Set Dry Gas	10.0 l/min
Scan End	1500 m/z	Set End Plate Offset	-500 V	Set Divert Valve	Source

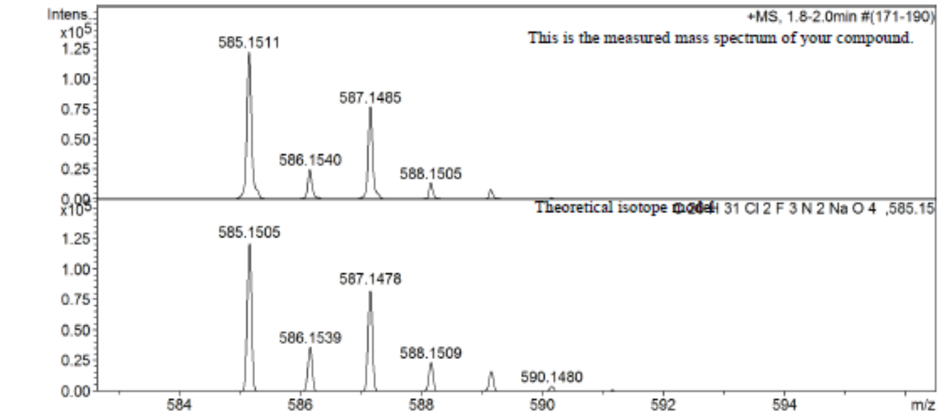

Meas. m/z	#	Formula	m/z	err [ppm]	Mean err [ppm]	rdb	e⁻ Conf	mSigma
585.1511								
	1	C 26 H 31 Cl 2 F 3 N 2 Na O 4	585.1505	-0.9	-0.9	9.5	even	56.77

High Resolution mass spectrum of **10**.

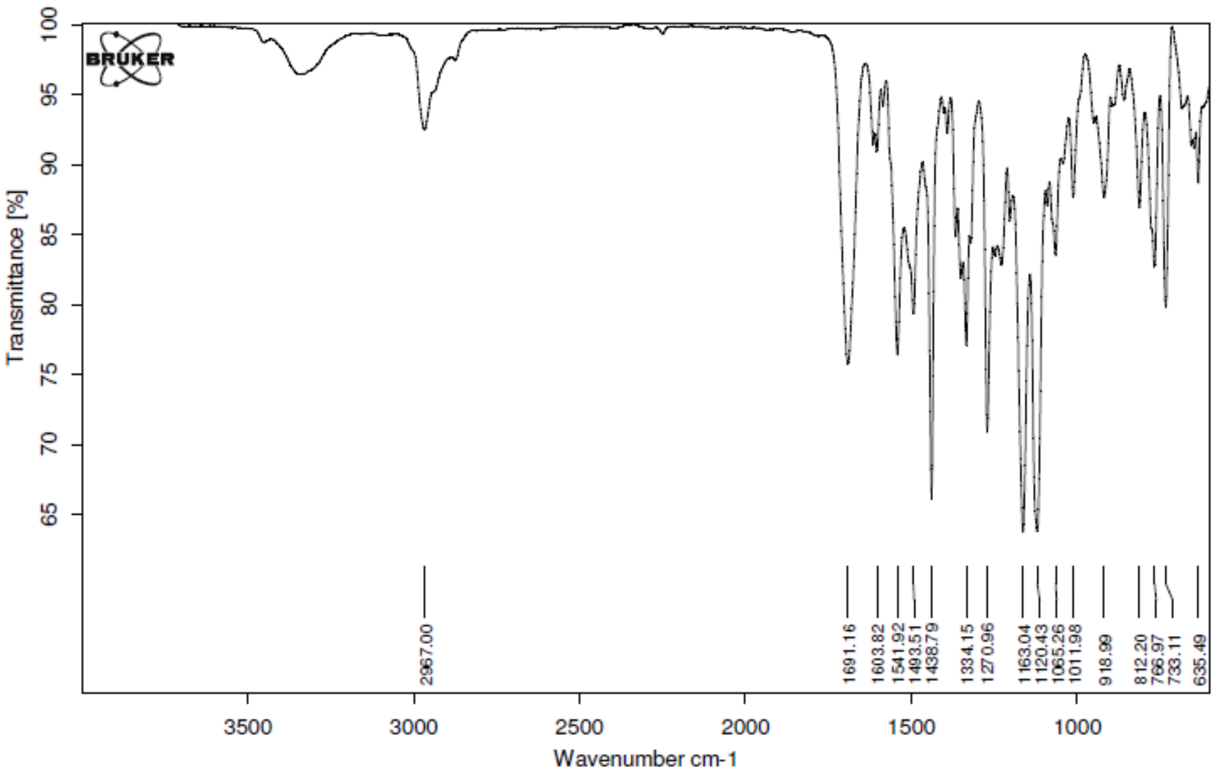

Solution State FT-IR of **10**.

(S)-tert-butyl (3,3-dimethyl-1-(2-(2-(4-nitrophenyl)acetamido)-4-(trifluoromethyl)phenoxy)butan-2-yl)carbamate [11]

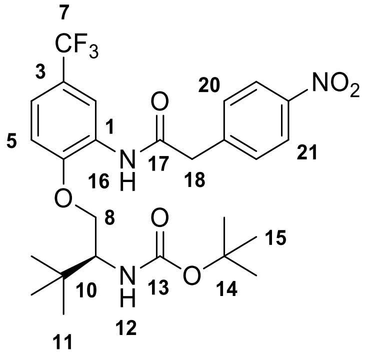

Prepared according to the representative procedure from (S)-tert-butyl (1-(2-amino-4-(trifluoromethyl)phenoxy)-3,3-dimethylbutan-2-yl)carbamate (75 mg, 0.196 mmol), 149 mg EDCI.HCl (.75 mmol), 141 mg 4-nitrobenzoic acid (0.78 mmol), 7 mg N,N-dimethylaminopyridine (0.05 mmol) and 5 mL pyridine. Chromatography (silica gel, petroleum ether : ethyl acetate 5:1), 2 separations, followed by recrystallization in EtOAc/n-pentane (1:10), 45 mg, 43%.

δH (500 MHz, C_6D_6, 23˚C): 9.46 (1H, s, H16), 8.85 (1H, d, *J* 1.9 Hz, H2), 7.80 (2H, d, *J* 9.0 Hz, H21), 7.15 (2H, d, *J* 9.0 Hz, H20), 7.15 (1H, obscured, H3), 6.15 (1H, d, *J* 8.6 Hz, H4), 4.29 (1H, d, *J* 10.8 Hz, H12), 3.84 (1H, d, *J* 15.2 Hz, H18), 3.79 (1H, d, *J* 9.0 Hz, H8), 3.69 (1H, d, *J* 15.2 Hz, H18), 3.52 (1H, dd, *J*1 9.0 Hz, *J*2 4.2 Hz, H8), 3.15 (1H, t, *J* 7.5 Hz, H9), 1.38 (9H, s, H15), 0.54 (9H, s, H11).

δH (500 MHz, $CDCl_3$, 23˚C): 8.95 (1H, s, H16), 8.73 (1H, d, *J* 1.9 Hz, H2), 8.19 (2H, d, *J* 8.6 Hz, H21), 7.60 (2H, d, *J* 8.6 Hz, H20), 7.27 (1H, dd, J_1 8.5 Hz, J_2 1.4 Hz, H3), 6.82 (1H, d, *J* 8.5 Hz, H4), 4.72 (1H, d, *J* 9.4 Hz, H12), 4.21 (1H, d, *J* 7.5 Hz, H8), 4.08 (1H, d, *J* 14.9 Hz, H18), 3.95-3.86 (3H, H18, H9, H8), 1.44 (9H, s, H15), 1.05 (9H, s, H11).

δC (125 MHz, $CDCl_3$, 23˚C): 168.32 (C17), 157.41 (C13), 149.86 (C6), 147.28 (C21), 142.80 (C19), 130.61 (C20), 128.77 (C1), 124.37 (C7, q, $^1J_{C-F}$ = 272 Hz), 123.87 (C21), 123.72 (C3, q, $^2J_{C-F}$ = 32 Hz), 121.00 (C4, d, $^3J_{C-F}$ = 4 Hz), 116.95 (C2, d, $^3J_{C-F}$ = 3 Hz), 110.43 (C5), 79.95 (C14), 70.84 (C8), 59.35 (C9), 43.81 (C18), 32.87 (C10), 28.67 (C15), 27.10 (C11).

δF (236 MHz, $CDCl_3$, 23˚C): -62.08 (F7).

HRMS: (ES+): found (539.2246); $C_{26}H_{32}F_3N_3O_6$, [M + H+] required 539.2243.

ν_{max} (neat, cm^{-1}): 3264.50, 2968.77, 1688.79, 1604.69, 1520.74, 1440.77, 1345.91, 1271.80, 1163.59, 1121.16, 1065.04, 1011.90, 908.18, 856.99, 814.76, 731.93, 635.93.

MP: 152-153 ˚C.

$[\alpha]_D^{25.0}$ +56.5 (c = 0.002, $CHCl_3$).

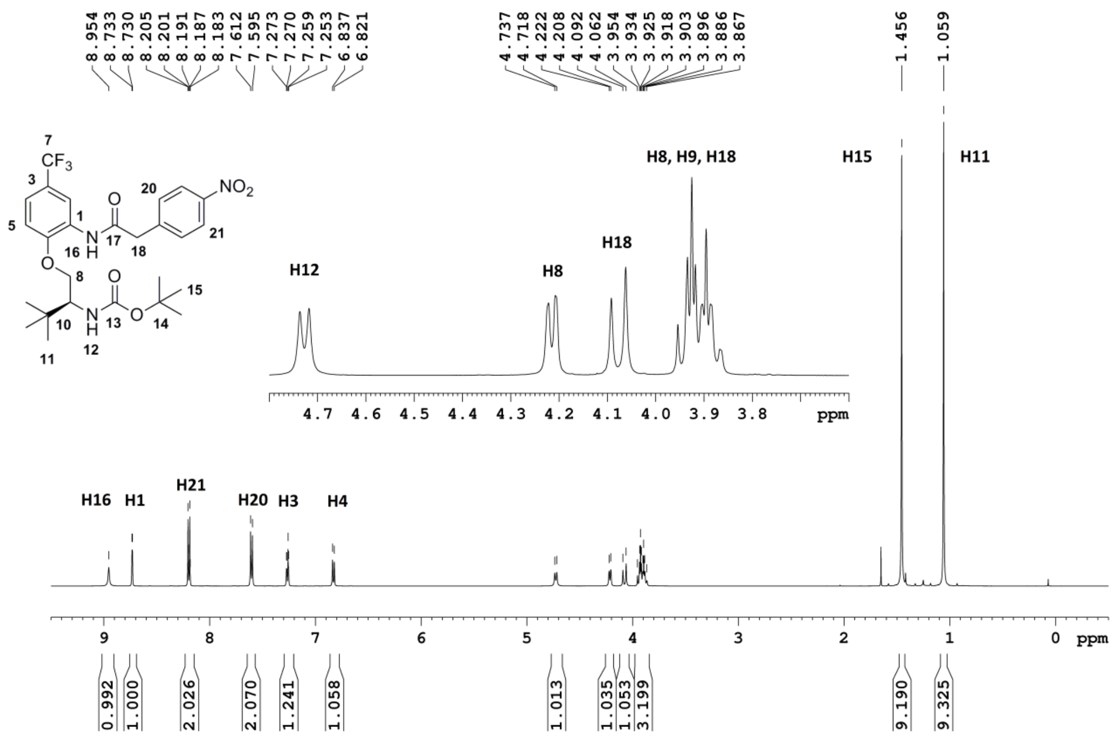

^{1}H NMR spectrum of **11** (23˚C, $CDCl_3$, 500 MHz).

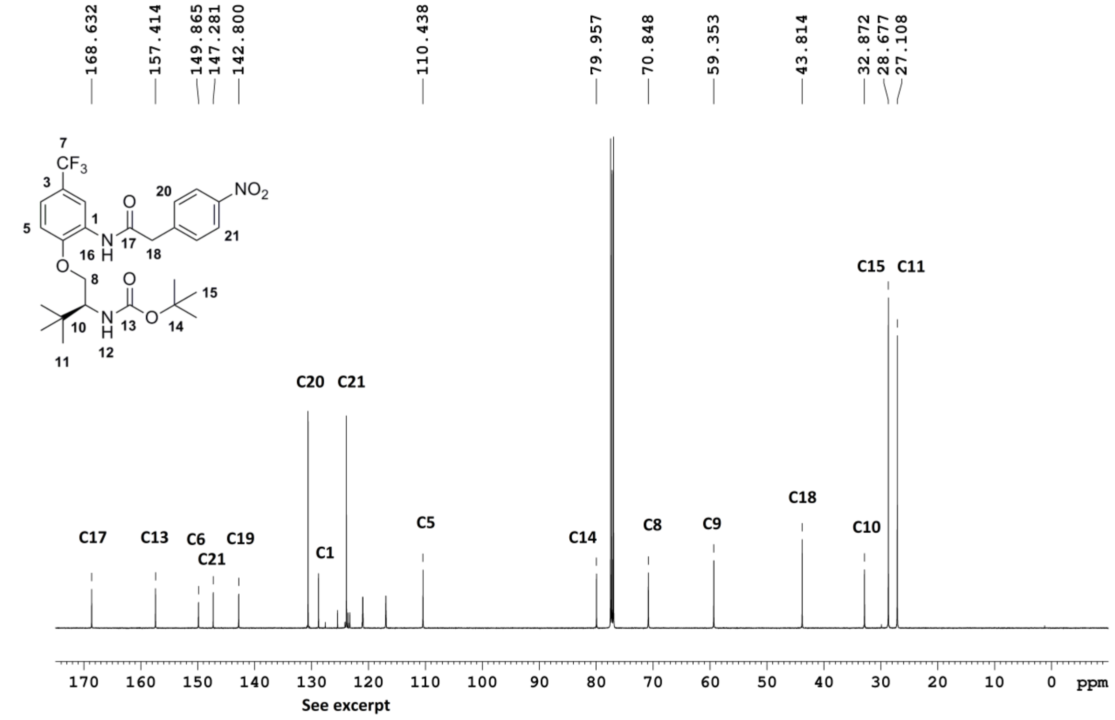

^{13}C NMR spectrum of **11** (23˚C, $CDCl_3$, 125 MHz).

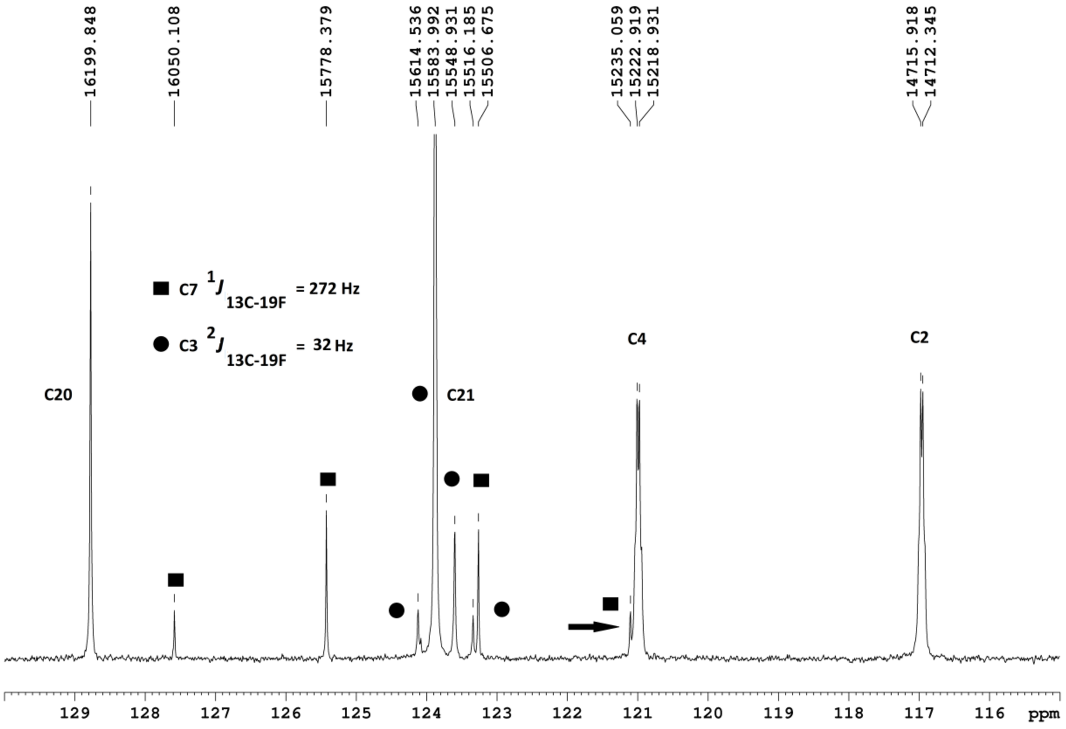

Excerpt from the ^{13}C NMR spectrum of **11** (23˚C, $CDCl_3$, 500 MHz).

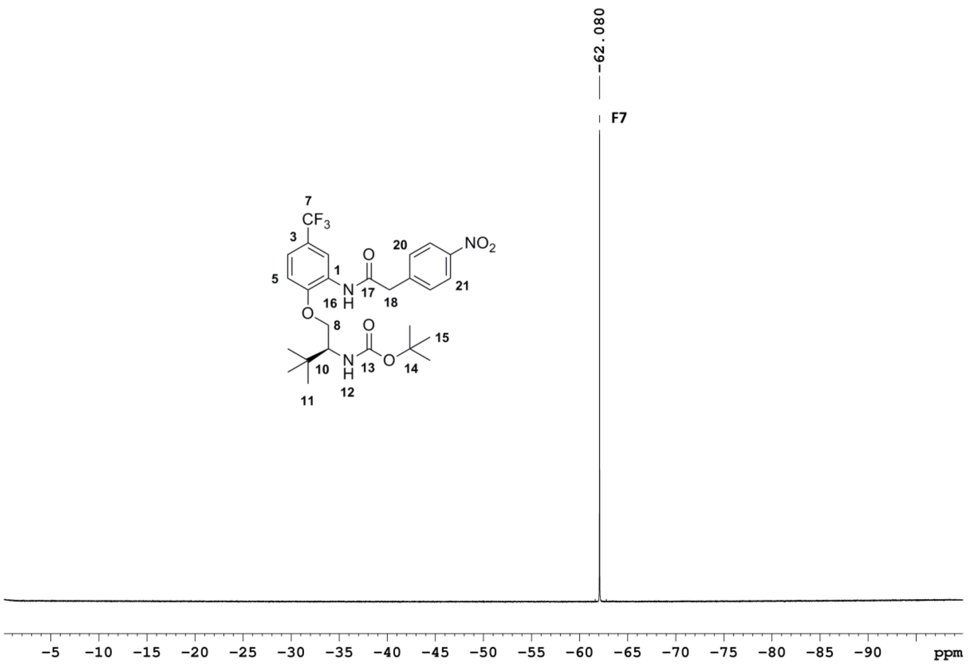

^{19}F NMR spectrum of **11** (23˚C, $CDCl_3$, 470 MHz).

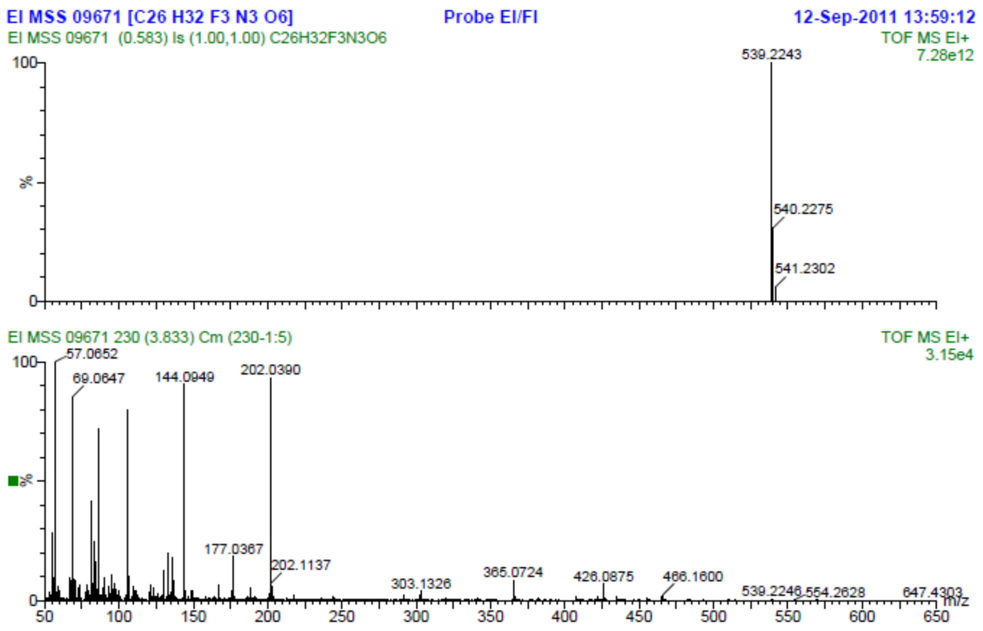

High resolution mass spectrum of **11**.

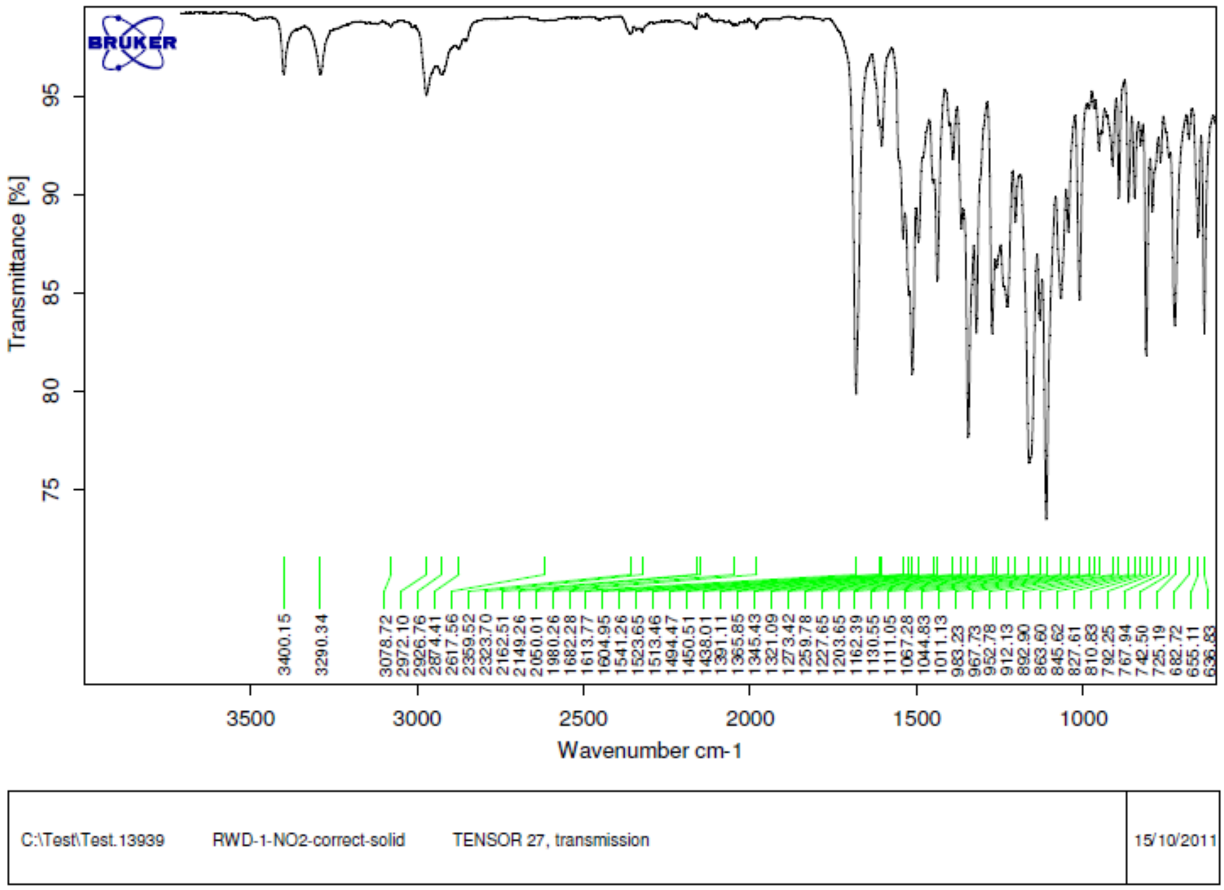

FT-IR of diffraction quality crystals of **11**.

tert-butyl ((-3,3-dimethyl-1-(2-(2-(perfluorophenyl)-2-phenylacetamido)-4-(trifluoromethyl)phenoxy)butan-2-yl)carbamate [12]

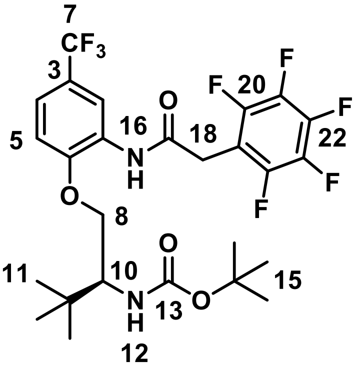

Prepared according to the representative procedure from tert-butyl (1-(2-amino-4-(trifluoromethyl)phenoxy)-3,3-dimethylbutan-2-yl)carbamate (100 mg, 0.52 mmol), 300 mg EDCI.HCl (1.52 mmol), 340 mg 2,3,4,5,6 pentafluorobenzoic acid (1.52 mmol), 5 mg N,N-dimethylaminopyridine (.04 mmol) and 0.3 mL pyridine. Chromatography (silica gel, petroleum ether : ethyl acetate 5:1), yielded 65 mg, 11% of the title compound as a white crystalline solid.

δH (500 MHz, C_6D_6, 23˚C): 9.39 (1H, d, *J* 2 Hz, H2), 9.07 (1H, bs, H16), 7.14 (1H, d, *J* 8 Hz, H4), 6.15 (1H, d, *J* 8 Hz, H5), 4.03 (1H, d, *J* 10 Hz, H18), 3.92 (1H, d, *J* 10 Hz, H18), 3.79 (1H, dt J_1 10 Hz, J_2 2 Hz, H9), 3.57 (1H, dd J_1 9 Hz, J_2 2 Hz, H8), 3.18 (1H, t *J* 10 Hz, H8), 1.43 (9H, s, H15), 0.56 (9H, s, H11).

δC (125 MHz, C_6D_6, 23˚C): 166.13 (C17), 157.70 (C13), 150.06 (C6), 147.24 (C20), 145.29 (C20), 141.97 (C22), 139.97 (C22), 139.09 (C21), 137.10 (C21), 129.95 (C1), 125.53 (C7, q, $^1J_{13C\text{-}19F}$ 272 Hz), 124.47 (C3, q, $^2J_{13C\text{-}19F}$ 32 Hz), 122.28 (C4, q, $^3J_{13C\text{-}19F}$ 4 Hz), 117.48 (C2, q, $^3J_{13C\text{-}19F}$ 4 Hz), 110.99 (C5), 109.63 (C19), 80.05 (C14), 70.77 (C8), 59.55 (C9), 32.58 (C10), 30.94 (C18), 28.76 (C15), 26.85 (C11).

δF (470 MHz, C_6D_6, 23˚C): -61.35 (F7), -143.01 (F20, dd, $^3J_{19F\text{-}19F}$ 22 Hz, $^4J_{19F\text{-}19F}$ 12 Hz), -158.48 (F22, t, J 22 Hz), -163.41 (F21, dt, $^3J_{19F\text{-}19F}$ 22 Hz, $^4J_{19F\text{-}19F}$ 12 Hz).

ν_{max} (neat, cm^{-1}): 3351.39, 2971.58, 1736.75, 1691.30, 1615.35, 1605.02, 1545.91, 1522.12, 1507.14, 1440.42, 1392.34, 1336.06, 1322.09, 1303.44, 1271.05, 1243.18, 1164.09, 1122.99, 1064.08, 1045.39, 1010.24, 981.50, 949.52, 906.52, 857.99, 815.45, 783.40, 750.96, 730.58, 685.58, 655.68, 635.63, 609.86.

HRMS: (ES+): $C_{26}H_{28}F_8N_2O_4Na$ [requires 607.1816]; observed 607.1814.

M.p.: 130°C

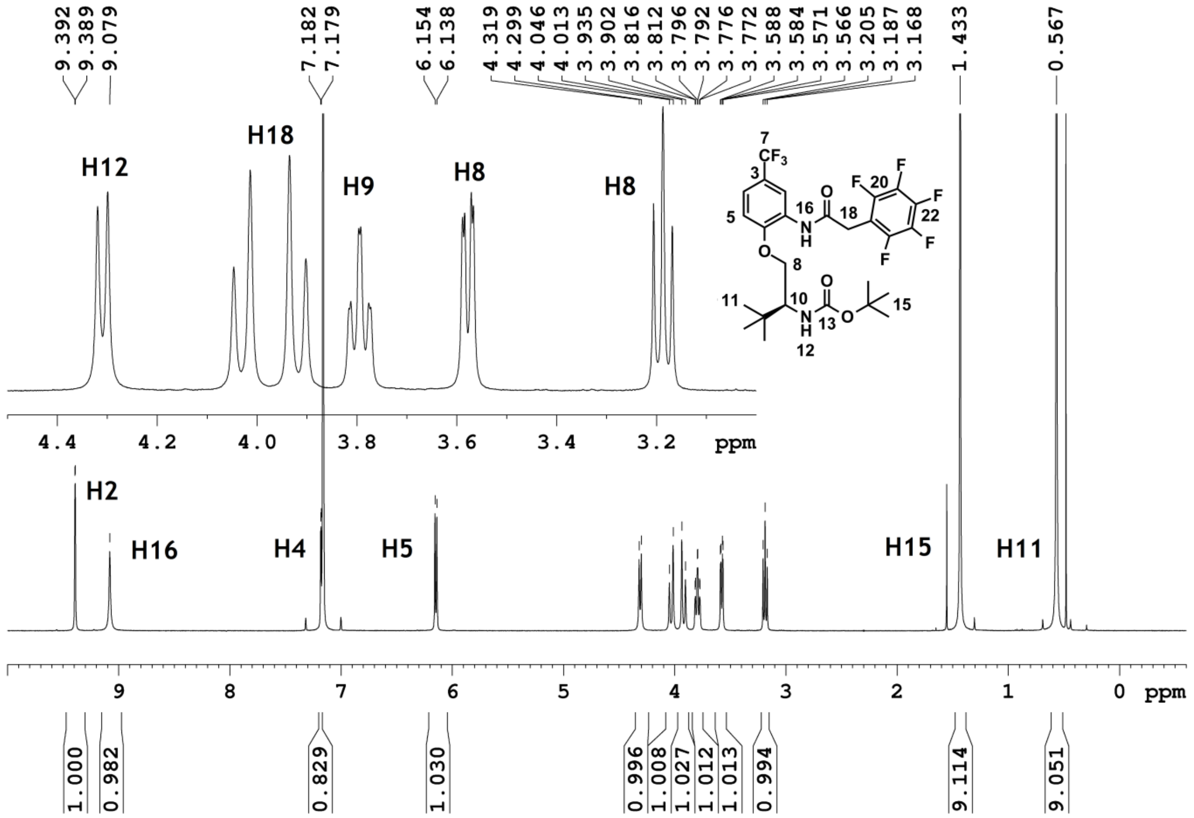

^{1}H spectrum of **12** (23˚C, C_6D_6, 500 MHz).

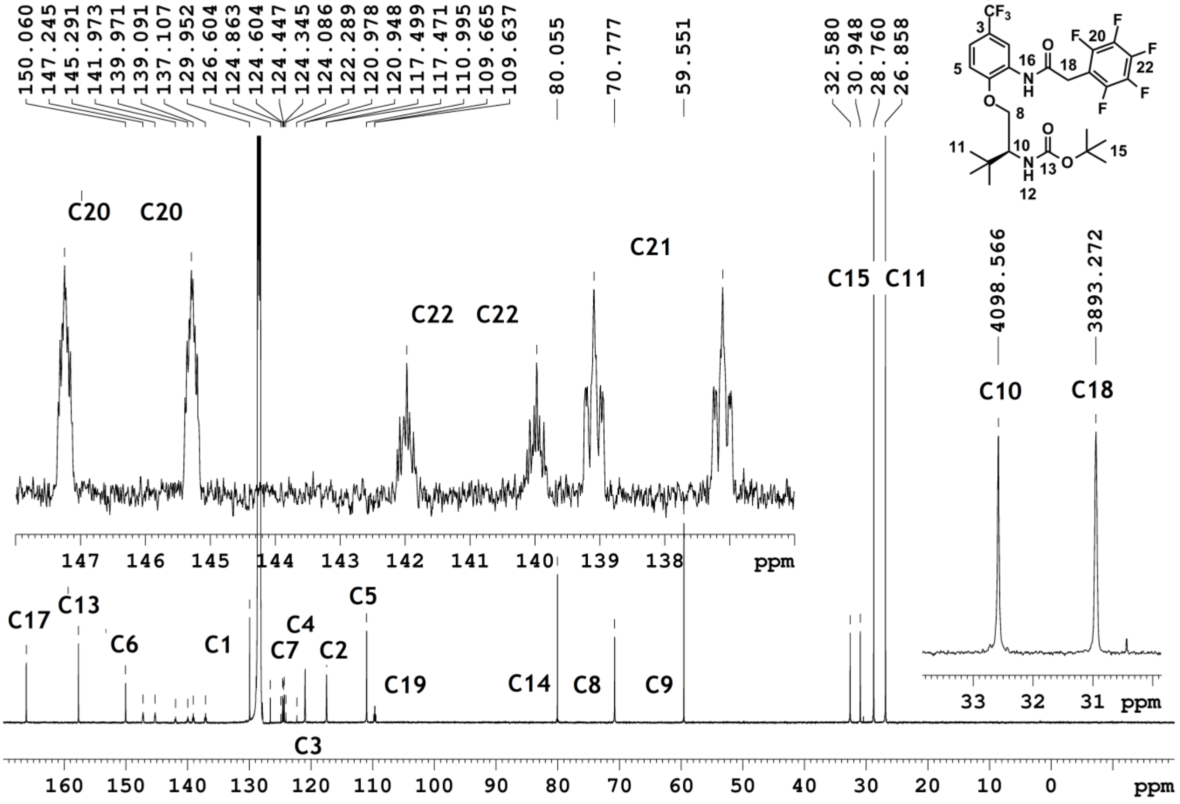

^{13}C spectrum of **12** (23˚C, C_6D_6, 125 MHz).

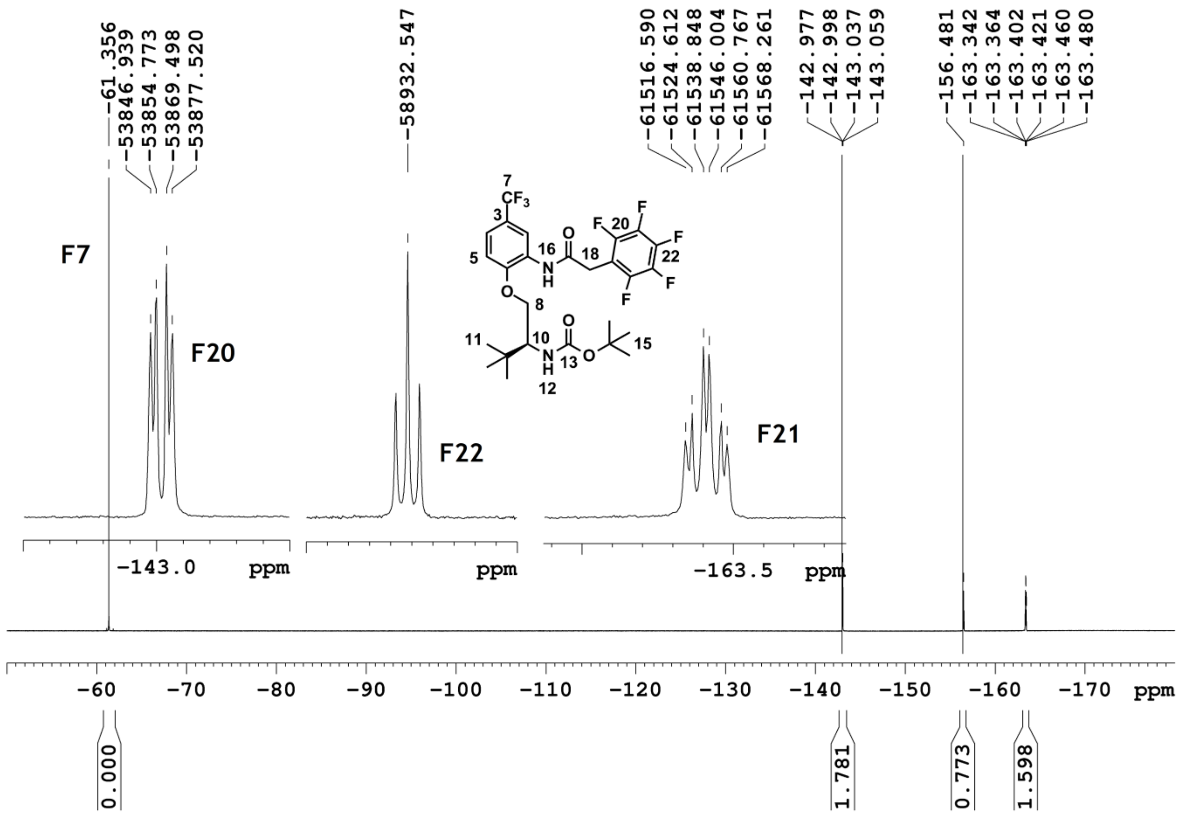

^{19}F spectrum of **12** (23˚C, C_6D_6, 376 MHz).

Mass Spectrum SmartFormula Report

Analysis Info

Analysis Name	\\Utof\data\Mar 14\ESI45138_11_01_17281.d	Acquisition Date	06/03/2014 08:17:55
Method	2.5min_cal_sample_pos_naf_05-08-13.m	Operator	Mass Spec
Sample Name	ESI45138	Instrument / Ser#	micrOTOF 92
Comment			

Acquisition Parameter

Source Type	ESI	Ion Polarity	Positive	Set Nebulizer	2.0 Bar
Focus	Not active			Set Dry Heater	180 °C
Scan Begin	100 m/z	Set Capillary	4500 V	Set Dry Gas	10.0 l/min
Scan End	1000 m/z	Set End Plate Offset	-500 V	Set Divert Valve	Source

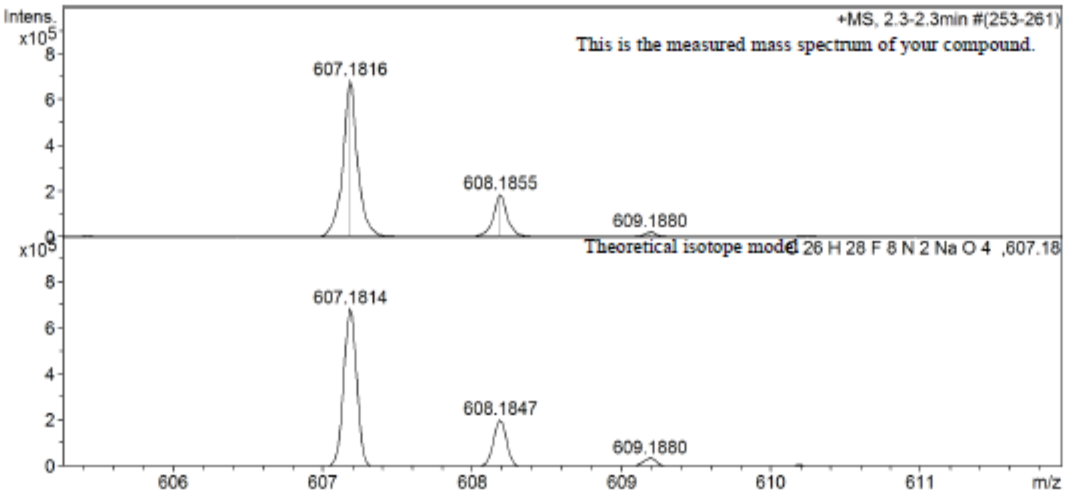

Meas. m/z	#	Formula	m/z	err [ppm]	Mean err [ppm]	rdb	e⁻ Conf	mSigma
607.1816								
	1	C 26 H 28 F 8 N 2 Na O 4	607.1814	-0.4	-0.6	9.5	even	11.38

High resolution mass spectrum of **12** showing molecular ion plus sodium cation.

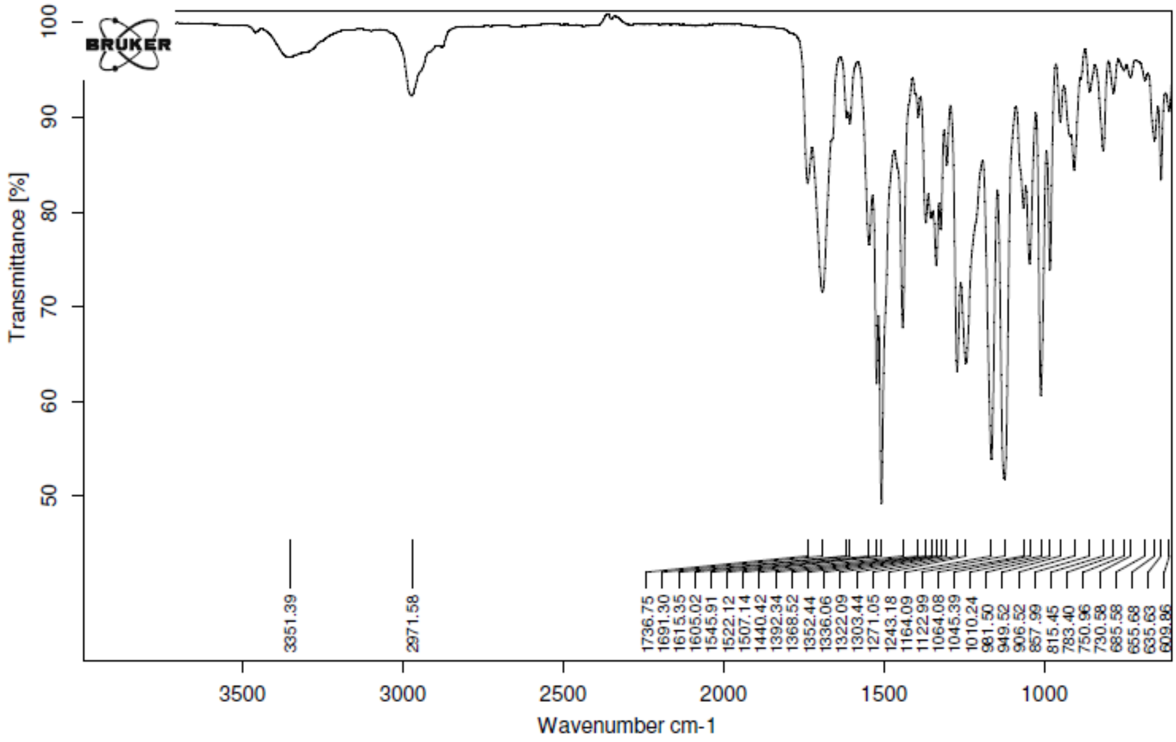

FT-IR of **12.**

(rac)-tert-butyl 2-((2-((tert-butoxycarbonyl)amino)-3,3-dimethylbutoxy)-5-(trifluoromethyl)phenyl)carbamoyl)pyrrolidine-1-carboxylate [13]

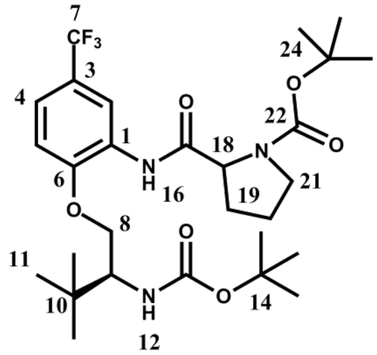

δH (700 MHz, C_6D_6, 23˚C): 9.91 (1H, s, H2), 9.58-9.20 (1H, bs, H16), 8.72 (1H, s, H16'), 7.13 (1H, d *J* 8 Hz, H4), 6.30 (1H, m, H5), 5.45 (1H, bs, H12), 5.33 (1H, bs, H12), 4.51 (1H, bs, H18), 3.99 (1H, bs, H9), 3.65 (2H, m, H8, H8), 3.22 (1H, bs, H21), 3.01 (1H, bs, H21), 2.29 (1H, bs, H19), 1.93 (1H, bs, H19), 1.47 (9H, s, H15), 1.41 (9H, s, H15), 0.84 (9H, s, H11), 0.83 (2H, bs, H20).

δC (175 MHz, C_6D_6, 23˚C): 170.37 (C17), 156.82 (C22), 150.46 (C20), 129.80 (C1), 125.54 (q, $^{1}J_{13C\text{-}19F}$ 27 Hz, C7), 124.60 (q, $^{2}J_{13C\text{-}19F}$ 34 Hz, C3), 121.01 (q, $^{3}J_{13C\text{-}19F}$ 3 Hz, C4), 117.92 (s, C2), 111.35 (C5), 80.36 (C14), 79.50 (C23), 70.28 (C8), 61.02 (C18), 58.28 (C9), 47.73 (C21), 34.57 (C10), 28.94 (C15), 28.77 (C25), 27.30 (C11).

δF (376 MHz, C_6D_6, 23˚C): -61.37.

HRMS: (ES-): found 596.2916; Formula $C_{28}H_{42}F_3N_3O_6Na$, [M + Na] requires 596.2918.

ν_{max} (neat, cm^{-1}): 3350.54, 2973.92, 2878.35, 2281.53, 1692.97, 1603.80, 1540.81, 1490.32, 1392.71, 1367.09, 1339.83, 1324.81, 1271.43, 1248.63, 1212.72, 1162.98, 1121.61, 1089.94, 1063.20, 1012.20, 930.60, 901.00, 885.88, 860.68, 813.40, 774.62, 736.27, 655.55, 635.67.

MP: 75-77°C (racemic mix of diastereomers)

X-ray crystallographic data provided for the (S)-enantiomer.

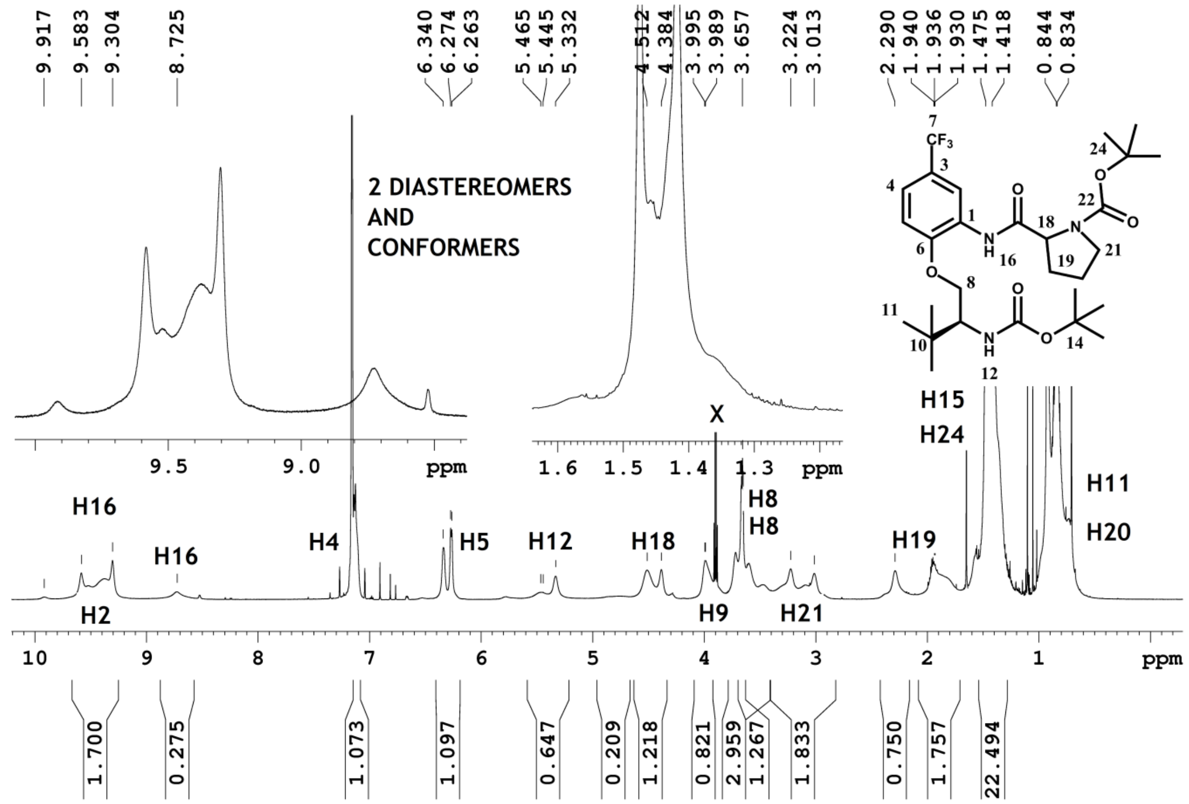

^{1}H NMR spectrum of **13** (700 MHz, 23˚C, C_6D_6).

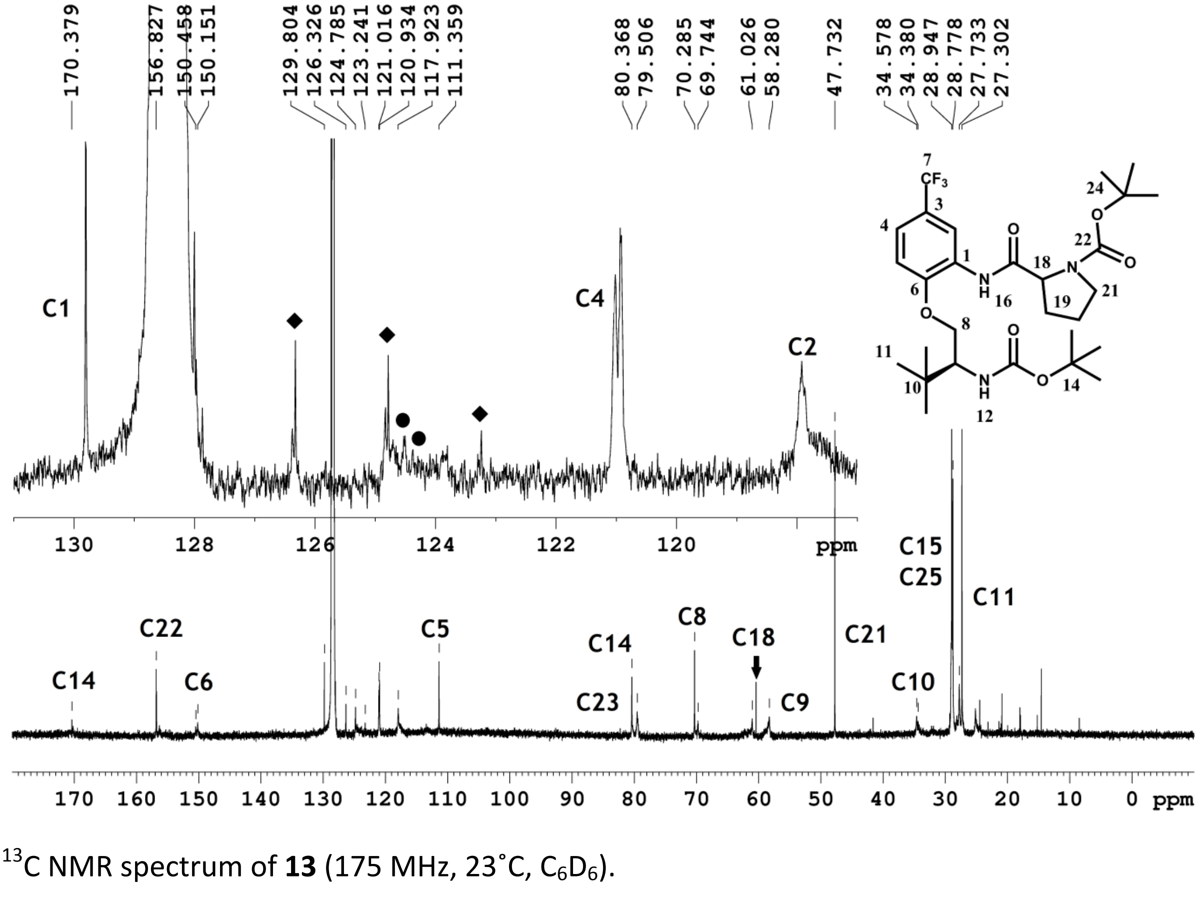

^{13}C NMR spectrum of **13** (175 MHz, 23˚C, C_6D_6).

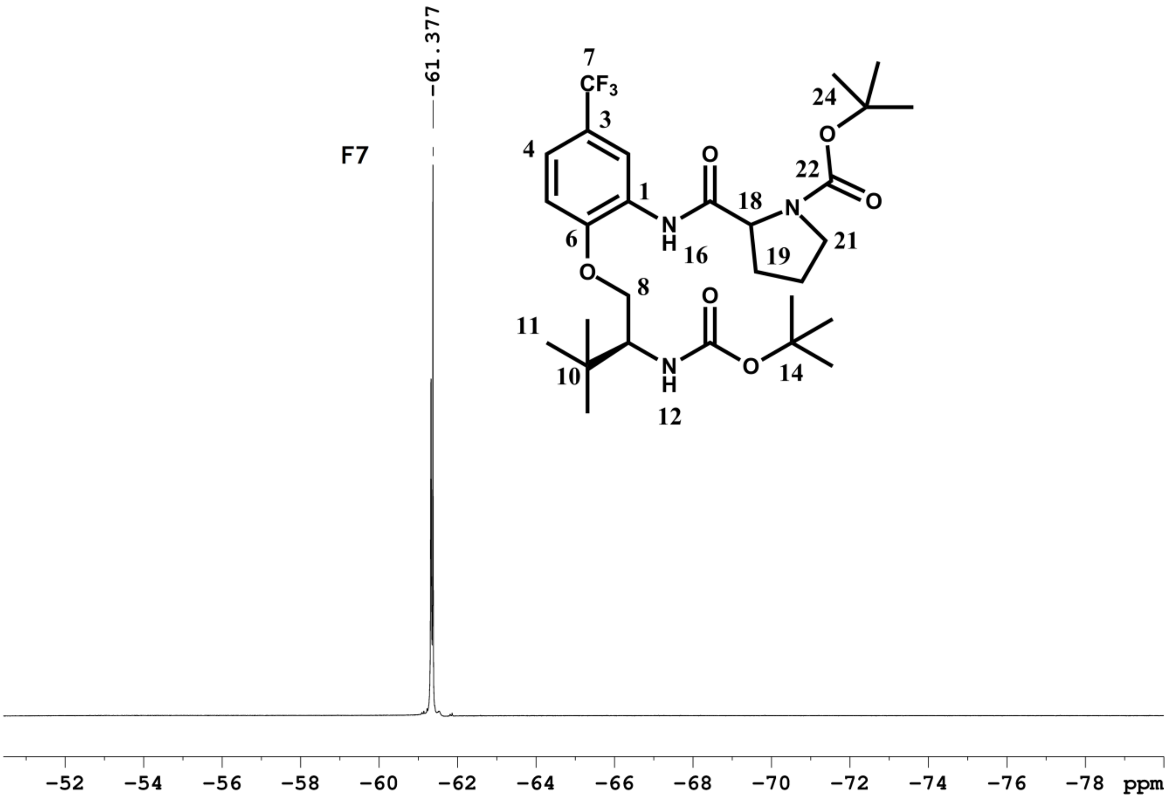

^{19}F NMR spectrum of **13** (376 MHz, 23C, C_6D_6).

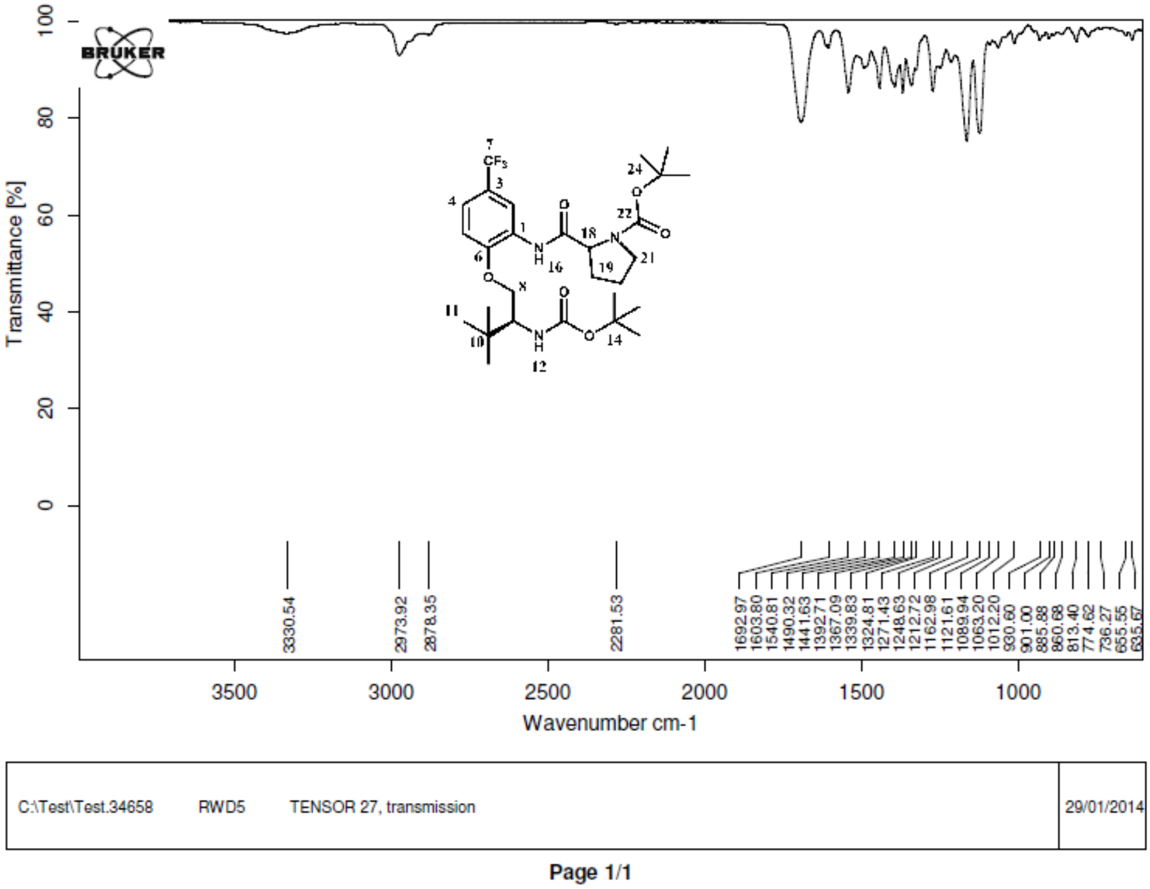

FT-IR of **13**.

Mass Spectrum SmartFormula Report

Analysis Info

Analysis Name	\\Utof\data\Jan 14\ESI44523_8_01_16073.d	Acquisition Date	31/01/2014 08:21:05
Method	2.5min_cal_sample_pos_naf_05-08-13.m	Operator	Mass Spec
Sample Name	ESI44523	Instrument / Ser#	micrOTOF 92
Comment			

Acquisition Parameter

Source Type	ESI	Ion Polarity	Positive	Set Nebulizer	2.0 Bar
Focus	Not active			Set Dry Heater	180 °C
Scan Begin	100 m/z	Set Capillary	4500 V	Set Dry Gas	10.0 l/min
Scan End	1000 m/z	Set End Plate Offset	-500 V	Set Divert Valve	Source

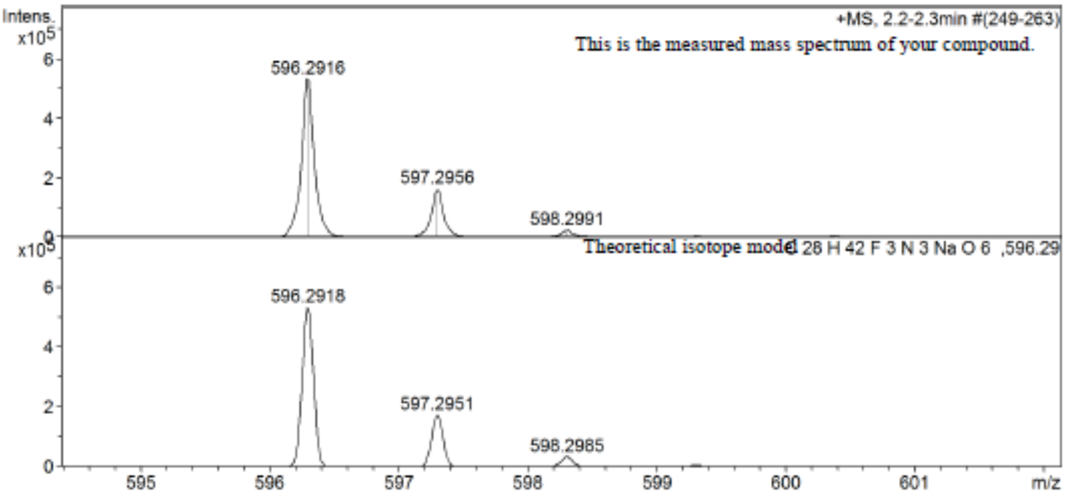

Meas. m/z	#	Formula	m/z	err [ppm]	Mean err [ppm]	rdb	e⁻ Conf	mSigma
596.2916								
	1	C 28 H 42 F 3 N 3 Na O 6	596.2918	0.3	-0.1	7.5	even	8.54

High Resolution MS of **13**.

(S)-tert-butyl (1-(2-acetamido-4-(trifluoromethyl)phenoxy)-3,3-dimethylbutan-2-yl)-N-tertbutyl carbamate glycine carbamate [14]

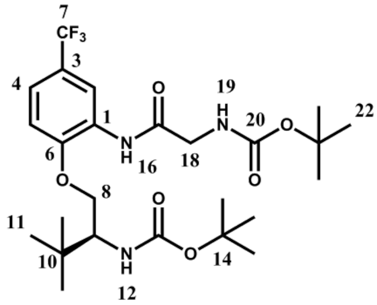

Prepared according to the representative procedure from racemic tert-butyl (1-(2-amino-4-(trifluoromethyl)phenoxy)-3,3-dimethylbutan-2-yl)carbamate on a 0.20 mmol scale. Chromatography (5:1 PET:EtOAc) to yield a white solid (45 mg, 40 %).

δH (500 MHz, C_6D_6, 23˚C): 9.35 (1H, s, H2), 8.96 (1H, s, H16), 7.24 (1H, dd, J_1 8 Hz, J_2 2 Hz, H4), 6.31 (1H, d, J 8 Hz, H5), 5.79 (1H, s, H19), 4.71 (1H, m, H12), 4.49 (1H, m, H9), 4.07 (1H, m, H18), 3.98 (1H, m, H9), 3.78 (1H, d, J 7 Hz, H8), 3.41 (1H, m, H8), 1.55 (9H, s, H15), 1.50 (9H, s, H22), 0.86 (9H, s, H11).

δC (125 MHz, C_6D_6, 23˚C): 168.71 (C17), 157.38 (C13), 156.59 (C20), 150.16 (C6) 129.57 (C1), 125.58 (q, $^1J_{13C\text{-}19F}$ 271 Hz, C7), 124.06 (q, $^2J_{13C\text{-}19F}$ 33 Hz, C3), 120.93 (q, $^3J_{13C\text{-}19F}$ 3 Hz, C4), 117.39 (s, C2), 111.12 (C5), 80.06 (C14), 79.66 (C21), 70.41 (C8), 59.13 (C9), 45.74 (C18), 33.22 (C10), 28.84 (C15), 28.79 (C22), 27.04.

δF (376 MHz, C_6D_6, 23˚C): -61.66.

HRMS: (ES-): found 556.2605; Formula $C_{25}H_{38}F_3N_2O_6Na$, [M + Na] requires 556.2605.

ν_{max} (neat, cm^{-1}): 3330.38, 2973.54, 1685.69, 1605.09, 1541.68, 1442.76, 1376.11, 1343.01, 1271.85, 1163.32, 1120.89, 1064.52, 1011.81, 901.48, 861.63, 812.79, 636.29.

MP: 172-73˚C.

$[\alpha]_D^{25.0}$ +16° (c = 0.01, $CHCl_3$).

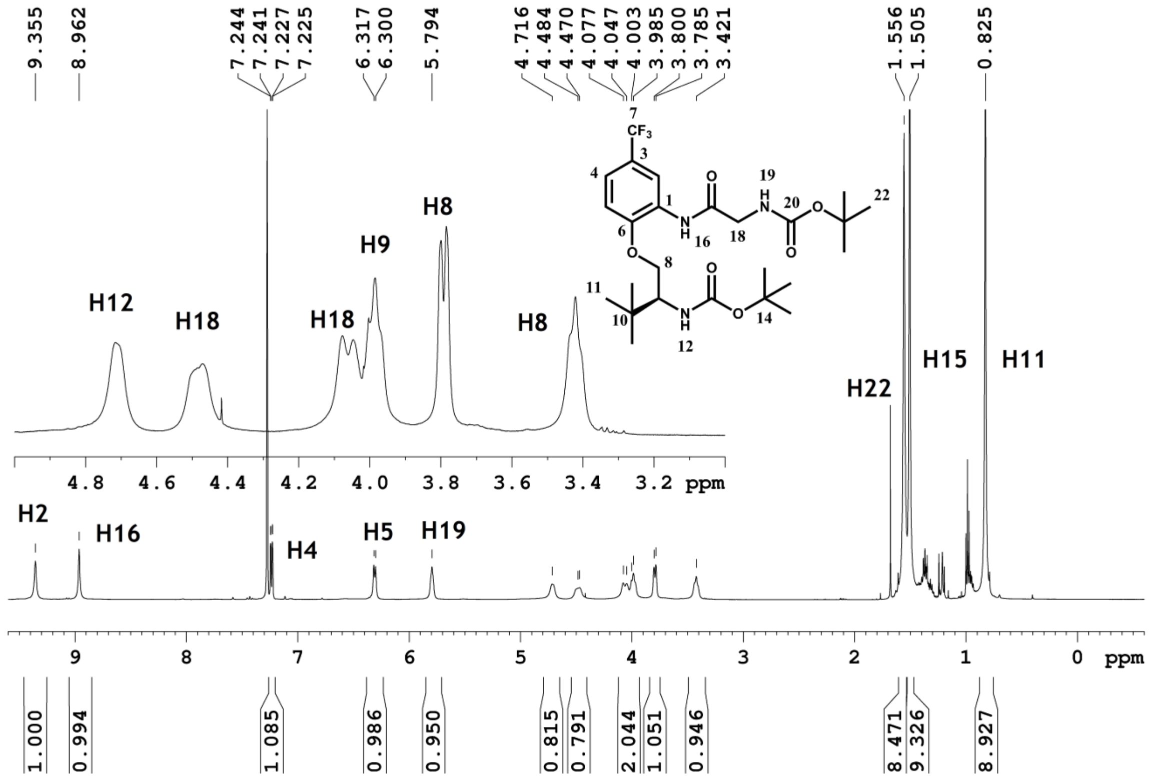

^{1}H NMR spectrum of **14** (23˚C, C_6D_6, 500 MHz).

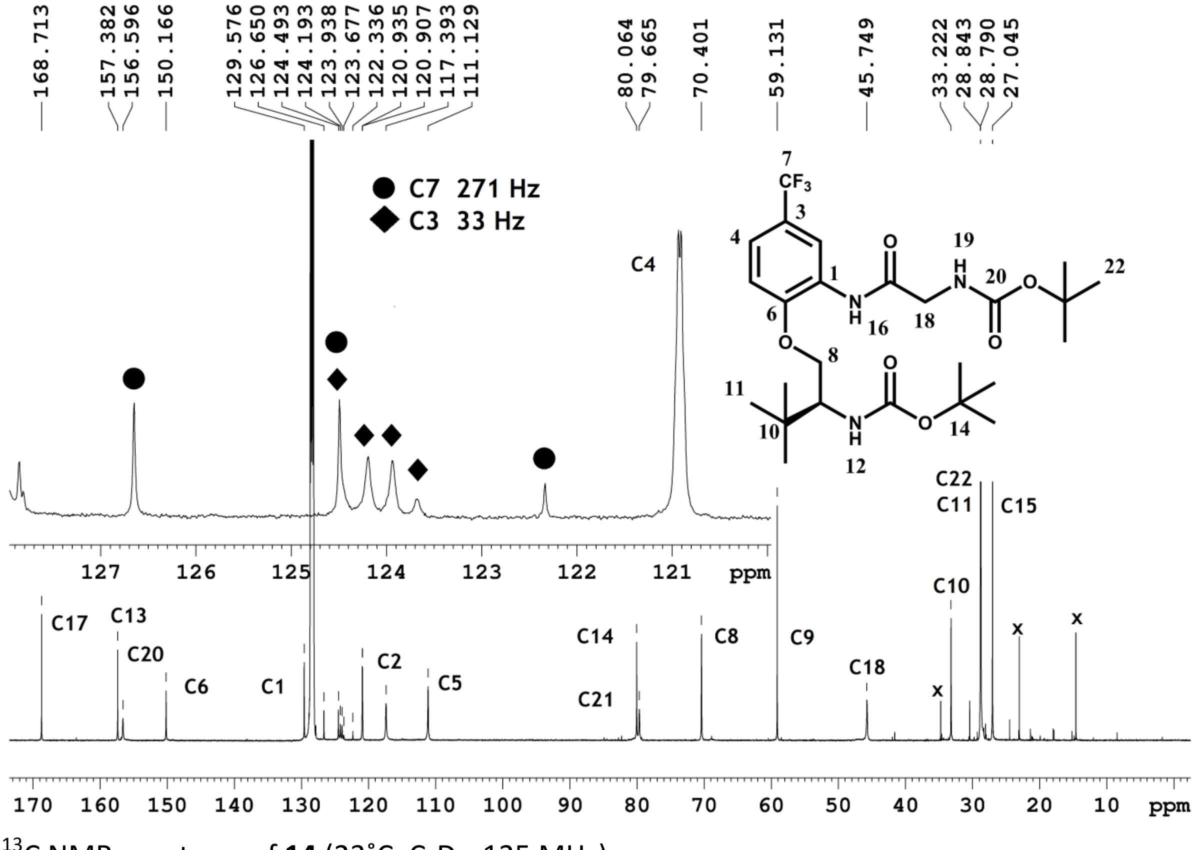

^{13}C NMR spectrum of **14** (23˚C, C_6D_6, 125 MHz).

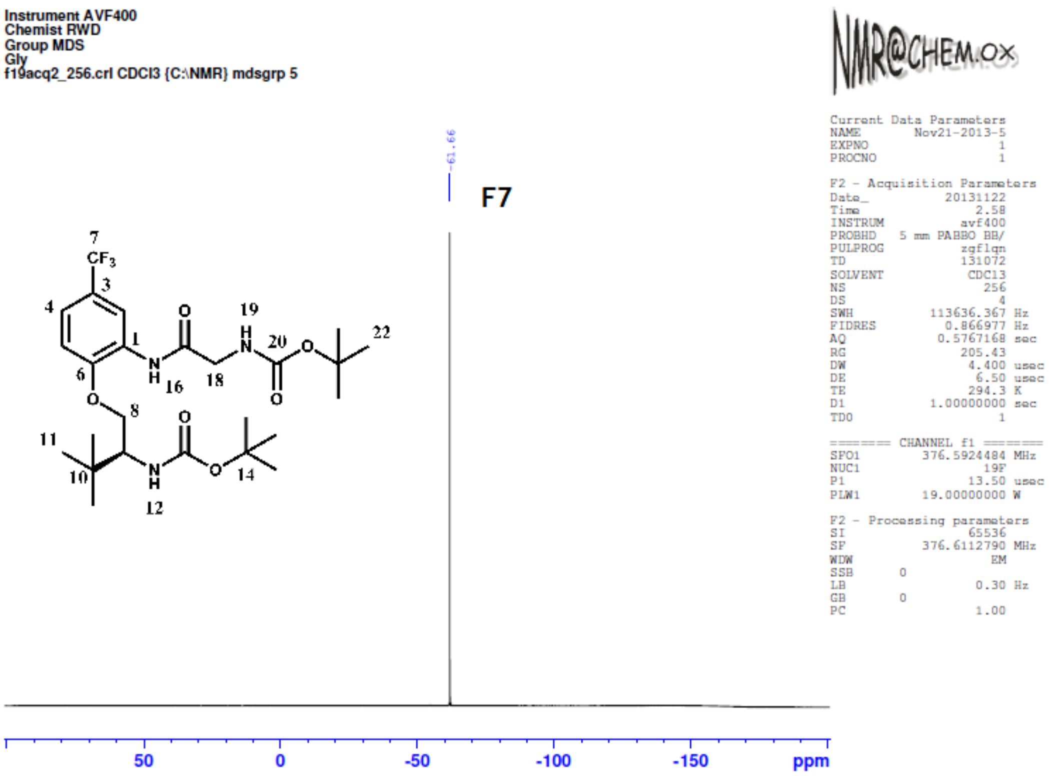

^{19}F NMR spectrum of **14** (23˚C, C_6D_6, 376 MHz).

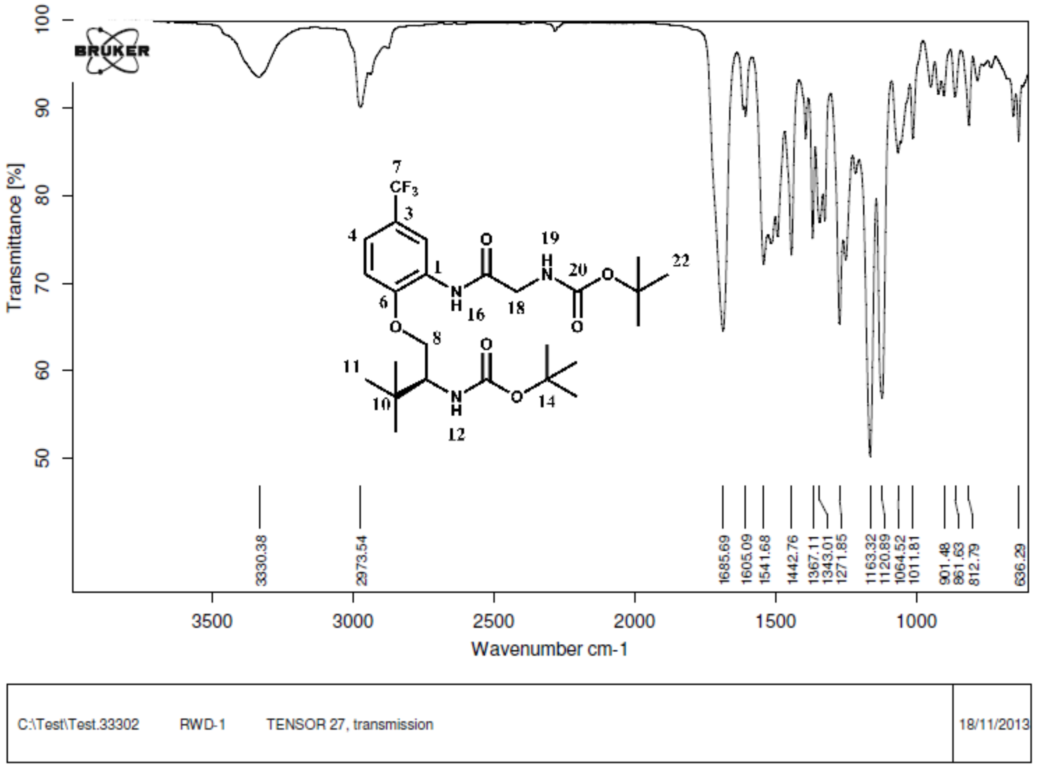

IR spectrum of **14**.

Mass Spectrum SmartFormula Report

Analysis Info

Analysis Name	\\Utof\data\Nov 13\ESI43506_11_01_14160.d	Acquisition Date	14/11/2013 08:21:44
Method	2.5min_cal_sample_pos_naf_05-08-13.m	Operator	Mass Spec
Sample Name	ESI43506	Instrument / Ser#	micrOTOF 92
Comment			

Acquisition Parameter

Source Type	ESI	Ion Polarity	Positive	Set Nebulizer	2.0 Bar
Focus	Not active			Set Dry Heater	180 °C
Scan Begin	100 m/z	Set Capillary	4500 V	Set Dry Gas	10.0 l/min
Scan End	1000 m/z	Set End Plate Offset	-500 V	Set Divert Valve	Source

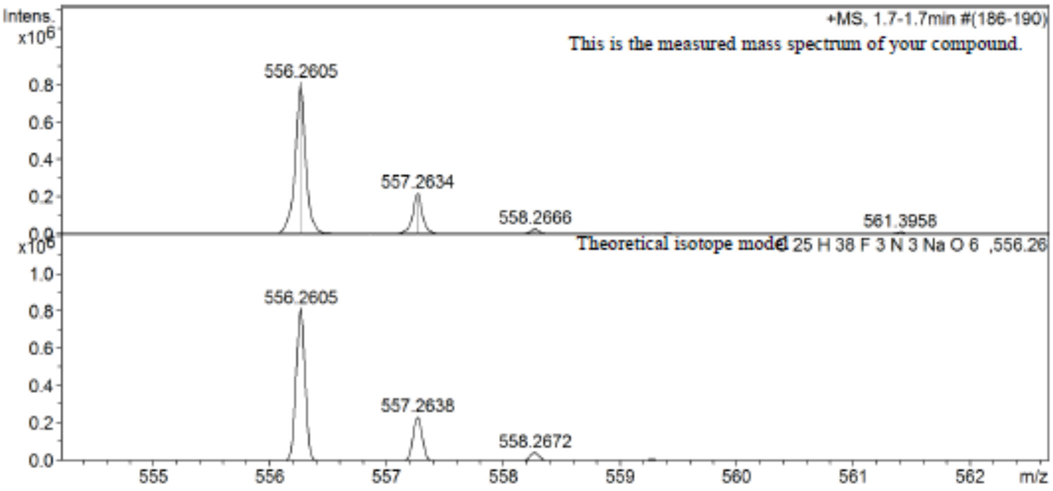

Meas. m/z	#	Formula	m/z	err [ppm]	Mean err [ppm]	rdb	e⁻ Conf	mSigma
556.2605								
	1	C 25 H 38 F 3 N 3 Na O 6	556.2605	0.1	0.2	6.5	even	11.05

High resolution mass spectrum of **14**.

(S)-tert-butyl (1-(2-(9H-fluorene-9-carboxamido)-4-(trifluoromethyl)phenoxy)-3,3-dimethylbutan-2-yl)carbamate [15]

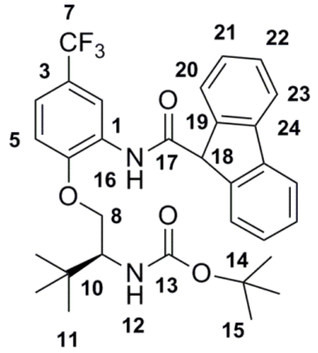

Prepared according to the representative procedure from (S)-tert-butyl (1-(2-amino-4-(trifluoromethyl)phenoxy)-3,3-dimethylbutan-2-yl)carbamate (75 mg, 0.196 mmol), EDCI.HCl (149 mg, 0.75 mmol), fluorenylcarboxylic acid (163 mg, 1.56 mmol), N,N-dimethylaminopyridine (7 mg, 0.05 mmol), pyridine (1 mL) and DCM (10mL). Chromatography (silica gel, petroleum ether : ethyl acetate 8:1), followed by recrystallization in EtOAc/n-pentane (1:10), 35 mg, 32%.

δH (500 MHz, C_6D_6, 23˚C): 9.41 (1H, s, H2), 7.81 (1H, m, H20), 7.78 (1H, s, H16), 7.55 (2H, m, *J* 7 Hz, H20, H23), 7.52 (1H, m, H23), 7.34 (1H, t, *J* 7 Hz, H21), 7.29 (1H, t, *J* 7 Hz, H21), 7.18 (2H, m, H22), 7.05 (1H, dd, J_1 8 Hz, J_2 1 Hz, H4), 6.08 (1H, d, *J* 8 Hz, H5), 4.88 (1H, s, H18), 4.28 (1H, d, J 10 Hz, H12), 3.57 (1H, m, H9), 3.31 (2H, m, H8), 1.45 (9H, s, H15), 0.44 (9H, s, H11).

δC (125 MHz, C_6D_6, 23˚C): 168.67 (C17), 156.14 (C13), 149.67 (C6), 143.01 (C19), 142.25 (C24), 141.95 (C19), 141.66 (C24), 129.23 (C22), 129.05 (C23), 128.97 (C22), 128.79 (C23), 128.69 (C1), 126.39 (C20), 126.12 (C20), 125.53 (C7, q, $^1J_{13C\text{-}19F}$ = 270 Hz), 124.02 (C3, q, $^2J_{13C\text{-}19F}$ = 33 Hz), 121.31 (C21), 121.18 (C4, d, $^3J_{13C\text{-}19F}$ = 4 Hz), 120.80 (C21), 117.52 (C2, q, $^3J_{13C\text{-}19F}$ = 4 Hz), 110.73 (C5), 79.68 (C14), 69.08 (C8), 157.84 (C9), 157.75 (C18), 34.20 (C10), 28.84 (C15), 27.09 (C11).

δF (236 MHz, C_6D_6, 23˚C): -61.64 (F7).

HRMS: found (591.2430); $C_{32}H_{35}F_3N_2O_4Na$, requires 591.2441.

ν_{max} (neat, cm^{-1}): 3324.06, 3250.56, 3068.97, 2965.74, 1680.79, 1670.74, 1613.10, 1532.62, 1487.36, 1450.15, 1435.55, 1401.98, 1388.87, 1363.88, 1334.32, 1309.37, 1273.43, 1241.11, 1205.63, 1158.48, 1107.62, 1065.42, 1030.31, 1006.42, 983.77, 952.47, 925.21, 899.74, 877.89, 861.58, 821.98, 788.19, 746.60, 733.93, 706.95, 695.60, 629.21, 620.91.

MP: 140 C.

$[\alpha]_D^{25.0}$ +41.0 (c = 0.01, $CHCl_3$).

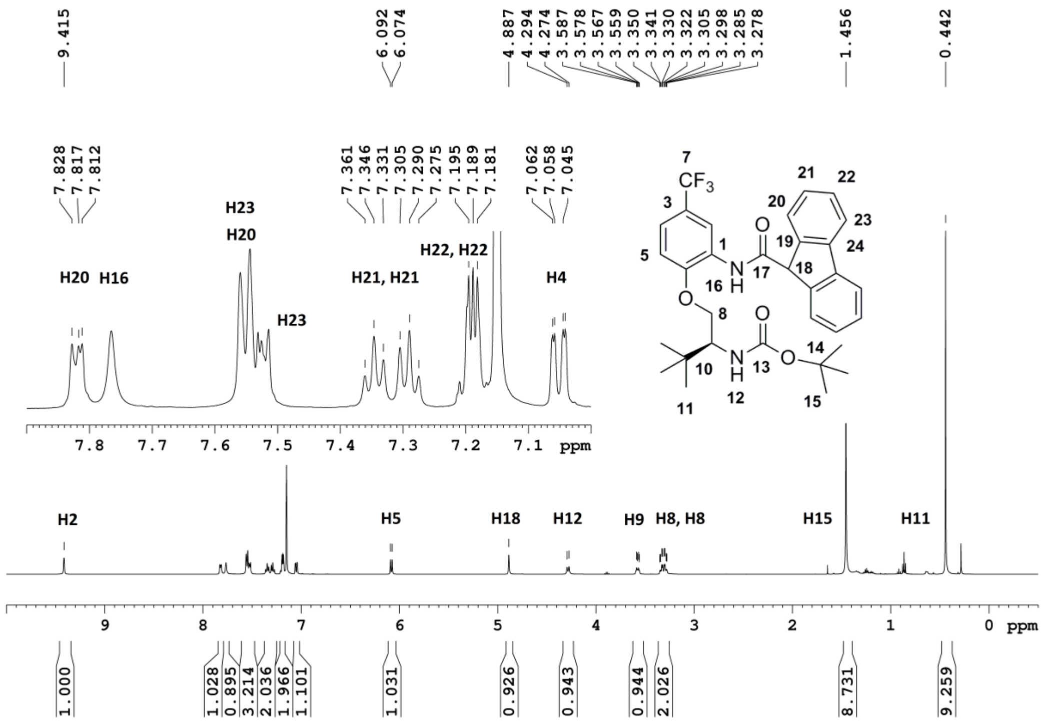

^{1}H NMR spectrum of **15** (23˚C, C_6D_6, 500 MHz).

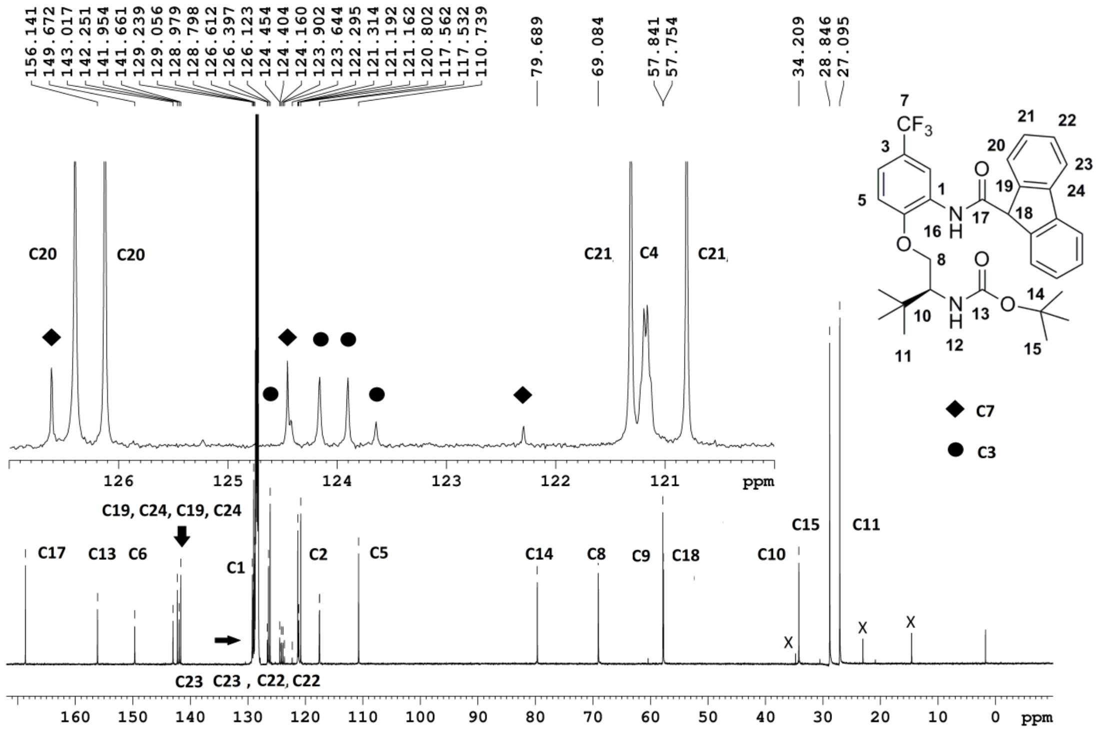

^{13}C NMR spectrum of **15** (23˚C, C_6D_6, 125 MHz).

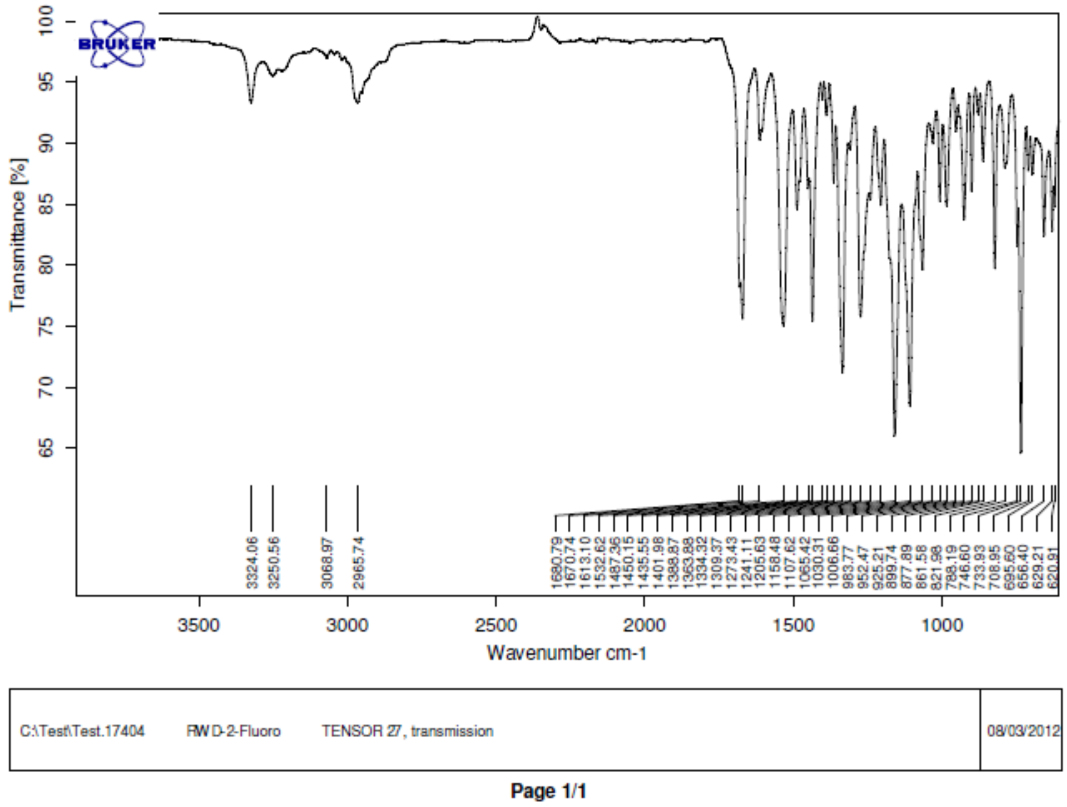

FT-IR of diffraction quality crystals of **15**.

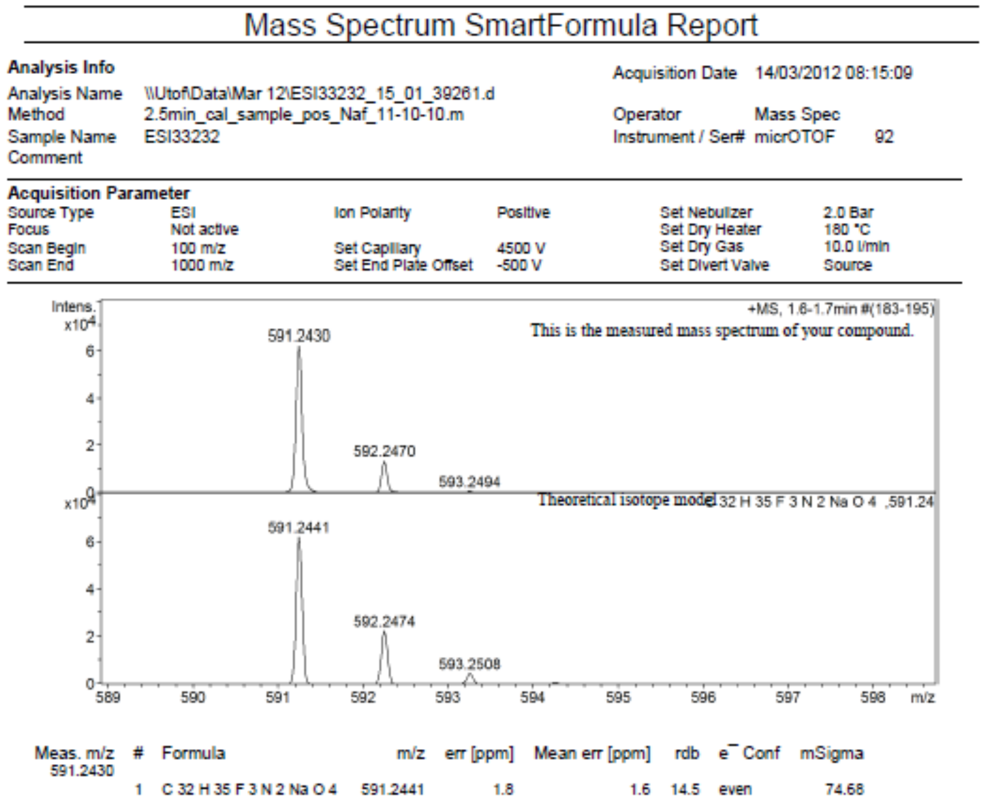

High-Resolution mass spectrum of **15**.

(S)-N-(2-(2-(3-(3,5-bis(trifluoromethyl)phenyl)ureido)-3,3-dimethylbutoxy)-5-(trifluoromethyl)phenyl)-3,3,3-trifluoro-2-(trifluoromethyl)propanamide [16]

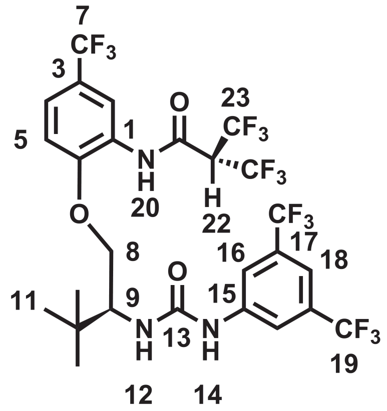

TFA (5mL) was added dropwise to a stirring solution of **8** (100 mg, 0.18 mmol) in DCM (5mL), at 0˚C and the reaction stirred for 1 hour. The mixture was concentrated in vacuo, suspended in Et_2O and 0.5mL HCl (2.0M in Et_2O) was added. The resultant white solid was filtered (*ca*. 75mg), DCM was added (50mL) and $NaHCO_3$ (50 mL, aq., saturated) was added. The organic phase was extracted, dried ($MgSO_4$) and concentrated to yield free amine as a white solid which was used without further purification. Crude amine and 3,5-Bis (trifluoromethyl) phenyl isocyanate (1.0 mL) were was stirred for 18h, and concentrated in vacuo. The resulting solid was purified by column chromatography (4:1 PET:EtOAc) to yield 15 mg (0.021 mmol, 11 %) of the title compound. The title compound is insoluble in both $CDCl_3$ and C_6D_6.

δH (500 MHz, C_6D_6, 23˚C, very low solubility): 9.15 (1H, d, *J* 1 Hz, H14), 9.06 (1H, s, H2), 7.80 (1H, bs, H18), 7.50 (3H, m, H16, H4), 6.12 (1H, d, *J* 8 Hz, H5), 5.78 (1H, bs, H12), 5.31 (1H, s, H14), 4.65 (1H, septet, *J* 8 Hz, H22), 3.96 (2H, m, H8, H9), 3.53 (1H, dd, J_1 9 Hz, J_2 2 Hz, H8), 1.41 (9H, s, H11).

δH (500 MHz, DMSO-d_6, 23˚C): 9.98 (1H, s, H20), 9.12 (1H, s, H14), 8.25 (1H, s, H2), 8.00 (2H, s, H16), 7.58 (2H, m, H4, H18), 7.44 (1H, d, *J* 9 Hz, H5), 6.68 (1H, d, *J* 8 Hz, H12), 5.30 (1H, sept, *J* 8 Hz, H22), 4.44 (1H, dd, J1 10 Hz, J_2 4 Hz, H8), 4.18 (1H, apparent triplet, *J* 10 Hz, H8), 4.08 (1H, m, H9), 1.03 (9H, s, H11).

δC (125 MHz, DMSO-d_6, 23˚C): 156.77 (C21), 155.32 (C6), 151.60 (C13), 142.12 (C15), 130.62 (q, $^2J_{13C\text{-}19F}$ = 32 Hz, C17), 125.15 (q, C7), 124.58 (q, $^1J_{13C\text{-}19F}$ 273 Hz, C23), 123.7 (q, $^1J_{13C\text{-}19F}$ 263 Hz, C19), 121.40 (q, $^2J_{13C\text{-}19F}$ = 32 Hz, C3), 118.45 (m, C2), 118.09 (m, C18), 113.56 (C5), 70.02 (C8), 57.03 (C9), 52.95 (septet, $^2J_{13C\text{-}19F}$ = 31 Hz, C20), 34.13 (C10), 26.97 (C11).

δF {^{1}H} (470 MHz, C_6D_6, 23˚C): -62.03 (F7), -63.53 (F19), -64.57 (F23, q, $^4J_{19F\text{-}19F}$ 9 Hz), -64.33 (F23, q, $^4J_{19F\text{-}19F}$ 9 Hz).

HRMS: (ES+): found 710.1479; Formula $C_{26}H_{23}F_{15}N_3NO_3$, theoretical: 710.1494.

ν_{max} (neat, cm^{-1}): 3405.64, 3211.38, 3100.63, 2945.81, 2164.39, 2047.18, 1979.92, 1692.22, 1655.42, 1617.82, 1548.23, 1500.07, 1470.92, 1445.91, 1385.93, 1369.16, 1341.17, 1321.16, 1296.14, 1274.60, 1247.60, 1234.30, 1210.36, 1179.05, 1170.09, 1144.86, 1117.81, 1095.79,

1076.77, 1064.67, 1040.73, 1013.28, 960.99, 927.56, 918.12, 899.09, 887.45, 865.92, 849.89, 839.24, 819.11, 777.71, 745.47, 721.62, 700.74, 683.94, 657.00, 642.50, 628.45, 617.96.

MP: 182 C.

$[\alpha]_D^{25.0}$ +27.0° (c = 0.15, $CHCl_3$).

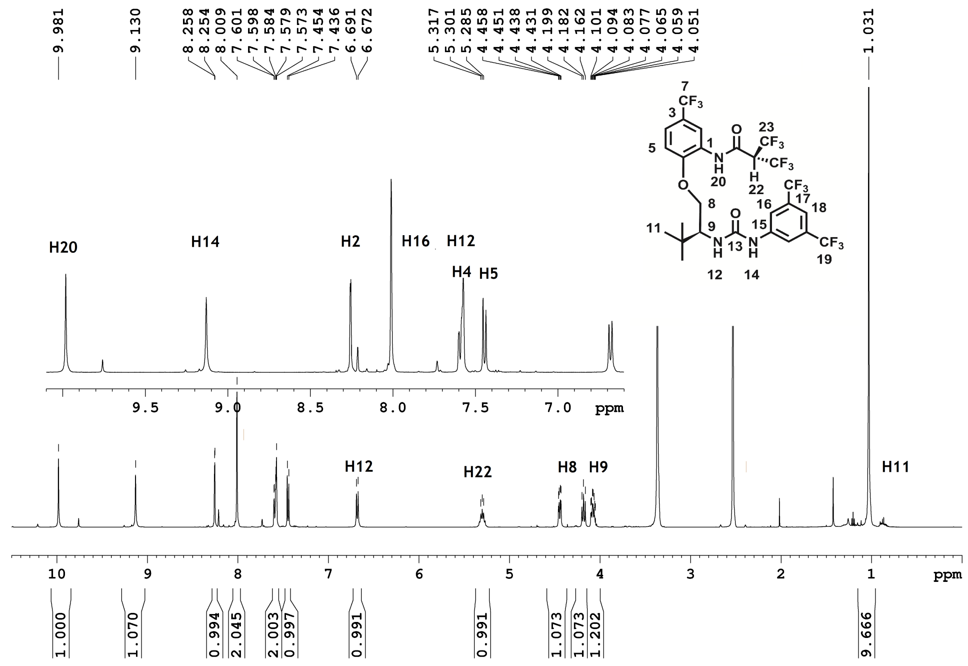

^{1}H NMR spectrum of **16** (23˚C, DMSO-d_6, 500 MHz).

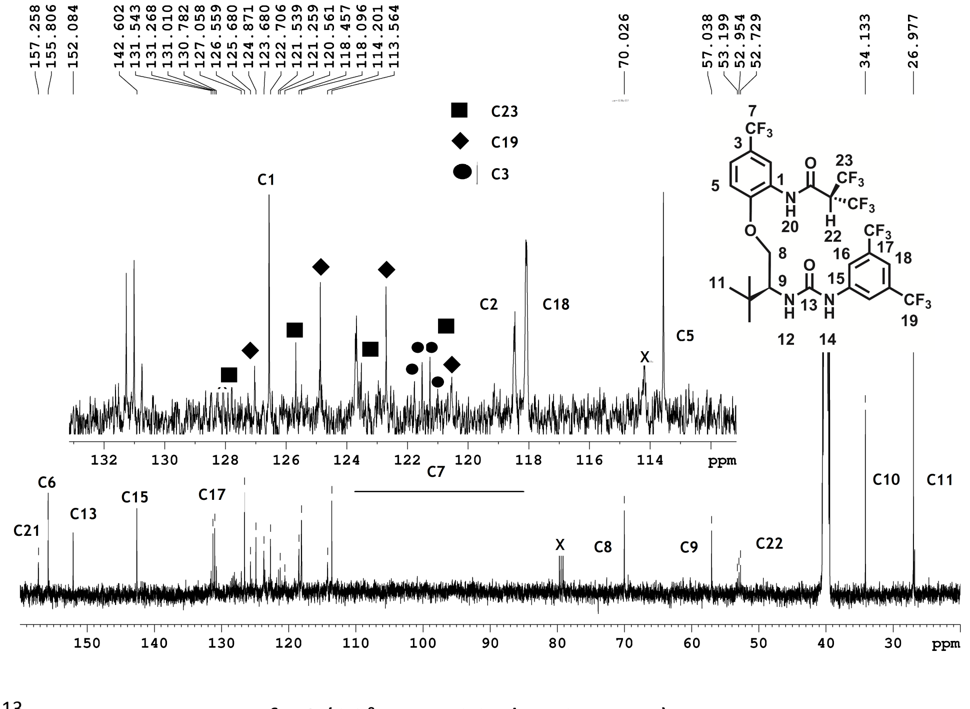

^{13}C NMR spectrum of **16** (23˚C, DMSO-d_6, 125 MHz).

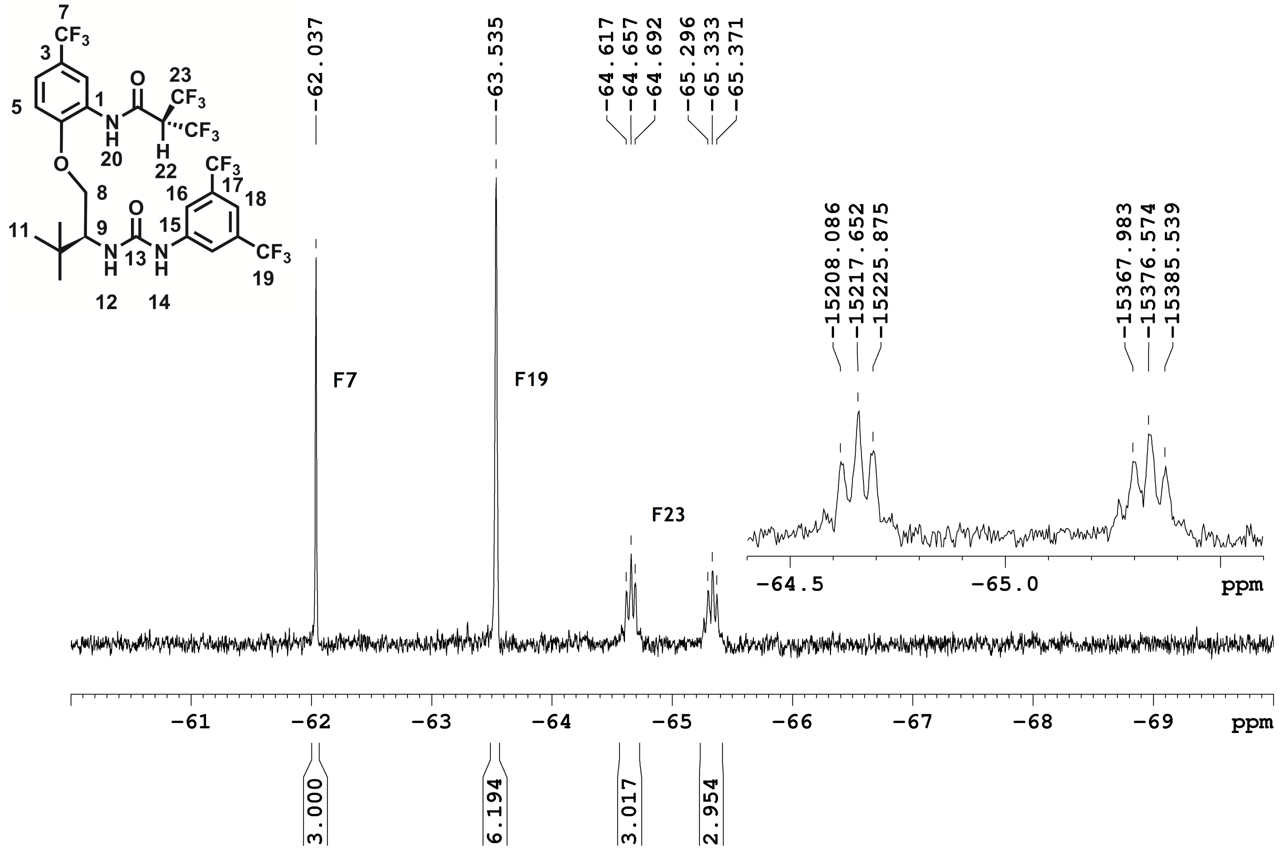

^{19}F NMR spectrum of **16** (23˚C, C_6D_6, 470 MHz).

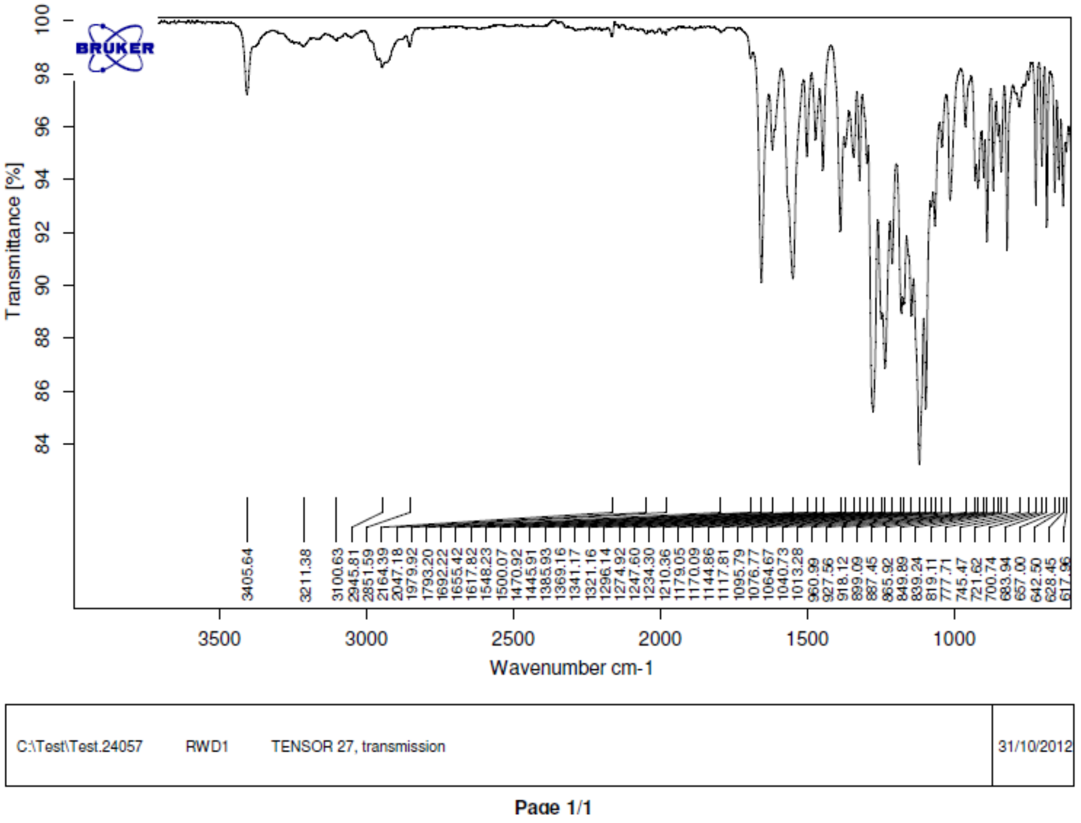

Solid-state FT-IR spectrum of **16**.

Mass Spectrum SmartFormula Report

Analysis Info

Analysis Name	\\Utof\data\Oct 12\ESI37288_13_01_48316.d	Acquisition Date	12/10/2012 08:25:32
Method	2.5min_cal_sample_pos_Naf_11-10-10.m	Operator	Mass Spec
Sample Name	ESI37288	Instrument / Ser#	micrOTOF 92
Comment			

Acquisition Parameter

Source Type	ESI	Ion Polarity	Positive	Set Nebulizer	2.0 Bar
Focus	Not active			Set Dry Heater	180 °C
Scan Begin	100 m/z	Set Capillary	4500 V	Set Dry Gas	10.0 l/min
Scan End	1000 m/z	Set End Plate Offset	-500 V	Set Divert Valve	Source

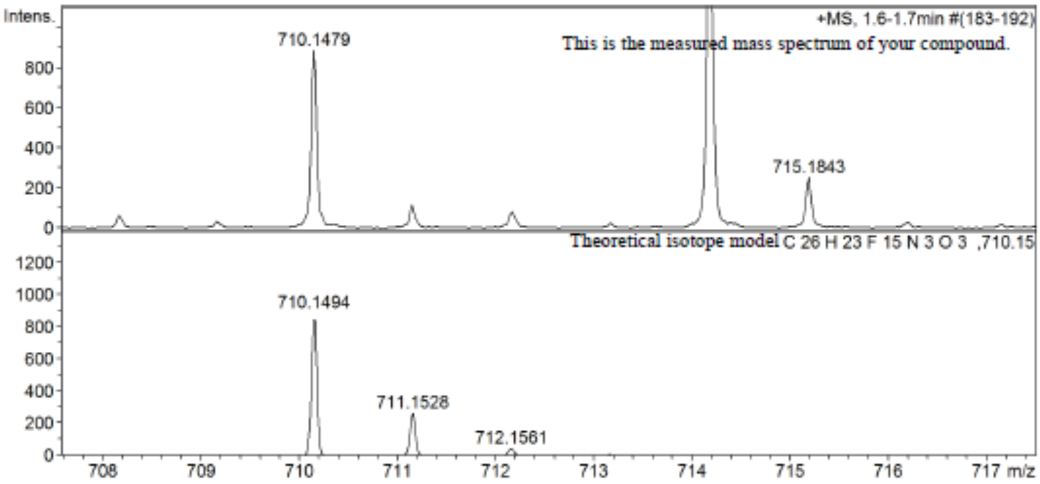

Meas. m/z	#	Formula	m/z	err [ppm]	Mean err [ppm]	rdb	e⁻ Conf	mSigma
710.1479								
	1	C 26 H 23 F 15 N 3 O 3	710.1494	2.2	2.2	9.5	even	170.95

High-Resolution Mass Spectrum of **16**.

(S)-N-(3,3-dimethyl-1-(2-(3,3,3-trifluoro-2-(trifluoromethyl)propanamido)-4-(trifluoromethyl)phenoxy)butan-2-yl)-9H-fluorene-9-carboxamide [17]

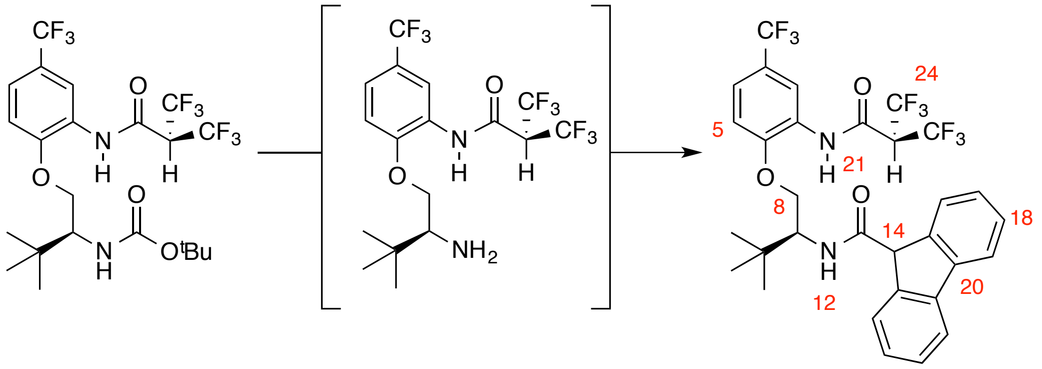

TFA (5mL) was added dropwise to a stirring solution of **8** (100 mg, 0.18 mmol) in DCM (5mL), at 0˚C and the reaction stirred for 1 hour. The mixture was concentrated in vacuo, suspended in Et_2O and 0.5mL HCl (2.0M in Et_2O) was added. The resultant white solid was filtered (*ca*. 75mg), DCM was added (50mL) and $NaHCO_3$ (50 mL, aq., saturated) was added. The organic phase was extracted, dried ($MgSO_4$) and concentrated to yield free amine as a white solid which was used without further purification. This crude amine was used according to the representative coupling procedure: 65 mg EDCI.HCl (0.18 mmol), 38 mg flourenylcarboxylic acid (0.18 mmol), 3 mg N,N-dimethylaminopyridine (0.02 mmol) and 0.5 mL pyridine. Chromatography (silica gel, petroleum ether : ethyl acetate 4:1), 20mg, 26% as a white solid.

δH (500 MHz, C_6D_6, 23˚C): 10.00 (1H s, H21), 9.47 (3H, d, *J* 2.0 Hz, H2), 7.61 (1H, dd, J_1 7 Hz, J_2 1 Hz, H16), 7.56 (1H, d, *J* 8 Hz, H19), 7.53 (1H, d, *J* 7 Hz, H19), 7.42 (1H, d, *J* 7 Hz, H16), 7.25 (1H, m, H17), 7.21 (1H, dd, J_1 8 Hz, J_2 2 Hz, H4), 7.15 (2H, m, H17, H18), 7.03 (1H, dt, J_1 8 Hz, J_2 1 Hz, H18), 6.06 (1H, d, J 8 Hz, H5), 5.87 (1H, sept, *J* 8 Hz, H23), 4.66 (1H, s, H14), 3.92 (1H, dt, J_1 10, J_2 3 Hz, H9), 3.47 (1H, dd, J_1 9 Hz, J_2 3 Hz, H8), 3.08 (1H, m, H8), 0.34 (9H, s, H11).

δC (125 MHz, C_6D_6, 23˚C): 171,96 (C13), 157.81 (C23), 150.26 (C6), 142.25 (C15), 142.19 (C15), 141.69 (C20), 141.66 (C20), 129.36 (C1), 129.26 (C17), 129.20 (C17), ca 128.39 (C18, C18), 125.28 (C7, q, $^1J_{13C\text{-}19F}$ 271 Hz), 125.70 (C16), 125.40 (C16), 124.66 (C3, q, $^2J_{13C\text{-}19F}$ 32 Hz), 123.06 (C24, q, $^1J_{13C\text{-}19F}$ 279 Hz), 122.03 (C4, q, $^3J_{13C\text{-}19F}$ 4 Hz), 121.13 (C19), 120.91 (C19), 117.63 (C2, $^3J_{13C\text{-}19F}$ 4 Hz), 111.21 (C5), 70.32 (C8), 58.74 (C9), 55.55 (C23, sept, $^2J_{13C\text{-}19F}$ 29 Hz), 32.08 (C10), 23.06 (C11).

δF (470 MHz, C_6D_6, 23˚C): -61.26 (F7), -63.54 (F24, quintet), -63.87 (F24, quintet).

ν_{max} (neat, cm^{-1}): 3376.40, 3258.40, 3067.13, 2961.88, 2872.91, 1700.97, 1654.15, 1620.76, 1561.91, 1522.57, 1502.75, 1468.34, 1447.33, 1357.73, 1346.15, 1322.30, 1290.36, 1275.26, 1239.78, 1218.12, 1203.60, 1160.35, 1146.20, 1121.22, 1095.55, 1072.81, 1044.61, 1022.00, 1008.52, 930.37, 914.94, 886.82, 865.36, 839.47, 817.56, 801.63, 743.64, 723.11, 695.68, 654.68, 633.47, 621.28

HRMS: (ES+Na): $C_{31}H_{27}F_9N_2NaO_3$, found (669.1770);, [M + Na] requires 669.1764.

MP: 140 C.

$[\alpha]_D^{25.0}$ +41.0 (c = 0.01, $CHCl_3$).

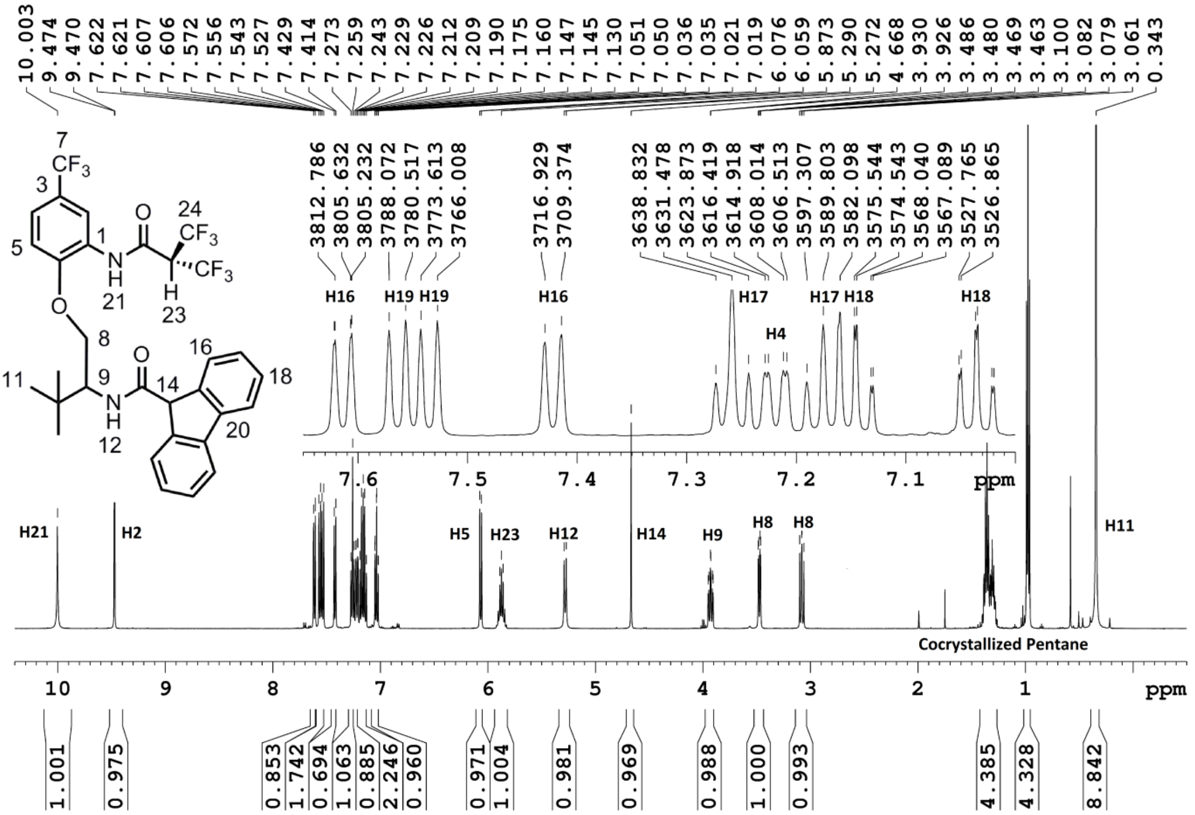

^{1}H NMR spectrum of **17** (23˚C, C_6D_6, 500 MHz).

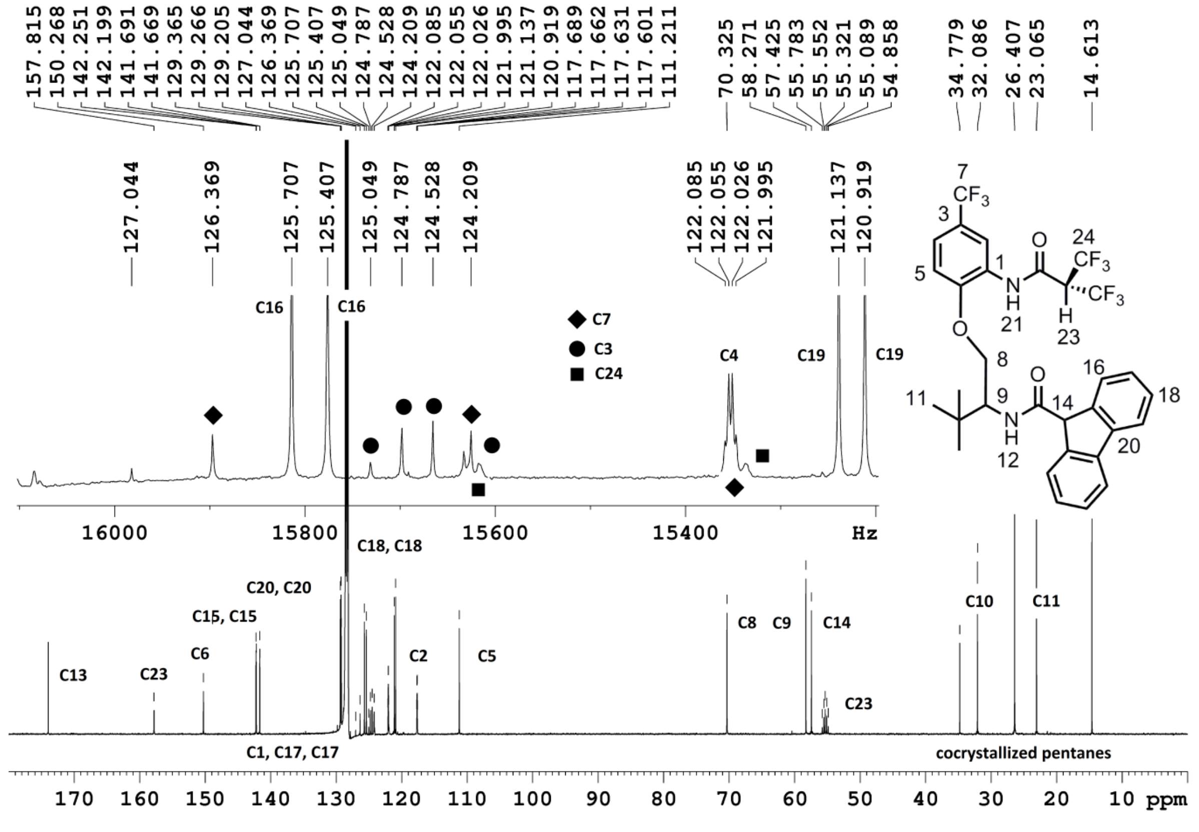

^{13}C NMR spectrum of **17** (23˚C, C_6D_6, 125 MHz).

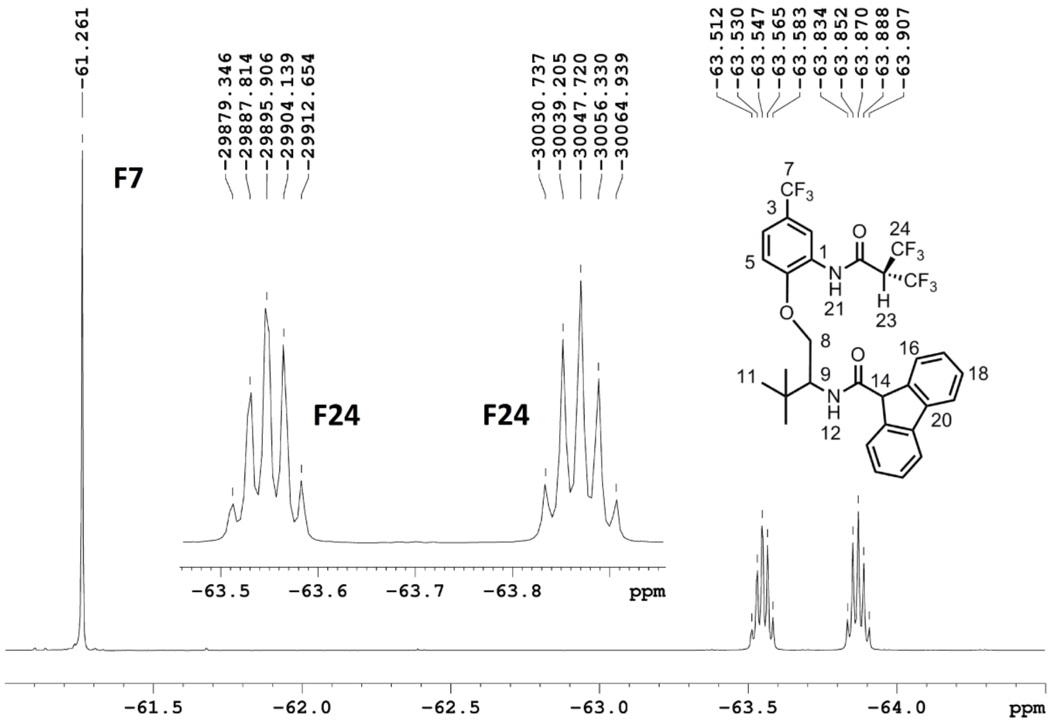

^{19}F NMR spectrum of **17** (23˚C, C_6D_6, 470 MHz).

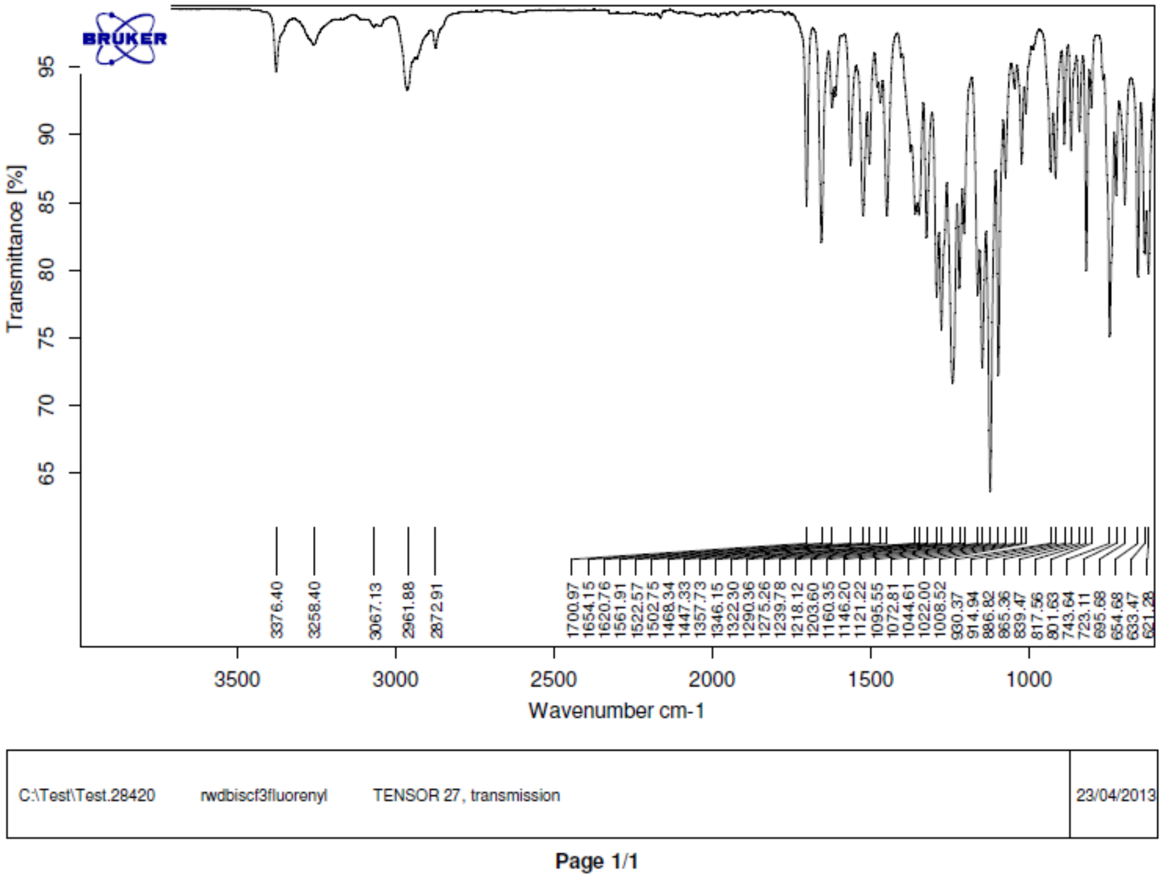

FT-IR spectrum of diffraction quality crystals of **17**.

Mass Spectrum SmartFormula Report

Analysis Info

Analysis Name	\\Utof\data\Mar 13\ESI39615_3_01_4935.d	Acquisition Date	15/03/2013 07:59:22
Method	2.5min_cal_sample_pos_naf_11-03-13_test.m	Operator	Mass Spec
Sample Name	ESI39615	Instrument / Ser#	micrOTOF 92
Comment			

Acquisition Parameter

Source Type	ESI	Ion Polarity	Positive	Set Nebulizer	2.0 Bar
Focus	Not active			Set Dry Heater	180 °C
Scan Begin	100 m/z	Set Capillary	4500 V	Set Dry Gas	10.0 l/min
Scan End	1000 m/z	Set End Plate Offset	-500 V	Set Divert Valve	Source

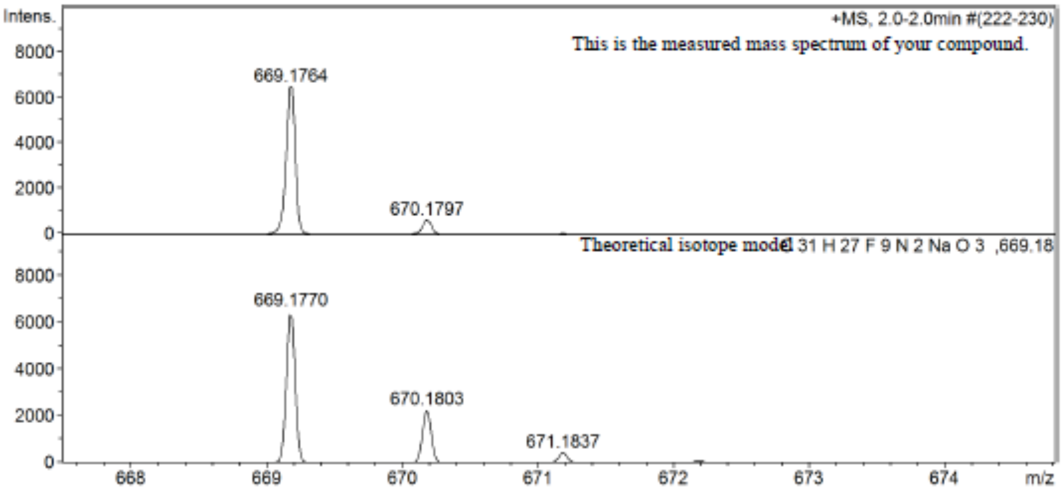

Meas. m/z	#	Formula	m/z	err [ppm]	Mean err [ppm]	rdb	e⁻ Conf	mSigma
669.1764								
	1	C 31 H 27 F 9 N 2 Na O 3	669.1770	1.0	1.0	14.5	even	129.13

High Resolution Mass Spectrum of **17**.

(S)-N-(2-(3,3-dimethyl-2-(2,2,2-trifluoroacetamido)butoxy)-5-(trifluoromethyl)phenyl)-3,3,3-trifluoro-2-(trifluoromethyl)propanamide [18]

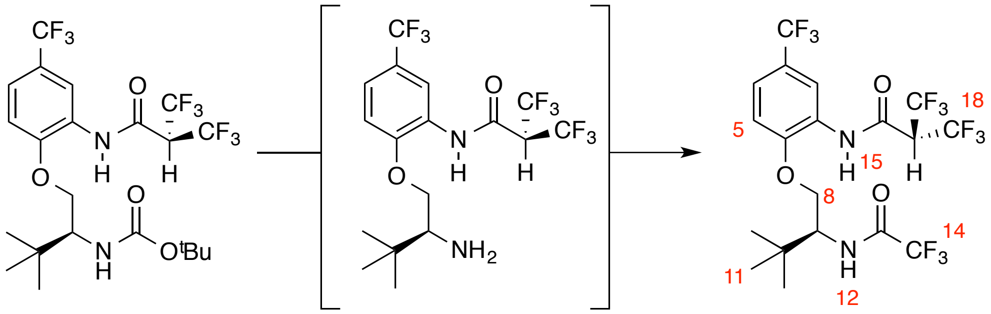

TFA (5mL) was added dropwise to a stirring solution of **8** (100 mg, 0.18 mmol) in DCM (5mL), at 0˚C and the reaction stirred for 1 hour. The mixture was concentrated in vacuo, suspended in Et_2O and 0.5mL HCl (2.0M in Et_2O) was added. The resultant white solid was filtered (*ca*. 75mg), DCM was added (50mL) and $NaHCO_3$ (50 mL, aq., saturated) was added. The organic phase was extracted, dried ($MgSO_4$) and concentrated to yield a white solid (*ca.* 65 mg). DCM (1mL), pyridine (0.3 mL) and trifluoroacetic anhydride (0.3 mL, 2.2 mmol) were added. The reaction was stirred for 12h before being concentrated in vacuo. The resulting solid was purified by column chromatography (6:1 PET:EtOAc) and recrystallized (pentanes) to yield 14 mg (0.025 mmol, 12 %) of the title compound. The title compound is almost insoluble in C_6D_6.

δH (500 MHz, C_6D_6, 23˚C, very low solubility): 9.23 (1H, s, H2), 8.82 (1H, bs, H15), 7.16 (1H, obscured, H4), 5.90 (1H, d, *J* = 8 Hz, H5), 5.77 (1H, d, *J* = 9 Hz, H12), 5.04 (1H, sept, *J* = 7 Hz, H17), 3.73 (1H, apparent triplet, *J* = 10 Hz, H8), 3.32 (1H, dd, J_1 = 9 J_2 = 2, H8), 2.87 (1H, apparent triplet, *J* =10 Hz, H9), 0.34 (9H, s, H11).

δH (500 MHz, $CDCl_3$, 23˚C): 8.87 (1H, d, *J* = 2 Hz, H2), 8.76 (1H, s, H15), 7.36 (1H, dd, J_1 = 8 Hz, J_2 = 2 Hz, H4), 6.85 (1H, d, *J* = 8 Hz, H5), 6.43 (1H, d, *J* = 10 Hz, H12), 4.56 (1H, sept, *J* = 7 Hz, H17), 4.38 (1H, dd, J_1= 9 Hz, J_2 = 3 Hz, H8), 4.34 (1H, apparent triplet, *J* = 9 Hz, H9), 4.02 (1H, apparent triplet, *J* =10 Hz, H9), 1.13 (9H, s, H11).

δC (125 MHz, $CDCl_3$, 23˚C): 159.74 (q, $^2J_{13C-19F}$ = 37 Hz, C13), 157.27 (m, C16), 149.08 (C6), 127.66 (C1), 124.62 (q, $^2J_{13C-19F}$ = 32 Hz, C3), 124.01 (q, $^1J_{13C-19F}$ = 271 Hz, C7), 122.14 (q, $^3J_{13C-19F}$ = 3 Hz, C2), 117.33 (q, $^2J_{13C-19F}$ = 3 Hz, C4), 121.43 (q, $^1J_{13C-19F}$ = 274 Hz, C18), 110.17 (C5), 69.29 (C8), 59.22 (C9), 54.55 (sept, $^2J_{13C-19F}$ = 30 Hz, C17), 32.43 (C10), 26.94 (C11).

δF {^{1}H} (236 MHz, C_6D_6, 23˚C): -61.77 (s, F7), -64.60 (m, F18), -76.41 (s, F14).

HRMS: (ES+): found 573.1028; Formula $C_{19}H_{18}F_{12}N_2NaO_3$, theoretical: 573.1018.

ν_{max} (neat, cm^{-1}): 3316.36, 2969.67, 1704.50, 1618.70, 1555.12, 1497.30, 1446.59, 1322.33, 1290.85, 1275.07, 1237.97, 1170.82, 1124.16, 1093.30, 932.12, 865.75, 816.45, 724.21, 636.54.

MP: 64-68 C.

$[\alpha]_D^{25.0}$ +24.0 (c = 0.001, $CHCl_3$)

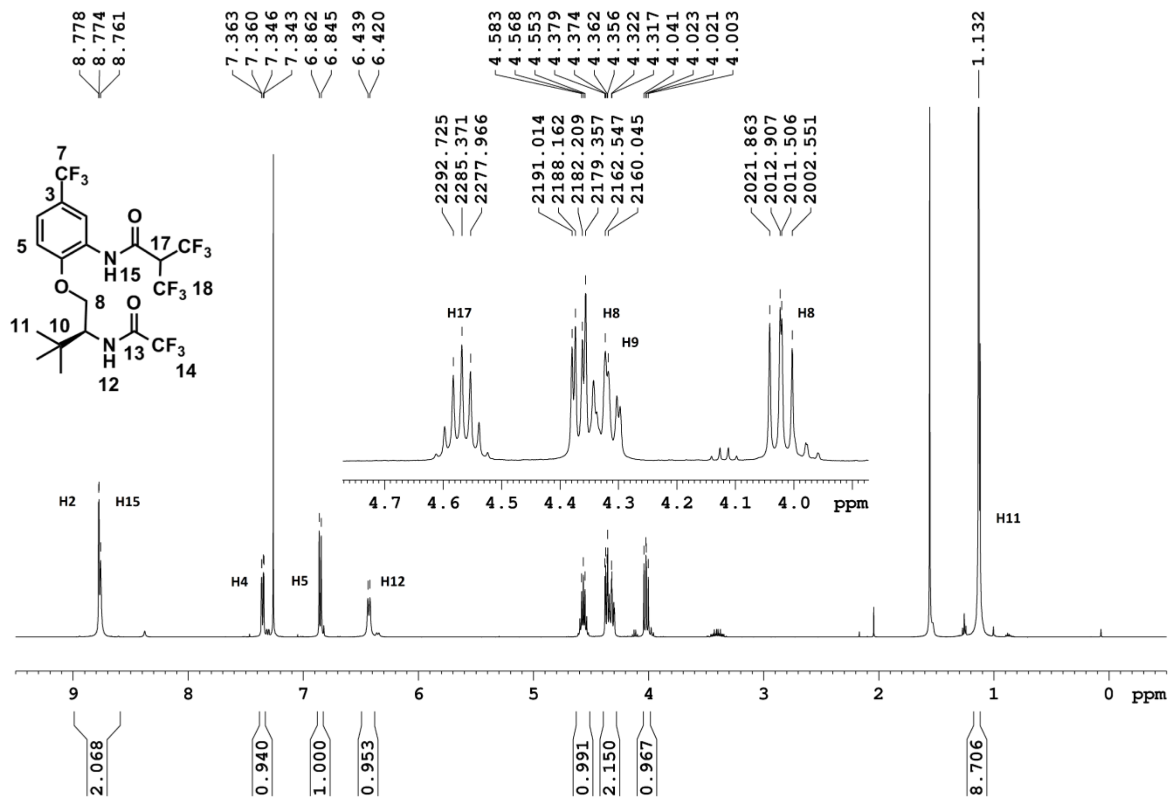

^{1}H NMR spectrum of **18** (23˚C, $CDCl_3$, 500 MHz).

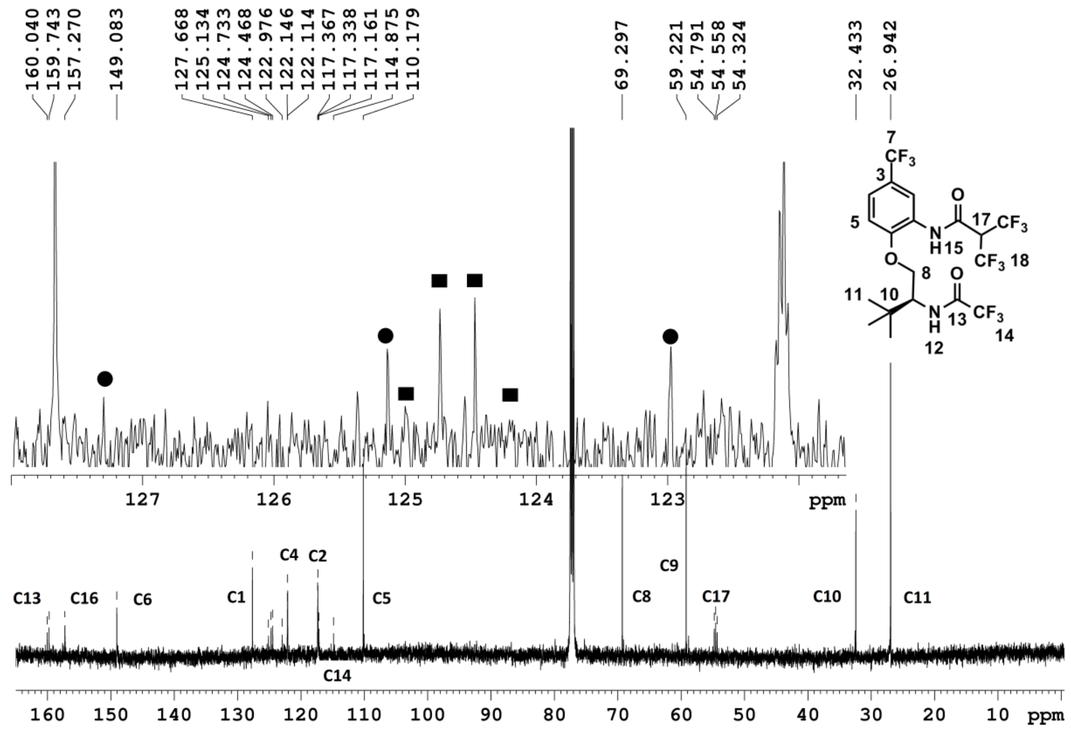

^{13}C NMR spectrum of **18** (23C, $CDCl_3$, 125 MHz).

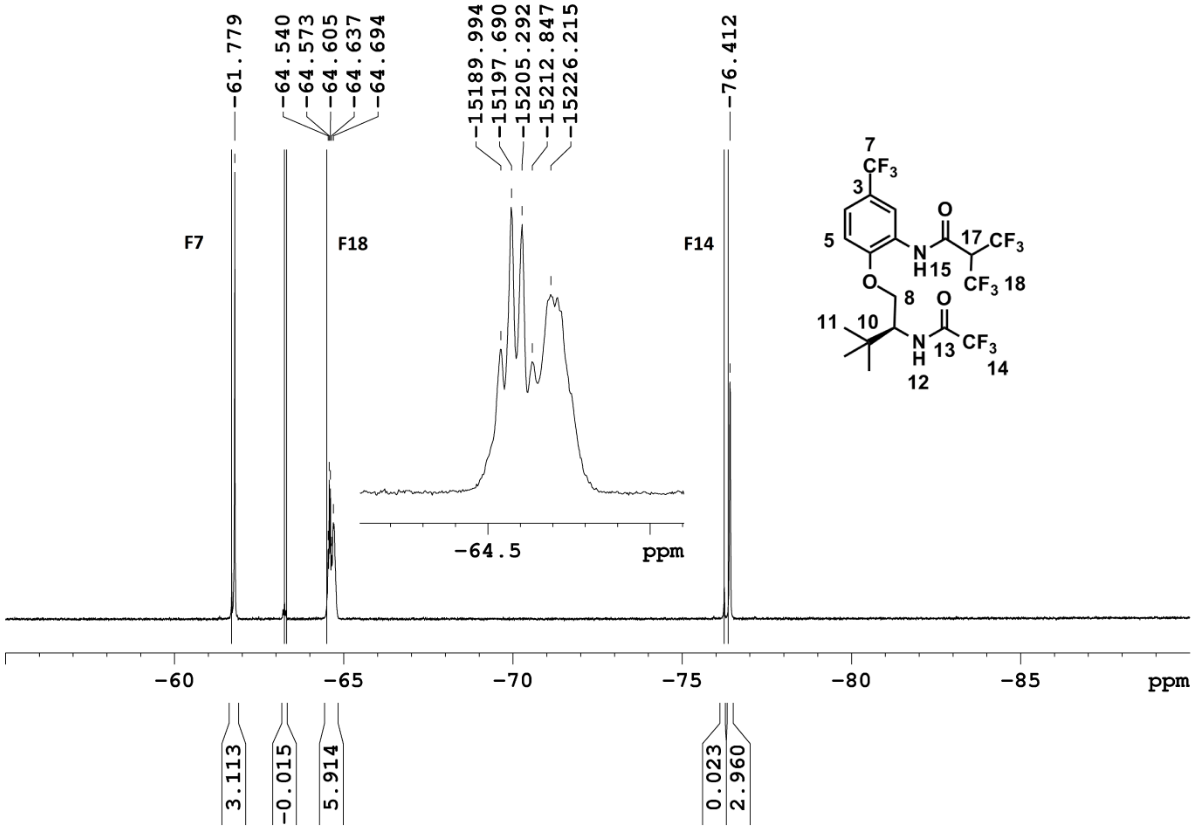

^{19}F NMR spectrum of **18** (23C, $CDCl_3$, 236 MHz).

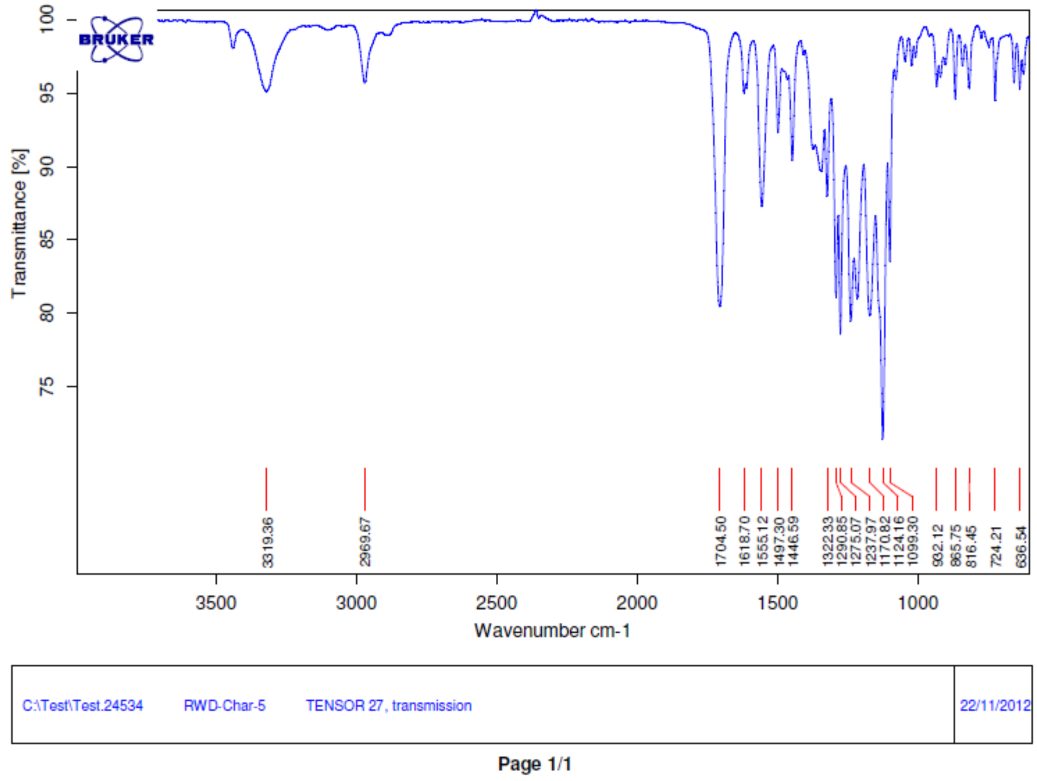

FT-IR spectrum of **18**.

(S)-N-(3,3-dimethyl-1-(4-(trifluoromethyl)-2-(9H-xanthene-9-carboxamido)phenoxy)butan-2-yl)-9H-xanthene-9-carboxamide [19]

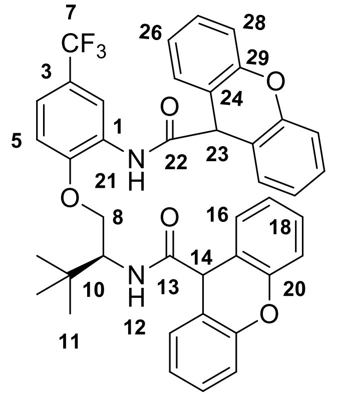

TFA (5mL) was added dropwise to a stirring solution of 8 (100 mg, 0.18 mmol) in DCM (5mL), at 0˚C and the reaction stirred for 1 hour. The mixture was concentrated in vacuo, suspended in Et_2O and 0.5mL HCl (2.0M in Et_2O) was added. The resultant white solid was filtered (*ca*. 75mg), DCM was added (50mL) and $NaHCO_3$ (50 mL, aq., saturated) was added. The organic phase was extracted, dried ($MgSO_4$) and concentrated to yield **a** white solid (*ca.* 65 mg). This was directly subjected to the representative procedure: 60 mg amine (0.18 mmol), 150 mg EDCI.HCl (0.75 mmol), 170 mg xanthene-9-carboxylic acid (1.5 mmol), N,N-dimethylaminopyridine (7 mg, 0.05 mmol), 1 mL pyridine and 5 mL DCM. Chromatography (silica gel, petroleum ether : ethyl acetate 6:1), 2 separations, followed by recrystallization in *n*-heptane, 10 mg, 8%. Diffraction quality crystals were grown from EtOAc/Pentanes using the diffusion method but the structure could not be solved.

δH (500 MHz, C_6D_6, 23˚C): 7.34 (1H, *d*, 7 Hz, H25), 7.33 (1H, d, *J* 2 Hz, H2), 7.20 (1H, dd, J_1 8 Hz, J_2 2 Hz, H4), 7.08 (3H, m, H28, H18, H18), 7.00 (3H, m, H25, H19, H16), 6.95 (2H, m, H27, H19), 6.90 (3H, m, H26, H18, H16), 6.83 (1H, dt, J_1 7 Hz, J_2 2 Hz, H26), 6.79 (1H, dt, J_1 8 Hz, J_2 1 Hz, H17), 6.68 (1H, dt, J_1 8 Hz, J_2 1 Hz, H17), 6.22 (1H, s, H21), 6.15 (1H, d, *J* 9 Hz, H5), 4.80 (1H, s, H23), 4.77 (1H, s, H14), 3.61 (1H, m, H8), 3.51 (1H, m, H8), 3.50 (1H, d, *J* 10 Hz, H12), 2.93 (1H, dt, J_1 7 Hz, J_2 4 Hz, H9), 0.46 (9H, s, H11).

δC (125 MHz, C_6D_6, 23˚C): 172.42 (C22), 171.00 (C13), 152.29 (C29), 152.07 (C29), 151.85 (C20), 151.72 (C20), 146.97 (C6), 130.09 (C25), 130.03 (C25), 129.93 (C16), 129.91 (C16), 129.75 (C27), 129.67 (C27), 129.60 (C18), 129.49 (C18), 125.86 (q, $^1J_{13C-19F}$ 270 Hz, C7), 125.79 (q, $^3J_{13C-19F}$ 4 Hz, C4), 124.98 (q, $^3J_{13C-19F}$ 4 Hz, C2), 124.56 (C26), 124.46(C26), 123.73 (C17), 123.70 (C17), 122.00 (C1), 119.68 (C24), 119.58 (C24), 119.31(C15), 119.10 (C15), 118.30 (q, $^2J_{13C-19F}$ 32 Hz, C3),117.86 (C28), 117.83 (C28), 117.55 (C19), 117.29 (C19), 111.00 (C5), 65.32(C8), 60.31 (C9), 47.84 (C23), 46.12 (C14), 34.95 (C10), 26.68 (C11).

δF (236 MHz, C_6D_6, 23˚C): -60.68 (s, F7).

HRMS: (M+Na): found (715.2408); $C_{41}H_{35}F_3N_2O_5Na$, [715.2396] required.

ν_{max} (neat, cm^{-1}): 3421.57, 3250.89, 3069.69, 3044.47, 2963.49, 2871.47, 2470.16, 2360.30, 2341.87, 2279.67, 1735.41, 1655.88, 1618.82, 1603.44, 1576.52, 1536.65, 1481.02, 1455.78,

1400.09, 1385.00, 1330.07, 1304.07, 1258.20, 1226.76, 1209.69, 1156.17, 1110.00, 1075.65, 1033.79.

MP: 98-100 C.

$[\alpha]_D^{25.0}$ +26.0° (c = 0.001, $CHCl_3$).

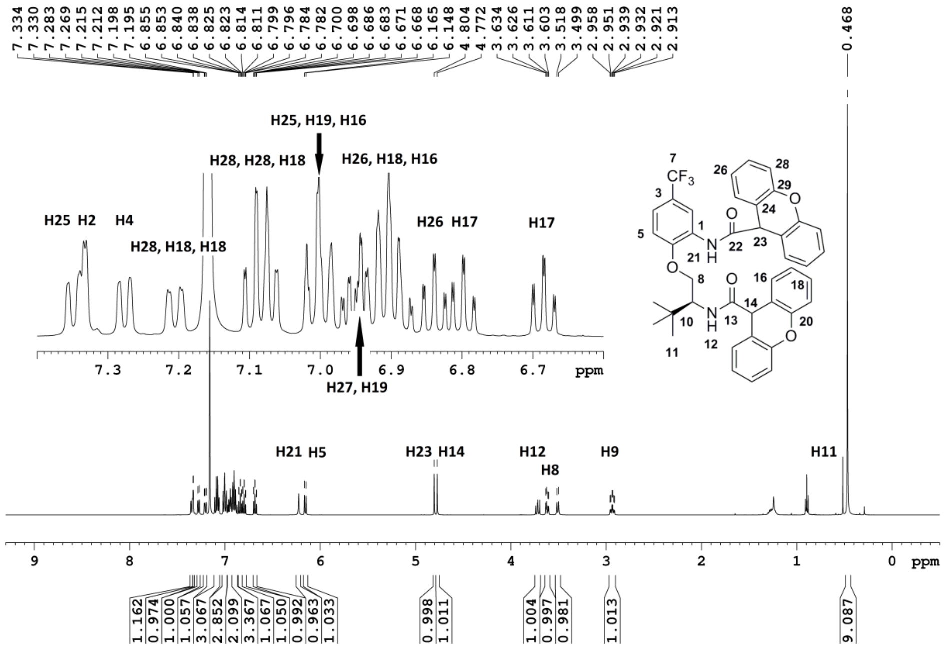

^{1}H NMR spectra of **19** (23˚C, C_6D_6, 500 MHz).

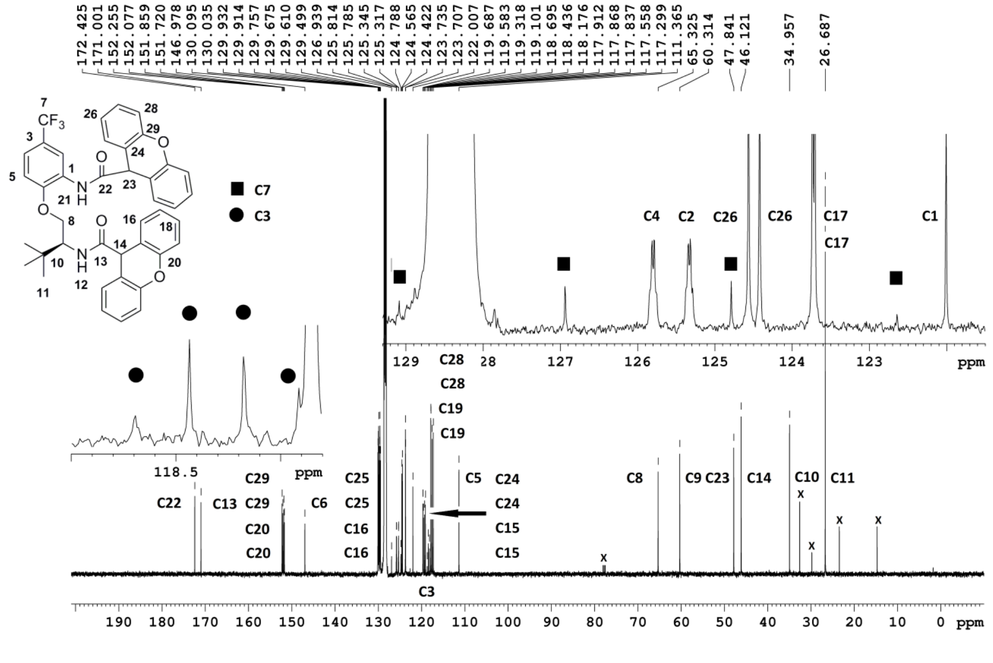

^{13}C NMR spectrum of **19** (23˚C, C_6D_6, 125 MHz).

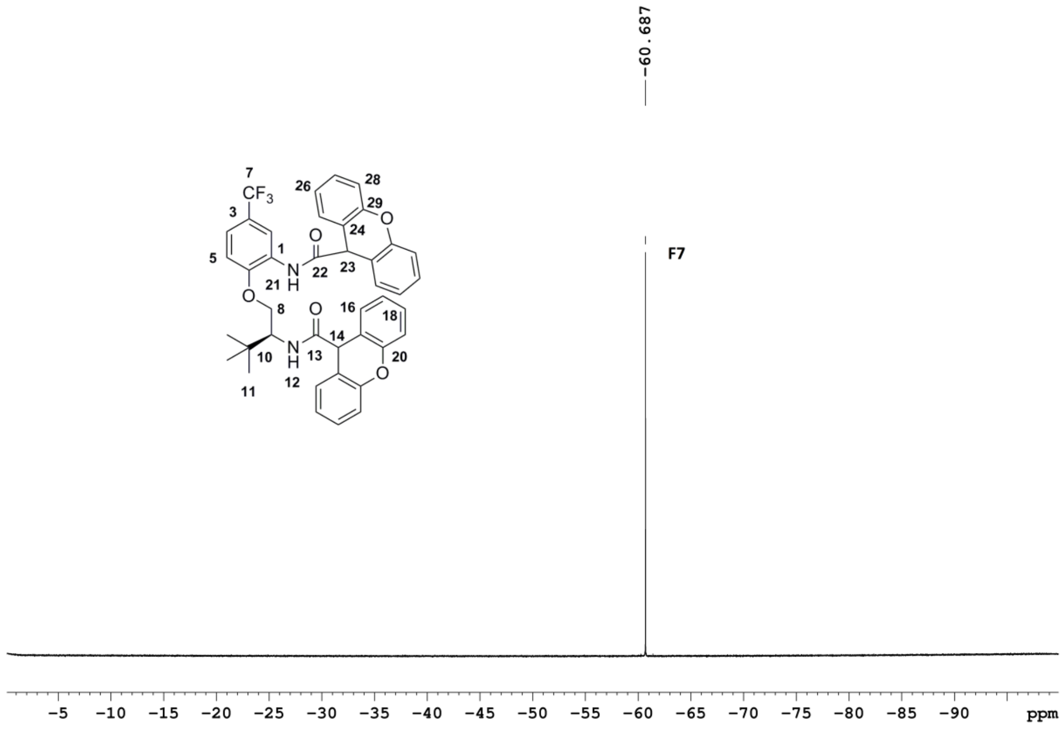

^{19}F NMR spectrum of **19** (23˚C, C_6D_6, 376 MHz).

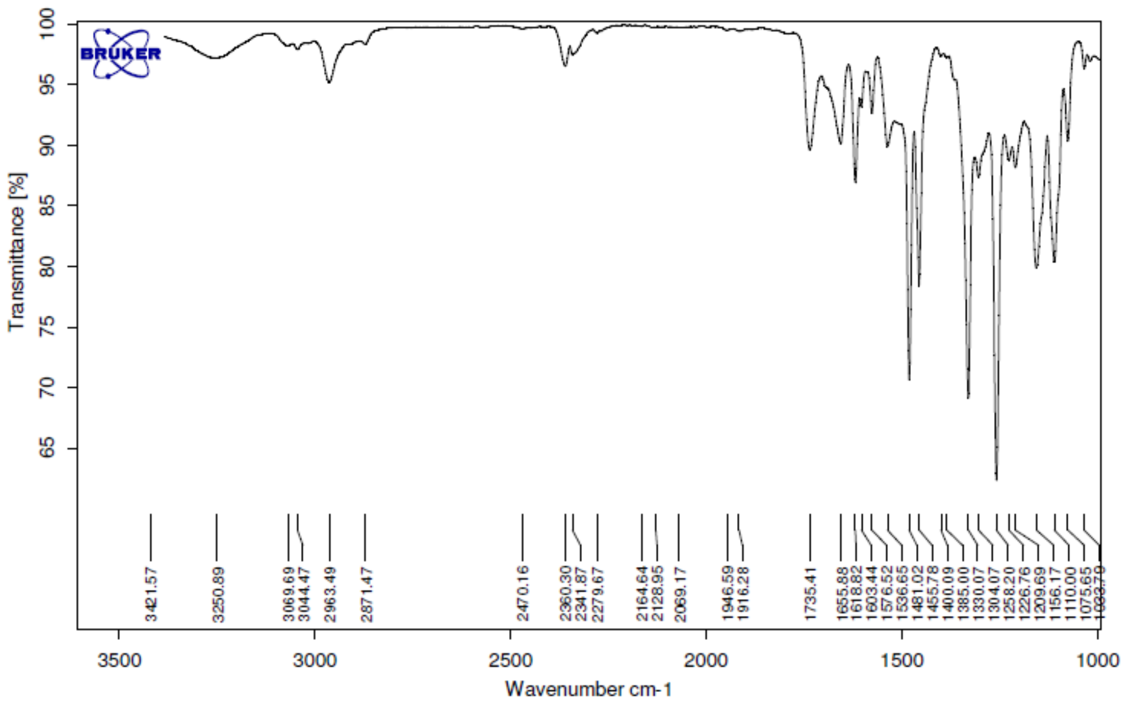

Solution State FT-IR spectrum of **19**.

Mass Spectrum SmartFormula Report

Analysis Info

Analysis Name	Z:\Sep 11\ESI30139_7_01_32266.d	Acquisition Date	12/09/2011 7:15 am
Method	2.5min_cal_sample_pos_Naf_11-10-10.m	Operator	Mass Spec
Sample Name	ESI30139	Instrument / Ser#	micrOTOF 92
Comment			

Acquisition Parameter

Source Type	ESI	Ion Polarity	Positive	Set Nebulizer	2.0 Bar
Focus	Not active			Set Dry Heater	180 °C
Scan Begin	100 m/z	Set Capillary	4500 V	Set Dry Gas	10.0 l/min
Scan End	1500 m/z	Set End Plate Offset	-500 V	Set Divert Valve	Source

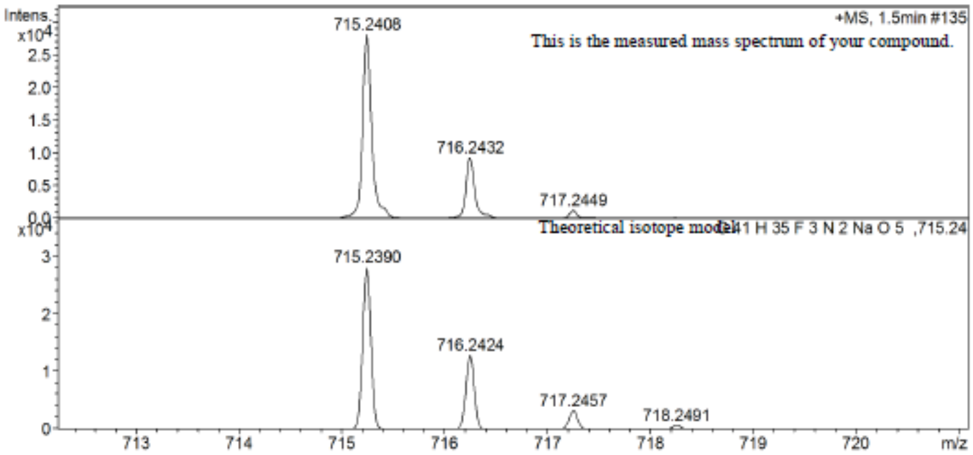

Meas. m/z	#	Formula	m/z	err [ppm]	Mean err [ppm]	rdb	e⁻ Conf	mSigma
715.2408								
	1	C 41 H 35 F 3 N 2 Na O 5	715.2390	-2.5	-2.1	23.5	even	71.48

High resolution mass spectrum of **19**.

(S)-N-(2-(3,3-dimethyl-2-pivalamidobutoxy)-5-(trifluoromethyl)phenyl)-3,3,3-trifluoropropanamide [20]

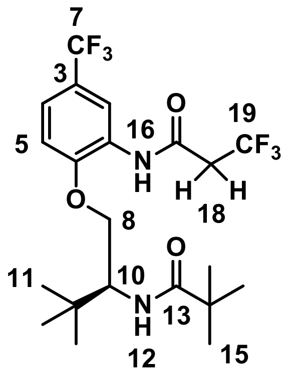

20 was isolated (4mg) during the synthesis of **21**.

δH (500 MHz, C_6D_6, 23˚C): 9.37 (1H, d, *J* 2 Hz, H2), 9.16 (1H, s, H16), 7.12 (1H, dd, J_1 9 Hz, J_2 2 Hz, H4), 6.13 (1H, d, *J* 8 Hz, H5), 5.29 (1H, d, *J* 10 Hz, H12), 4.11 (1H, dt, J_1 10 Hz, J_2 3 Hz, H9), 3.73 (3H, m, H18, H18, H8), 3.23 (1H, dd, J_1 10 Hz, J_2 9 Hz, H8), 0.91 (9H, H15), 0.63 (9H, H11).

δC (125 MHz, C_6D_6, 23˚C): 180.31 (C13), 162.51 (C17, q, $^3J_{13C-19F}$ 4 Hz), 150.07 (C6), 129.79 (C1), 125.66 (C19, q, $^1J_{13C-19F}$ 276 Hz), 125.47 (C7, q, $^1J_{13C-19F}$ 272 Hz), 124.28 (C3, q, $^2J_{13C-19F}$ 34 Hz), 122.30 (C4, q, $^3J_{13C-19F}$ 4 Hz), 117.30 (C2, q, $^3J_{13C-19F}$ 3 Hz), 110.69 (C5), 70.47 (C8), 57.38 (C9), 42.01 (C18, q, $^2J_{13C-19F}$ 29 Hz), 39.43 (C14), 32.38 (C10), 27.65 (C15), 26.87 (C11).

δF (236 MHz, C_6D_6, 23˚C): -62.10 (F7), -63.12 (3F, t, $^3J_{19F-1H}$ 9 Hz).

HRMS: $C_{21}H_{28}F_6N_2O_3$ theoretical [493.1869], observed [493.1889].

ν_{max} (neat, cm^{-1}): 3470.40, 3422.02, 3282.77, 2968.84, 2874.33, 1709.05, 1692.49, 1638.31, 1615.39, 1551.69, 1519.16, 1495.05, 1442.32, 1401.79, 1371.53, 1350.65, 1324.03, 1270.45, 1222.86, 1201.68, 1166.22, 1131.24, 1110.06, 1075.78, 1045.67, 1011.23, 953.52.

MP: 138˚C.

$[\alpha]_D^{25.0}$ + 37.5 (c = 0.001, $CHCl_3$).

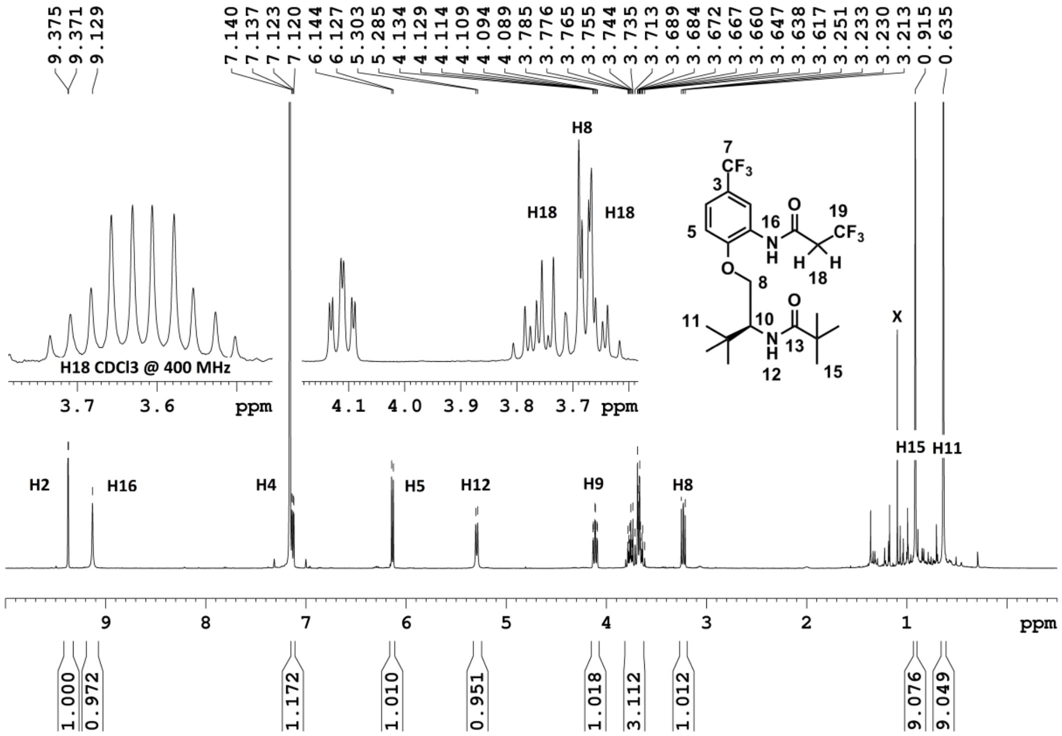

^{1}H NMR spectrum of **20** (23˚C, C_6D_6, 500 MHz).

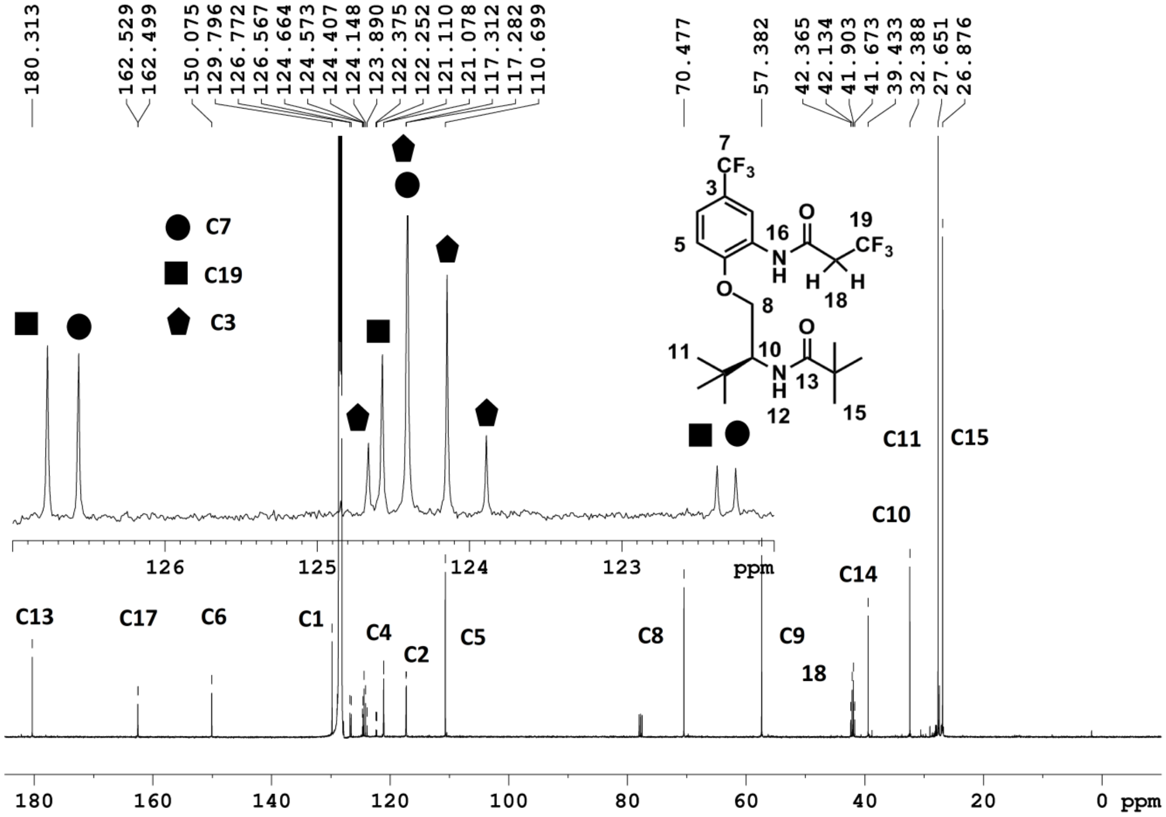

^{13}C NMR spectrum of **20** (23˚C, C_6D_6, 125 MHz).

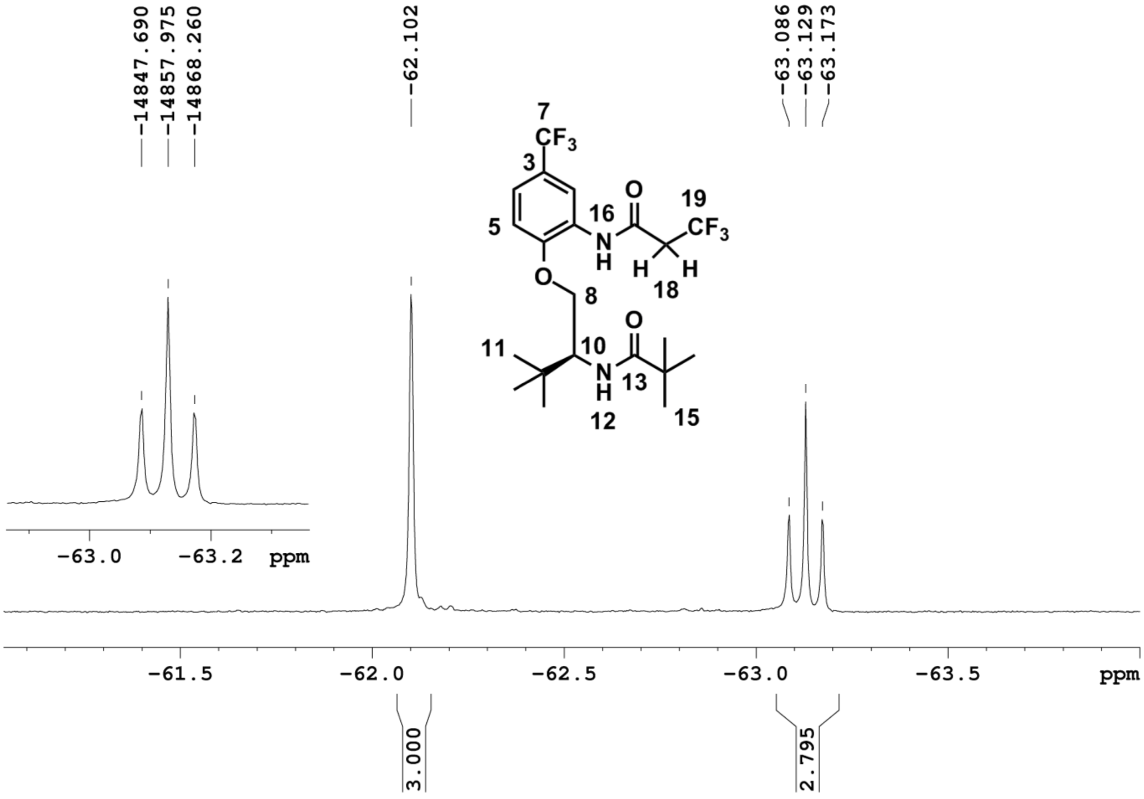

^{19}F NMR spectrum of **20** (23˚C, C_6D_6, 236 MHz).

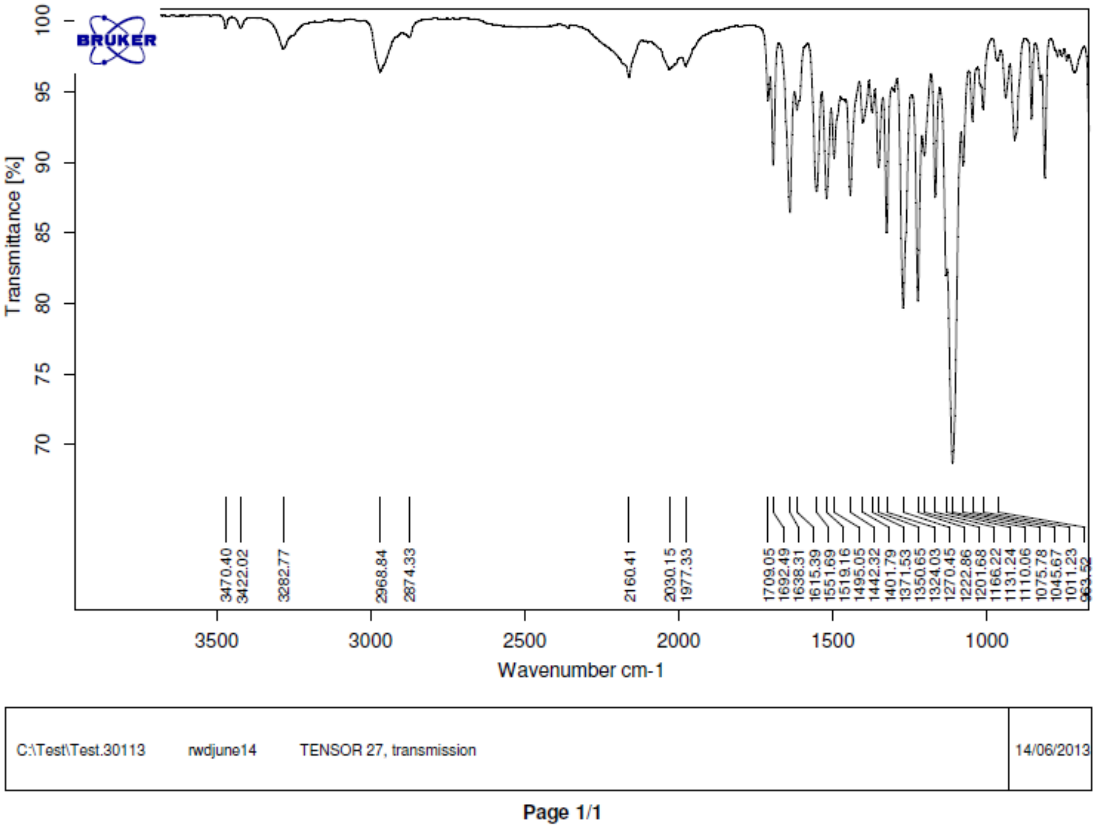

FT-IR of diffraction quality crystals of **20**.

Mass Spectrum SmartFormula Report

Analysis Info

Analysis Name	\\Utof\data\June 13\ESI41345_2_01_8902.d	Acquisition Date	12/06/2013 08:04:36
Method	2.5min_cal_sample_pos_naf_14-05-10 test.m	Operator	Mass Spec
Sample Name	ESI41345	Instrument / Ser#	micrOTOF 92
Comment			

Acquisition Parameter

Source Type	ESI	Ion Polarity	Positive	Set Nebulizer	2.0 Bar
Focus	Not active			Set Dry Heater	180 °C
Scan Begin	100 m/z	Set Capillary	4500 V	Set Dry Gas	10.0 l/min
Scan End	1000 m/z	Set End Plate Offset	-500 V	Set Divert Valve	Source

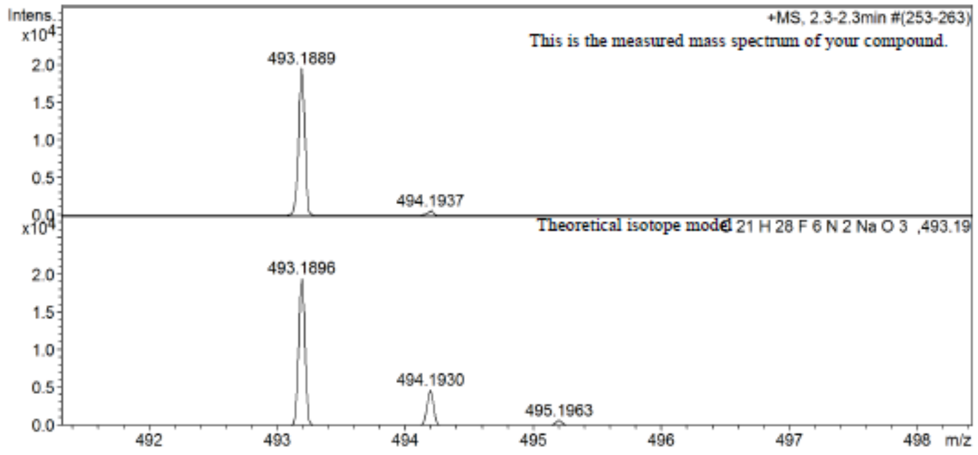

Meas. m/z	#	Formula	m/z	err [ppm]	Mean err [ppm]	rdb	e⁻ Conf	mSigma
493.1889								
	1	C 21 H 28 F 6 N 2 Na O 3	493.1896	1.4	1.3	5.5	even	119.38

High resolution mass spectrum of **20**.

(S)-tert-butyl (3,3-dimethyl-1-(4-(trifluoromethyl)-2-(3,3,3-trifluoropropanamido)phenoxy)butan-2-yl)carbamate [21]

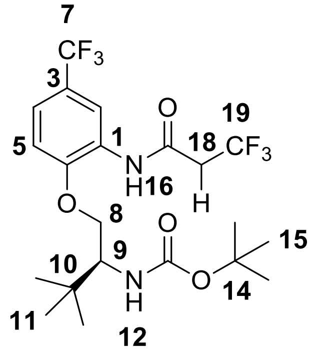

Prepared according to the representative procedure from (S)-tert-butyl (1-(2-amino-4-(trifluoromethyl)phenoxy)-3,3-dimethylbutan-2-yl)carbamate (100 mg, 0.26 mmol), EDCI.HCl (200 mg, 1.06 mmol), 3,3,3 trifluoromethylpropionic acid (136 mg, 1.06 mmol), N,N-dimethylaminopyridine (7 mg, 0.05 mmol), pyridine (1.1 mL) and DCM (20 mL). Chromatography (silica gel, petroleum ether : ethyl acetate 5:1), 98 mg, 80%. Diffraction quality crystals, colorless blocks, obtained from pure *n*-pentanes.

δH (500 MHz, C_6D_6, 23°C): 9.34 (1H, s, H2), 9.08 (1H, s, H16), 7.13 (1H, d, obscured, H4), 6.14 (1H, d, *J* = 9 Hz, H5), 4.34 (1H, d, *J* = 10 Hz, H12), 3.72 (1H, apparent triplet, J = 10 Hz, H9), 3.56 (1H, doublet, *J* = 9 Hz, H8), 3.44 (2H, ABX_3, v = 0.02, $^{1}J_{1H\text{-}1H}$ = 15 Hz, $^{3}J_{1H\text{-}19F}$ = 9 Hz, H18), 3.19 (1H, apparent triplet, *J* = 10 Hz, H8), 1.34 (9H, s, H15), 0.56 (9H, s, H11).

δC (125 MHz, C_6D_6, 23°C): 161.95 (C17, d, $^{3}J_{13C\text{-}19F}$ = 3 Hz), 157.77 (C13), 150.81 (C6), 129.61 (C4), ca 129-128 (C1, obscured by benzene signal), 125.47 (C7, q, $^{1}J_{13C\text{-}19F}$ = 272 Hz), 125.71 (C19, q, $^{1}J_{13C\text{-}19F}$ = 277 Hz), 124.39 (C3, q, $^{2}J_{13C\text{-}19F}$ = 33 Hz), 121.34 (C4, q, $^{3}J_{13C\text{-}19F}$ = 4 Hz), 117.48 (C2, bm), 111.13 (C5), 80.15 (C14), 70.85 (C8), 59.92 (C9), 42.20 (C18, q, $^{2}J_{13C\text{-}19F}$ = 20 Hz), 32.55 (C10), 28.68 (C15), 28.81 (C11).

δF (470 MHz, C_6D_6, 23°C): -61.58 (F7), -62.64 (F19, apparent triplet, $^{3}J_{19F\text{-}1H}$ = 10 Hz).

HRMS: found (509.1845); $C_{21}H_{28}F_3N_2O_4Na$, requires 509.1846.

ν_{max} (neat, cm^{-1}): 3456.53, 3402.32, 3324.46, 2977.63, 2938.75, 2878.88, 2294.03, 2162.87, 2049.92, 1681.51, 1615.04, 1607.00, 1545.28, 1509.75, 1495.19, 1440.35, 1394.27, 1370.00, 1347.11, 1323.31, 1297.60, 1269.03, 1254.64, 1221.76, 1166.16, 1120.50, 1096.47, 1040.65, 1007.93, 968.03, 952.88, 939.28, 906.88, 856.07, 826.76, 809.94, 783.42, 757.27, 700.29, 669.40, 652.42, 636.96.

MP: 111-112 C.

$[\alpha]_D^{25.0}$ +12.0° (c = 0.001, $CHCl_3$).

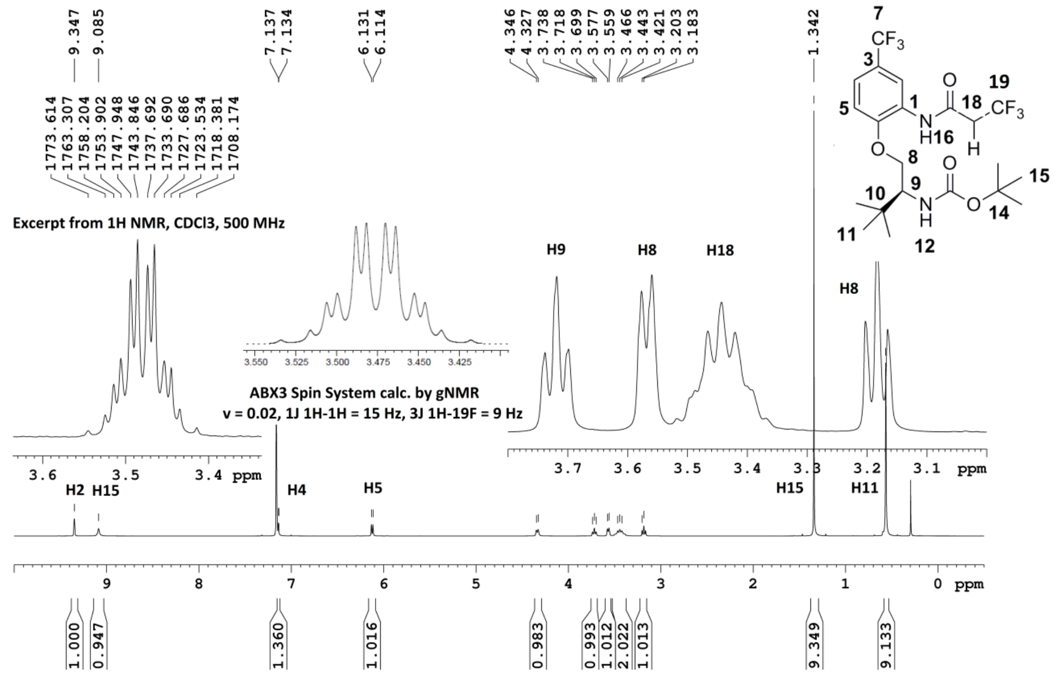

^{1}H NMR spectrum of **21** (23˚C, C_6D_6, 500 MHz) showing complex ABX_3 splitting pattern (3.45 ppm) resulting from diastereotopic geminal protons. An excerpt from the ^{1}H NMR spectrum in $CDCl_3$ at 23C is shown in comparison to a ABX_3 spin system calculated by gNMR.

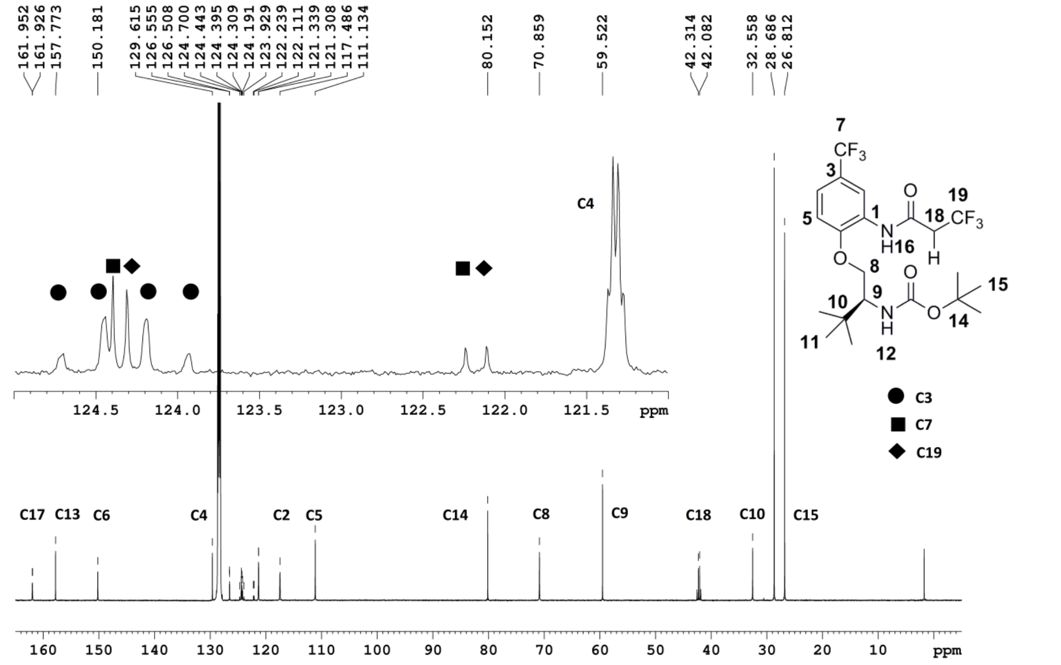

^{13}C NMR spectrum of **21** (23˚C, C_6D_6, 125 MHz)

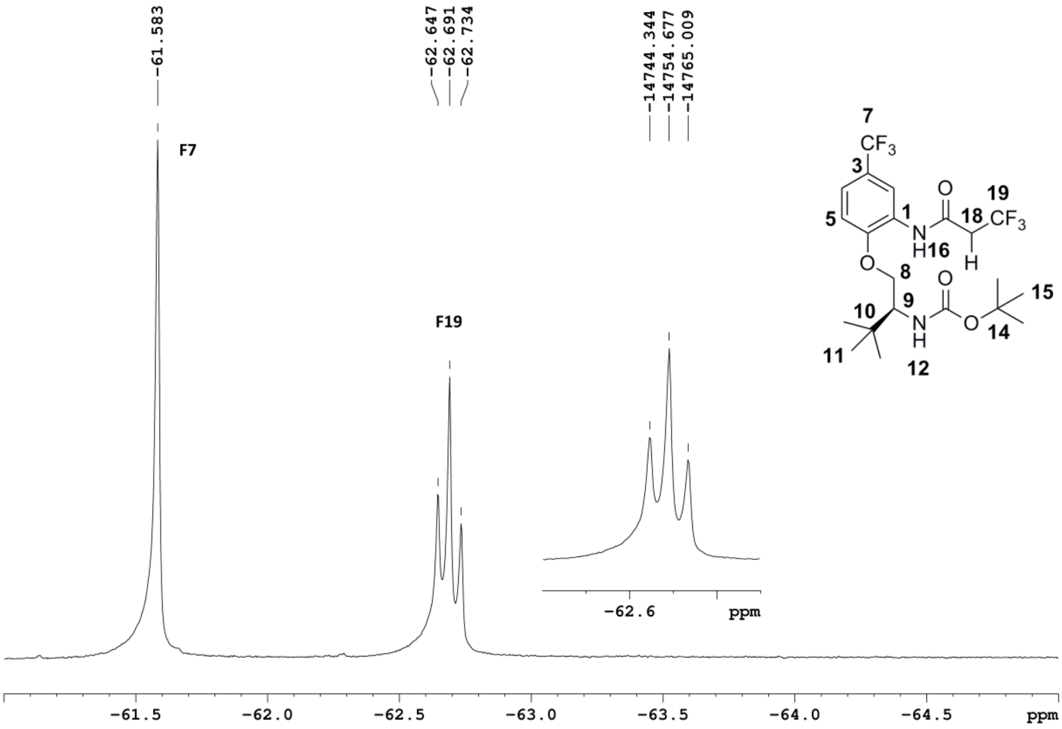

^{19}F

^{19}F NMR spectrum of **21** (23˚C, C_6D_6, 470 MHz)

Mass Spectrum SmartFormula Report

Analysis Info

Analysis Name	Z:\Mar 12\ESI33088_4_01_38987.d	Acquisition Date	08/03/2012 7:50 am
Method	2.5min_cal_sample_pos_Naf_11-10-10.m	Operator	Mass Spec
Sample Name	ESI33088	Instrument / Ser#	micrOTOF 92
Comment			

Acquisition Parameter

Source Type	ESI	Ion Polarity	Positive	Set Nebulizer	2.0 Bar
Focus	Not active			Set Dry Heater	180 °C
Scan Begin	100 m/z	Set Capillary	4500 V	Set Dry Gas	10.0 l/min
Scan End	1000 m/z	Set End Plate Offset	-500 V	Set Divert Valve	Source

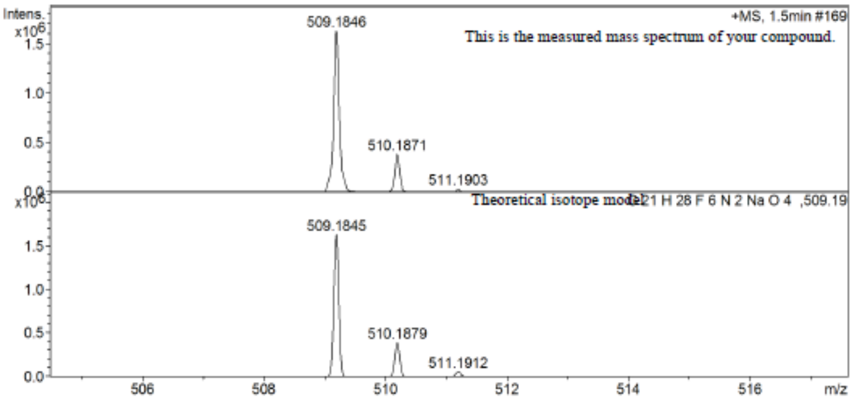

Meas. m/z	#	Formula	m/z	err [ppm]	Mean err [ppm]	rdb	e⁻ Conf	mSigma
509.1846								
	1	C 21 H 28 F 6 N 2 Na O 4	509.1845	-0.1	0.2	5.5	even	8.79

High-resolution mass spectrum of **21**.

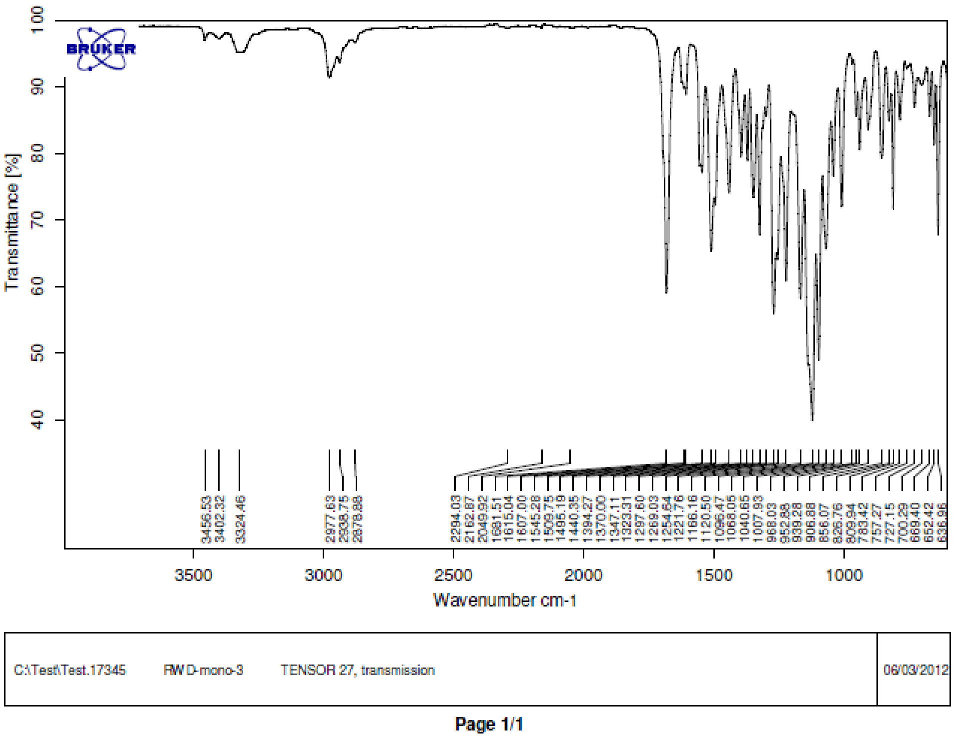

Solid State FT-IR of **21**.

(S)-tert-butyl (3,3-dimethyl-1-(2-(2-(phenylsulfonyl)acetamido)-4-(trifluoromethyl)phenoxy)butan-2-yl)carbamate [22]

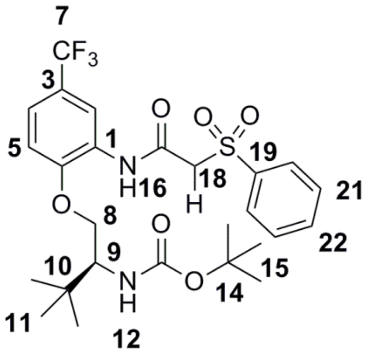

Prepared according to the representative procedure from (S)-tert-butyl (1-(2-amino-4-(trifluoromethyl)phenoxy)-3,3-dimethylbutan-2-yl)carbamate (60 mg, 0.16 mmol), 122 mg EDCI.HCl (.63 mmol), 126 mg phenyl(sulfonyl)acetic acid (0.64 mmol), 3 mg N,N-dimethylaminopyridine (.02 mmol), 0.5 mL pyridine and 10 mL DCM. Chromatography (silica gel, petroleum ether : ethyl acetate 1:2), yielded 35 mg, 39% of the title compound as a white solid.

δH (500 MHz, C_6D_6, 23˚C): 9.49 (1H, s, H16), 9.25 (1H, s, H2), 7.92 (1H, d, *J* 8 Hz, H20), 7.22 (1H, d, *J* 9 Hz, H4), 6.96 (3H, m, H21, H21, H22), 6.31 (1H, d, *J* 8 Hz, H5), 5.75 (1H, d, *J* 10 Hz, H12), 4.06 (1H, m, H9), 3.965, 3.955 (2H, dd, $^1J_{1H\text{-}1H}$ 14 Hz, H18), 3.73 (2H, m, H8, H8), 1.50 (9H, s, H15), 0.98 (9H, s, H11).

δC (125 MHz, C_6D_6, 23˚C): 159.40 (C17), 157.10 (C13), 150.27 (C6), 139.31 (C19), 134.36 (C21), 129.58 (C20), 128.83 (C22), 125.37 (q, $^1J_{13C\text{-}19F}$ 270 Hz, C7), 122.24 (q, $^2J_{13C\text{-}19F}$ 32 Hz, C3), 121.91 (q, $^3J_{13C\text{-}19F}$ 3 Hz, C4), 117.45 (q, $^1J_{13C\text{-}19F}$ 4 Hz, C2), 111.64 (C5), 79.50 (C14), 70.06 (C8), 63.16 (C18), 58.33 (C9), 34.34 (C10), 28.82 (C15), 27.54 (C11).

δF (470MHz, C_6D_6, 23˚C): -61.26 (F7).

IR solution phase (cm^{-1}): 3326.36, 2969.53, 2361.16, 2341.48, 1687.59, 1615.08, 1605.92, 1547.49, 1520.44, 1495.45, 1444.35, 1392.20, 1367.32, 1324.74, 1273.76, 1249.03, 1206.56, 1160.67, 1120.86, 1084.83, 1065.67, 1013.60, 1013.60, 950.28, 933.01, 899.10, 857.97, 813.04, 753.55, 735.45, 686.89, 668.18, 655.43, 635.82.

Mp: 68˚C.

$[\alpha]_D^{25.0}$ +12.0 (c = 0.001, $CHCl_3$).

HRMS: (ES+): $C_{26}H_{33}F_3N_2O_6SNa$ [requires 581.1904]; observed 581.1896.

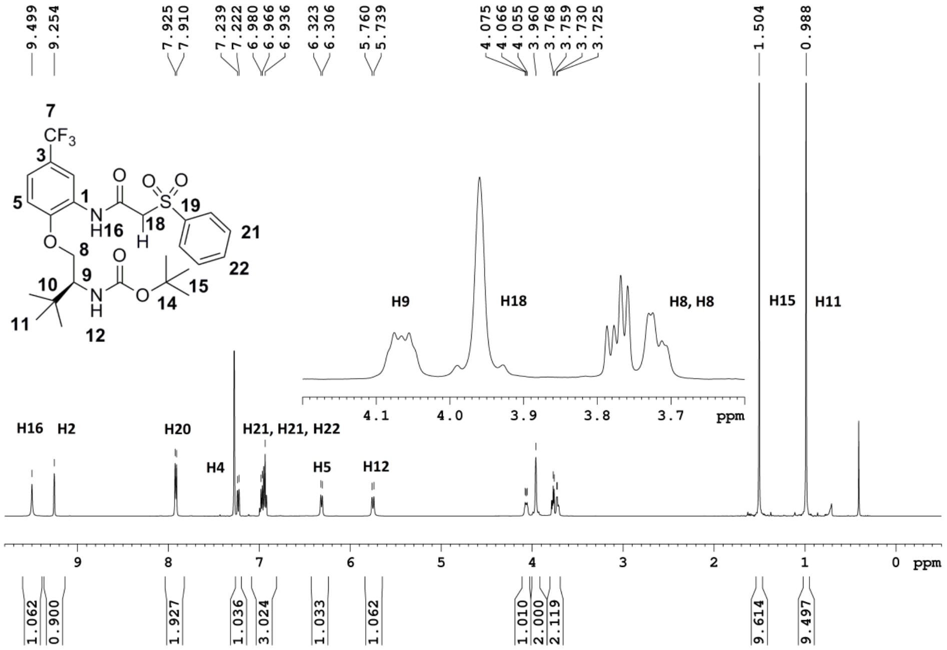

^{1}H NMR spectrum of **22** (23˚C, C_6D_6, 500 MHz).

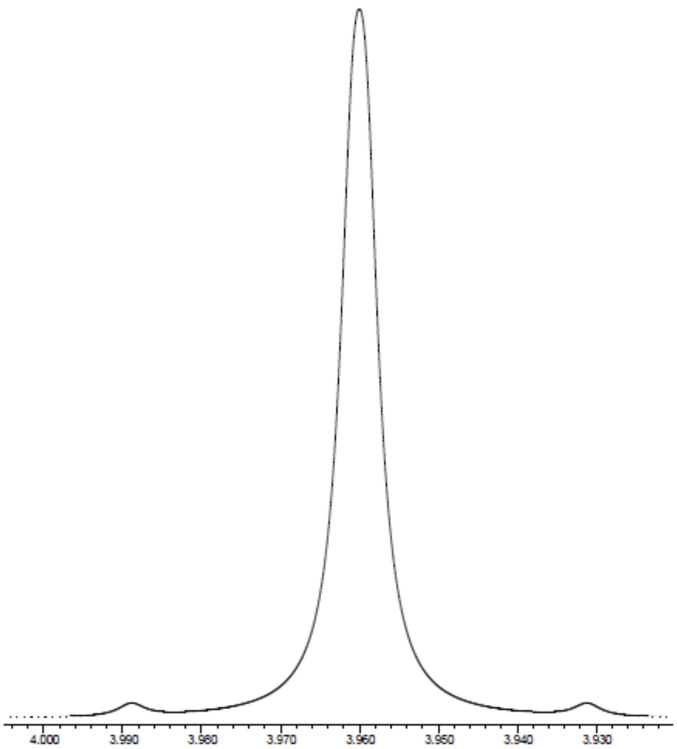

gNMR calculated spectrum of **22** showing doublet of doublet splitting pattern from diastereotopic H18 protons. d = 0.01 ppm (3.955, 3.965), $^1J_{1H\text{-}1H}$ = 14 Hz, , lb = 2.0. The calculated spectrum reproduces exactly the observed spectrum.

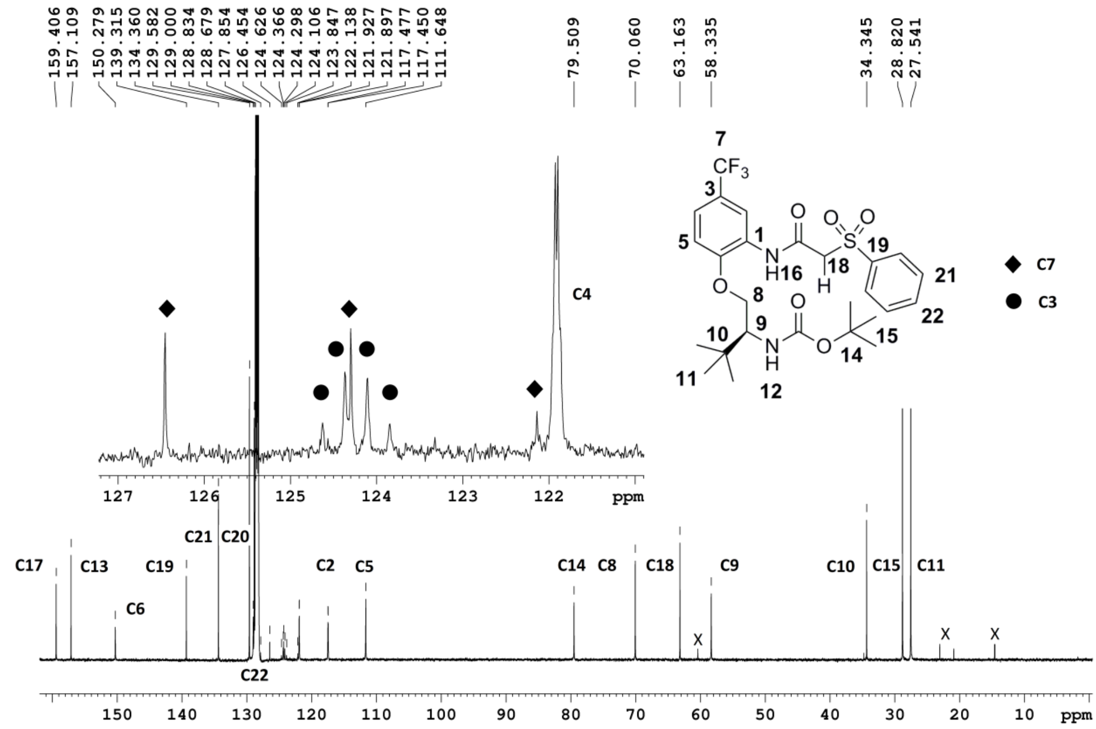

^{13}C NMR spectrum of **22** (23˚C, C_6D_6, 125 MHz).

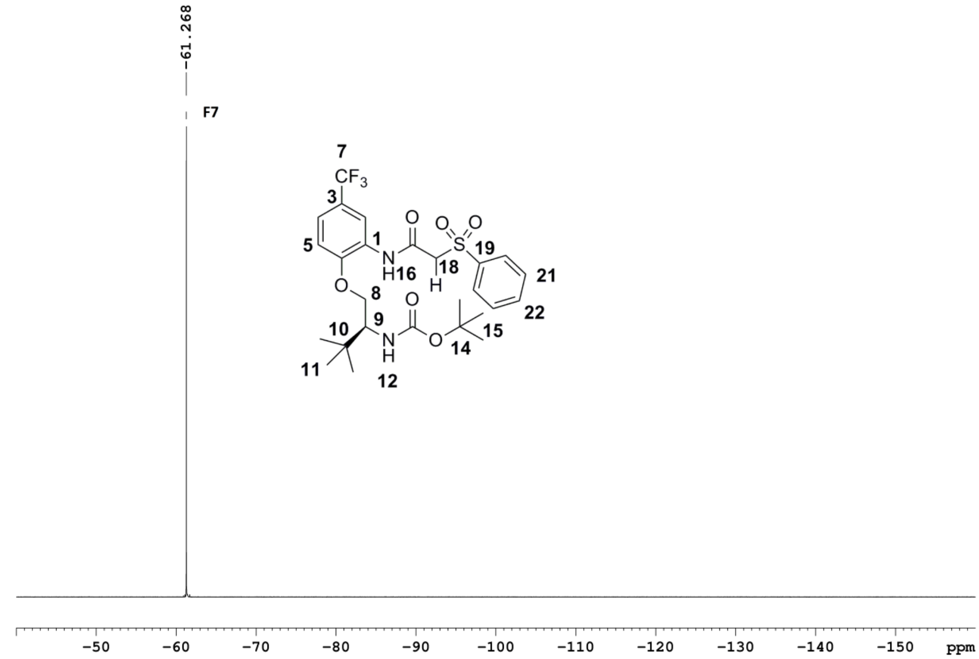

^{19}F NMR spectrum of **22** (23˚C, C_6D_6, 470 MHz).

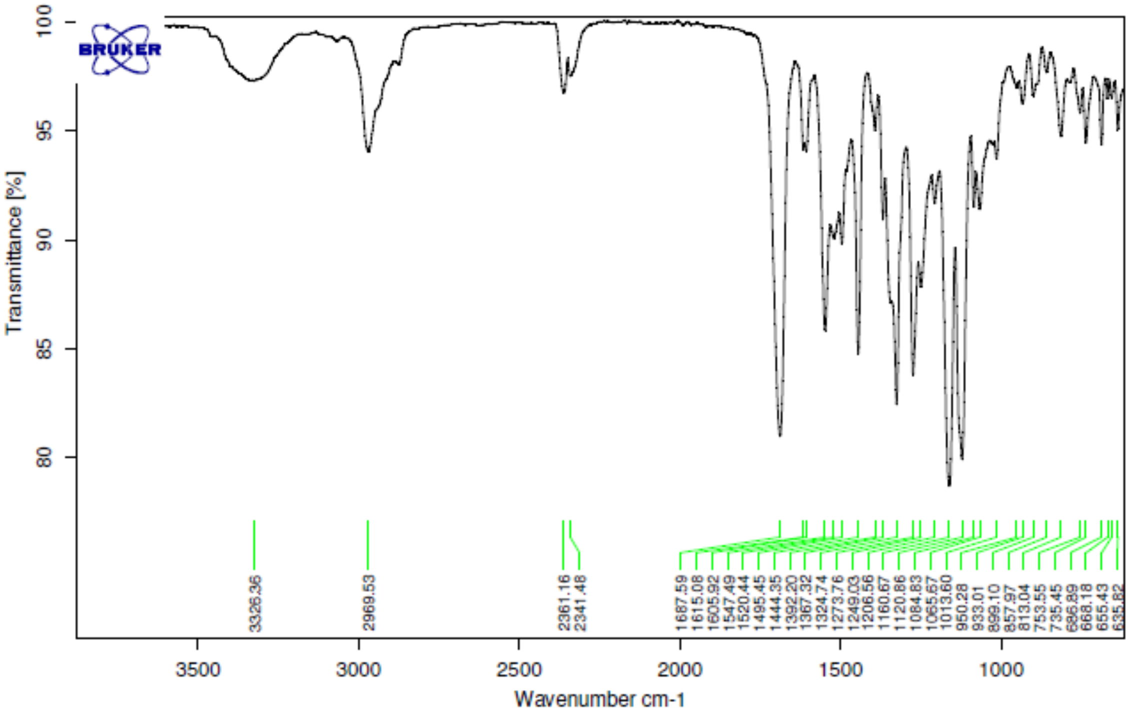

FT-IR of diffraction quality crystals of **22**.

Mass Spectrum SmartFormula Report

Analysis Info			
		Acquisition Date	13/04/2012 08:22:33
Analysis Name	\\Utof\Data\Apr12\ESI33937_15_01_40643.d		
Method	2.5min_cal_sample_pos_Naf_11-10-10.m	Operator	Mass Spec
Sample Name	ESI33937	Instrument / Ser#	micrOTOF 92
Comment			

Acquisition Parameter					
Source Type	ESI	Ion Polarity	Positive	Set Nebulizer	2.0 Bar
Focus	Not active			Set Dry Heater	180 °C
Scan Begin	100 m/z	Set Capillary	4500 V	Set Dry Gas	10.0 l/min
Scan End	1000 m/z	Set End Plate Offset	-500 V	Set Divert Valve	Source

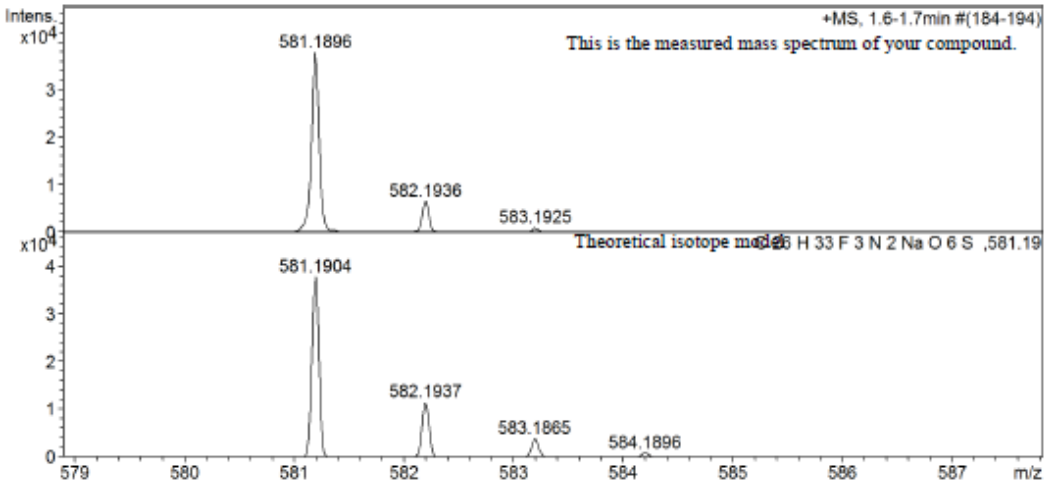

Meas. m/z	#	Formula	m/z	err [ppm]	Mean err [ppm]	rdb	e⁻ Conf	mSigma
581.1896								
	1	C 26 H 33 F 3 N 2 Na O 6 S	581.1904	1.4	0.9	9.5	even	73.73

High resolution mass spectrum of **22**.

(S)-tert-butyl (3,3-dimethyl-1-(4-(trifluoromethyl)-2-(9H-xanthene-9-carboxamido)phenoxy)butan-2-yl)carbamate [23]

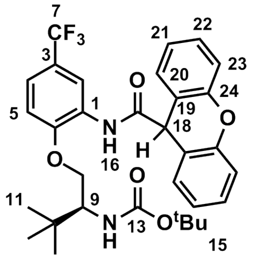

Prepared according to the representative procedure from (S)-tert-butyl (1-(2-amino-4-(trifluoromethyl)phenoxy)-3,3-dimethylbutan-2-yl)carbamate (60 mg, 0.16 mmol), EDCI.HCl (122 mg, 0.63 mmol), 9H-xanthene-9-carboxylic acid (144 mg 0.64 mmol), N,N-dimethylaminopyridine (3 mg, 0.02 mmol), pyridine (0.5 mL) and DCM (10 mL). Chromatography (silica gel, petroleum ether : ethyl acetate 1:2), yielded 15 mg, 16% of the title compound as a white solid.

δH (500 MHz, C_6D_6, 23˚C): 8.66 (1H, s, H2), 8.40 (1H, s, H16), 7.61 (1H, d, *J* 7 Hz, H20), 7.33 (2H, m, H20, H22), 7.29 (1H, m, H22), 7.21 (2H, H4, H21), 7.12 (1H, H21), 7.15 (2H, H23, H23), 6.82 (1H, d, *J* 9 Hz, H5), 5.19 (1H, s, H18), 4.57 (1H, d, *J* 9 Hz, H12), 4.09 (1H, dd, J_1 9 Hz, J_2 4 Hz, H8), 4.00 (1H, m, H8), 3.98 (1H, m, H9), 1.47 (9H, s, H15), 0.94 (9H, H11).

δC (125 MHz, C_6D_6, 23˚C): 170.56 (C17), 156.50 (C13), 151.87 (C6), 151.42 (C24), 129.43, 129.29 (C20, C20, C22, C22), 123.88, 123.74 (C23, C23, C1), 121.14 (C4, q, *J* 4 Hz), 119.27 (C19, q, *J* 4 Hz), 117.44 (C21), 117.32 (C2, C21), 110.63 (C5), 79.91 (C14), 69.89 (C8), 58.30 (C9), 47.94 (C18), 33.61 (C10), 28.65 (C15), 27.11 (C11).

δF (470 MHz, C_6D_6, 23˚C): -62.11 (F7).

ν_{max} (neat, cm^{-1}): 3400, 2964.59, 2360.60, 2341.51, 1687.71, 1603.48, 1538.51, 1481.63, 1439.89, 1337.26, 1258.26, 1161.45, 1118.69, 1013.66, 927.60, 862.54, 800.45, 752.16, 668.99.

$[\alpha]_D^{25.0}$ + 27° (c = 0.01, $CHCl_3$).

HRMS: $C_{32}H_{35}F_3N_2NaO_5$, [requires 607.2390]; observed 607.2394.

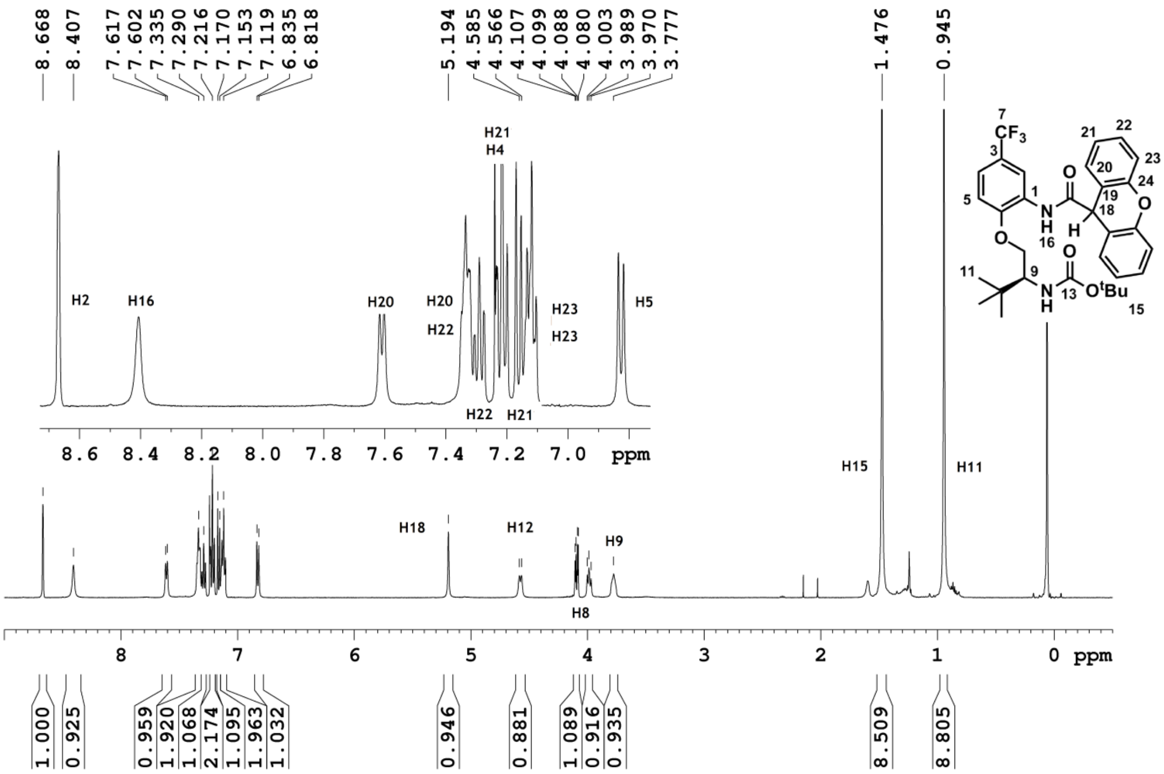

^{1}H NMR spectrum of **23** (23˚C, C_6D_6, 500 MHz).

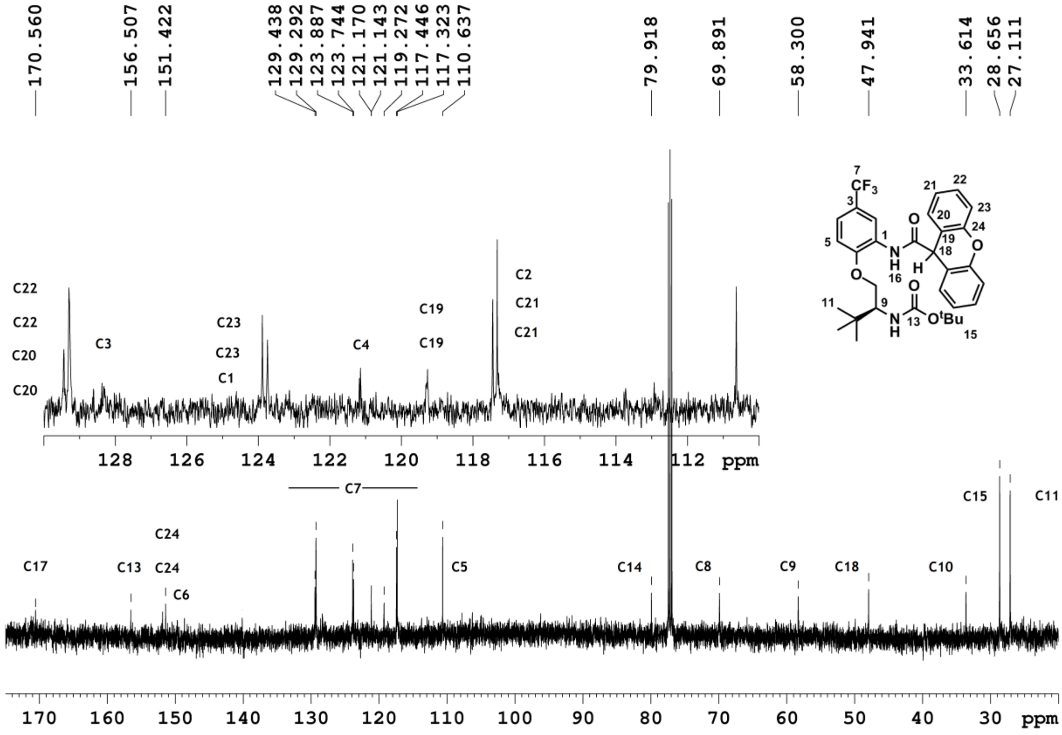

^{13}C NMR spectrum of **23** (23˚C, C_6D_6, 125 MHz).

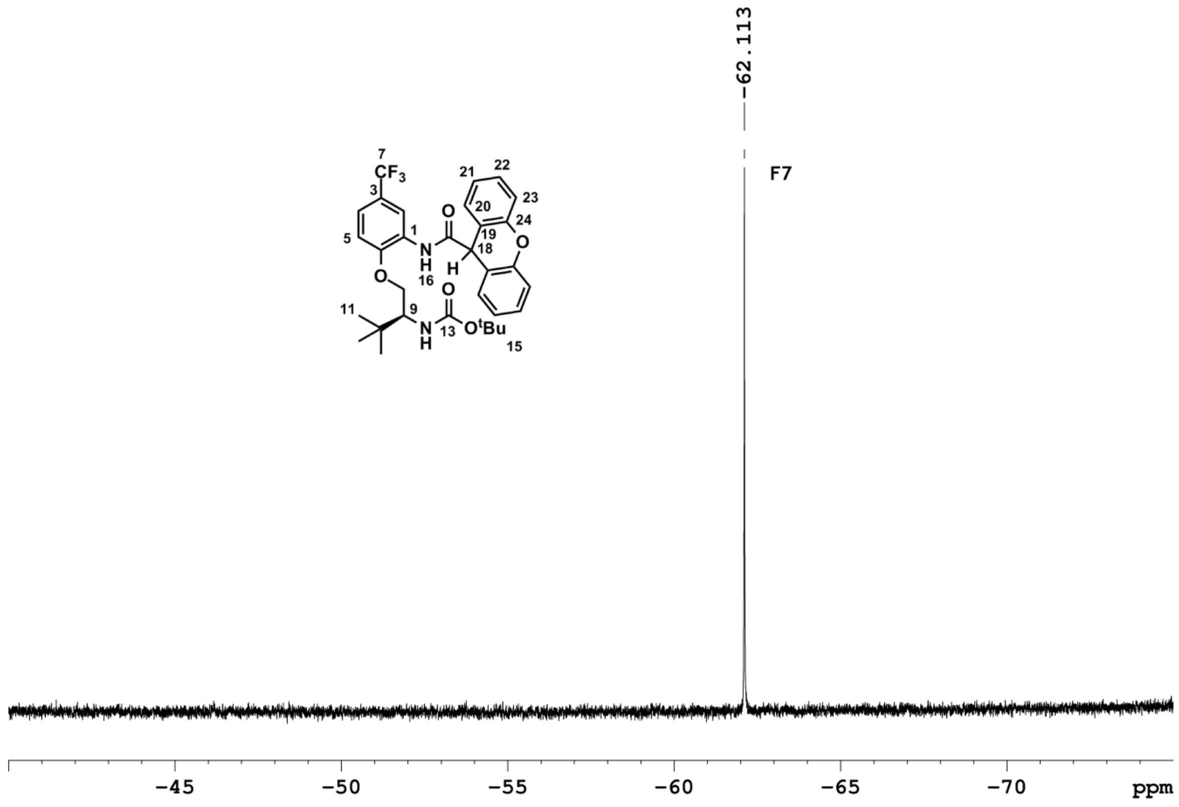

^{19}F NMR spectrum of **23** (23˚C, C_6D_6, 470 MHz).

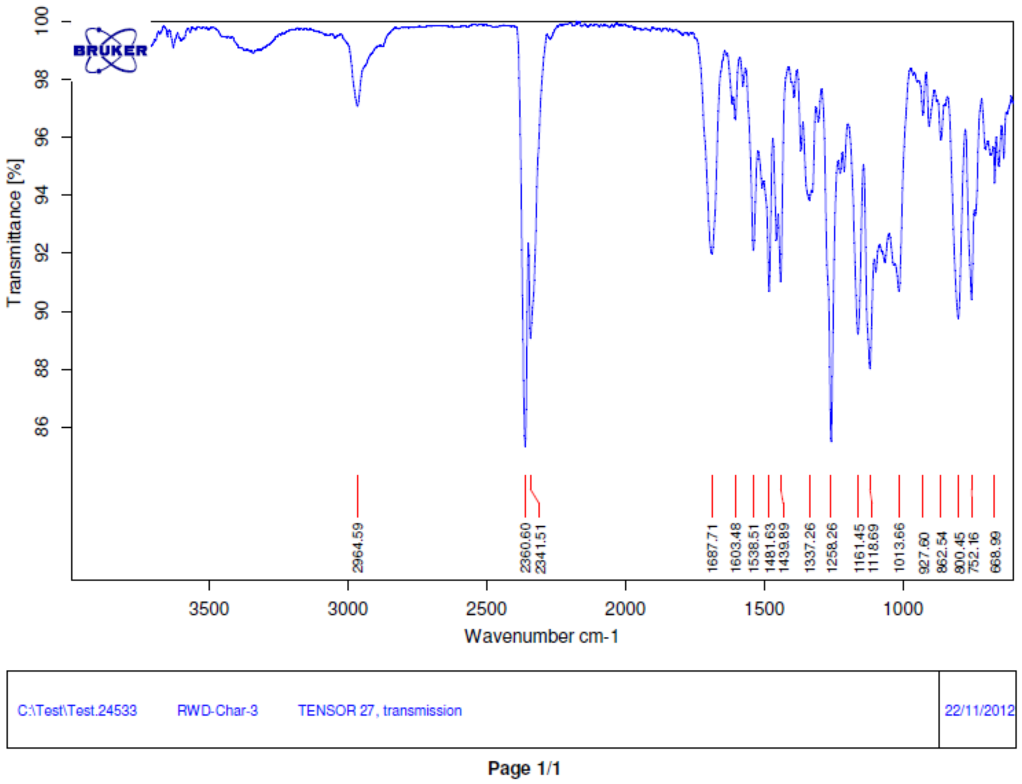

FT-IR of diffraction quality crystals of **23**.

Mass Spectrum SmartFormula Report

Analysis Info

Analysis Name	\\Utof\data\nov 12\ESI37803_18_01_738.d	Acquisition Date	23/11/2012 10:38:23
Method	2.5min_cal_sample_pos_naf_11-10-10.m	Operator	Mass Spec
Sample Name	ESI37803	Instrument / Ser#	micrOTOF 92
Comment			

Acquisition Parameter

Source Type	ESI	Ion Polarity	Positive	Set Nebulizer	2.0 Bar
Focus	Not active			Set Dry Heater	180 °C
Scan Begin	100 m/z	Set Capillary	4500 V	Set Dry Gas	10.0 l/min
Scan End	1000 m/z	Set End Plate Offset	-500 V	Set Divert Valve	Source

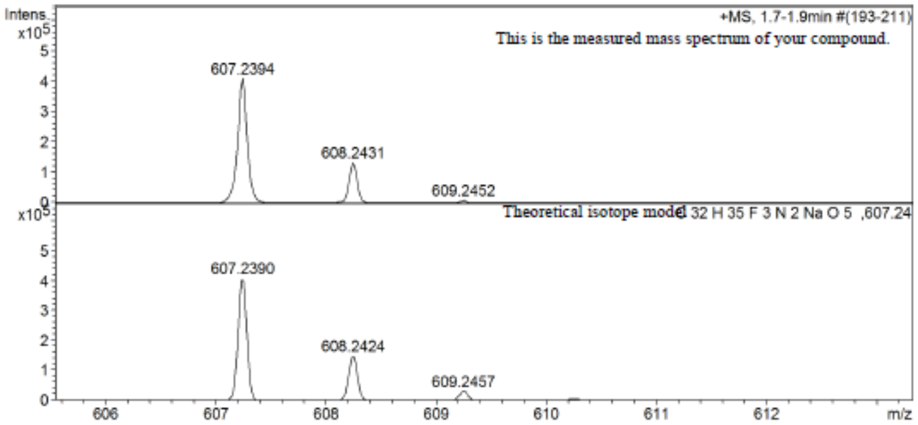

Meas. m/z	#	Formula	m/z	err [ppm]	Mean err [ppm]	rdb	e¯ Conf	mSigma
607.2394								
	1	C 32 H 35 F 3 N 2 Na O 5	607.2390	-0.6	-0.7	14.5	even	32.25

High resolution mass spectrum of **23**.

(S)-tert-butyl (1-(2-isobutyramido-4-(trifluoromethyl)phenoxy)-3,3-dimethylbutan-2-yl)carbamate [24]

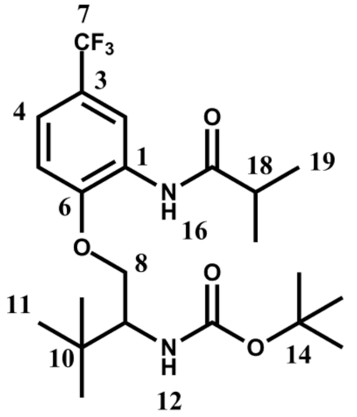

Prepared according to the representative procedure from (S)-tert-butyl (1-(2-amino-4-(trifluoromethyl)phenoxy)-3,3-dimethylbutan-2-yl)carbamate on a 0.1 mmol scale. Chromatography (silica gel, petroleum ether : ethyl acetate 6:1), 23 mg, 36% as a clear oil.

δH (700 MHz, C_6D_6, 23˚C): 9.54 (s, H2), 8.58 (H16), 7.17 (obscured by solvent signal, H4), 6.20 (1H, d, *J* 7 Hz, H5), 4.39 (1H, d, *J* 10 Hz, H12), 3.84 (1H, t, *J* 8 Hz, H9), 3.61 (1H, dd, J_1 8 Hz J_2 2 Hz, H8), 3.24 (1H, apparent triplet, J 9 Hz, H8), 2.91 (1H, sept, J 7 Hz, H18), 1.40 (9H, s, H15), 1.31 (3H, d, *J* 7 Hz, H19), 1.29 (3H, d, *J* 7 Hz, H19), 0.61 (9H, s, H11).

δC (175 MHz, C_6D_6, 23˚C): 175.85 (C17), 156.88 (C13), 149.69 (C6), 129.98 (C1), 125.38 (C7, q, $^1J_{13C-19F}$ 270 Hz), 123.88 (C3, q, $^2J_{13C-19F}$ 32 Hz), 120.02 (C4, q, $^3J_{13C-19F}$ 4 Hz), 117.19 (C2, m), 110.25 (C5), 79.35 (s, C14), 69.83 (C8), 58.74 (C9), 36.75 (C18), 32.72 (C10), 28.43 (C15), 26.57 (C11), 20.39 (C19), 19.62 (C19).

δF (376 MHz, $CDCl_3$, 23˚C): -61.65.

HRMS: (ES+Na): $C_{22}H_{33}F_3N_2NaO_4$, found (469.2265);, [M + Na] requires 469.2285.

ν_{max} (neat, cm^{-1}): 3336.49, 2965.91, 2931.03, 2874.33, 2360.59, 2341.97, 1681.46, 1614.67, 1603.50, 1538.06, 1489.35, 1459.28, 1438.90, 1391.07, 1366.79, 1337.44, 1323.05, 1268.87, 1214.47, 115.96, 1118.99, 1062.44, 1040.93, 1011.90, 942.47, 920.95, 898.76, 859.97, 810.46, 735.32, 654.13, 635.63.

MP: oil.

$[\alpha]_D^{25.0}$ +25.0 (c = 0.001, $CHCl_3$) .

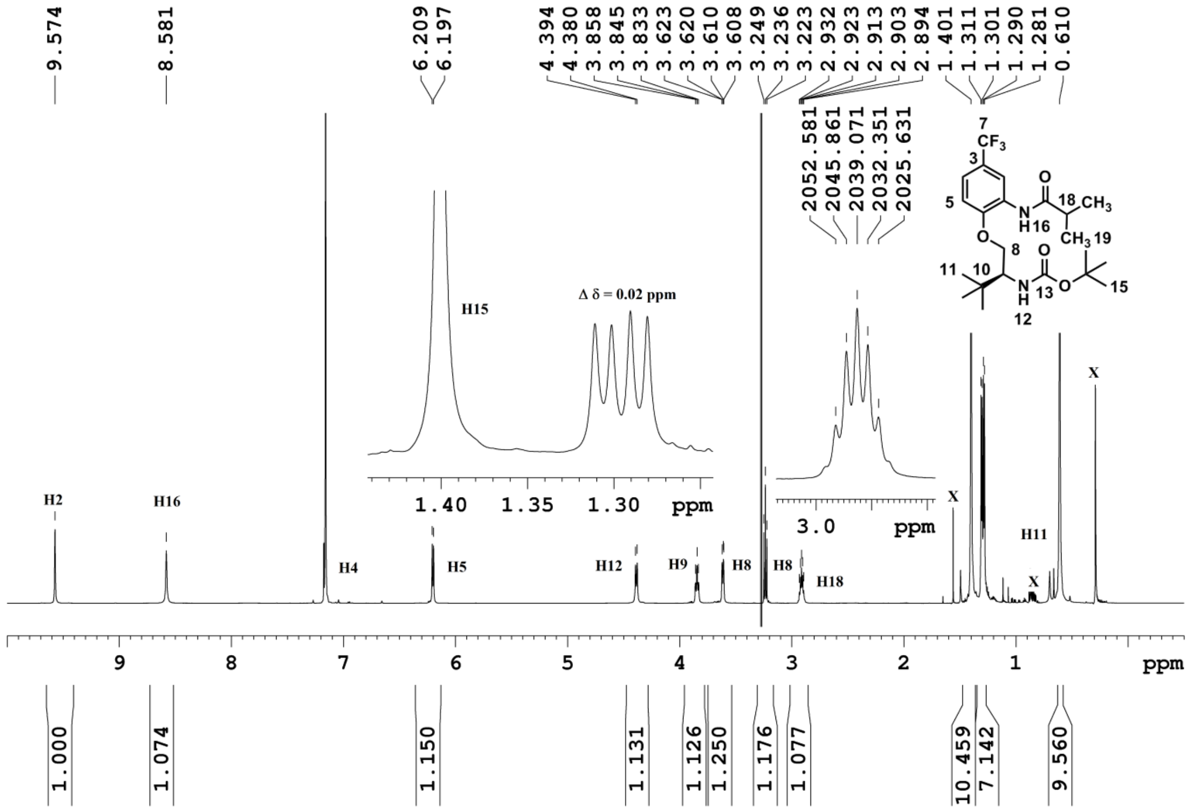

^{1}H NMR spectrum of **24** (23˚C, C_6D_6, 700 MHz).

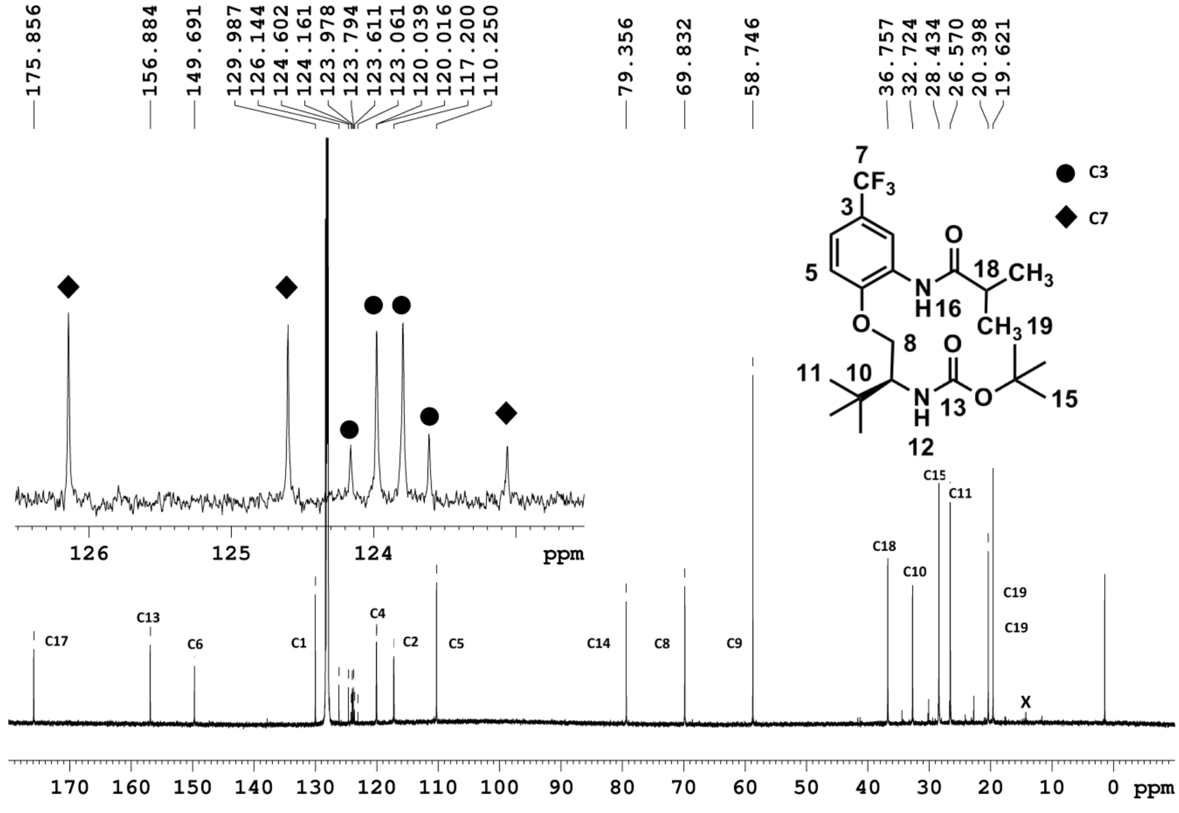

^{13}C NMR spectrum of **24** (23˚C, C_6D_6, 175 MHz).

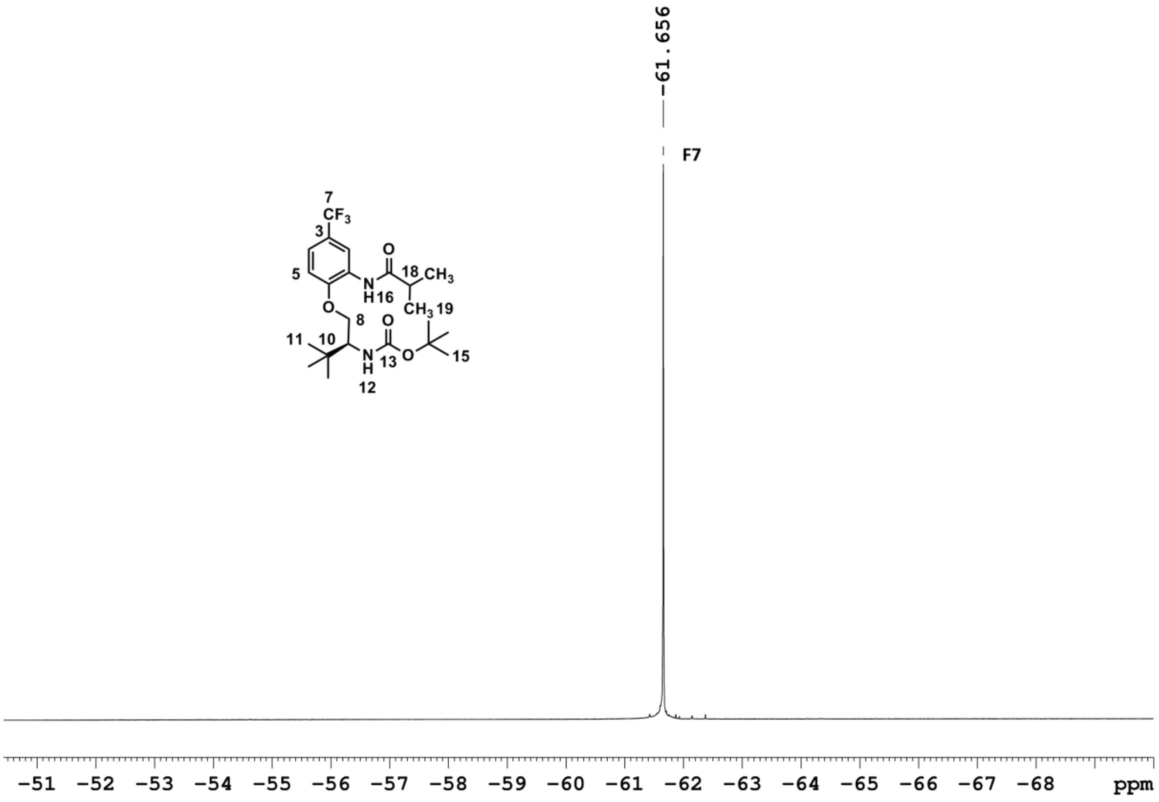

^{19}F NMR spectrum of **24** (23˚C, $CDCl_3$, 376 MHz).

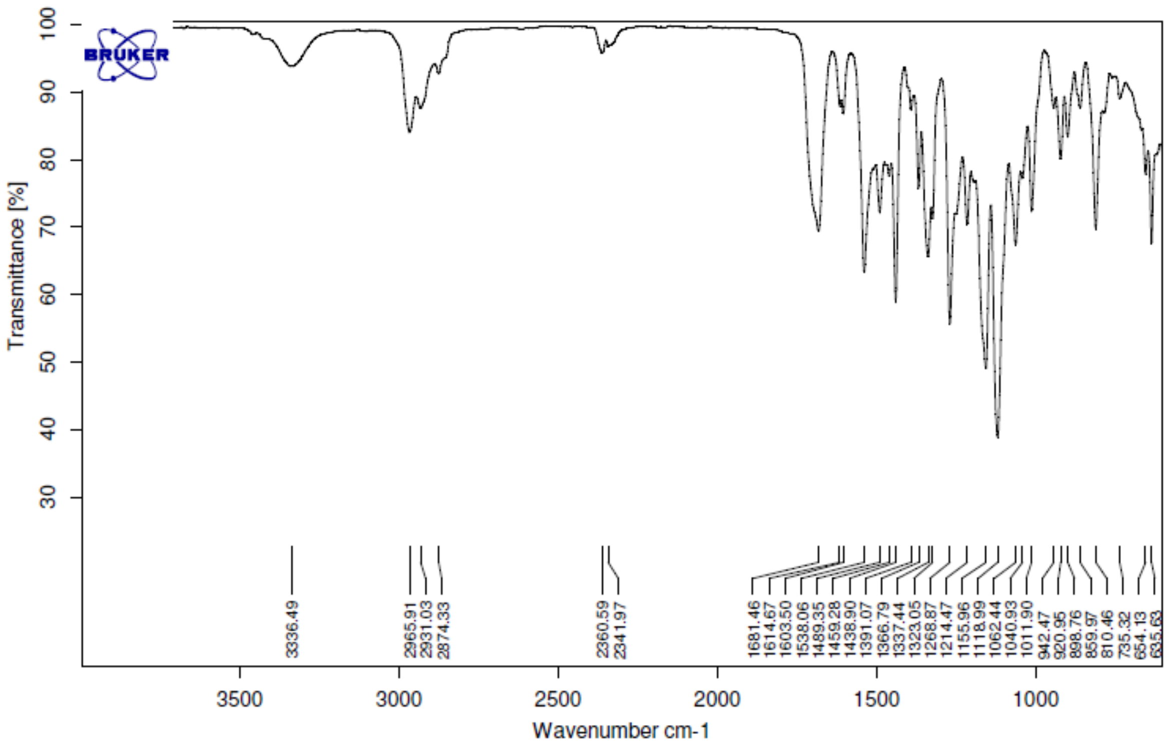

FT-IR spectrum of **24**.

Mass Spectrum SmartFormula Report

**Analysis Info**			
Analysis Name	\\Utof\data\nov 12\ESI37804_19_01_739.d	Acquisition Date	23/11/2012 10:42:03
Method	2.5min_cal_sample_pos_naf_11-10-10.m	Operator	Mass Spec
Sample Name	ESI37804	Instrument / Ser#	micrOTOF 92
Comment			

**Acquisition Parameter**					
Source Type	ESI	Ion Polarity	Positive	Set Nebulizer	2.0 Bar
Focus	Not active			Set Dry Heater	180 °C
Scan Begin	100 m/z	Set Capillary	4500 V	Set Dry Gas	10.0 l/min
Scan End	1000 m/z	Set End Plate Offset	-500 V	Set Divert Valve	Source

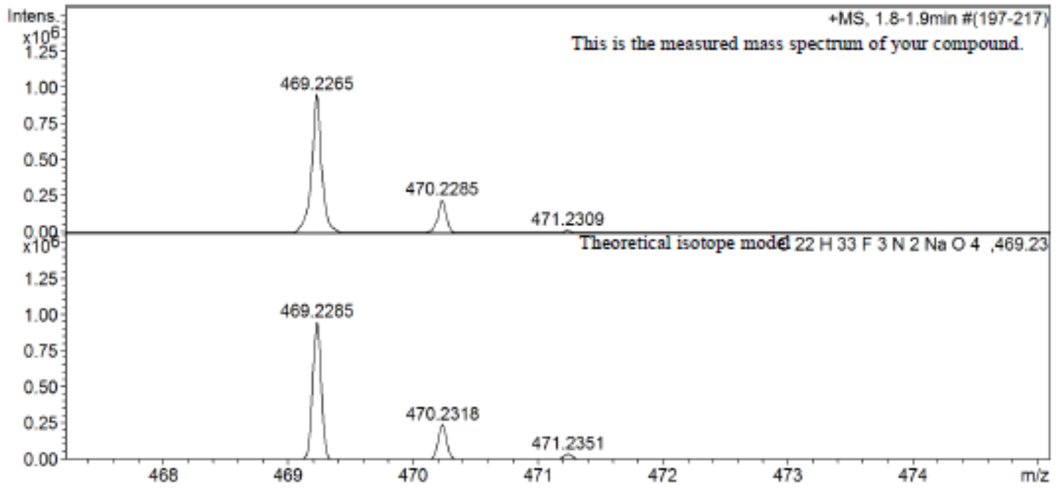

Meas. m/z	#	Formula	m/z	err [ppm]	Mean err [ppm]	rdb	e⁻ Conf	mSigma
469.2265								
	1	C 22 H 33 F 3 N 2 Na O 4	469.2285	4.2	4.8	5.5	even	14.53

High-Resolution Mass Spectrum of **24**.

3,3,3-trifluoro-N-(2-methoxy-5-(trifluoromethyl)phenyl)-2-(trifluoromethyl)propanamide [25]

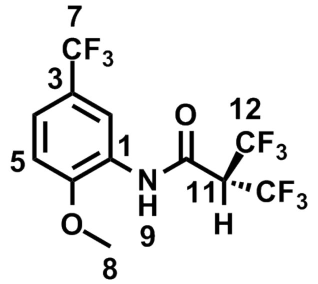

Prepared according to the representative procedure on a 0.2 mmol scale from 2-methoxy-5-(trifluoromethyl)aniline. Chromatography (silica gel, petroleum ether : ethyl acetate 10:1), followed by crystallization in EtOAc/Pentanes, 17 mg, 23% as a white solid.

δH (500 MHz, C_6D_6, 23 ˚C): 9.02 (1H, d, *J* 2 Hz, H2), 7.76 (bs, H9), 7.18 (1H, dd, J_1 8 Hz, J_2 2 Hz, H4), 6.03 (1H, d, *J* 9 Hz, H5), 2.96 (3H, s, H8), 2.69 (1H, sept, *J* 8Hz, H11).

δC (125 MHz, $CDCl_3$, 23 ˚C): 155.653 (C10), 150.405 (C6), 126.59 (C1), 124.11 (C3, quartet, $^2J_{13C\text{-}19F}$ = 32 Hz), 124.07 (C7, quartet, $^1J_{13C\text{-}19F}$ = 271 Hz), 122.94 (C4), 117.55 (C2), 110.12 (C5), 121.60 (C12, quartet, $^1J_{13C\text{-}19F}$ = 283 Hz), 56.84 (C11, septet, $^2J_{13C\text{-}19F}$ = 31 Hz), 56.62 (C8).

δF (470 MHz, C_6D_6, 23˚C): - 61.50 (F7), -64.32 (F19, d, $^2J_{19F\text{-}13C}$ = 7 Hz).

HRMS: (ES-): found 392.0292; Formula $C_{12}H_8F_9NO_2Na$, [M + Na] requires 392.0304.

ν_{max} (neat, cm^{-1}): 3355.06, 2968.86, 1687.62, 1620.40, 1545.53, 1499.53, 1444.45, 1365.95, 1342.25, 1323.73, 1278.16, 1238.17, 1180.53, 1117.18, 1095.30, 1027.03, 995.02, 920.13, 893.77, 867.04, 833.33, 818.15, 752.71, 721.49, 663.73, 616.24.

MP: 166-168˚C.

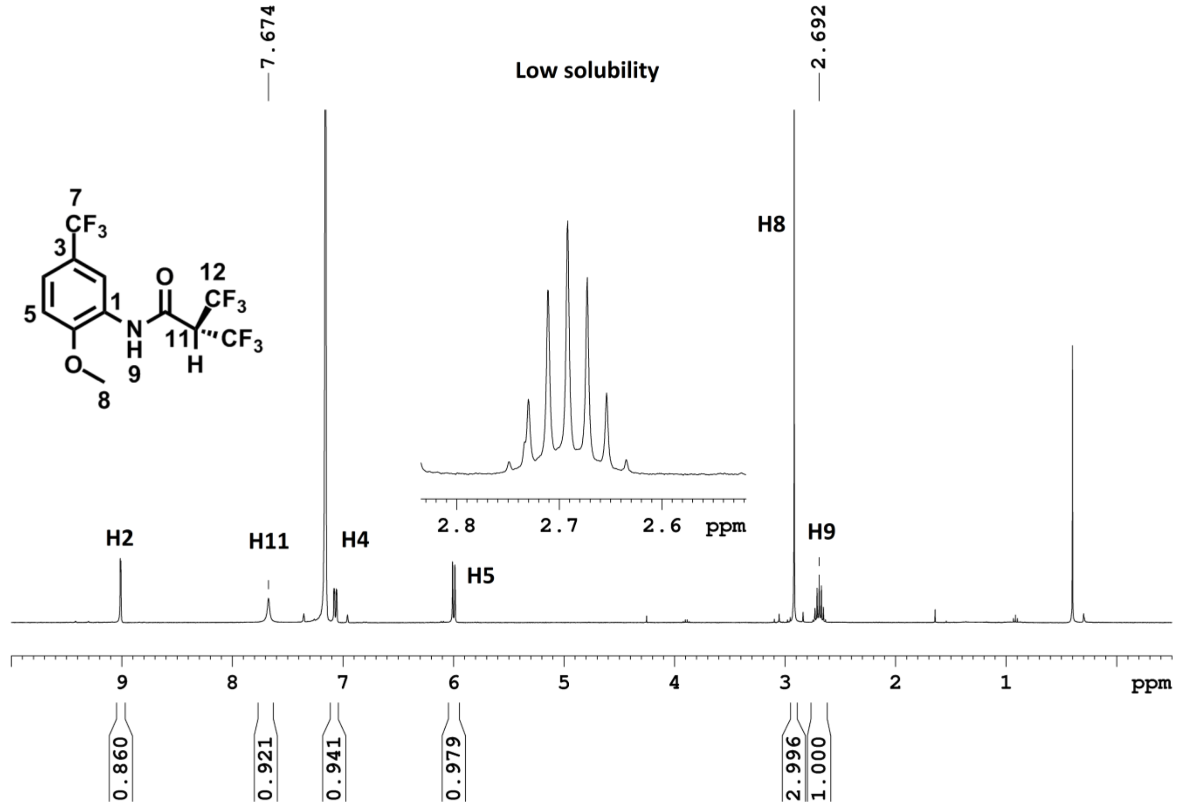

^{1}H NMR spectrum of **25** (23˚C, C_6D_6, 500 MHz).

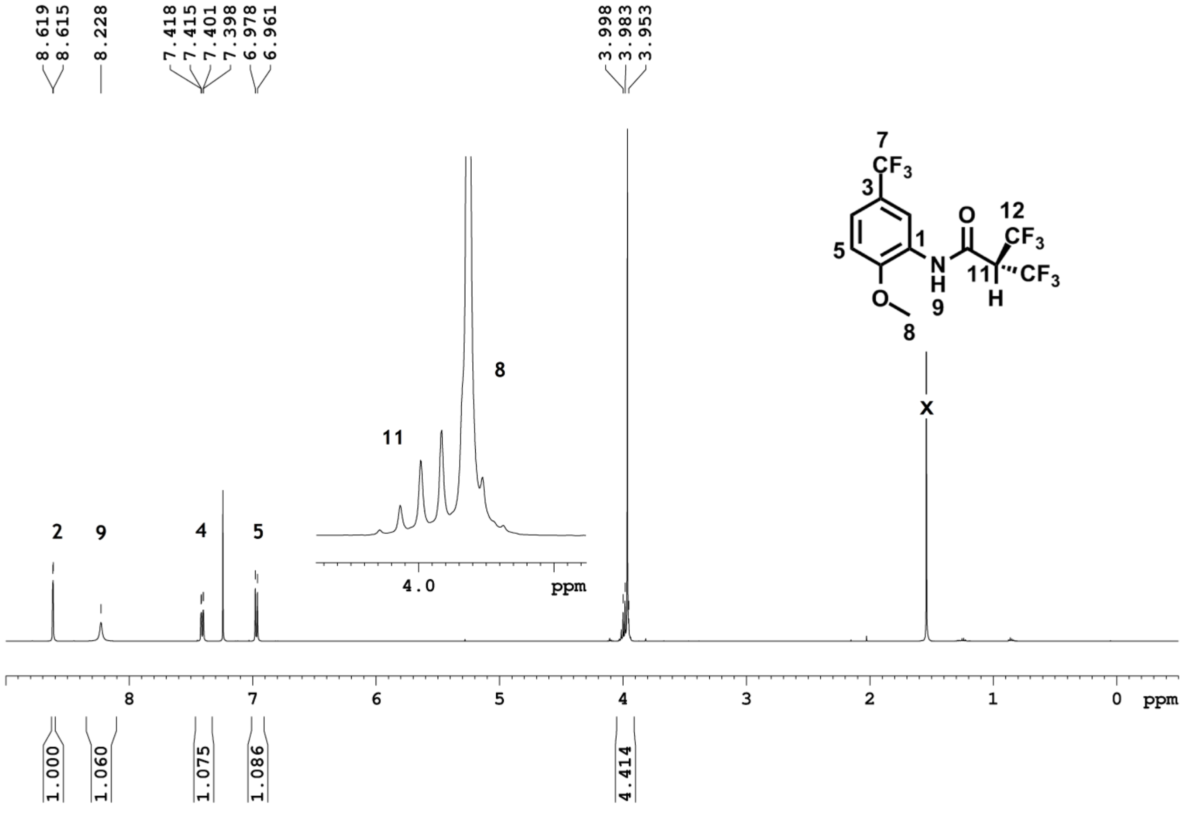

^{13}C NMR spectrum of **25** (23˚C, $CDCl_3$, 125 MHz).

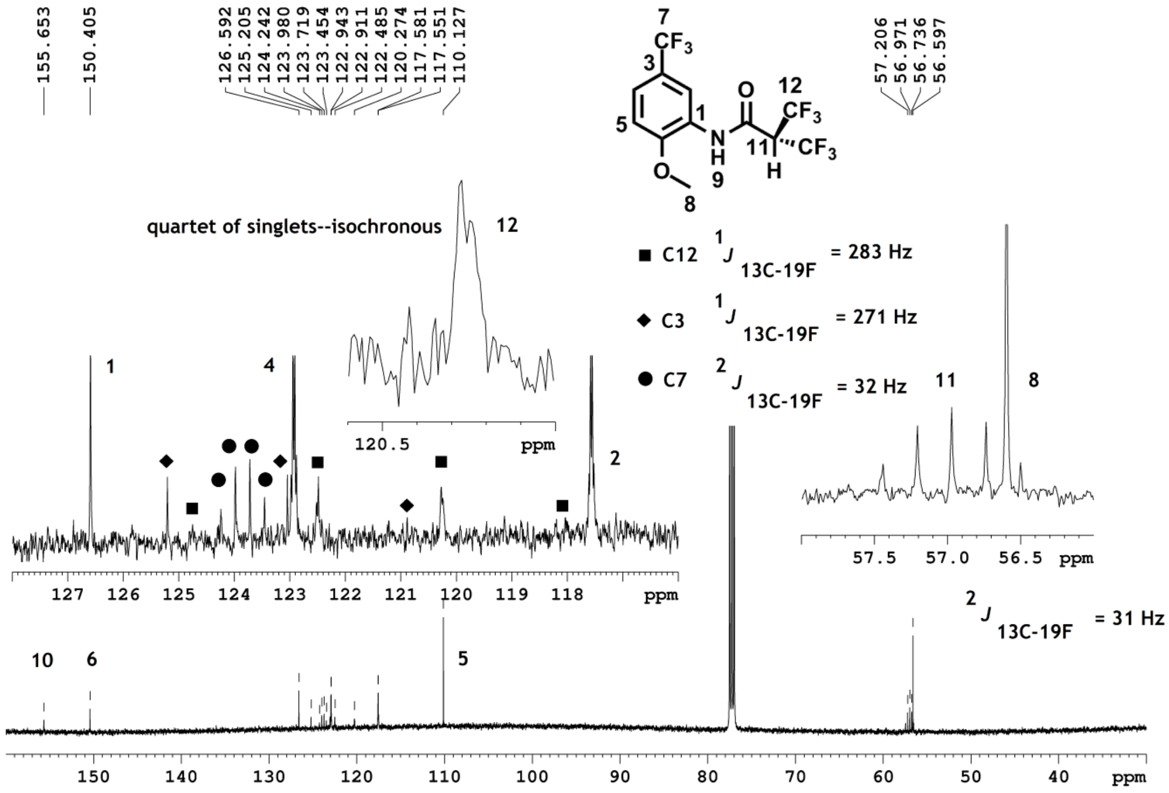

^{13}C NMR spectrum of **25** (23˚C, $CDCl_3$, 125 MHz) instrument showing a quartet splitting pattern for both isochronous CF_3 carbon atoms.

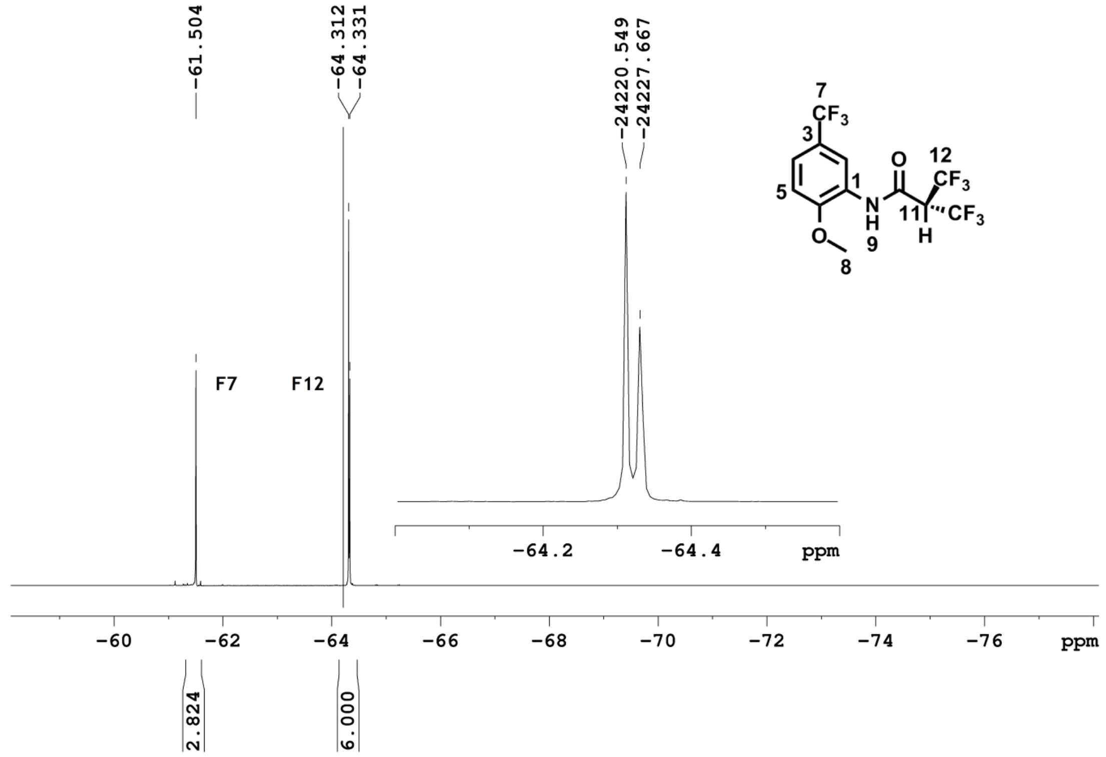

^{19}F NMR spectrum of **10** (23˚C, C_6D_6, 470 MHz) showing a double splitting pattern as both sets of fluorine atoms are equivalent.

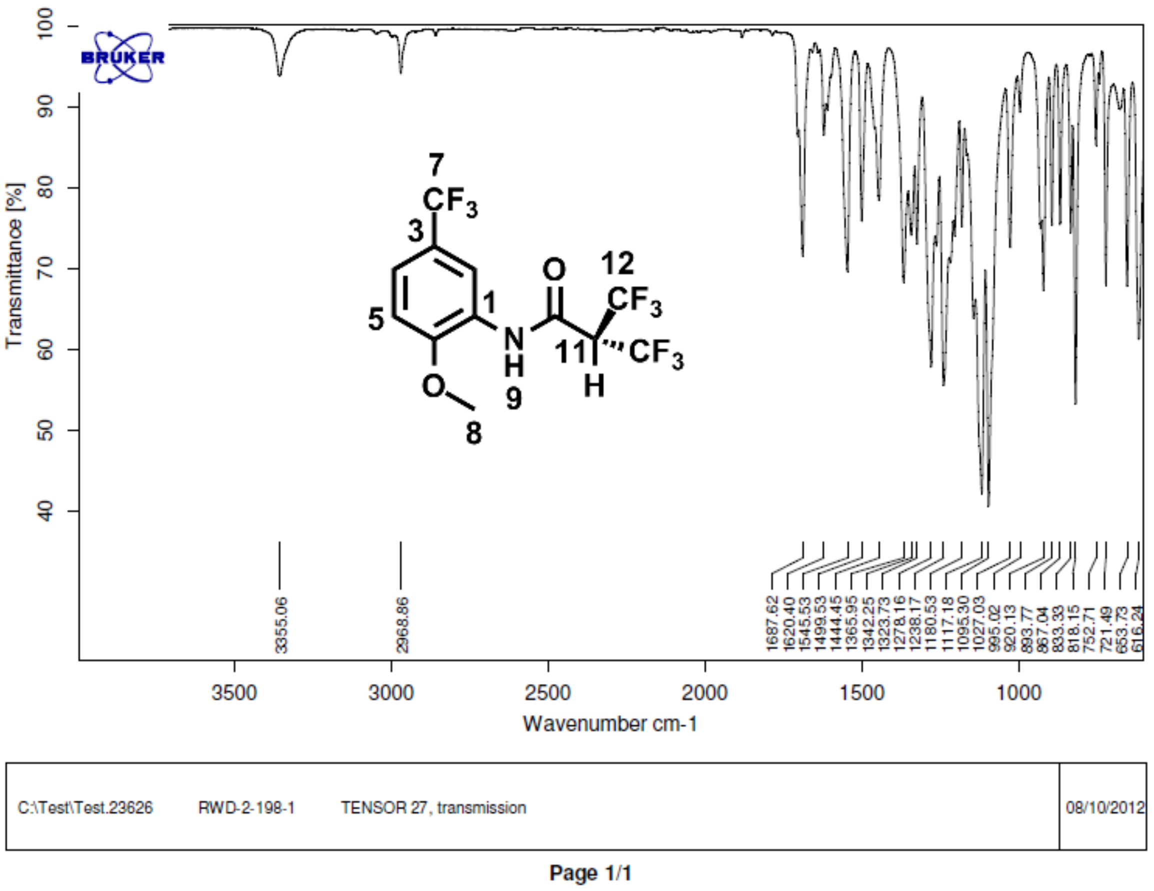

FT-IR of diffraction quality crystals of **25**.

Mass Spectrum SmartFormula Report

**Analysis Info**		Acquisition Date	10/10/2012 09:06:52
Analysis Name	\\Utof\Data\Oct 12\ESI37259_6_01_48241.d		
Method	2.5min_cal_sample_pos_Naf_11-10-10.m	Operator	Mass Spec
Sample Name	ESI37259	Instrument / Ser#	micrOTOF 92
Comment			

Acquisition Parameter

Source Type	ESI	Ion Polarity	Positive	Set Nebulizer	2.0 Bar
Focus	Not active			Set Dry Heater	180 °C
Scan Begin	100 m/z	Set Capillary	4500 V	Set Dry Gas	10.0 l/min
Scan End	1000 m/z	Set End Plate Offset	-500 V	Set Divert Valve	Source

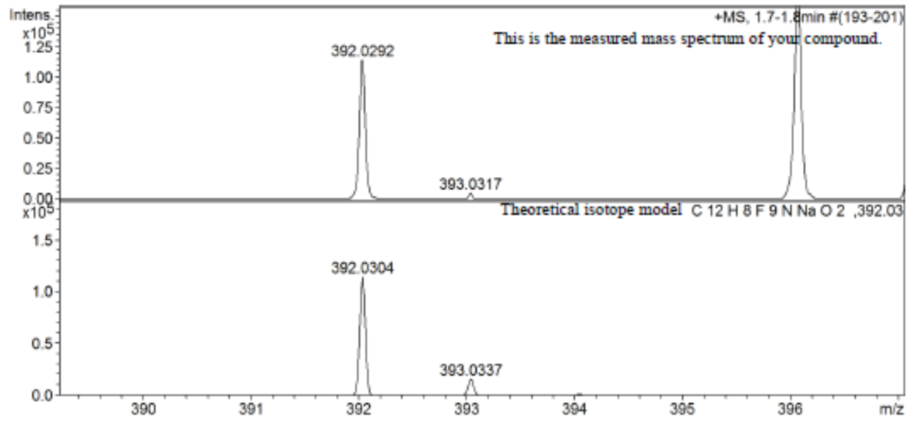

Meas. m/z	#	Formula	m/z	err [ppm]	Mean err [ppm]	rdb	e⁻ Conf	mSigma
392.0292								
	1	C 12 H 8 F 9 N Na O 2	392.0304	3.0	3.1	4.5	even	53.35

High resolution mass spectrum of **25**.

3,3,3-trifluoro-N-(2-fluoro-5-(trifluoromethyl)phenyl)-2-(trifluoromethyl)propanamide (25-F)

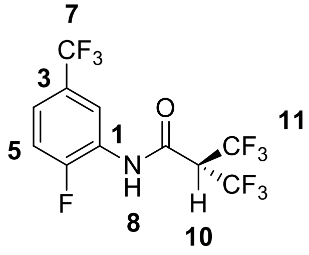

10% Pd/C (75 mg) was added to a stirring solution of 4-fluoro-3-nitrobenzotrifluoride (0.5 g, 1.2 mmol) and ammonium formate (750 mg, 11.9 mmol) in MeOH (20 mL) and ethyl acetate (3 mL) under nitrogen. The yellow solution became clear instantly. The reaction was stirred for 20 minutes, then the palladium removed by filtration. The solvent was removed *in vacuo* and the resulting white solid dissolved in ethyl acetate (200mL). The organic phase was washed with sat. aqueous ammonium formate (50 mL X 2), sat. sodium carbonate (50mL X 2) and brine (50mL X 2). The organic phase was dried with $MgSO_4$, then concentrated to yield 450 mg (1.2 mmol, 100%) of the 4-fluoro-3-benzotrifluoride aniline intermediate.

EDCI·HCl (365mg, 2.0 mmol) added in one portion to a stirring solution of 3,3,3 trifluoro-2-(trifluoromethyl) propionic acid (0.461g, 2.0 mmol), pyridine (1.0 mL) and N,N-dimethylaminopyridine (7 mg, 0.05 mmol) in DCM (30mL) at 0˚C under an atmosphere of nitrogen. After 20 min, the aniline intermediate (200 mg, 1.0 mmol) in DCM (5 mL) was added dropwise. The reaction was allowed to stir overnight, slowly warming to room temperature then quenched with water (20 mL). The reaction was diluted with ethyl acetate (400 mL), then washed with aqueous saturated ammonium chloride solution (1 X 200 mL), aqueous sodium carbonate solution (1 X 200 mL), then saturated brine solution (1 X 100 mL). The organic phase was dried with $MgSO_4$ and filtered to yield a white solid. Compound purified by FC 8:1 PET:EtOAc. The resulting white solid was dissolved in pentane/ethyl acetate (15:1) and colorless needle crystals grown for X-ray studies (192 mg, 54% after recrystallization).

δH (500 MHz, $CDCl_3$, 23˚C): 8.61 (1H, dd, J_1 7 Hz, J_2 1.5 Hz, H2), 7.90 (1H, s, H8), 7.45 (1H, m, H4), 7.30 (1H, d, *J* 9 Hz, H5), 4.06 (1H, septet, $^3J_{1H\text{-}19F}$ 7.0 Hz, H10).

δH (500 MHz, C_6D_6, 23˚C, low solubility): 8.76 (1H, dd, J_1 8 Hz, J_2 2 Hz, H2), 7.07 (1H, s, H8), 6.91 (m, H4), 6.47 (apparent triplet, *J* 10 Hz, H5), 2.67 (septet, *J* 7Hz, H10).

δC (125 MHz, C_6D_6, 23˚C, low solubility): 156.16 (s, C8), 154.46 (d, $^1J_{13C\text{-}19F}$ 150 Hz, C6), 127.97 (dq, $^2J_{13C\text{-}19F}$ 36 Hz, $^4J_{13C\text{-}19F}$ 6 Hz, C3), 125.88 (d, $^2J_{13C\text{-}19F}$ 9 Hz, C1), 124.30 (quartet of doublets, $^1J_{13C\text{-}19F}$ 282 Hz, $^5J_{13C\text{-}19F}$ 3 Hz, C7), 124.26 (quartet, $^1J_{13C\text{-}19F}$ 272 Hz, C10), 123.65 (m, C4), 120.18 (d, $^3J_{13C\text{-}19F}$ 2.5 Hz, C2), 115.93 (d, $^2J_{13C\text{-}19F}$ 21 Hz, C5), 55.16 (pentet, $^3J_{13C\text{-}19F}$ 9 Hz, C9).

δF (235 MHz, $CDCl_3$, 23˚C): -62.03 (F7), -64.18 (d, $^3J_{19F\text{-}1H}$ 7 Hz, F10), -124.68 (F6).

ν_{max} (neat, cm^{-1}): 3337.33, 2965.41, 1681.05, 1628.72, 1535.43, 1467.80, 1367.33, 1325.09, 1292.56, 1277.97, 1247.94, 1159.31, 1126.82, 1093.61, 1063.61, 1035.27, 1011.86, 999.26, 940.05, 895.86, 863.62, 836.54, 785.38, 758.87, 685.43, 633.52.

HRMS: (ES+): $C_{11}H_4F_{10}NO$ [requires 356.0139]; observed 356.0143.

MP:124-126˚C.

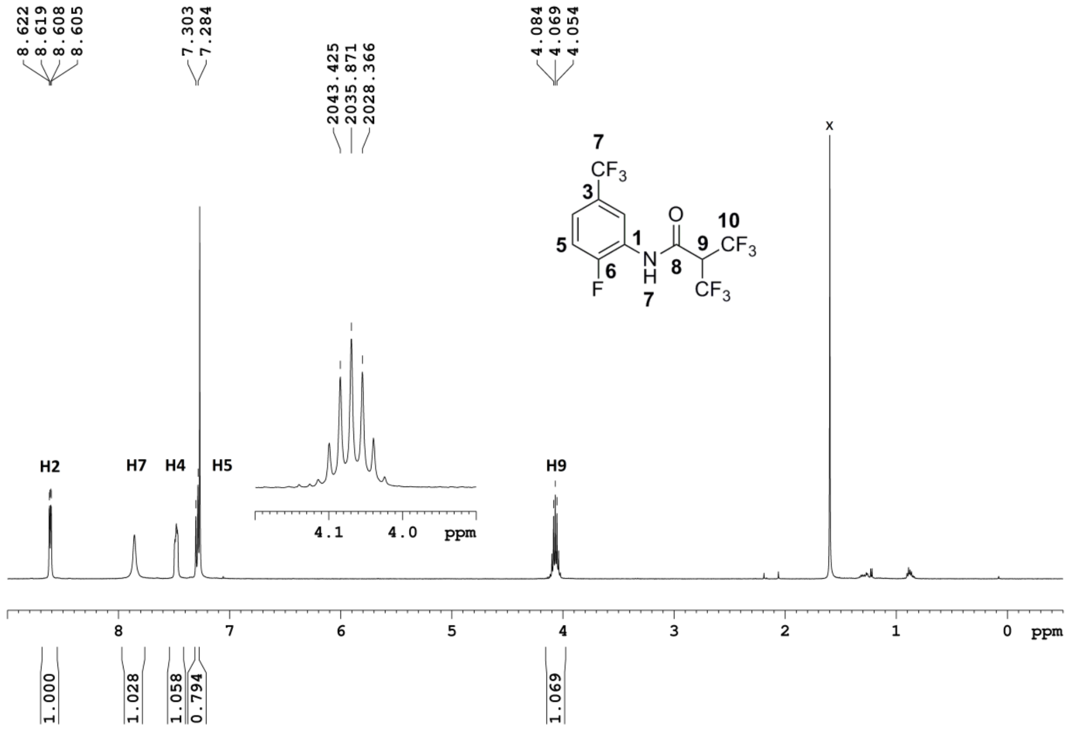

^{1}H NMR spectrum of **25-F** (23˚C, $CDCl_3$, 500 MHz).

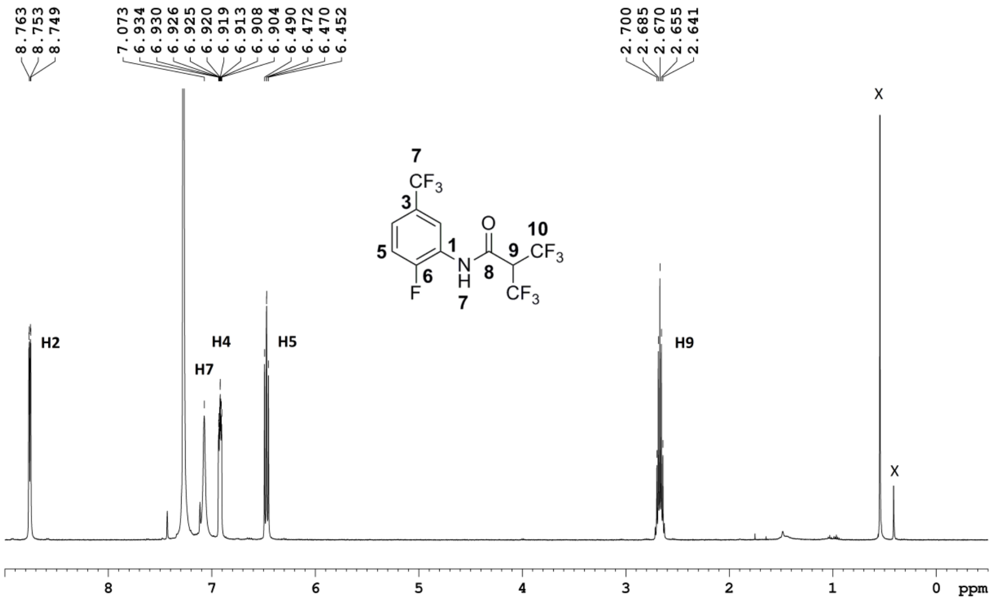

^{1}H NMR spectrum of **25-F** (23˚C, C_6D_6, 500 MHz). [low solubility].

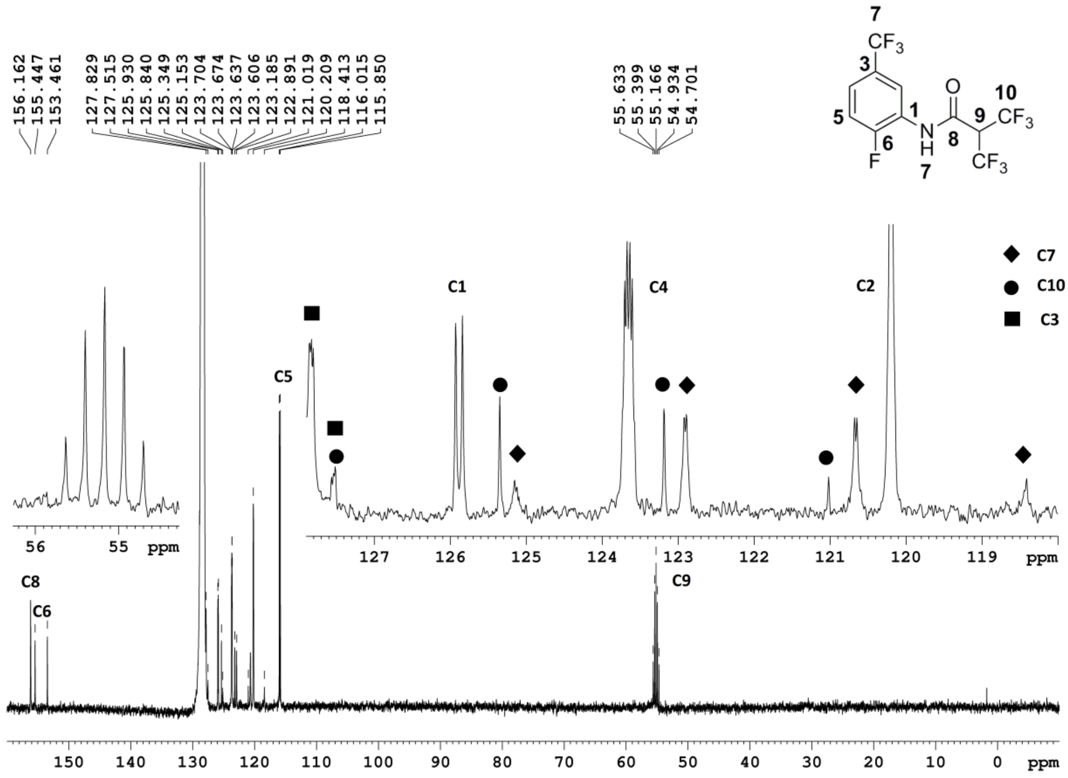

^{13}C NMR spectrum of **25-F** (C_6D_6, 23˚C, 125 MHz).

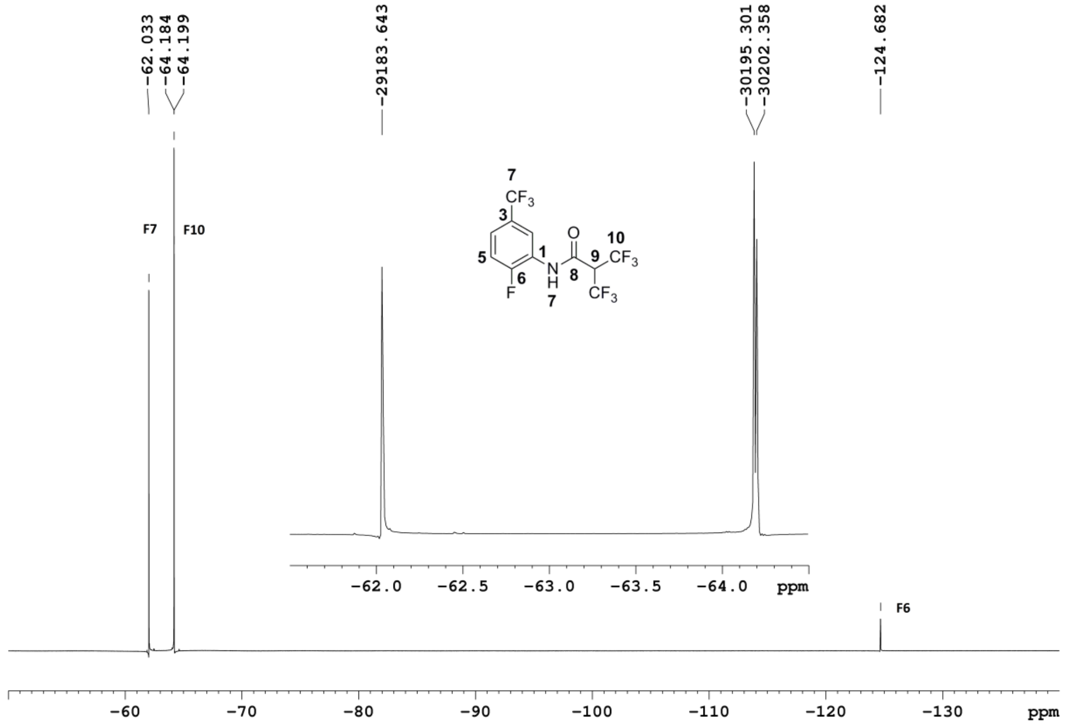

^{19}F NMR spectrum of **25-F** (C_6D_6, 23˚C, 470 MHz).

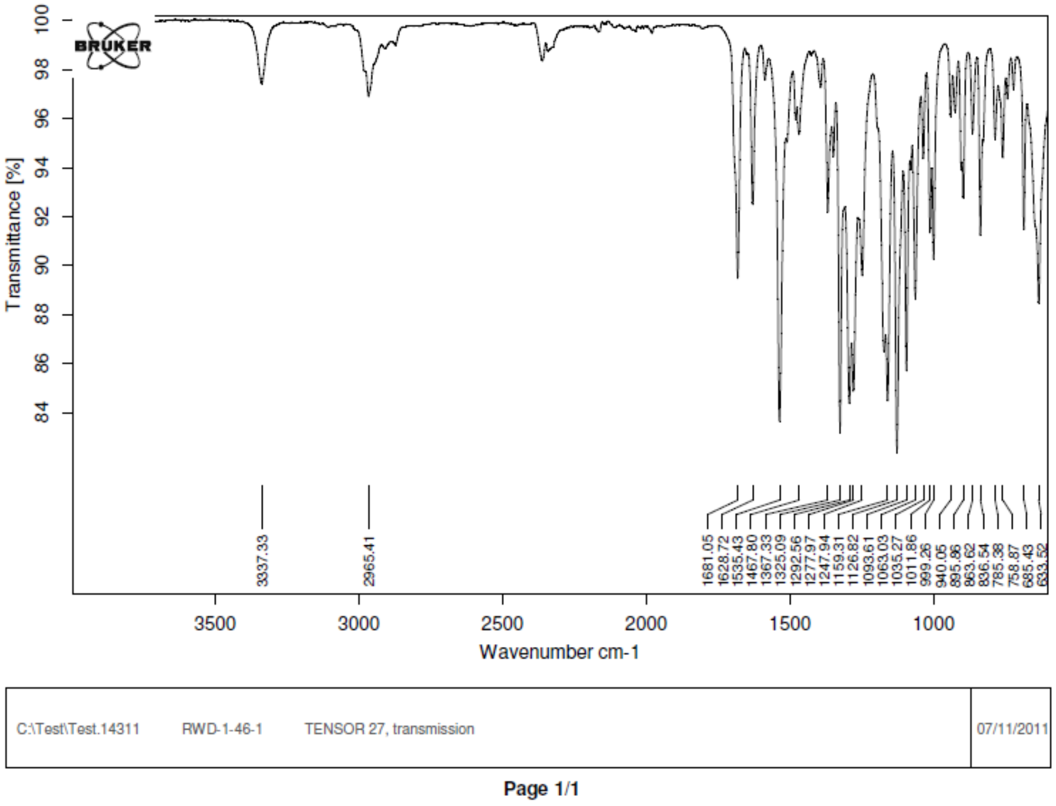

FT-IR of diffraction quality crystals of **25-F**.

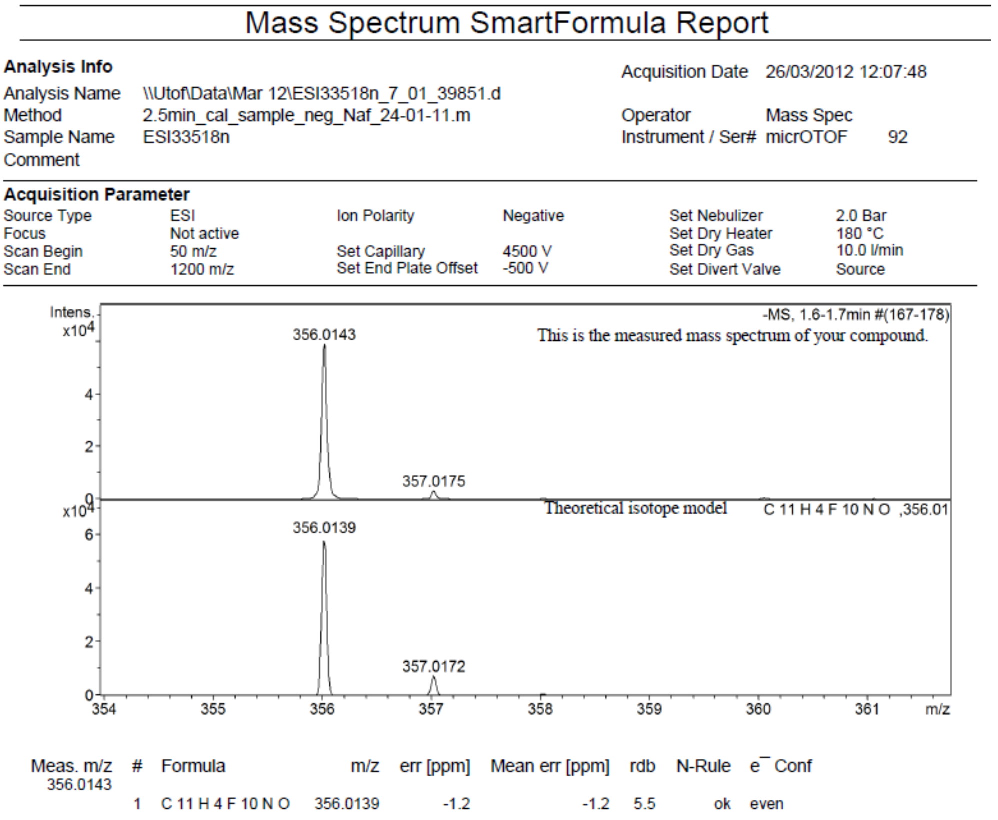

High resolution mass spectrum of **25-F**.

N-(2-methoxy-5-(trifluoromethyl)phenyl)cyclopropanecarboxamide [26]

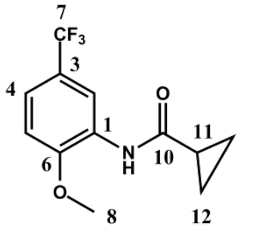

Prepared according to the representative procedure from 2-methoxy-5-(trifluoromethyl)aniline on a 0.28 mmol scale. Chromatography (3:1 PET:EtOAc) to yield a white precipitate (45 mg, 62%) for X-ray crystallographic and NMR studies.

δH (500 MHz, C_6D_6, 23˚C): 9.34 (1H, s, H2), 7.54 (1H, s, H9), 7.10 (1H, dd, J_1 8 Hz, J_2 2 Hz, H4), 6.19 (1H, d, J 8 Hz, H5), 3.10 (3H, s, H8), 1.05 (2H, m, H12), 0.66 (1H, m, H11), 0.35 (1H, m, H12).

δC (125 MHz, C_6D_6, 23˚C): 171.60 (C10), 149.93 (C6), 129.48 (C1), 125.65 (q, $^{1}J_{13C\text{-}19F}$ 270 Hz, C7), 123.45 (q, $^{2}J_{13C\text{-}19F}$ 32 Hz, C3), 120.56 (q, $^{3}J_{13C\text{-}19F}$ 4 Hz, C4), 117.23 (q, $^{3}J_{13C\text{-}19F}$ 3 Hz, C2), 109.97 (C5), 55.56 (C8), 16.11 (C11), 8.46 (C12).

HRMS: (ES-): 282.0712; Formula $C_{12}H_{12}F_3NO_2Na$, [M + Na] requires 282.0712.

ν_{max} (neat, cm^{-1}): 3319.59, 1666.87, 1604.09, 1539.54, 1490.79, 1431.12, 1397.46, 1350.01, 1320.84, 1265.76, 1224.71, 1196.59, 1179.20, 1159.90, 1112.98, 1078.80, 1056.68, 1029.02, 955.58, 924.51, 891.94, 872.42, 806.28, 776.12.

MP: 119˚C.

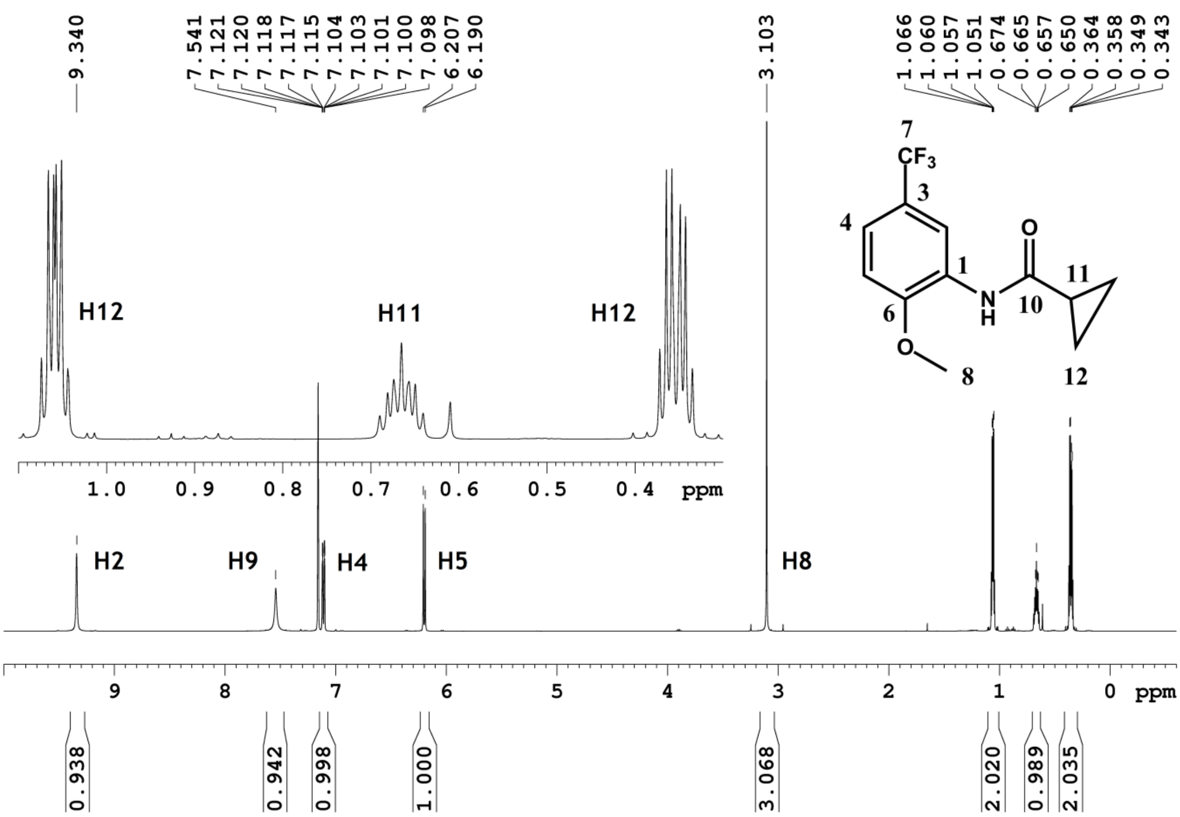

^{1}H NMR spectrum of **26** (23˚C, C_6D_6, 500 MHz).

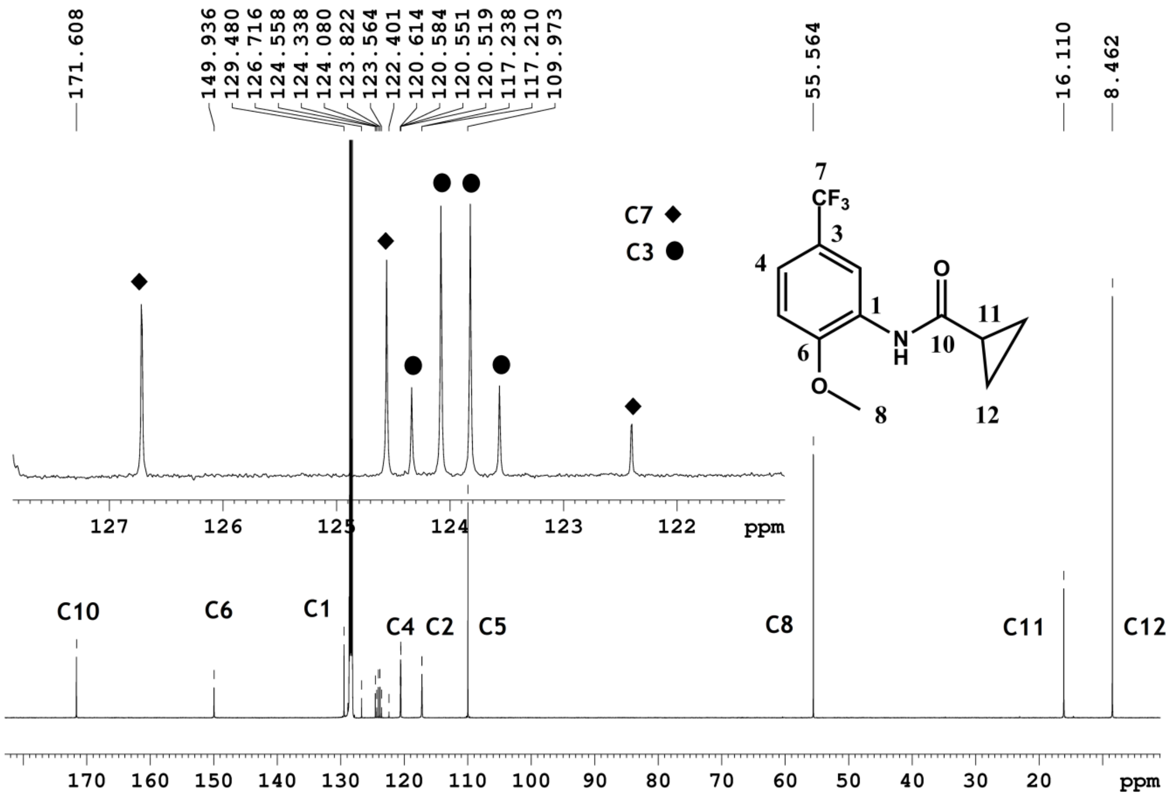

^{13}C NMR spectrum of **26** (C_6D_6, 23˚C, 125 MHz).

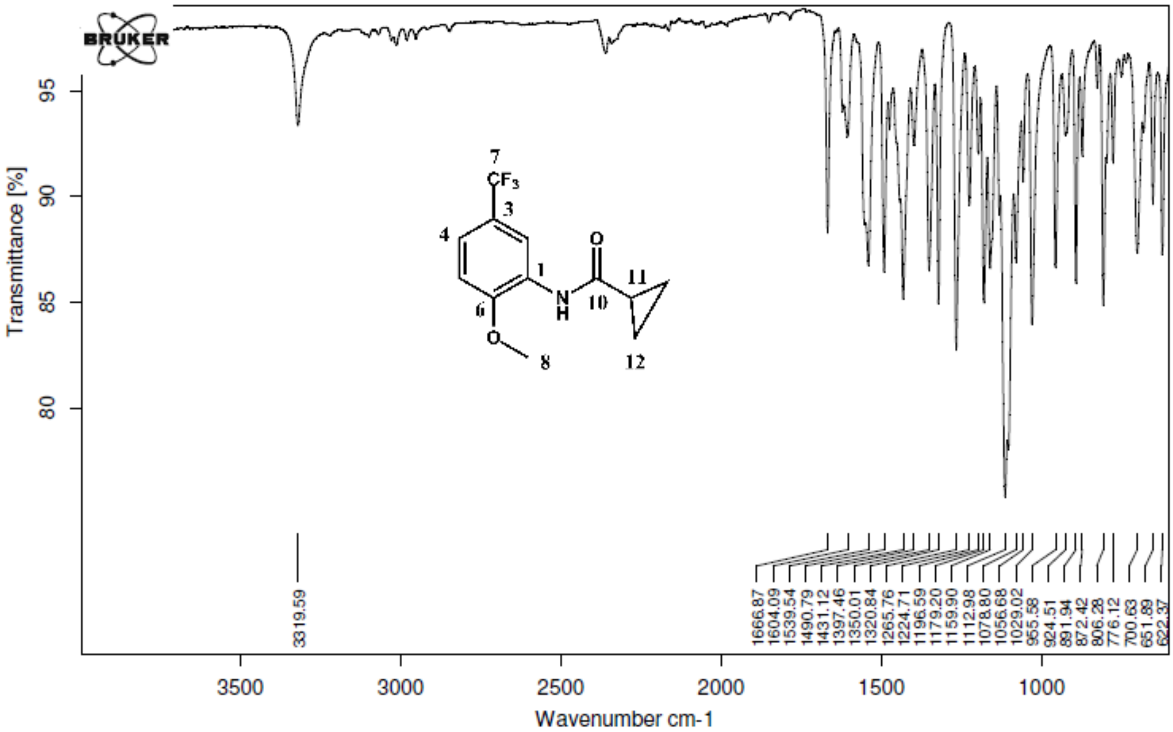

FT-IR of **26**.

Mass Spectrum SmartFormula Report

**Analysis Info**		Acquisition Date	17/12/2013 08:26:56
Analysis Name	\\Utof\data\Dec 13\ESI43957_12_01_15068.d		
Method	2.5min_cal_sample_pos_naf_05-08-13.m	Operator	Mass Spec
Sample Name	ESI43957	Instrument / Ser#	micrOTOF 92
Comment			

**Acquisition Parameter**					
Source Type	ESI	Ion Polarity	Positive	Set Nebulizer	2.0 Bar
Focus	Not active			Set Dry Heater	180 °C
Scan Begin	100 m/z	Set Capillary	4500 V	Set Dry Gas	10.0 l/min
Scan End	1000 m/z	Set End Plate Offset	-500 V	Set Divert Valve	Source

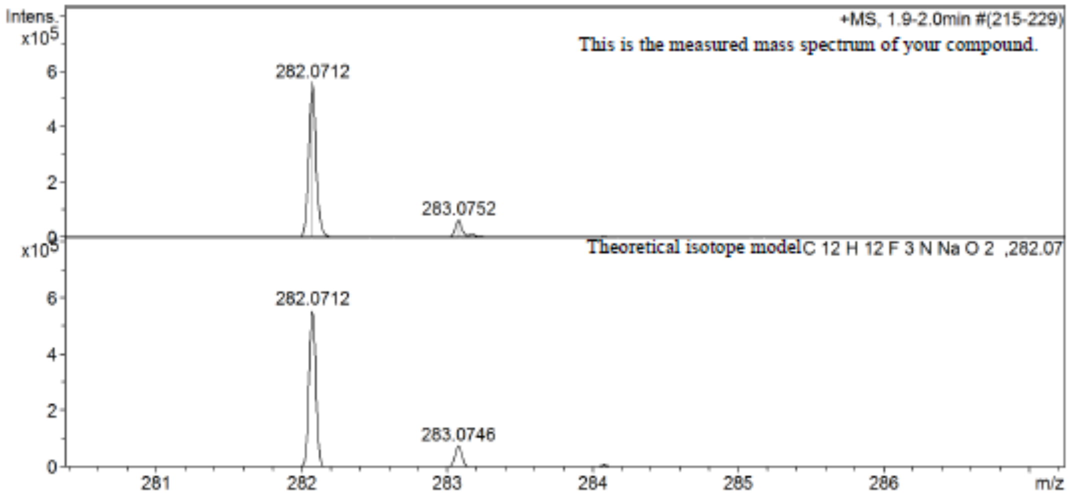

Meas. m/z	#	Formula	m/z	err [ppm]	Mean err [ppm]	rdb	e⁻ Conf	mSigma
282.0712								
	1	C 12 H 12 F 3 N Na O 2	282.0712	0.0	-0.2	5.5	even	9.07

High resolution mass spectrum of **26**.

tert-butyl 3-((2-methoxy-5-(trifluoromethyl)phenyl)carbamoyl)azetidine-1-carboxylate [27]

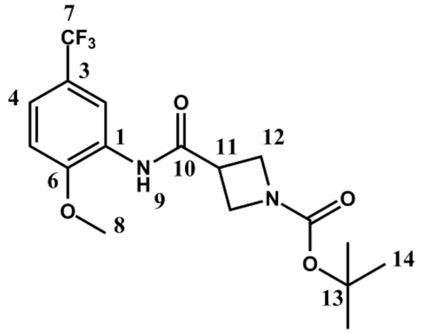

Prepared according to the representative procedure from 2-methoxy-5-(trifluoromethyl)aniline on a 0.37 mmol scale. Chromatography (silica gel, petroleum ether : ethyl acetate 3:1), 86mg, 61% as a white solid.

δH (500 MHz, C_6D_6, 23˚C): 9.25 (1H, bs, H2), 7.13 (1H, bs, H9), 7.11 (1H, dd, J_1 8 Hz, J_2 2 Hz, H4), 6.18 (1H, d, J 9 Hz, H5), 4.13 (2H, bs, H12), 3.66 (2H, t, J 8 Hz, H12), 3.09 (3H, m, H8), 2.25 (1H, m, H11), 1.43 (9H, s, H14).

δC (125 MHz, C_6D_6, 23˚C): 170.03 (C10), 156.67 (C15), 150.29 (C6), 128.39 (C1), 125.50 (q, $^1J_{13C-19F}$ 272 Hz, C7), 124.02 (q, $^2J_{13C-19F}$ 32 Hz, C3), 121.33 (q, $^3J_{13C-19F}$ 4 Hz, C4), 117.59 (q, $^3J_{13C-19F}$ 3 Hz, C2), 110.13 (C5), 79.72 (C13), 55.62 (C8), 52.42 (C12), 34.85 (C11), 28.75 (C14).

δF (370 MHz, C_6D_6, 23˚C): -61.24 (3F, s, F7).

HRMS: (ES- Na): 397.1343; Formula $C_{17}H_{21}F_3N_2O_4Na$, [M + Na] requires 397.1346.

ν_{max} (neat, cm^{-1}): 3291.63, 2977.43, 2981.62, 2162.72, 1979.36, 1675.80, 1615.55, 1541.57, 1491.17, 1409.86, 1366.03, 1339.58, 1321.51, 1269.11, 1224.31, 1160.23, 1112.39, 1079.13, 1023.50, 920.50, 898.08, 855.67, 813.15, 774.01, 653.46, 622.23.

MP: 43-45 °C

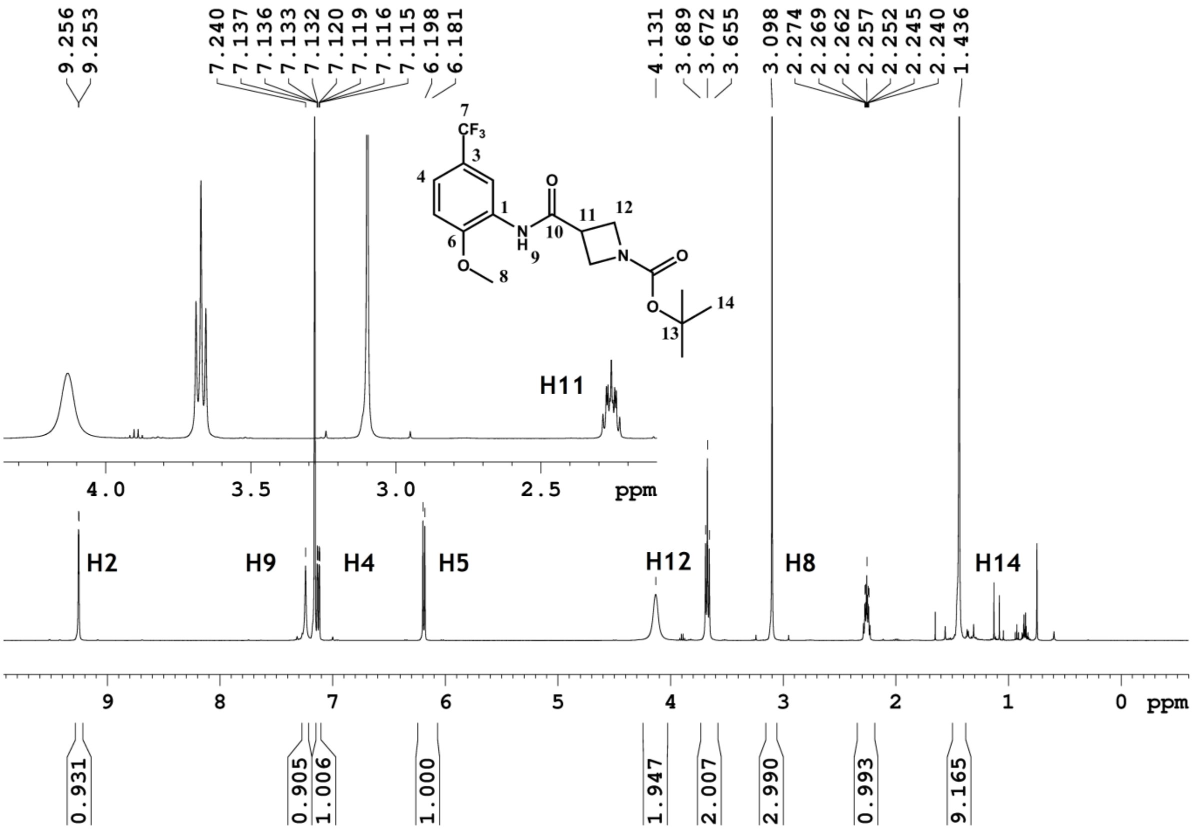

^{1}H NMR spectrum of **27** at (23˚C, C_6D_6, 500 MHz).

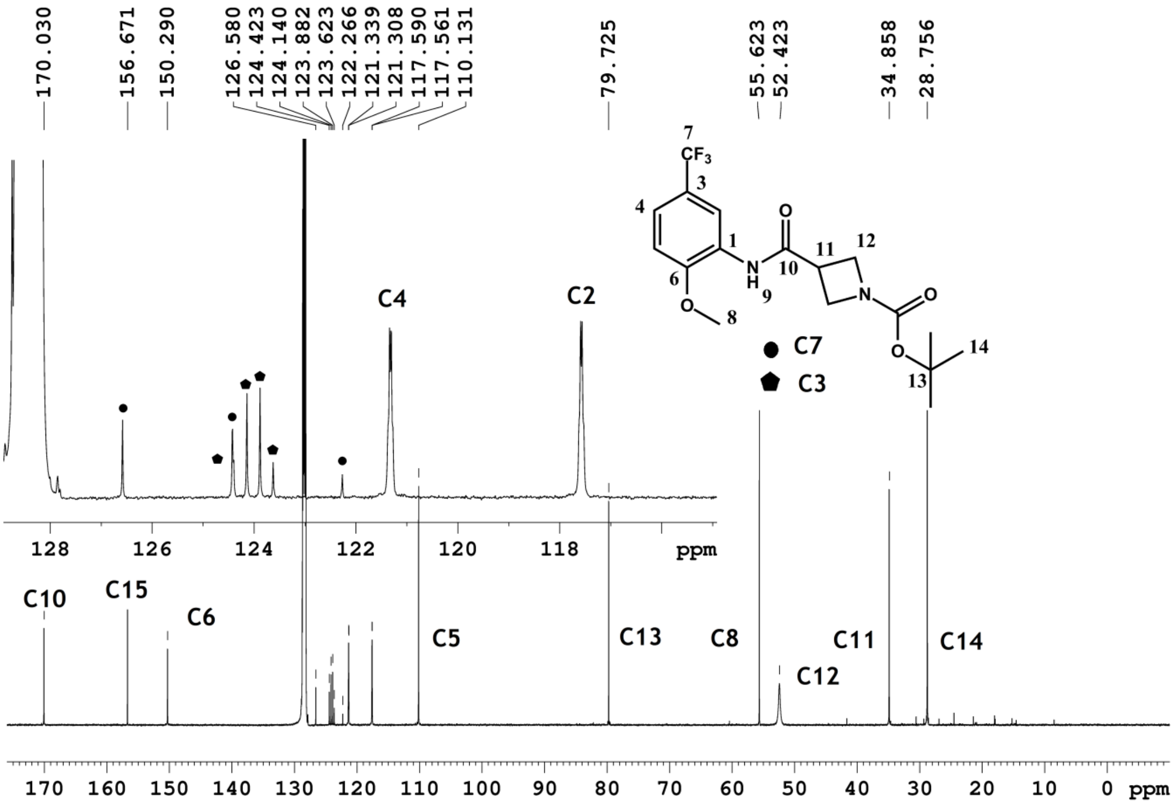

^{13}C NMR spectrum of **27** (23˚C, C_6D_6, 125 MHz)

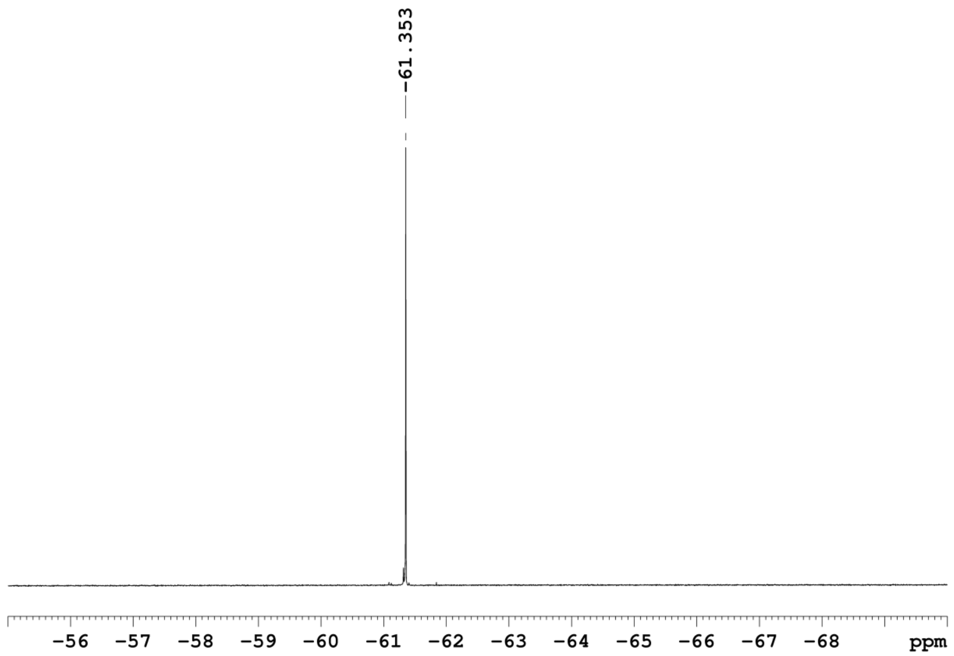

^{19}F NMR spectrum of **27** (23˚C, C_6D_6, 376 MHz).

Mass Spectrum SmartFormula Report

Analysis Info

Analysis Name	\\Utof\data\Jan 14\ESI44490_3_01_16026.d	Acquisition Date	30/01/2014 08:01:53
Method	2.5min_cal_sample_pos_naf_05-08-13.m	Operator	Mass Spec
Sample Name	ESI44490	Instrument / Ser#	micrOTOF 92
Comment			

Acquisition Parameter

Source Type	ESI	Ion Polarity	Positive	Set Nebulizer	2.0 Bar
Focus	Not active			Set Dry Heater	180 °C
Scan Begin	100 m/z	Set Capillary	4500 V	Set Dry Gas	10.0 l/min
Scan End	1000 m/z	Set End Plate Offset	-500 V	Set Divert Valve	Source

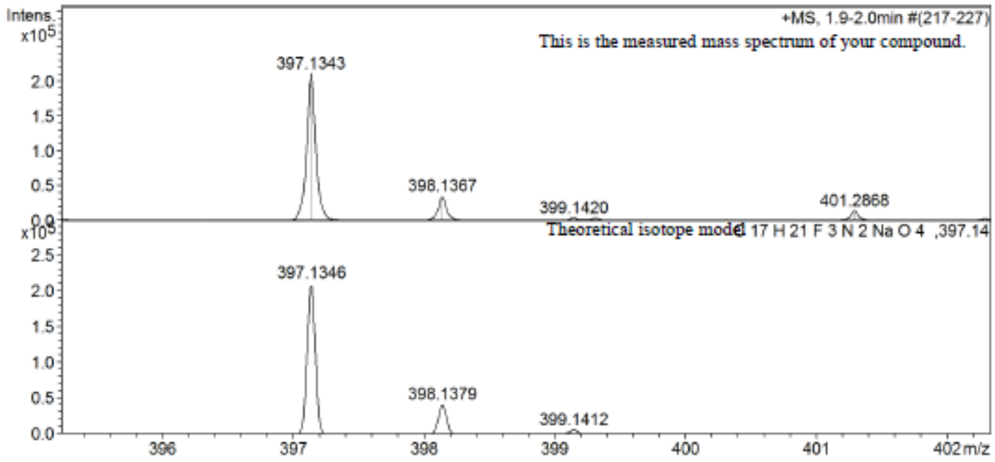

Meas. m/z	#	Formula	m/z	err [ppm]	Mean err [ppm]	rdb	e¯ Conf	mSigma
397.1343								
	1	C 17 H 21 F 3 N 2 Na O 4	397.1346	0.6	0.9	6.5	even	13.42

High resolution mass spectrum of **27**.

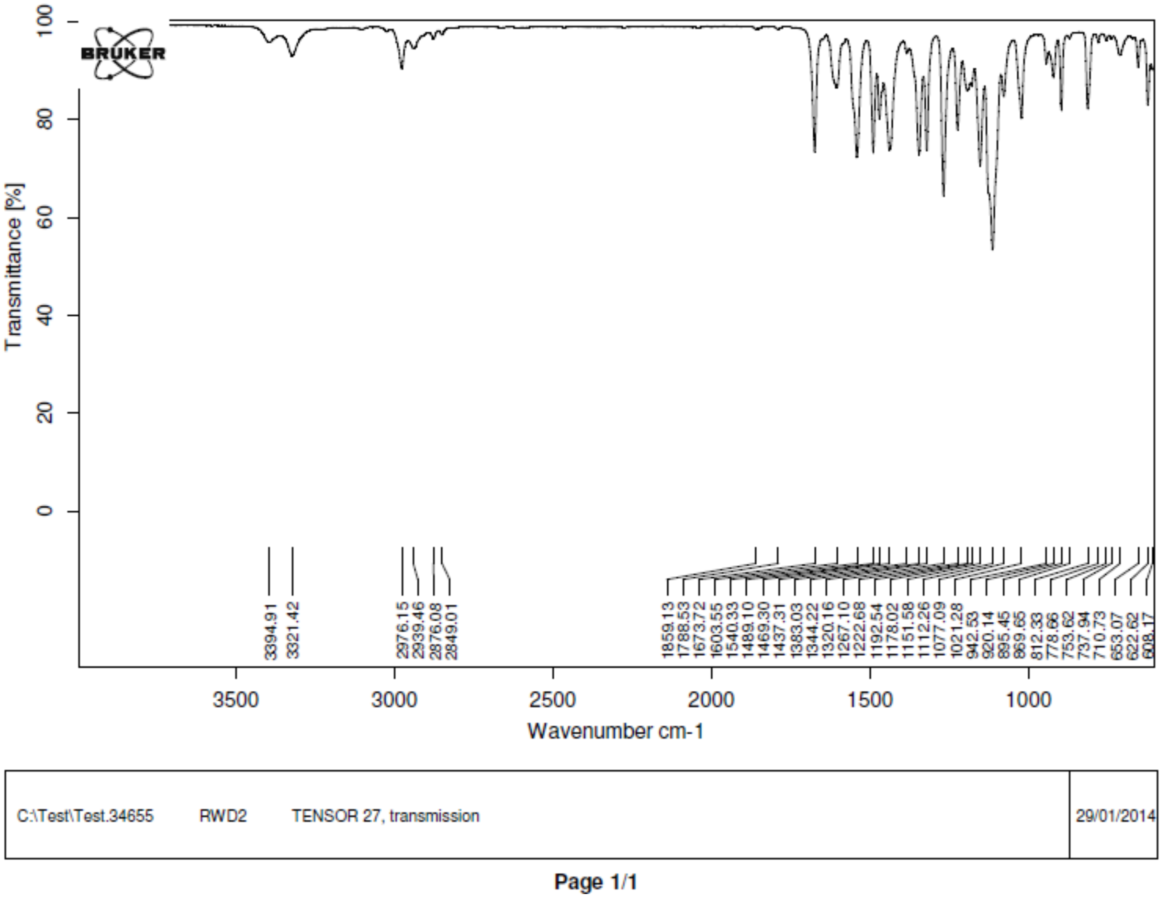

BRUKER
Transmittance [%]
Wavenumber cm-1
3394.91
3321.42
2976.15
2939.46
2876.08
2849.01
1859.13
1788.53
1673.72
1603.55
1540.33
1489.10
1469.30
1437.31
1383.03
1344.22
1320.16
1267.10
1222.68
1192.54
1178.02
1151.58
1112.26
1077.09
1021.28
942.53
920.14
895.45
869.65
812.33
778.66
753.62
737.94
710.73
653.07
622.62
608.17
C:\Test\Test.34655
RWD2
TENSOR 27, transmission
29/01/2014
Page 1/1

FT-IR of **27**.

N-(2-methoxy-5-(trifluoromethyl)phenyl)cyclobutanecarboxamide [28]

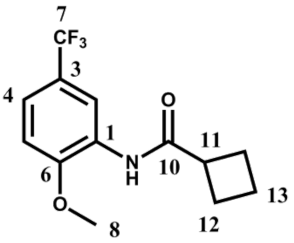

Prepared according to the representative procedure from 2-methoxy-5-(trifluoromethyl)aniline on a 0.29 mmol scale. Chromatography (4:1 PET:EtOAc) to yield a white solid (27 mg, 34%) for X-ray crystallographic and NMR studies.

δH (500 MHz, C_6D_6, 23˚C): 9.44 (1H, s, H2), 7.27 (1H, s, H9), 7.11 (1H, dd, J_1 8 Hz, J_2 2 Hz, H4), 6.19 (1H, d, J 8 Hz, H5), 3.06 (3H, s, H8), 2.44 (1H, pentet, J 8 Hz, H11), 2.30 (2H, m, H12), 1.81 (2H, m, H12), 1.68-1.61 (2H, m, H13).

δC (125 MHz, C_6D_6, 23˚C): 172.84 (C10), 150.06 (C6), 129.40 (C1), 125.61 (q, $^1J_{13C\text{-}19F}$ 271 Hz, C7), 124.07 (q, $^2J_{13C\text{-}19F}$ 32 Hz, C3), 120.61 (q, $^3J_{13C\text{-}19F}$ 4 Hz, C4), 117.13 (q, $^3J_{13C\text{-}19F}$ 3 Hz, C2), 109.92 (C5), 55.53 (C8), 41.46 (C11), 25.73 (C12), 18.67 (C13).

HRMS: (ES-): 296.0869; Formula $C_{13}H_{14}F_3NO_2Na$, [M + Na] requires 296.0870.

ν_{max} (neat, cm^{-1}): 3308.36, 2948.74, 1666.19, 1601.88, 1542.73, 1492.51, 1433.39, 1341.36, 1319.78, 1266.73, 1224.80, 1180.10, 1158.89, 1125.74, 1093.66, 1074.62, 1028.26, 944.33, 921.39, 896.04, 814.82, 791.43, 771.76, 733.22, 664.93, 622.45.

MP: 108˚C.

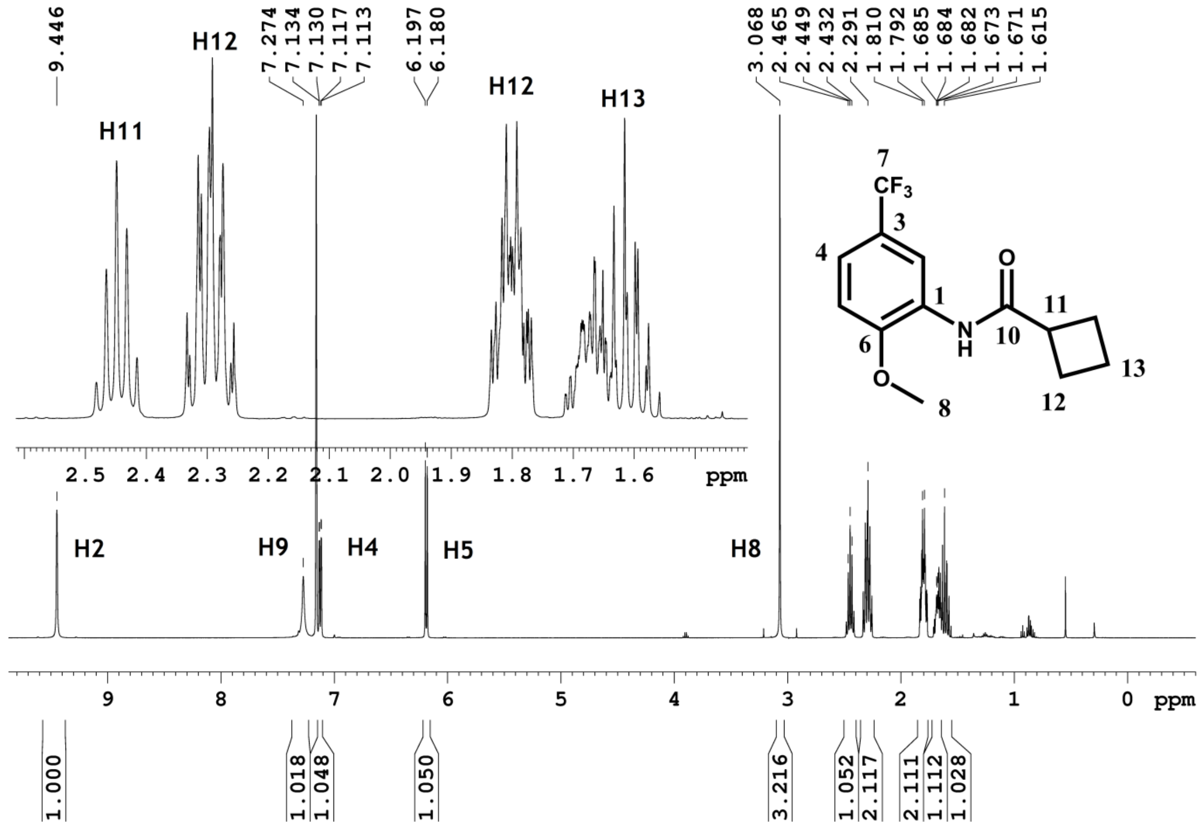

^{1}H NMR spectrum of **28** (C_6D_6 at 23˚C, 500 MHz).

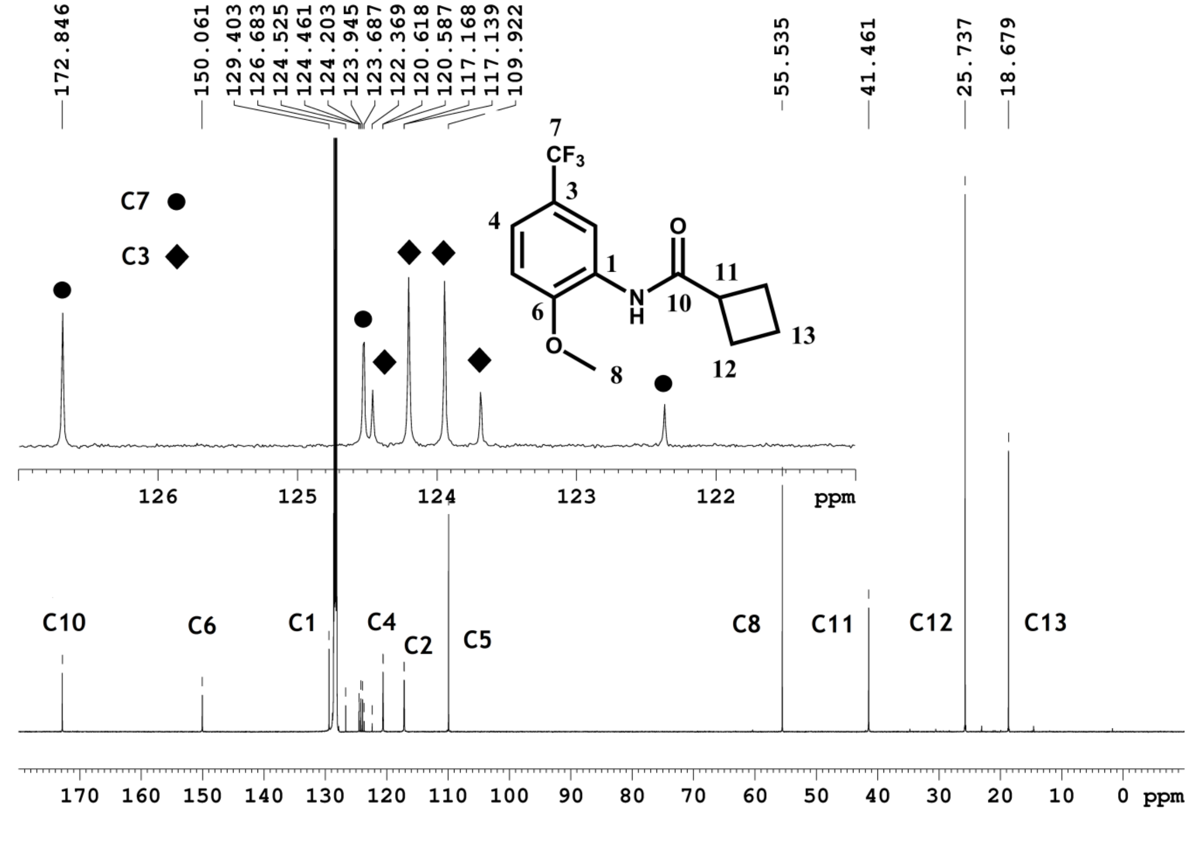

^{13}C NMR spectrum of **28** (C_6D_6 at 23˚C, 125MHz).

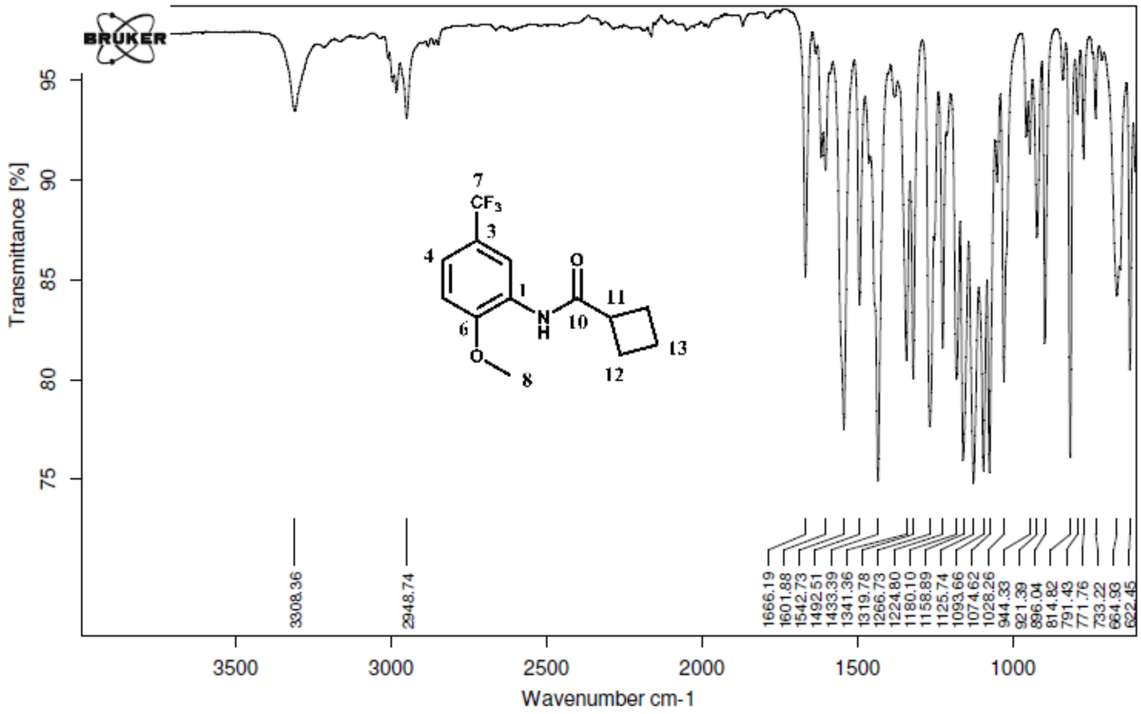

FT-IR of **28**.

Mass Spectrum SmartFormula Report

Analysis Info			
Analysis Name	\\Utof\data\Dec 13\ESI43957_12_01_15068.d	Acquisition Date	17/12/2013 08:26:56
Method	2.5min_cal_sample_pos_naf_05-08-13.m	Operator	Mass Spec
Sample Name	ESI43957	Instrument / Ser#	micrOTOF 92
Comment			

Acquisition Parameter					
Source Type	ESI	Ion Polarity	Positive	Set Nebulizer	2.0 Bar
Focus	Not active			Set Dry Heater	180 °C
Scan Begin	100 m/z	Set Capillary	4500 V	Set Dry Gas	10.0 l/min
Scan End	1000 m/z	Set End Plate Offset	-500 V	Set Divert Valve	Source

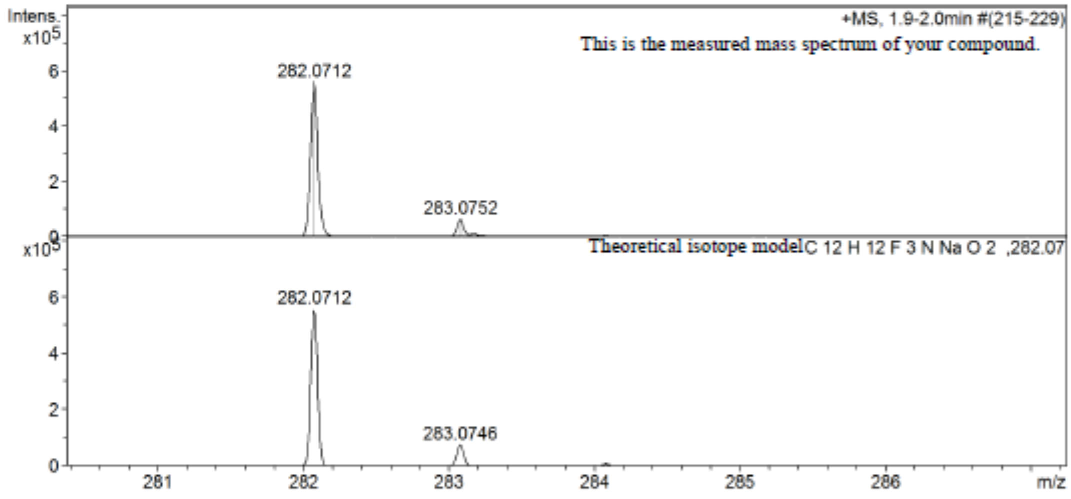

Meas. m/z	#	Formula	m/z	err [ppm]	Mean err [ppm]	rdb	e⁻ Conf	mSigma
282.0712								
	1	C 12 H 12 F 3 N Na O 2	282.0712	0.0	-0.2	5.5	even	9.07

FT-IR of **28**.

N-(2-methoxy-5-(trifluoromethyl)phenyl)isobutyramide [29]

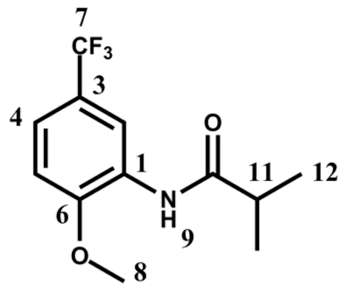

Prepared according to the representative procedure from 2-methoxy-5-(trifluoromethyl)aniline on a 0.26 mmol scale. Chromatography (3:1 PET:EtOAc) to yield a clear oil (15 mg, 20%) for X-ray crystallographic and NMR studies.

δH (700 MHz, C_6D_6, 23˚C): 9.42 (1H, d, *J* 1 Hz, H2), 7.42 (1H, bs, H9), 7.12 (1H, dd, J_1 8 Hz, J_2 1 Hz, H4), 6.18 (1H, d, *J* 9 Hz, H9), 3.06 (3H, s, H8), 1.88 (1H, sept, *J* 7 Hz, H11), 0.99 (3H, s, H12), 0.98 (3H, s, H12).

δC (175 MHz, C_6D_6, 23˚C): 174.75 (C10), 150.13 (C6), 129.41 (C1), 125.59 (q, $^1J_{13C\text{-}19F}$ 272 Hz, C7), 124.14 (q, $^2J_{13C\text{-}19F}$ 32 Hz, C3), 120.69 (q, $^3J_{13C\text{-}19F}$ 4 Hz, C4), 117.31 (q, $^3J_{13C\text{-}19F}$ 3 Hz, C2), 109.92 (C5), 55.57 (C8), 37.22 (C11), 19.89 (C12).

HRMS: (ES+Na): $C_{12}H_{33}F_3NNaO_2$, found (284.0871); [M + Na] requires 284.0869.

ν_{max} (neat, cm^{-1}): 3394.91, 3321.42, 2976.15, 2939.46, 2876.08, 2849.01, 1859.13, 1788.53, 1673.72, 1603.55, 1540.33, 1489.10, 1469.30, 1437.31, 1383.03, 1344.22, 1320.16, 1267.10, 1222.68, 1192.54, 1178.02, 1151.58, 1112.26, 1077.09, 1021.28, 942.53, 920.14, 895.45, 869.65, 812.33, 778.66, 753.62, 737.94, 710.73, 653.07, 622.62, 608.17.

MP: 67°C.

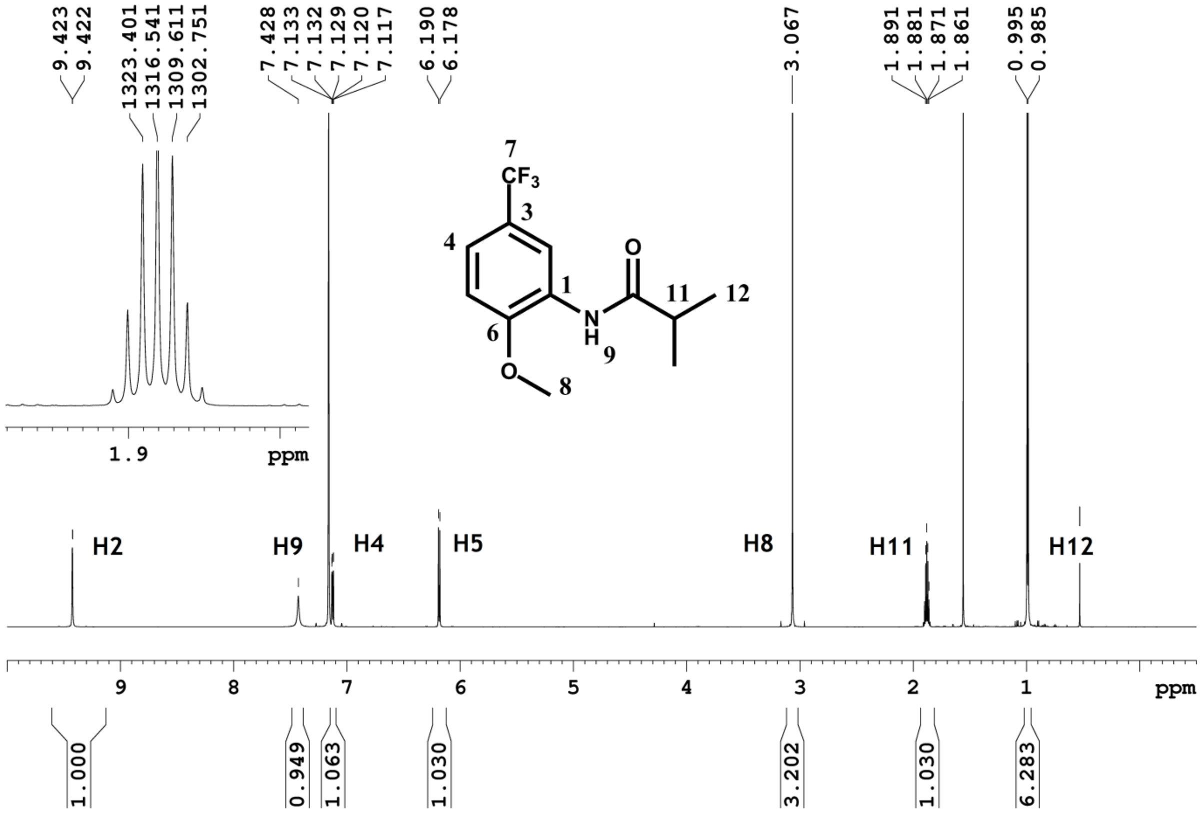

^{1}H NMR spectrum of **29** (23˚C, C_6D_6, 700 MHz).

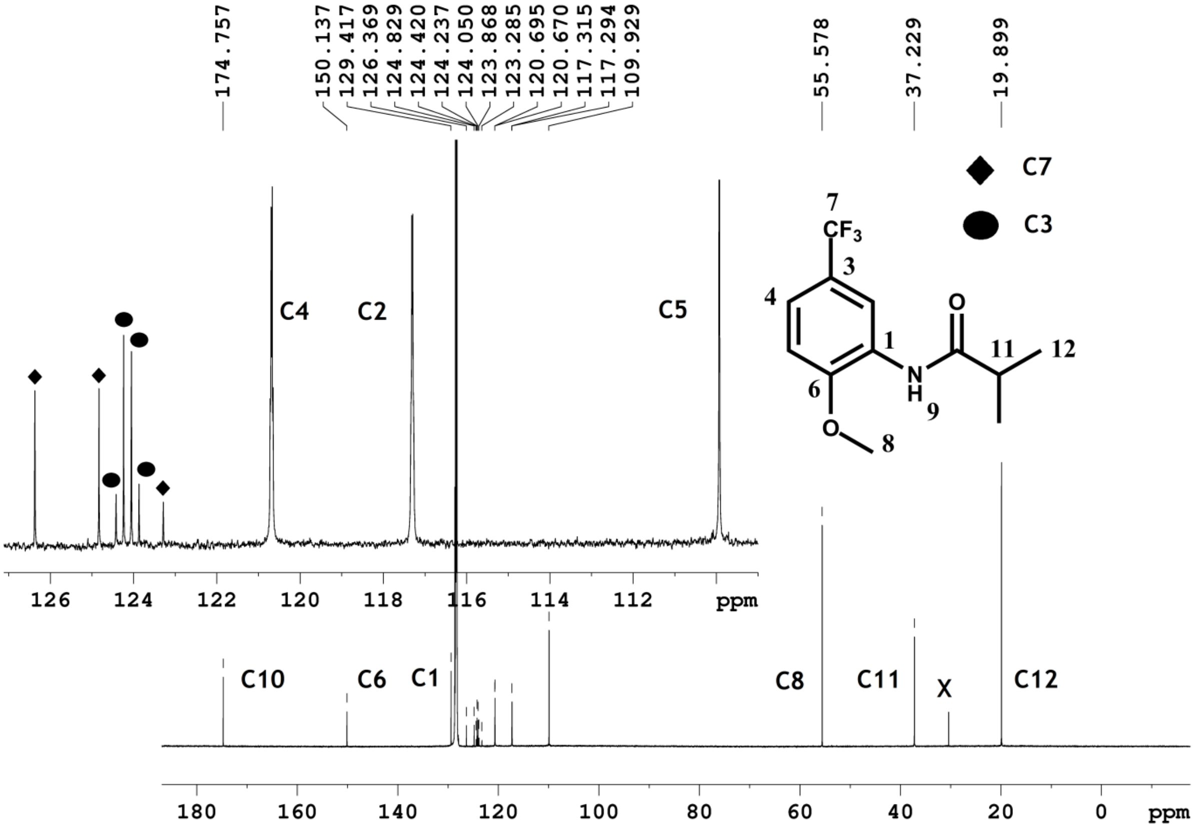

^{13}C NMR spectrum of **29** (23˚C, C_6D_6, 125 MHz).

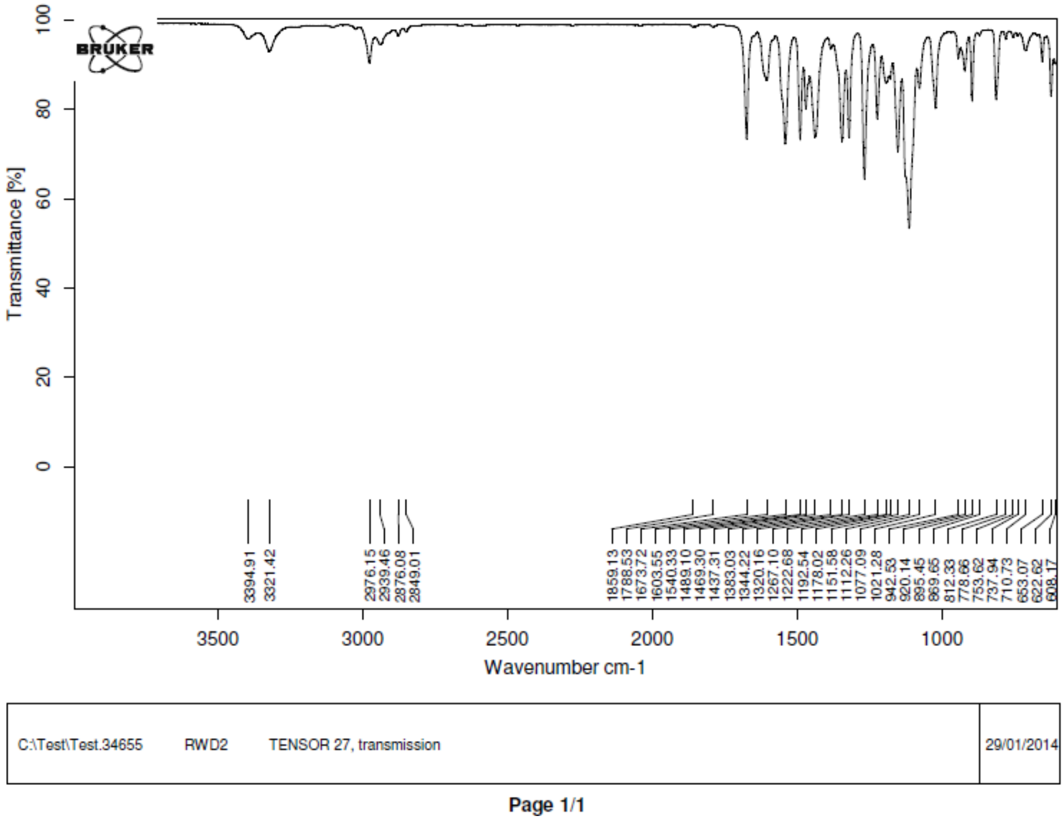

FT-IR of **29**

Mass Spectrum SmartFormula Report

Analysis Info

Analysis Name	\\Utofdata\Jan 14\ESI44521_6_01_16071.d	Acquisition Date	31/01/2014 08:13:25
Method	2.5min_cal_sample_pos_naf_05-08-13.m	Operator	Mass Spec
Sample Name	ESI44521	Instrument / Ser#	micrOTOF 92
Comment			

Acquisition Parameter

Source Type	ESI	Ion Polarity	Positive	Set Nebulizer	2.0 Bar
Focus	Not active			Set Dry Heater	180 °C
Scan Begin	100 m/z	Set Capillary	4500 V	Set Dry Gas	10.0 l/min
Scan End	1000 m/z	Set End Plate Offset	-500 V	Set Divert Valve	Source

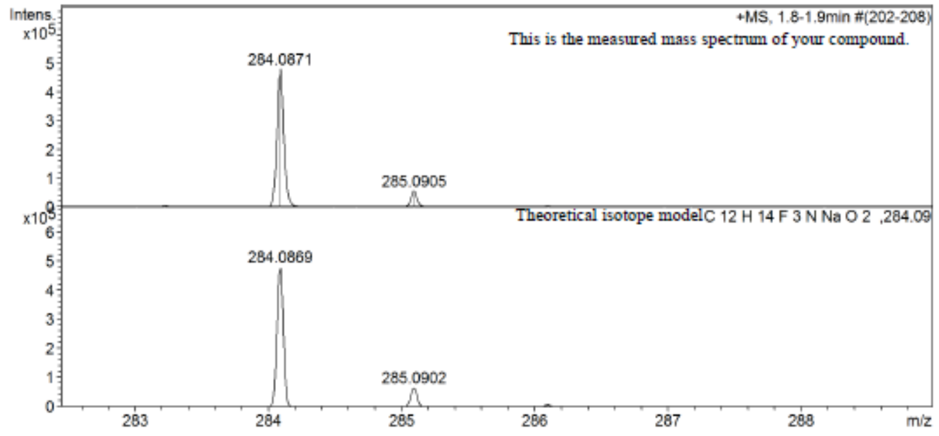

Meas. m/z	#	Formula	m/z	err [ppm]	Mean err [ppm]	rdb	e⁻ Conf	mSigma
284.0871								
	1	C 12 H 14 F 3 N Na O 2	284.0869	-0.8	-0.8	4.5	even	8.89

High resolution mass spectrum of **29**.

tert-butyl 4-((2-methoxy-5-(trifluoromethyl)phenyl)carbamoyl)piperidine-1-carboxylate [30]

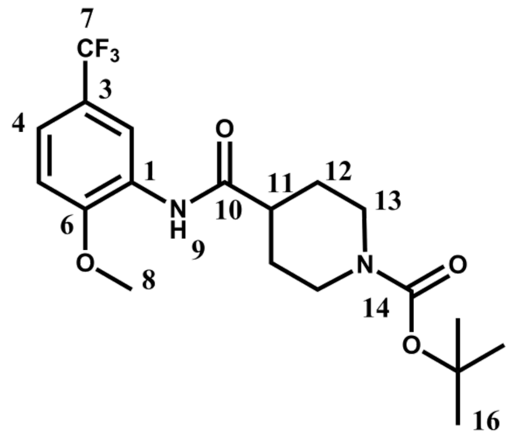

Prepared according to the representative procedure from 2-methoxy-5-(trifluoromethyl)aniline on a 0.26 mmol scale. Chromatography (3:1 PET:EtOAc) to yield clear, pale, colourless needles (32 mg, 31%) for X-ray crystallographic and NMR studies.

δH (500 MHz, C_6D_6, 23˚C): 9.37 (1H, s, H2), 7.36 (1H, bs, H9), 7.16 (1H, dd, obscured, H4), 6.17 (1H, d, *J* 9 Hz, H5), 4.13 (4H, bs, H13), 3.07 (3H, s, H8), 2.43 (2H, m, H12), 2.35 (2H, bs, H12), 1.93 (1H, m, H11), 1.46 (9H, s, H16).

δC (125 MHz, C_6D_6, 23˚C): 172.40 (C10), 154.91,154.84 (C14), 150.11 (C6), 126.60 (C1), 125.55 (q, $^1J_{13C\text{-}19F}$ 272 Hz, C7), 124.28 (q, $^2J_{13C\text{-}19F}$ 32 Hz, C3), 120.95 (q, $^3J_{13C\text{-}19F}$ 4 Hz, C4), 117.48 (q, $^3J_{13C\text{-}19F}$ 4 Hz, C2), 109.99 (C5), 79.59,79.46 (C15), 55.56 (C8), 43.81,43.28 (C13), 41.07 (C11), 28.82 (C16), 28.31 (C12).

δF (376 MHz, C_6D_6, 23˚C): -61.35 (F7).

HRMS: (ES-): 425.1669; Formula $C_{19}H_{25}F_3N_2O_4Na$, [M + Na] requires 425.1659.

ν_{max} (neat, cm^{-1}): 3722.57, 3619.09, 3429.04, 3315.14, 2977.27, 2863.43, 2360.19, 2341.34, 2183.27, 2164.12, 2138.65, 2010.12, 1965.50, 1732.71, 1692.29, 1615.65, 1540.73, 1481.32, 1432.73, 1366.96, 1333.89, 1270.10, 1243.13, 1221.24, 1163.60, 1120.90, 1079.26, 1028.23, 955.77, 923.64, 899.38, 867.75, 816.16, 770.41, 668.78, 654.42, 622.92.

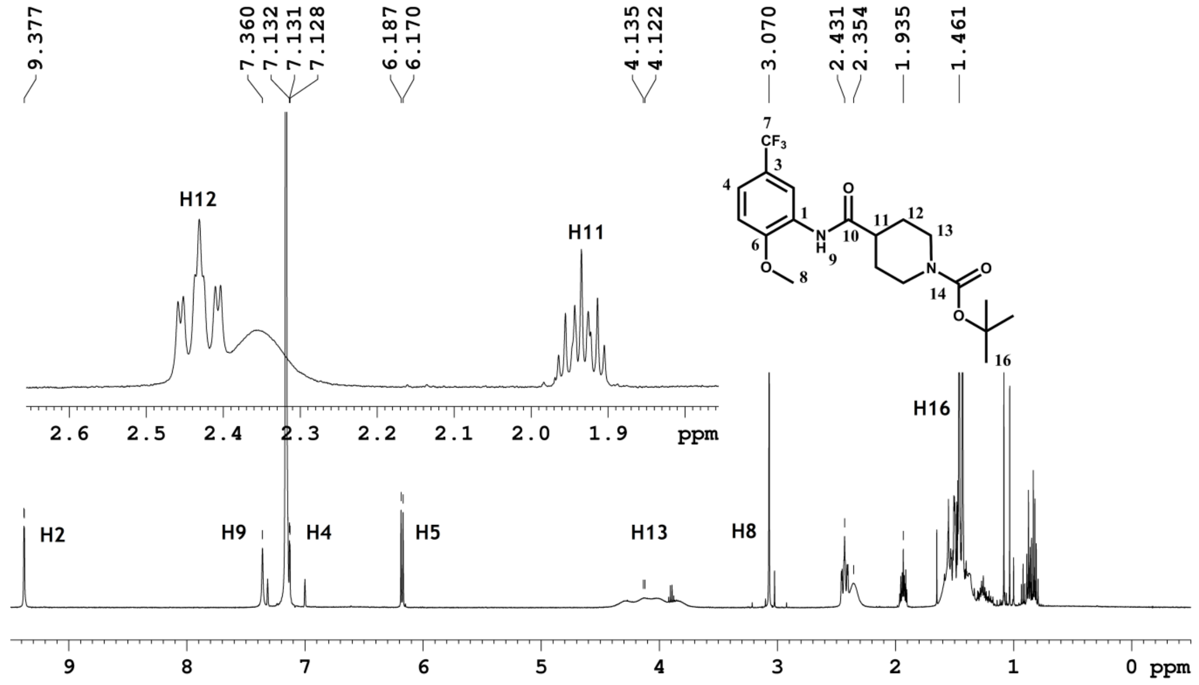

^{1}H NMR spectrum of **30** (23˚C, C_6D_6, 700 MHz).

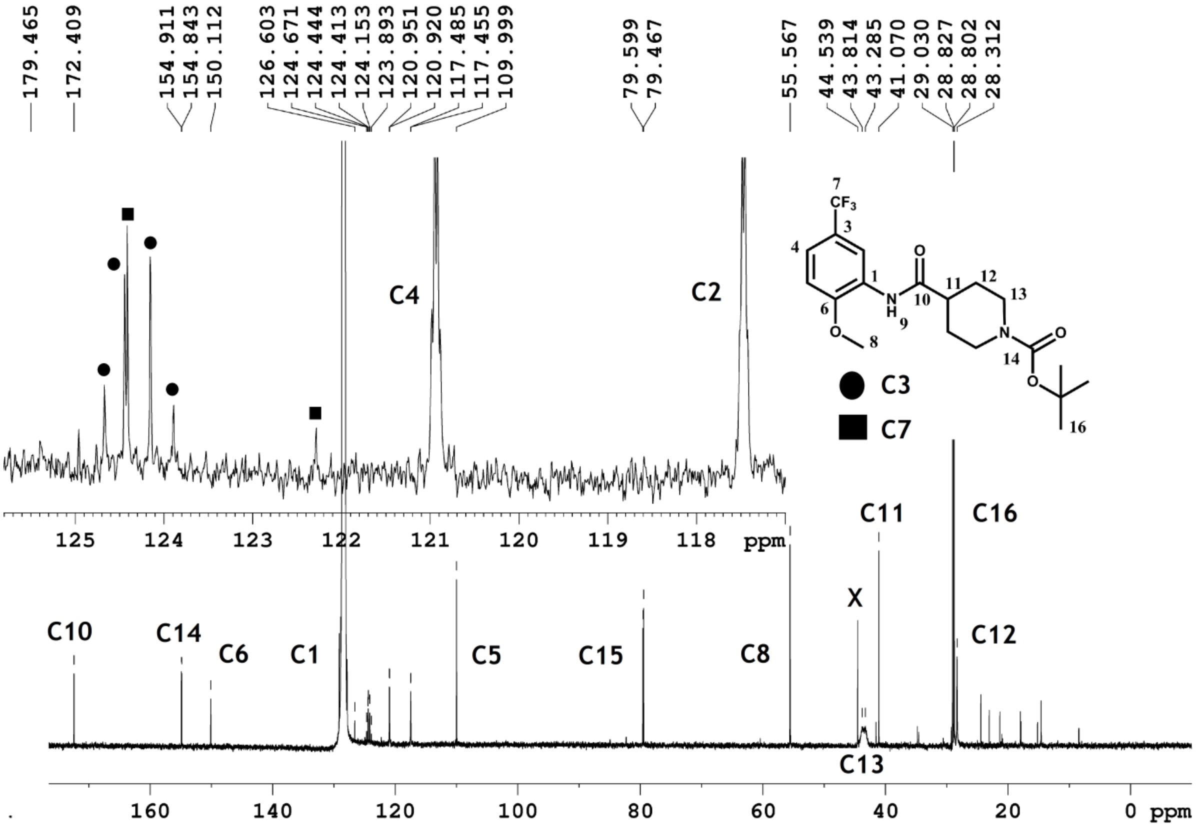

^{13}C NMR spectrum of **30** (23˚C, C_6D_6, 175MHz).

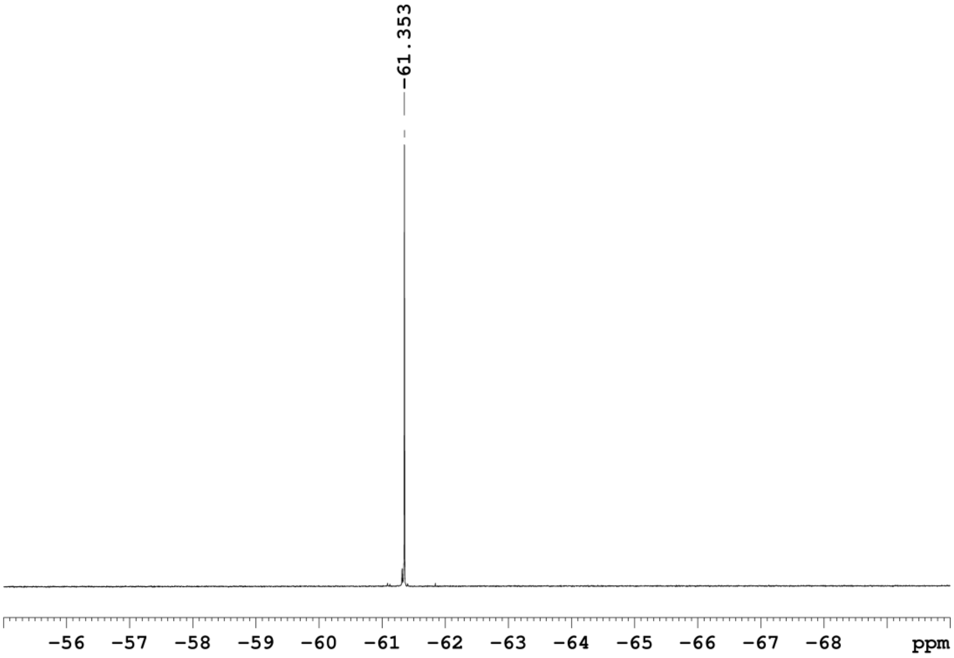

^{19}F NMR spectrum of **30** (23˚C, C_6D_6, 376 MHz).

Mass Spectrum SmartFormula Report

Analysis Info

Analysis Name	\\Utof\data\Feb 14\ESI44851a_13_01_16806.d	Acquisition Date	19/02/2014 10:21:58
Method	2.5min_cal_sample_pos_naf_05-08-13.m	Operator	Mass Spec
Sample Name	ESI44851a	Instrument / Ser#	micrOTOF 92
Comment			

Acquisition Parameter

Source Type	ESI	Ion Polarity	Positive	Set Nebulizer	2.0 Bar
Focus	Not active			Set Dry Heater	180 °C
Scan Begin	100 m/z	Set Capillary	4500 V	Set Dry Gas	10.0 l/min
Scan End	1000 m/z	Set End Plate Offset	-500 V	Set Divert Valve	Source

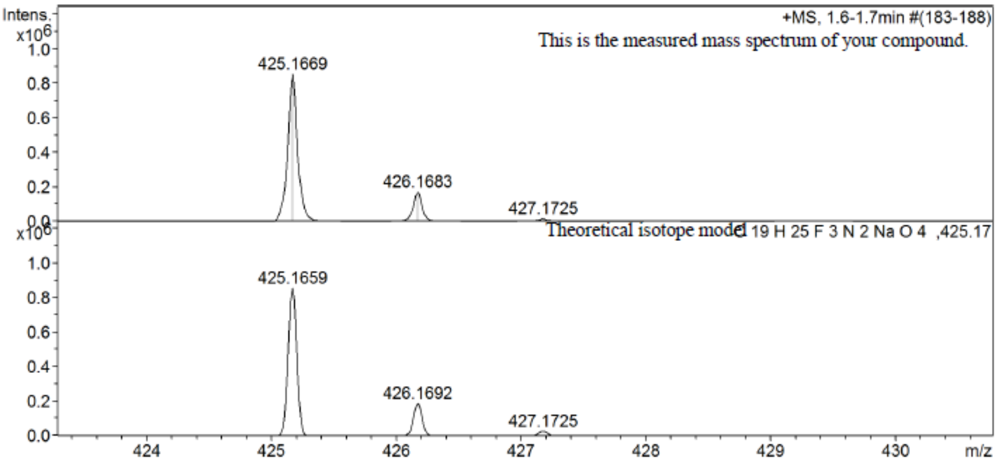

Meas. m/z	#	Formula	m/z	err [ppm]	Mean err [ppm]	rdb	e⁻ Conf	mSigma
425.1669								
	1	C 19 H 25 F 3 N 2 Na O 4	425.1659	-2.5	-1.7	6.5	even	8.79

High resolution mass spectrum of **30**.

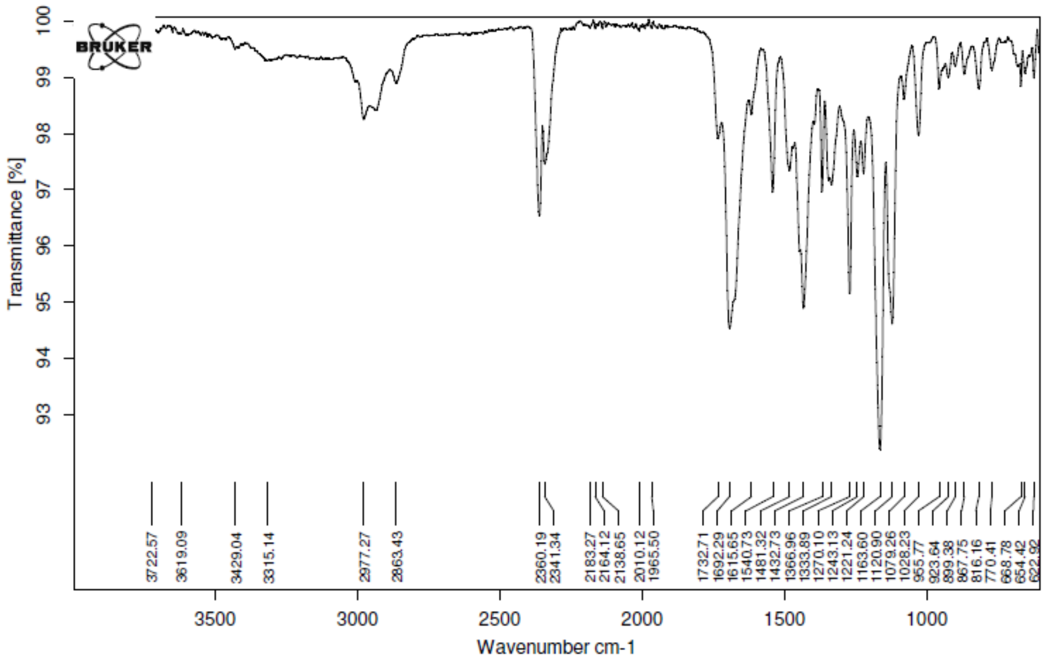

FT-IR of **30**.

N-(2-methoxy-5-(trifluoromethyl)phenyl)acetamide [31]

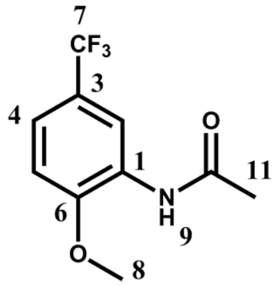

Prepared according to the representative procedure from 2-methoxy-5-(trifluoromethyl)aniline on a 0.4 mmol scale. Chromatography (silica gel, petroleum ether : ethyl acetate 3:1), 45mg, 51% as a white solid.

δH (700 MHz, C_6D_6, 23˚C): 9.32 (s, H2), 7.20 (H9), 7.12 (1H, dd, J_1 9 Hz J_2 2 Hz, H8), 6.19 (1H, d, J 8 Hz, H5), 3.08 (3H, s, H8), 1.47 (3H, s, H11).

δC (175 MHz, C_6D_6, 23˚C): 167.79 (C10), 150.00 (C6), 129.33 (C1), 125.59 (q, $^1J_{13C\text{-}19F}$ 269 Hz, C7), 124.00 (q, $^2J_{13C\text{-}19F}$ 33 Hz, C3), 120.75 (q, $^3J_{13C\text{-}19F}$ 4 Hz, C4), 117.14 (q, $^3J_{13C\text{-}19F}$ 4 Hz, C2), 109.98 (C5), 55.52 (C8), 24.30 (C11).

HRMS: (ES+Na): $C_{10}H_{10}F_3NNaO_2$, found (256.0056);, [M + Na] requires 256.0056.

ν_{max} (neat, cm^{-1}): 3309.40, 3109.57, 3024.46, 2978.54, 2949.78, 2847.52, 1873.37, 1789.77, 1672.70, 1607.43, 1541.99, 1493.11, 1466.40, 1434.21, 1374.70, 1344.52, 1322.51, 1269.18, 1248.16, 1213.84, 1171.25, 1116.45, 1077.62, 1024.94, 961.38, 912.29, 896.52, 814.06, 795.76, 739.66, 712.98, 655.51, 622.57.

MP: 93°C

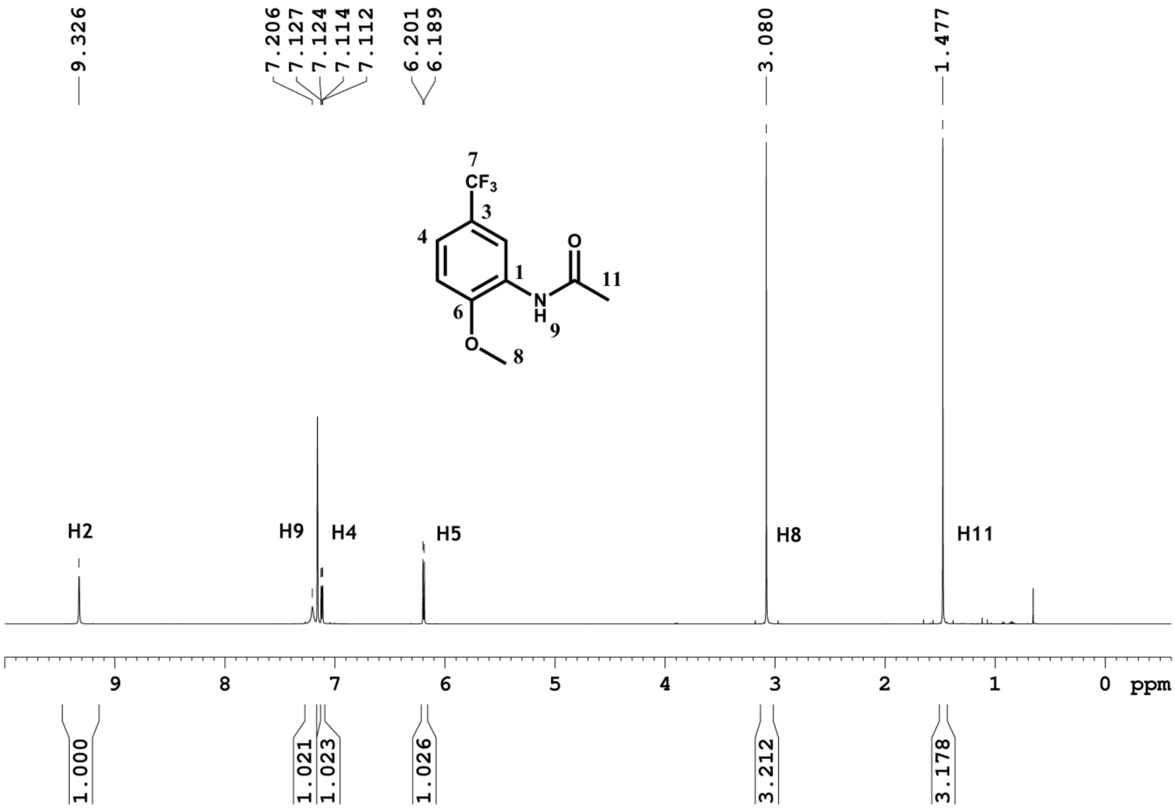

^{1}H NMR spectrum of **31** (23˚C, C_6D_6, 700 MHz).

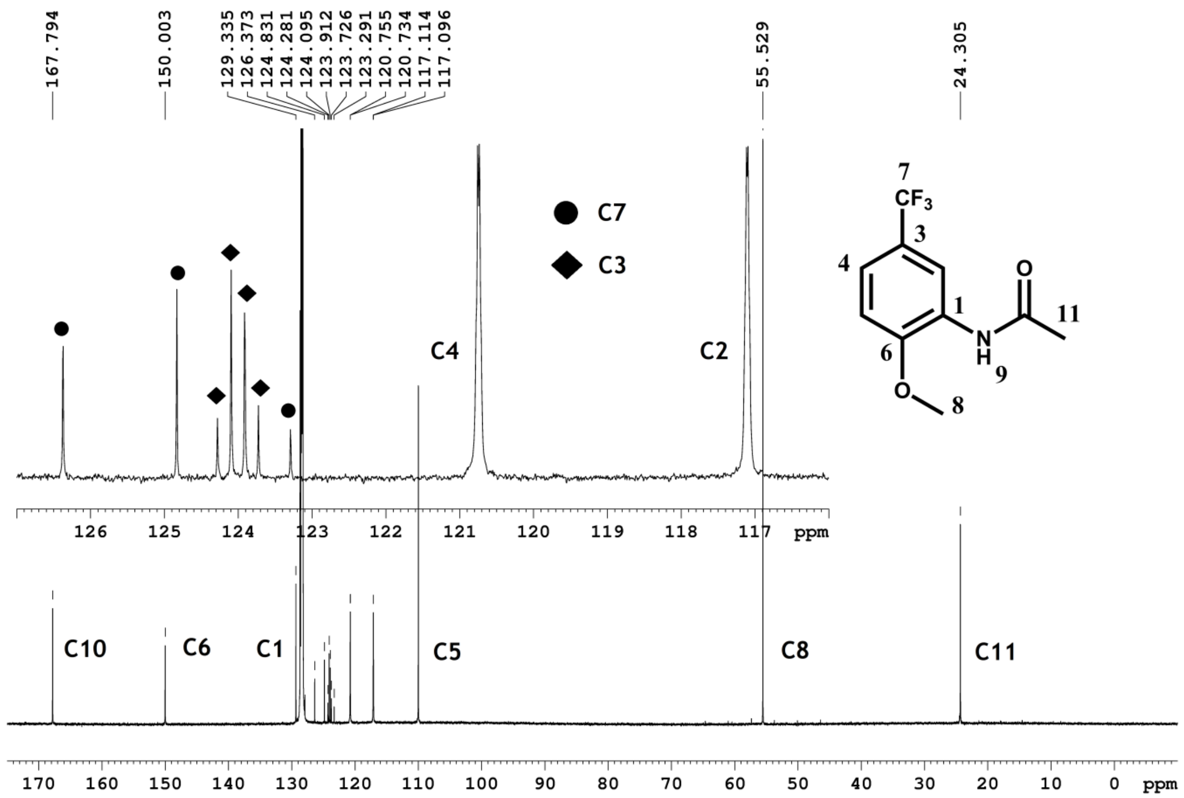

^{13}C NMR spectrum of **31** (23˚C, C_6D_6, 175 MHz).

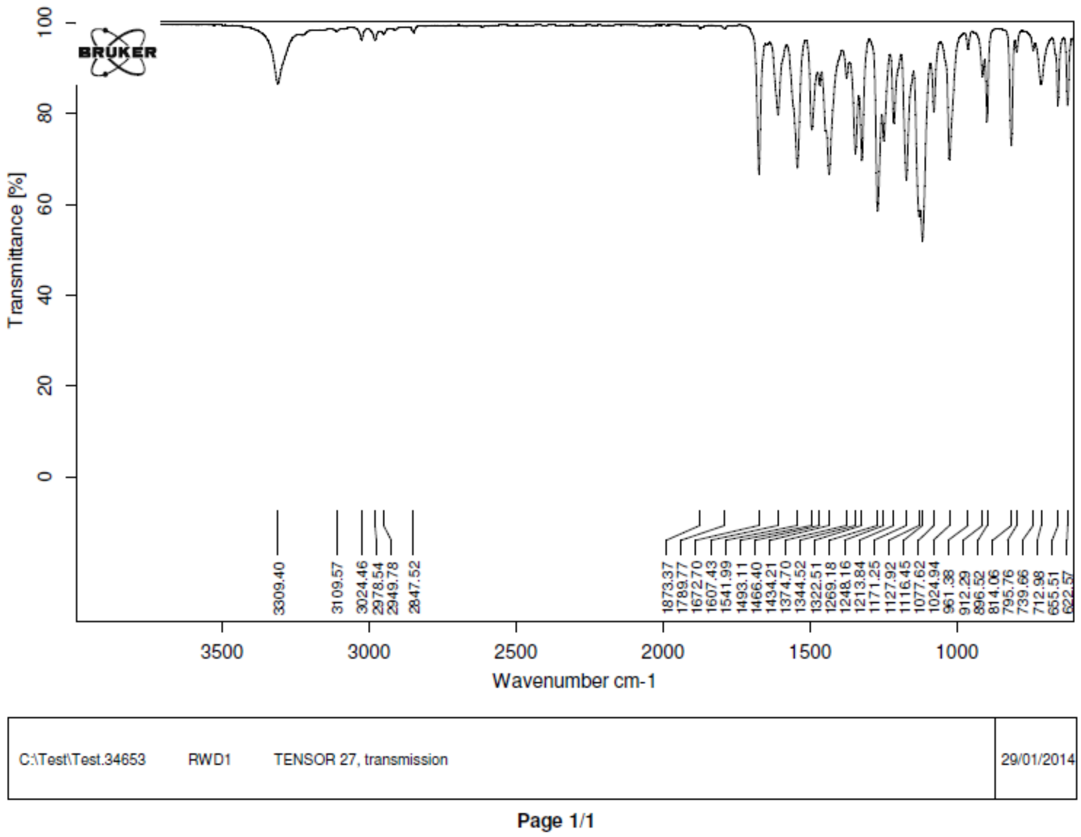

FT-IR of **31**.

Mass Spectrum SmartFormula Report

Analysis Info

Analysis Name	\\Utof\data\Jan 14\ESI44520_5_01_16070.d	Acquisition Date	31/01/2014 08:09:35
Method	2.5min_cal_sample_pos_naf_05-08-13.m	Operator	Mass Spec
Sample Name	ESI44520	Instrument / Ser#	micrOTOF 92
Comment			

Acquisition Parameter

Source Type	ESI	Ion Polarity	Positive	Set Nebulizer	2.0 Bar
Focus	Not active			Set Dry Heater	180 °C
Scan Begin	100 m/z	Set Capillary	4500 V	Set Dry Gas	10.0 l/min
Scan End	1000 m/z	Set End Plate Offset	-500 V	Set Divert Valve	Source

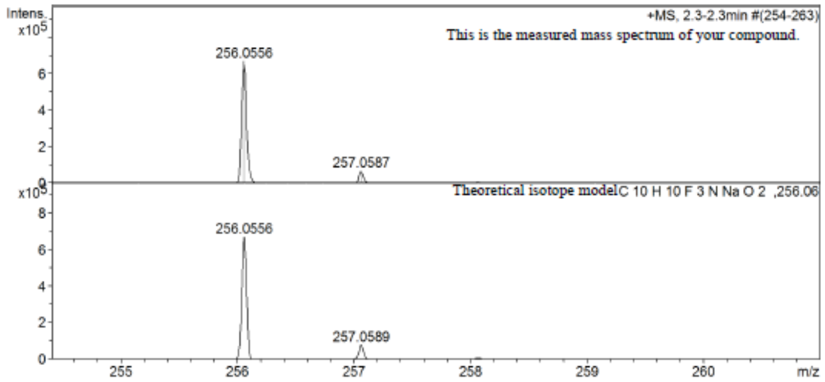

Meas. m/z	#	Formula	m/z	err [ppm]	Mean err [ppm]	rdb	e¯ Conf	mSigma
256.0556								
	1	C 10 H 10 F 3 N Na O 2	256.0556	0.1	0.2	4.5	even	6.64

High-resolution mass spectrum of **31**.

N-(2-methoxy-5-(trifluoromethyl)phenyl)-9H-fluorene-9-carboxamide [32]

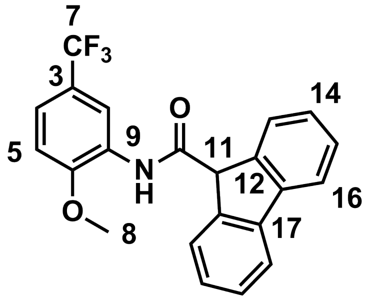

Prepared according to the representative procedure from 2-methoxy-5-(trifluoromethyl)aniline on a 0.26 mmol scale (16% yield). The resulting white solid was dissolved in pentane/ethyl acetate (15:1) and colourless needle crystals grown by the diffusion method for X-ray crystallographic studies.

δH (500 MHz, C_6D_6, 23˚C): 9.35 (1H, d, *J* 2 Hz, H2), 7.61 (2H, d, *J* 6 Hz, H16), 7.50 (2H, d, *J* 7 Hz, H9, H13), 7.18-7.14 (4H, m, H14, H15), 6.97 (1H, dd, J_1 8 Hz, J_2 2 Hz, H4), 5.89 (1H, d, *J* 8 Hz, H16), 4.75 (1H, s, H11), 2.65 (3H, s, H8).

δH (500 MHz, $CDCl_3$, 23˚C): 8.65 (1H, d, *J* 2 Hz, H2), 7.80 (1H, d, *J* 8 Hz, H16), 7.76 (1H, d, *J* 7 Hz, H13), 7.75 (1H, bs, H9), 7.46 (1H, t, *J* 8 Hz, H15), 7.38 (1H, td, J_1 8 Hz, J_1 1 Hz H14), 7.22 (1H, dd, J_1 8 Hz, J_1 2 Hz H14), 4.92 (1H, s, H11), 3.63 (1H, s, H8).

δC (125 MHz, $CDCl_3$, 23˚C): 168.911 (C10), 150.370 (C6), 141.665 (C17), 141.034 (C12), 128.81 (C15), 128.03 (C14), 127.80 (C1), 125.74 (C13), 124.36 (C7, $^1J_{13\text{-}19F}$ 272 Hz), 123.42 (C3, $^2J_{13\text{-}19F}$ 32 Hz), 121.24 (C4, $^3J_{13\text{-}19F}$ 4 Hz), 120.62 (C16), 116.58 (C2, $^3J_{13\text{-}19F}$ 4 Hz), 109.60 (C5), 57.26 (C11), 56.07 (C8).

δF (470 MHz, C_6D_6, 23˚C): -61.69 (F7).

HRMS: $C_{22}H_{16}F_3NaN_2O_2$ theoretical [406.1025], observed [406.1026].

ν_{max} (neat, cm^{-1}): 3289.40, 1664.45, 1614.12, 1542.66, 1493.66, 1345.01, 1327.28, 1270.36, 1222.84, 1163.97, 1116.74, 1078.53, 1022.00, 971.54, 924.86, 896.76, 816.12, 773.04, 735.39, 690.86, 652.20, 621.46.

MP: 185°C.

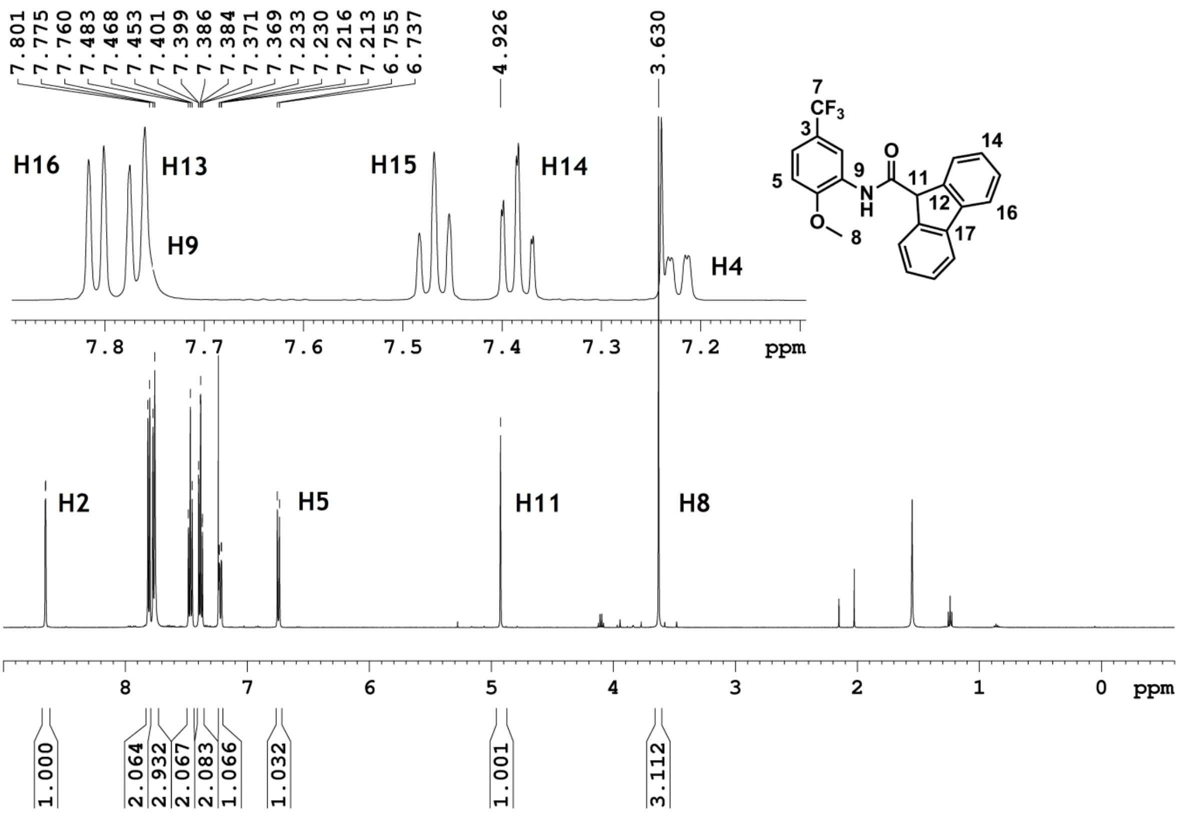

^{1}H NMR spectrum of **32** (23˚C, $CDCl_3$, 500 MHz).

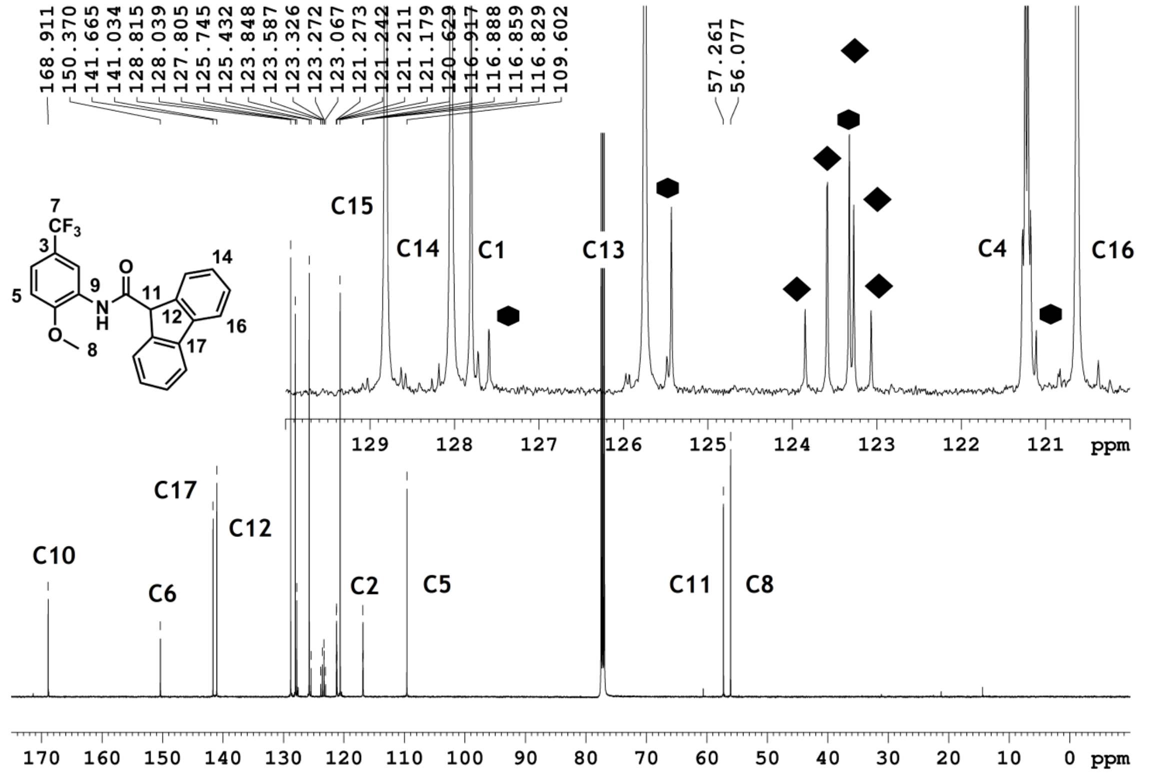

^{13}C NMR spectrum of **32** (23˚C, $CDCl_3$, 125 MHz).

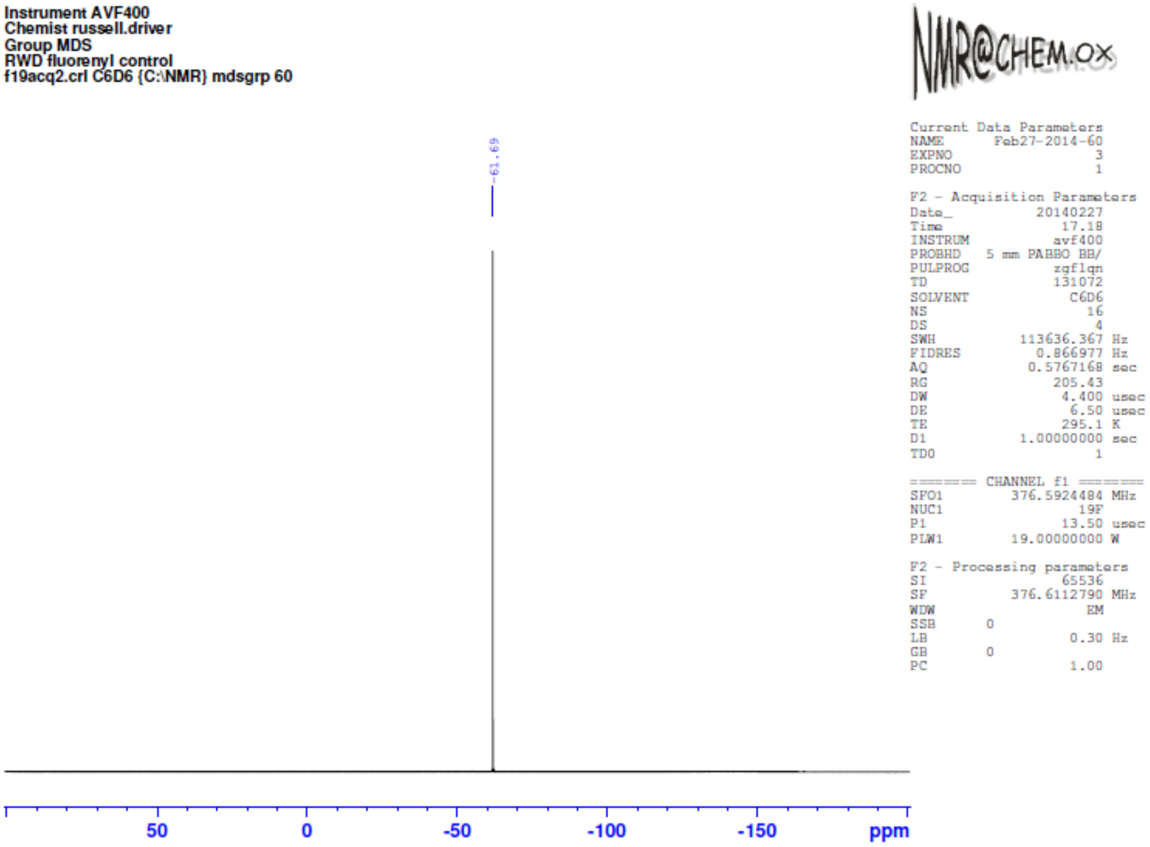

^{19}F NMR spectrum of **32** (23˚C, $CDCl_3$, 376 MHz).

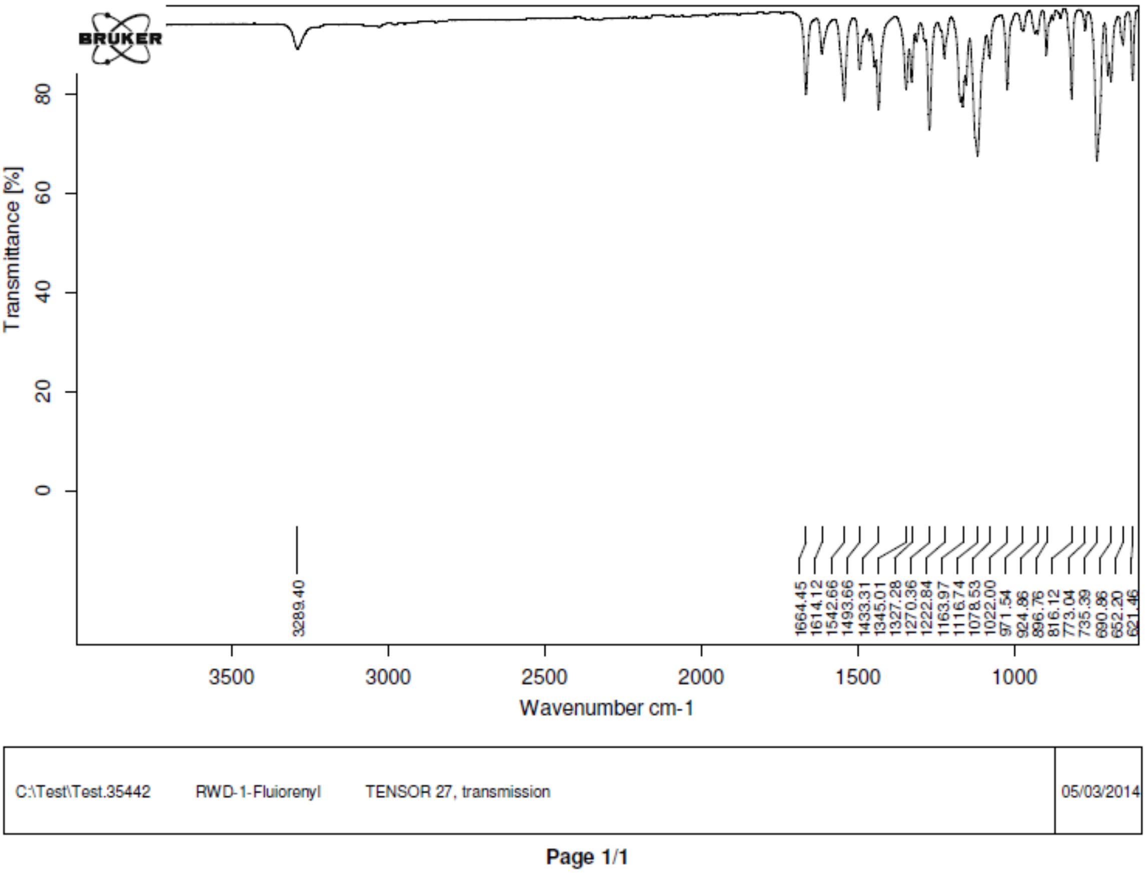

FT-IR of **32**.

Mass Spectrum SmartFormula Report

Analysis Info

Analysis Name	\\Utof\data\Mar 14\ESI45140_13_01_17283.d	Acquisition Date	06/03/2014 08:25:34
Method	2.5min_cal_sample_pos_naf_05-08-13.m	Operator	Mass Spec
Sample Name	ESI45140	Instrument / Ser#	micrOTOF 92
Comment			

Acquisition Parameter

Source Type	ESI	Ion Polarity	Positive	Set Nebulizer	2.0 Bar
Focus	Not active			Set Dry Heater	180 °C
Scan Begin	100 m/z	Set Capillary	4500 V	Set Dry Gas	10.0 l/min
Scan End	1000 m/z	Set End Plate Offset	-500 V	Set Divert Valve	Source

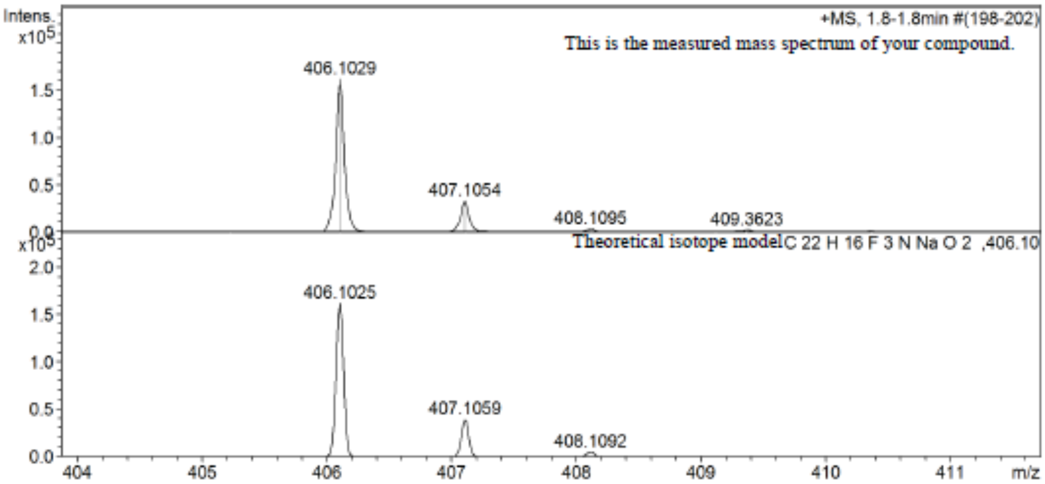

Meas. m/z	#	Formula	m/z	err [ppm]	Mean err [ppm]	rdb	e⁻ Conf	mSigma
406.1029								
	1	C 22 H 16 F 3 N Na O 2	406.1025	-0.8	-0.4	13.5	even	20.84

High-resolution mass spectrum of **32** showing parent ion plus Na cation.

(S)-N-(2-(3,3-dimethyl-2-pivalamidobutoxy)-5-(trifluoromethyl)phenyl)-3,3,3-trifluoro-2-(trifluoromethyl)propanamide [33]

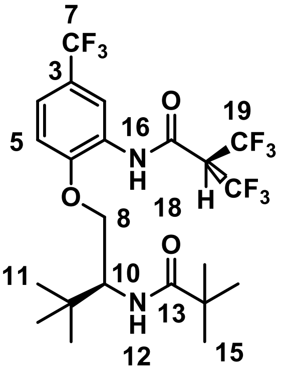

TFA (5mL) was added dropwise to a stirring solution of **8** (100 mg, 0.18 mmol) in DCM (5mL), at 0˚C and the reaction stirred for 15 min. The mixture was concentrated in vacuo, suspended in Et_2O and 0.5mL HCl (2.0M in Et_2O) was added. The resultant white solid was filtered (*ca*. 75mg), DCM was added (50mL) and $NaHCO_3$ (50 mL, aq., saturated) was added. The organic phase was extracted, dried ($MgSO_4$) and concentrated to yield the free amine as a white solid which was used without further purification.

DMAP (2.0 mg) followed by pivaloyl chloride (0.048g, 0.4 mmol) were added to a stirring solution of the crude free amine in DCM (7.0 mL) and DIPEA (0.5 mL). The reaction was stirred overnight before being concentrated in vacuo and the product dissolved in DCM (50 mL). The organic phase was washed with ammonium chloride solution (1 X 25 mL) and brine (1 X 25 mL). The resulting colorless oil was purified by column chromatography (7:1 PET:EtOAc) to yield ca 18 mg (0.039 mmols, 18 %) of the title compound as a colourless oil.

δH (500 MHz, C_6D_6, 23˚C): 9.82 (1H, s, H16), 9.35 (1H, d, *J* 2 Hz, H2), 7.14 (1H, dd, J_1 8 Hz, J_2 2 Hz, H4), 6.09 (1H, d, *J* 9 Hz, H5), 5.70 (1H, sept, *J* 8 Hz, H18), 5.35 (1H, d, *J* 10 Hz, H12), 3.90 (1H, dt, J_1 9 Hz, J_2 3 Hz, H9), 3.51 (1H, dd, J_1 9 Hz, J_2 3 Hz, H8), 3.21 (1H, dd, J_1 11 Hz, J_2 9 Hz, H8), 0.92 (9H, H15), 0.51 (9H, H11).

δC (125 MHz, C_6D_6, 23˚C): 180.08 (C13), 157.87 (C17), 150.07 (C6), 129.40 (C1), 125.27 (C7, q, $^1J_{13C-19F}$ 273 Hz), 124.69 (C3, q, $^2J_{13C-19F}$ 2 Hz), 125.47 (C19, q, $^1J_{13C-19F}$ 276 Hz), 121.95 (C4, q, $^3J_{13C-19F}$ 4 Hz), 117.45 (C2, q, $^3J_{13C-19F}$ 3 Hz), 111.30 (C5), 70.80 (C8), 57.81 (C9), 55.16 (C18, sept, $^3J_{13C-19F}$ 9 Hz), 39.50 (C14), 32.19 (C10), 27.55 (C15), 26.76 (C11).

δF (470 MHz, C_6D_6, 23˚C): -61.29 (F7), -63.44 (3F, quintet, $^3J_{19F-1H}$ 9 Hz, $^4J_{19F-19F}$ 9 Hz), -64.10 (3F, quintet, $^3J_{19F-1H}$ 9 Hz, $^4J_{19F-19F}$ 9 Hz).

HRMS: $C_{22}H_{27}F_9N_2O_3Na$ theoretical [561.1770], observed [561.1764].

ν_{max} (neat, cm^{-1}): 3466.57, 3251.05, 3100.55, 2966.69, 2876.42, 1713.95, 1648.82, 1617.27, 1557.22, 1516.42, 1498.93, 1444.33, 1396.92, 1343.04, 1322.03, 1291.28, 1274.78, 1237.56, 1203.47, 1167.51, 1123.58, 1099.53, 1076.02, 1044.13, 1019.77, 1005.24, 931.21, 920.50, 901.09, 866.65, 838.56, 817.60, 758.65, 721.29, 658.09, 635.93, 625.96.

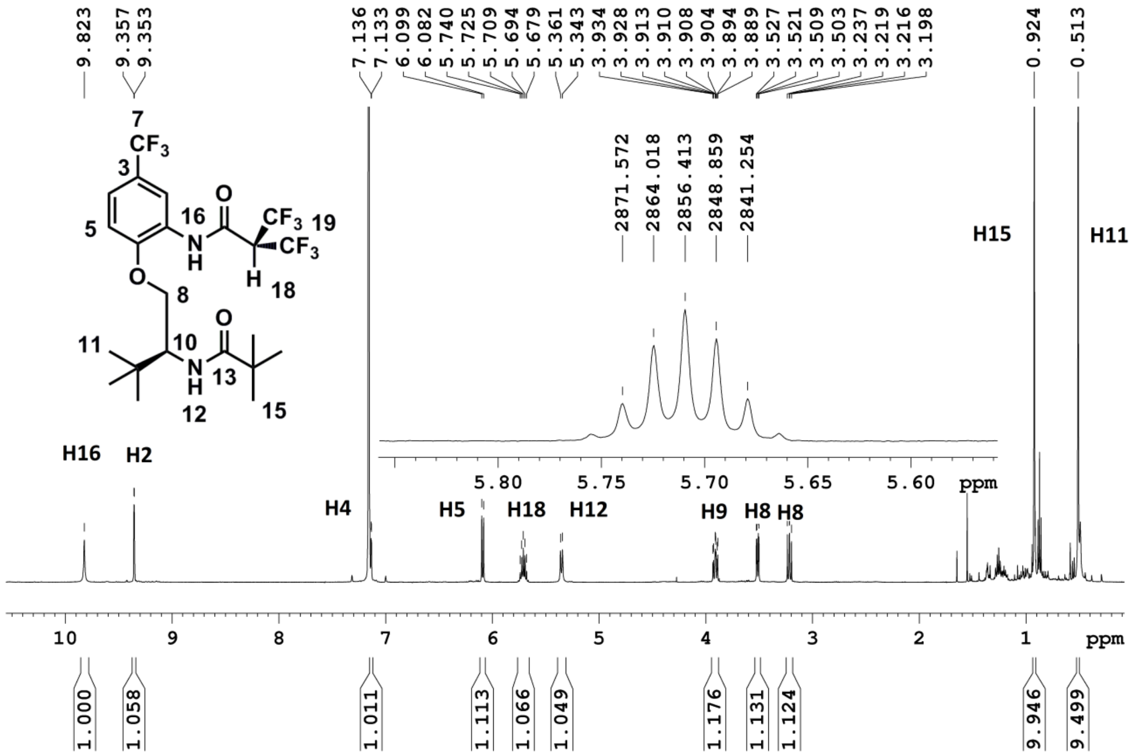

^{1}H NMR spectrum of **33** (23˚C, C_6D_6, 500 MHz).

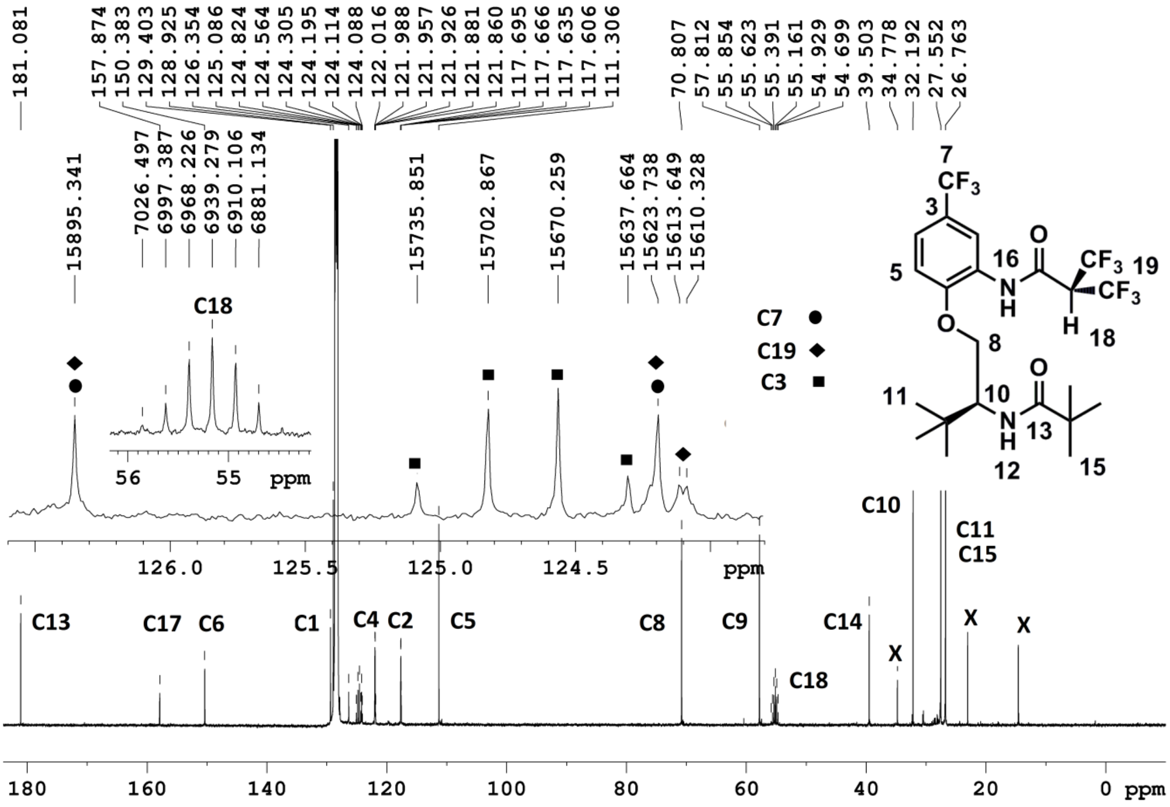

^{13}C NMR spectrum of **33** (23˚C, C_6D_6, 125 MHz).

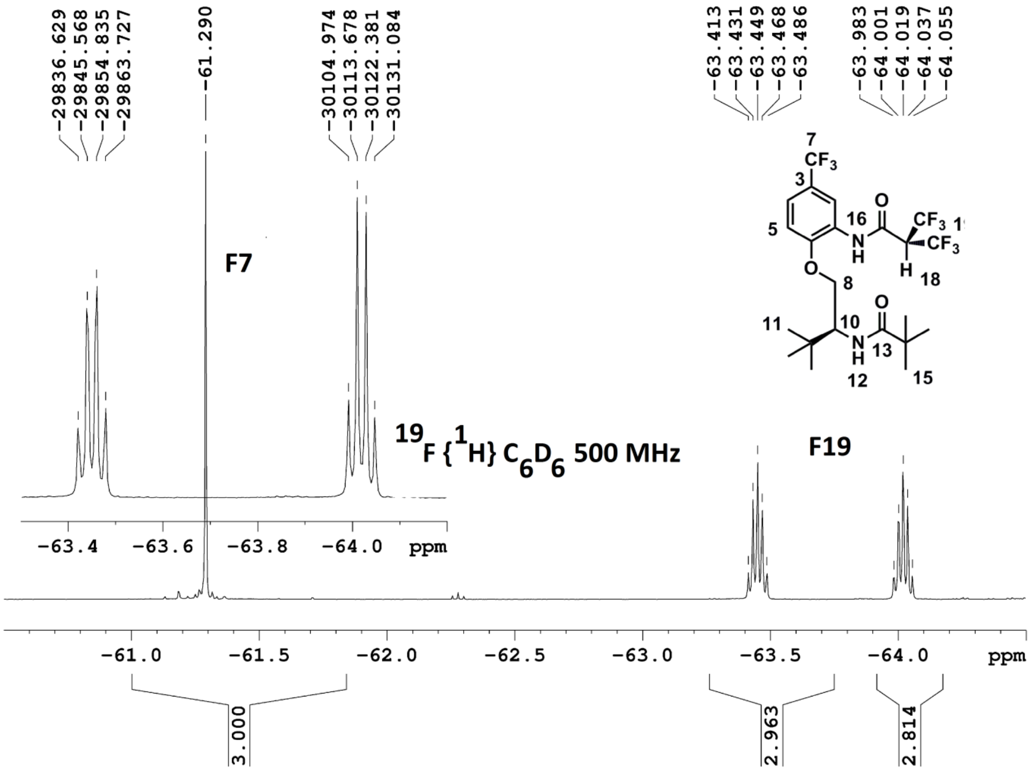

^{19}F NMR spectrum of **33** (23˚C, C_6D_6, 470 MHz).

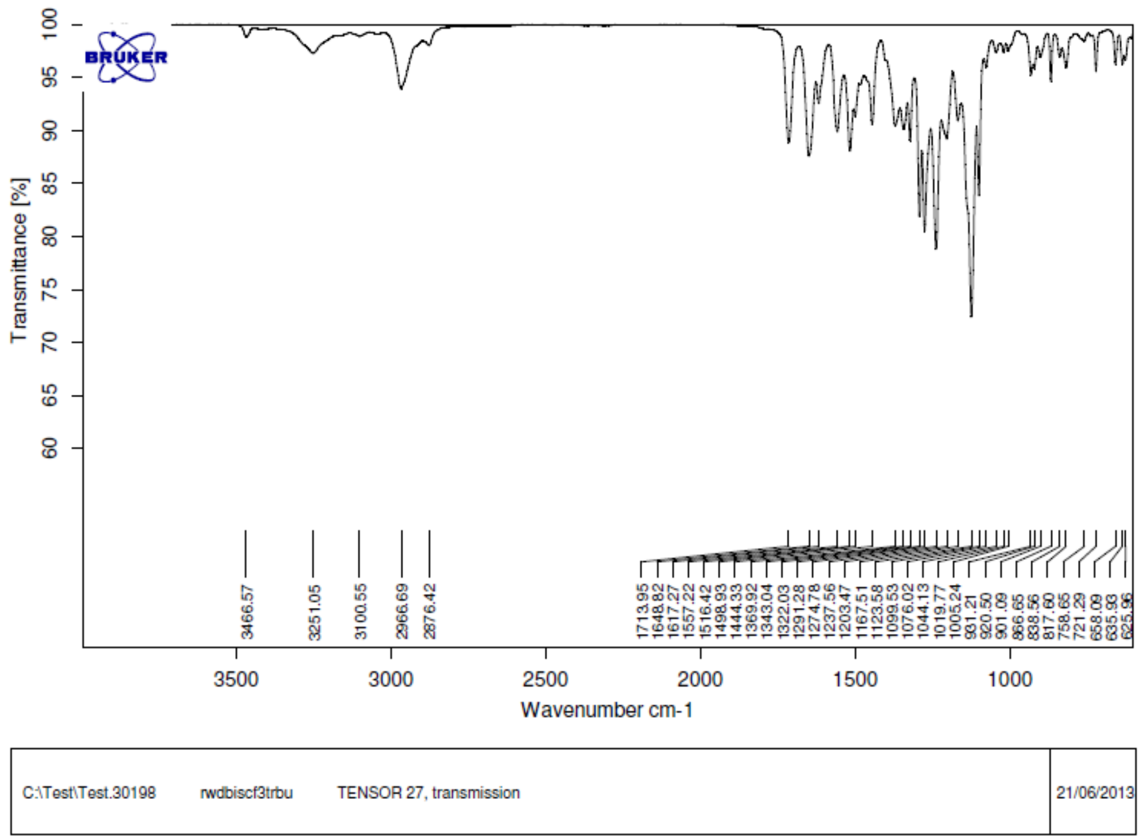

FT-IR of **33**.

Mass Spectrum SmartFormula Report

Analysis Info

Analysis Name	\\Utof\data\June 13\ESI41493_5_01_9335.d	Acquisition Date	24/06/2013 10:44:36
Method	2.5min_cal_sample_pos_naf_14-05-10 test.m	Operator	Mass Spec
Sample Name	ESI41493	Instrument / Ser#	micrOTOF 92
Comment			

Acquisition Parameter

Source Type	ESI	Ion Polarity	Positive	Set Nebulizer	2.0 Bar
Focus	Not active			Set Dry Heater	180 °C
Scan Begin	100 m/z	Set Capillary	4500 V	Set Dry Gas	10.0 l/min
Scan End	1000 m/z	Set End Plate Offset	-500 V	Set Divert Valve	Source

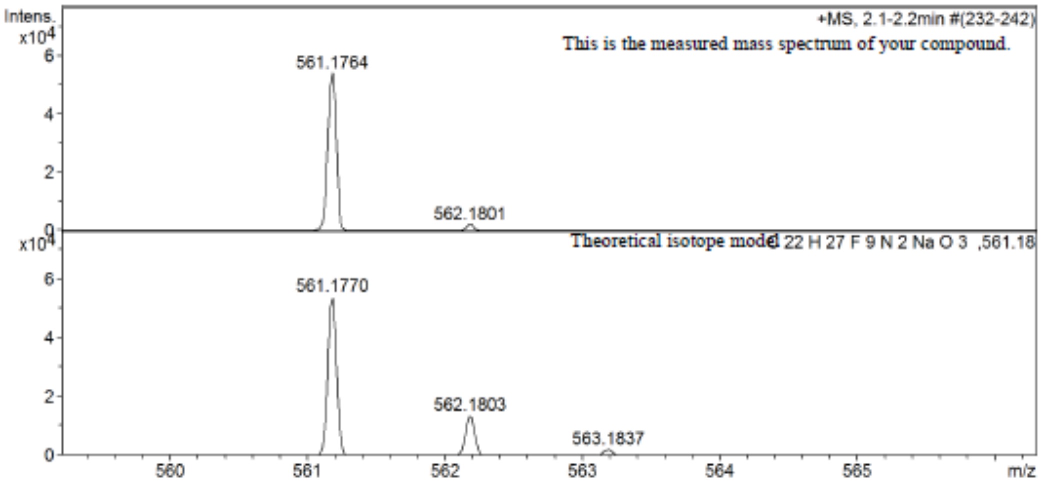

Meas. m/z	#	Formula	m/z	err [ppm]	Mean err [ppm]	rdb	e⁻ Conf	mSigma
561.1764								
	1	C 22 H 27 F 9 N 2 Na O 3	561.1770	1.1	1.0	5.5	even	121.52

High Resolution mass spectrum of **33**.

(S)-methyl (3,3-dimethyl-1-(2-(3,3,3-trifluoro-2-(trifluoromethyl)propanamido)-4-(trifluoromethyl)phenoxy)butan-2-yl)carbamate [34]

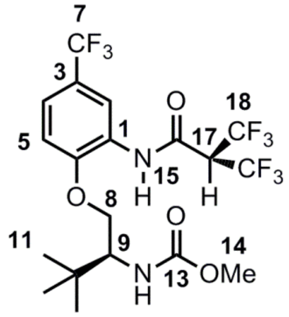

TFA (5mL) was added dropwise to a stirring solution of **8** (100 mg, 0.18 mmol) in DCM (5mL), at 0˚C and the reaction stirred for 1 hour. The mixture was concentrated in vacuo, suspended in Et_2O and 0.5mL HCl (2.0M in Et_2O) was added. The resultant white solid was filtered (*ca*. 75mg), DCM was added (50mL) and $NaHCO_3$ (50 mL, aq., saturated) was added. The organic phase was extracted, dried ($MgSO_4$) and concentrated to yield the free amine as a white solid which was used without further purification.

DMAP (2.0 mg) followed by methyl chloroformate (0.03 mL, 0.4 mmol) were added to a stirring solution of the crude free amine in DCM (7.0 mL) and DIPEA (0.5 mL). The reaction was stirred overnight before being concentrated in vacuo and the product dissolved in DCM (50 mL). The organic phase was washed with ammonium chloride solution (1 X 25 mL) and brine (1 X 25 mL). The resulting colorless oil was purified by column chromatography (7:1 PET:EtOAc) to yield 20 mg (0.039 mmols, 22 %) of the title compound.

δH (500 MHz, C_6D_6, 23˚C): 9.58 (1H, s, H15), 9.24 (1H, d, *J* 2 Hz, H2), 7.09 (1H, d, obscured, H4), 5.97 (1H, d, *J* 8 Hz, H5), 5.27 (1H, septet, *J* 7 Hz, H17), 4.36 (1H, d, *J* 9 Hz, H12), 3.53 (dt, J_1 10 Hz, J_2 2 Hz, H9), 3.42 (1H, dd, J_1 8 Hz, J_2 2 Hz, H8), 3.50 (3H, s, H 14), 3.04 (dd, J_1 11 Hz, J_2 8 Hz, H8), 0.47 (9H, s, H11).

δC (125 MHz, C_6D_6, 23˚C): 159.50 (C13), 157.42 (C16), 150.10 (C6), 125.55 (C1), 124.91 (C7, q $^1J_{13C-19F}$ = 272 Hz), 124.07 (C3, q, $^2J_{13C-19F}$ = 32 Hz), 122.50 (C20 and C20', qq, $^1J_{13C-19F}$ 282 Hz, Δ δ C20 and C20' = 0.15 ppm), 121.77 (C4, q, $^3J_{13C-19F}$ 4 Hz) 117.08 (C4, q, $^3J_{13C-19F}$ 4 Hz), 110.08 (C5), 70.36 (C8), 60.12 (C9), 55.19 (C19, $^2J_{13C-19F}$ = 30 Hz), 52.38 (C14), 32.20 (C10), 26.64 (C11).

δF {1H} (470 MHz, C_6D_6, 23˚C): -61.27 (F7), -63.75 (F20, q, $^4J_{19F-19F}$ 8 Hz), -64.09 (F20, q, $^4J_{19F-19F}$ 8 Hz).HRMS: (ES+): found 511.1298; Formula $C_{19}H_{20}F_9N_2NO_4$, [M - H+] requires 511.1285.

ν_{max} (neat, cm^{-1}): 3455.35, 3292.74, 2963.28, 1696.31, 1618.03, 1554.72, 1522.26, 1498.56, 1445.03, 1371.92, 1346.41, 1321.00, 1290.32, 1273.09, 1234.59, 1211.67, 1167.28, 1119.39, 1097.06, 1073.90, 1043.95, 1018.05, 969.17, 930.79, 919.45, 901.26, 866.74, 839.15, 815.07, 781.30, 758.23, 721.62, 656.94, 635.94, 623.83.

$[\alpha]_D^{25.0}$ +57.0° (c = 0.1, $CHCl_3$).

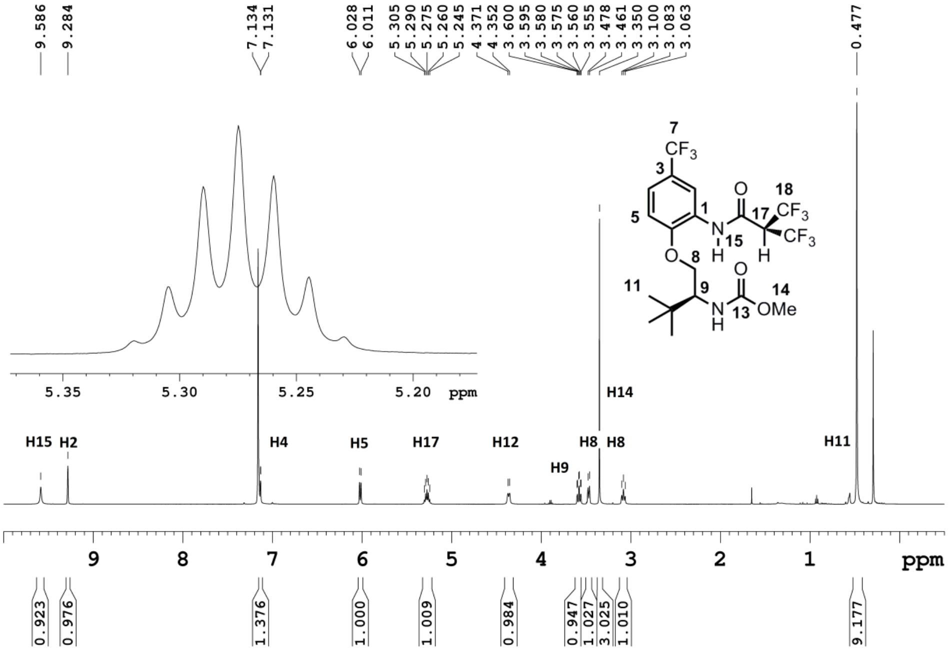

^{1}H NMR spectrum of **34** (23˚C, C_6D_6, 500 MHz).

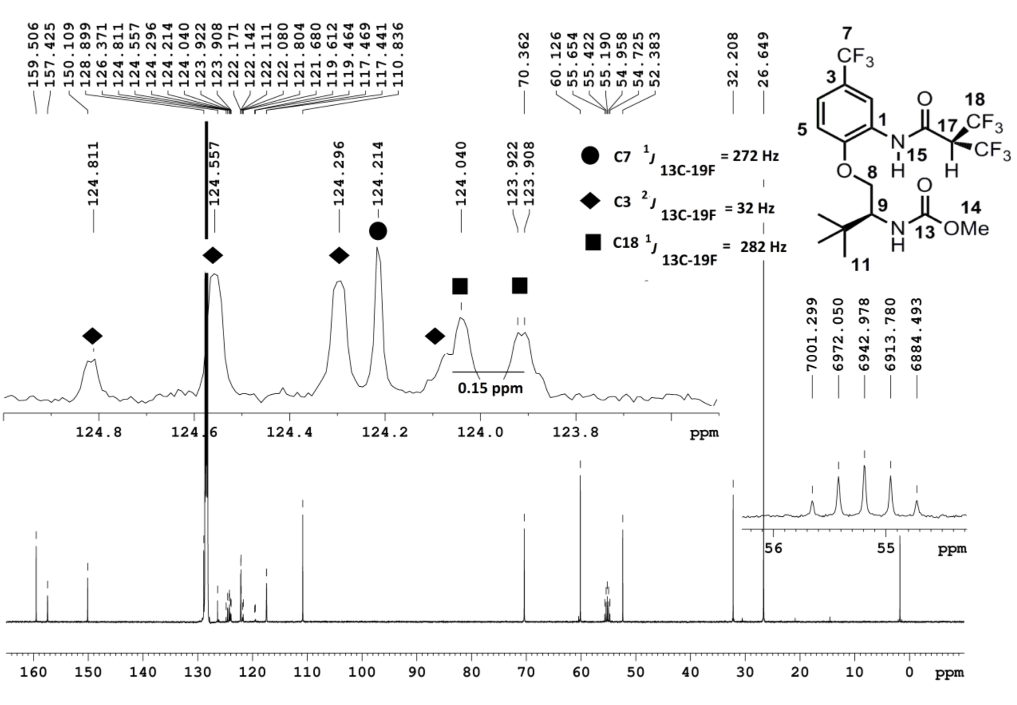

^{13}C NMR spectrum of **34** (23˚C, C_6D_6, 125 MHz).

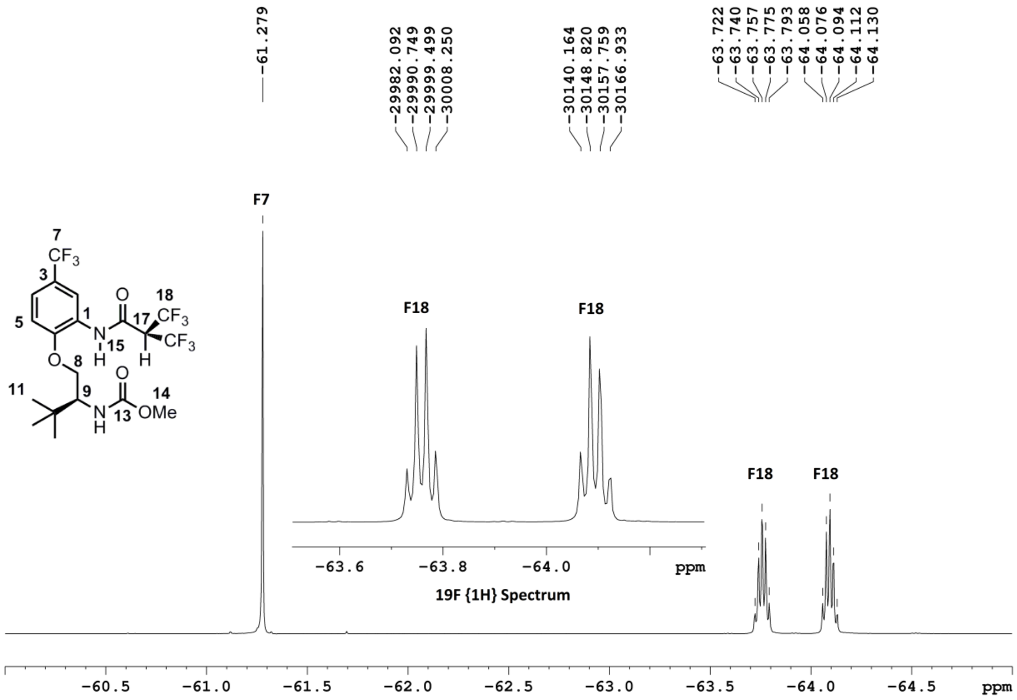

^{19}F NMR spectrum of **34** (23˚C, C_6D_6, 470 MHz). Insert shows ^{19}F {^1H} spectrum and doublet of quartets splitting pattern resulting from anisochronous sets of fluorine atoms.

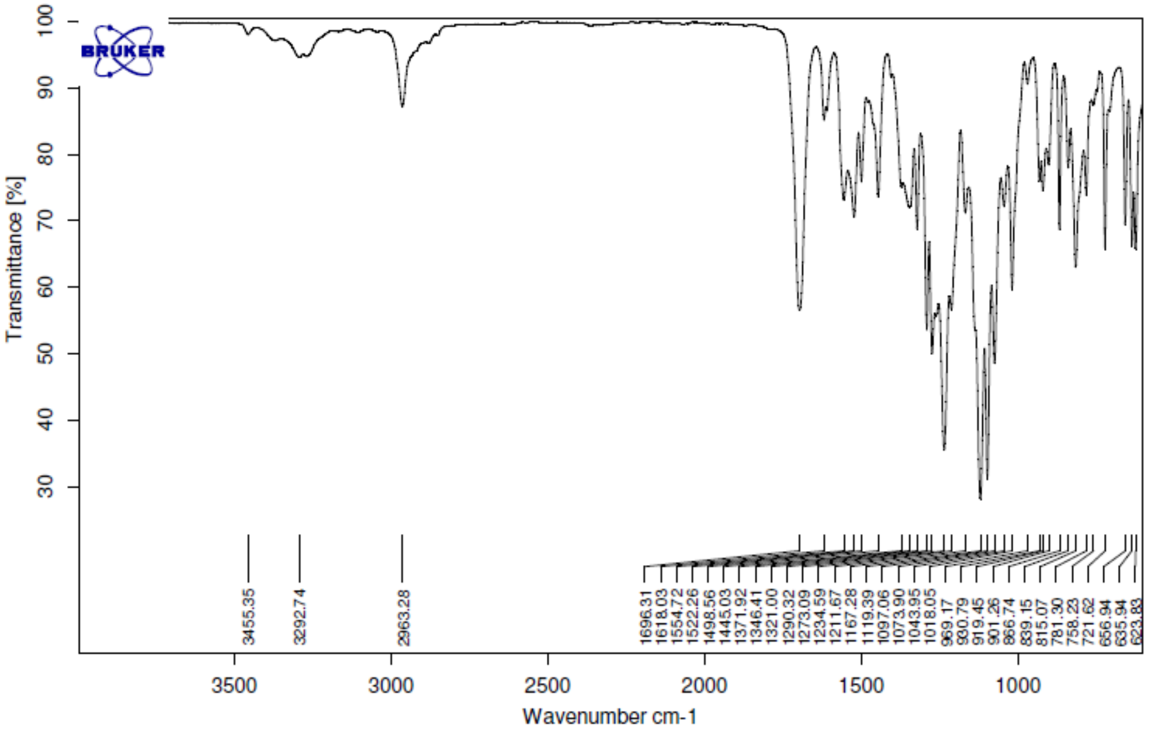

FT-IR of **34**.

Mass Spectrum SmartFormula Report

Analysis Info

Analysis Name	\\Utof\Data\Aug12\ESI36076n_8_01_45712.d	Acquisition Date	02/08/2012 10:53:30
Method	2.5min_cal_sample_neg_Naf_24-01-11.m	Operator	Mass Spec
Sample Name	ESI36076n	Instrument / Ser#	micrOTOF 92
Comment			

Acquisition Parameter

Source Type	ESI	Ion Polarity	Negative	Set Nebulizer	2.0 Bar
Focus	Not active			Set Dry Heater	180 °C
Scan Begin	50 m/z	Set Capillary	4500 V	Set Dry Gas	10.0 l/min
Scan End	1200 m/z	Set End Plate Offset	-500 V	Set Divert Valve	Source

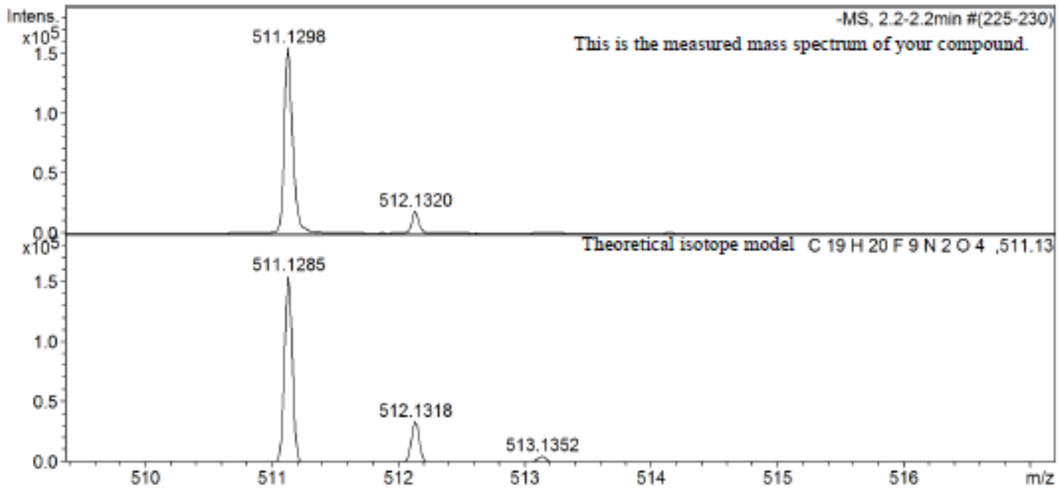

Meas. m/z	#	Formula	m/z	err [ppm]	Mean err [ppm]	rdb	N-Rule	e⁻ Conf	mSigma
511.1298									
	1	C 19 H 20 F 9 N 2 O 4	511.1285	-2.5	-2.2	6.5	ok	even	57.19

High-Resolution mass spectrum of **34**.

(S)-N-(2-(2-(3-ethylureido)-3,3-dimethylbutoxy)-5-(trifluoromethyl)phenyl)-3,3,3-trifluoro-2-(trifluoromethyl)propanamide [35]

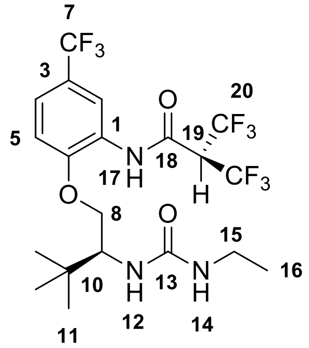

TFA (5mL) was added dropwise to a stirring solution of **8** (100 mg, 0.18 mmol) in DCM (5mL), at 0˚C and the reaction stirred for 1 hour. The mixture was concentrated in vacuo, suspended in Et_2O and 0.5mL HCl (2.0M in Et_2O) was added. The resultant white solid was filtered (*ca*. 75mg), DCM was added (50mL) and $NaHCO_3$ (50 mL, aq., saturated) was added. The organic phase was extracted, dried ($MgSO_4$) and concentrated to yield the free amine as a white solid which was used without further purification.

The crude free amine was stirred overnight in ethyl isocyanate (1.0 mL) before being concentrated and the resulting solid was purified by column chromatography (5:1 PET:EtOAc) to yield 25 mg (0.047 mmols, 26 %) of the title compound.

δH (500 MHz, C_6D_6, 23˚C, very low solubility): 10.18 (1H, s, H17), 9.35 (1H, s, H2), 7.13 (1H, obscured, H4), 6.09 (1H, d, *J* 9 Hz, H5), 5.72 (1H, hept, $^3J_{1H\text{-}19F}$ 7 Hz, H19), 3.81 (1H, td, J_1 10 Hz, J_2 2 Hz, H9), 3.55 (1H, dd, J_1 9 Hz, J_2 2 Hz, H8), 3.32 (1H, d, *J* 10 Hz, H12), 3.18 (1H, t, *J* 9 Hz, H8), 3.09 (1H, t, *J* 5 Hz, H14), 2.82 (2H, m, H15), 0.74 (3H, t, *J* 8 Hz, H16), 0.57 (9H, s, H11).

δH (500 MHz, $CDCl_3$, 23˚C): 9.98 (1H, s, H17), 8.72 (1H, d, *J* 2 Hz, H2), 7.30 (1H, dd, J_1 8 Hz, J_2 1 Hz, H4), 6.82 (1H, d, *J* 8 Hz, H5), 5.16 (1H, hept, $^3J_{1H\text{-}19F}$ 7 Hz, H19), 4.54 (1H, d, *J* 9 Hz, H12), 4.51 (1H, t, *J* 5 Hz, H14), 4.24 (1H, dd, J_1 8.6 Hz, J_2 2 Hz, H8), 4.02 (1H, dt, J_1 9 Hz, J_2 2.0 Hz, H9), 3.86 (1H, m, H8), 3.19 (1H, m, H15), 3.10 (1H, m, H15), 1.09 (3H, t, J_1 6 Hz, H16), 1.01 (9H, s, H11).

δC (125 MHz, $CDCl_3$, 23˚C): 171.64 (C18), 159.69 (C13), 158.06 (C6), 128.43 (C1), 124.29 (C7, q $^1J_{13C\text{-}19F}$ = 271 Hz), 123.74 (C3, q, $^2J_{13C\text{-}19F}$ = 32 Hz), 121.91 (C20 and C20', qq, $^1J_{13C\text{-}19F}$ 281 Hz, Δ δ C20 and C20' = 0.23 ppm), 122.12 (C4, q, $^3J_{13C\text{-}19F}$ 4 Hz) 117.15 (C4, q, $^3J_{13C\text{-}19F}$ 4 Hz), 111.01 (C5), 71.31 (C9), 58.77 (C8), 54.07 (C19, $^2J_{13C\text{-}19F}$ = 29 Hz), 35.69 (C15), 32.71 (C10), 27.12 (C11), 15.15 (C16).

δF {1H} (470 MHz, C_6D_6, 23˚C): -62.25 (F7), -63.62 (F20, q, $^4J_{19F\text{-}19F}$ 9 Hz), -64.00 (F20, q, $^4J_{19F\text{-}19F}$ 9 Hz).

HRMS: (ES+): found 548.1563; Formula $C_{20}H_{24}F_9N_3NaO_3$, [M + H+] requires 548.1566

ν_{max} (neat, cm^{-1}): 3414.10, 3242.87, 2967.86, 2361.65, 1698.45, 1651.38, 1618.74, 1558.57, 1499.50, 1443.80, 1371.35, 1338.46, 1321.56, 1290.97, 1273.58, 1237.68, 1213.07, 1167.88, 1123.48, 1099.20, 1077.25, 1043.93, 1029.63, 1007.04, 930.63, 919.75, 866.52, 839.24, 817.56,

768.17, 735.34, 721.98.

MP: 209-211 C.

$[\alpha]_D^{25.0}$ +22.0° (c = 0.15, $CHCl_3$).

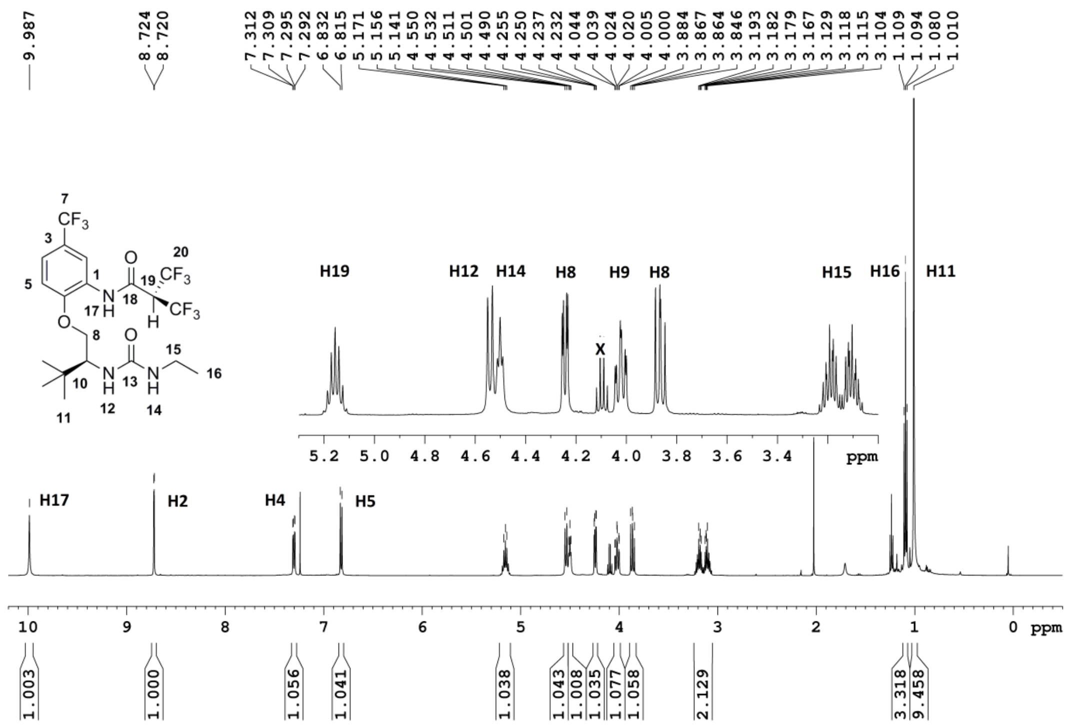

^{1}H NMR spectrum of **35** (23˚C, $CDCl_3$, 500 MHz).

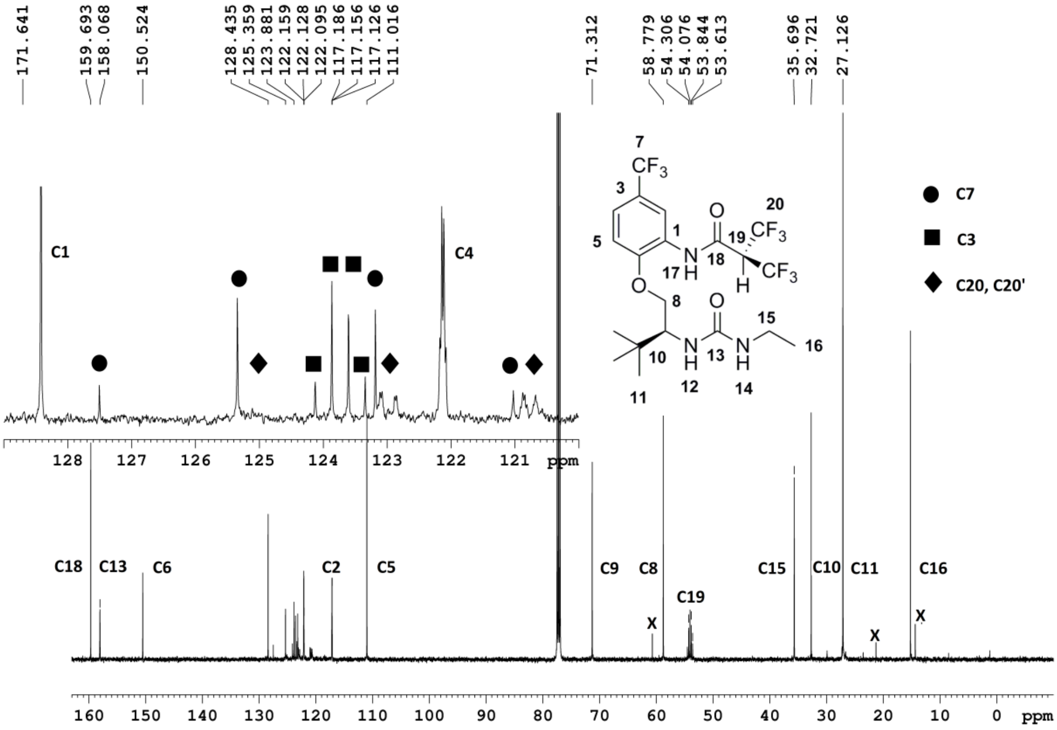

^{13}C NMR spectrum of **35** (23˚C, $CDCl_3$, 125 MHz).

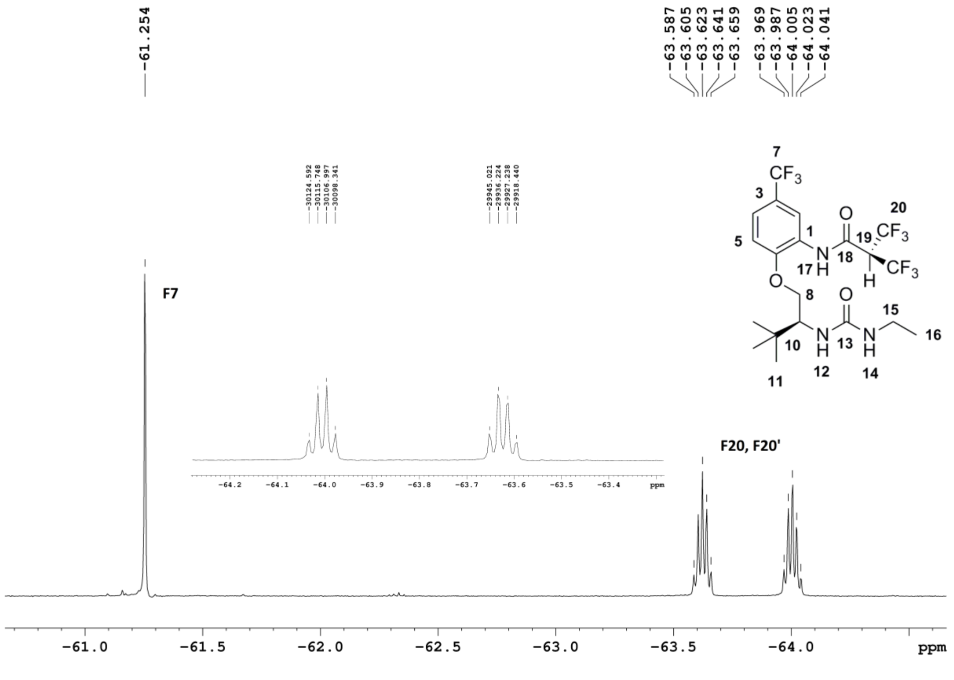

^{19}F NMR spectrum of **35** (23˚C, $CDCl_3$, 470 MHz).

Mass Spectrum SmartFormula Report

Analysis Info			
Analysis Name	D:\utofdata\ESI30809_9_01_33855.d	Acquisition Date	25/10/2011 07:53:40
Method	2.5min_cal_sample_pos_Naf_11-10-10.m	Operator	Mass Spec
Sample Name	ESI30809	Instrument / Ser#	micrOTOF 92
Comment			

Acquisition Parameter

Source Type	ESI	Ion Polarity	Positive	Set Nebulizer	2.0 Bar
Focus	Not active			Set Dry Heater	180 °C
Scan Begin	100 m/z	Set Capillary	4500 V	Set Dry Gas	10.0 l/min
Scan End	1500 m/z	Set End Plate Offset	-500 V	Set Divert Valve	Source

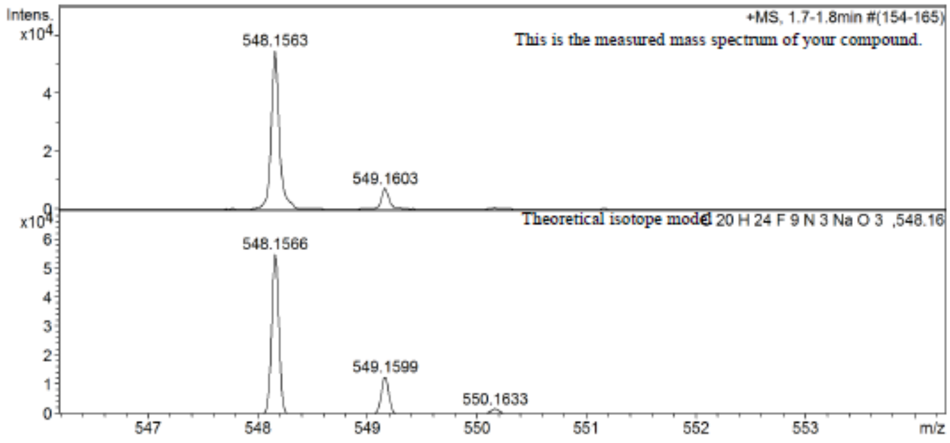

Meas. m/z	#	Formula	m/z	err [ppm]	Mean err [ppm]	rdb	e⁻ Conf	mSigma
548.1563								
	1	C 20 H 24 F 9 N 3 Na O 3	548.1566	0.6	0.5	5.5	even	57.93

High Resolution mass spectrum of **35**.

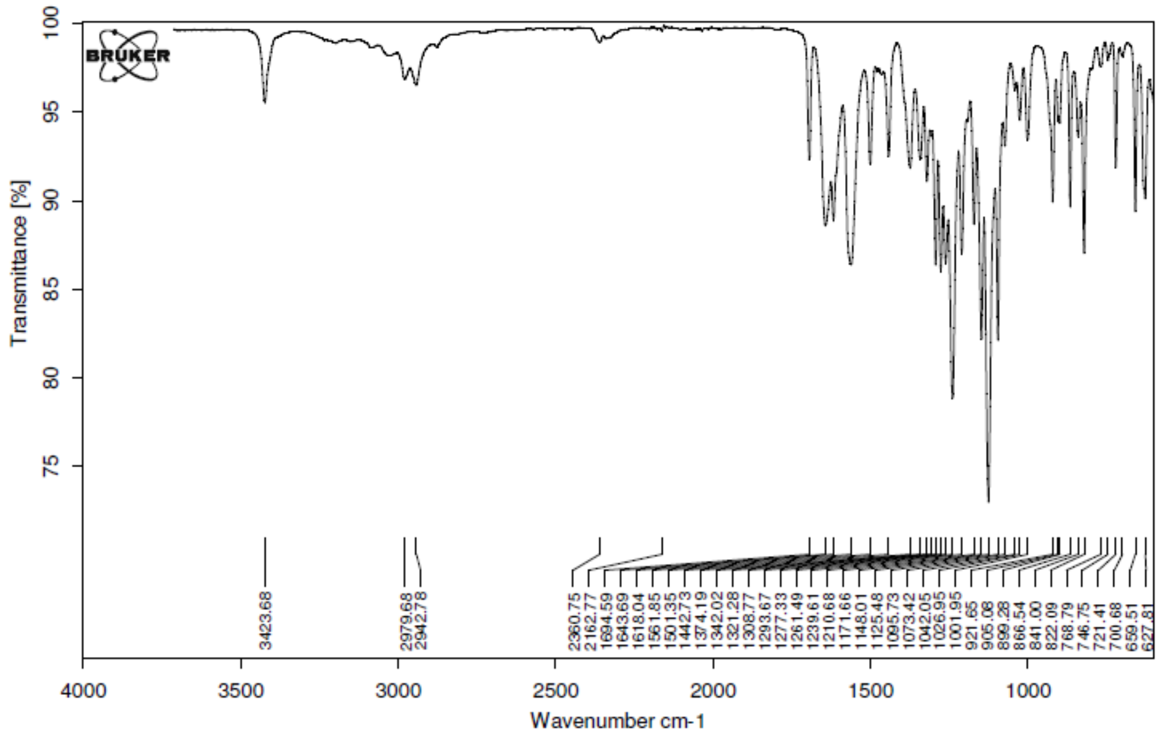

FT-IR of **35**.

3.2 Hydrogen/Deuterium Isotope Exchange Experiments

(S)-tert-butyl (3,3-dimethyl-1-(2-(3,3,3-trifluoro-2-(trifluoromethyl)propanamido)-4-(trifluoromethyl)phenoxy)butan-2-yl)carbamate

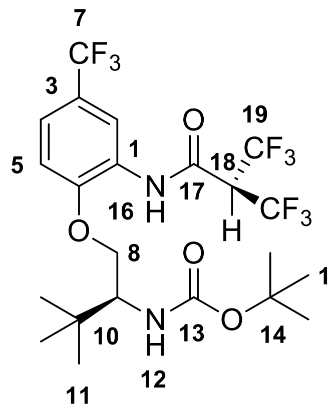

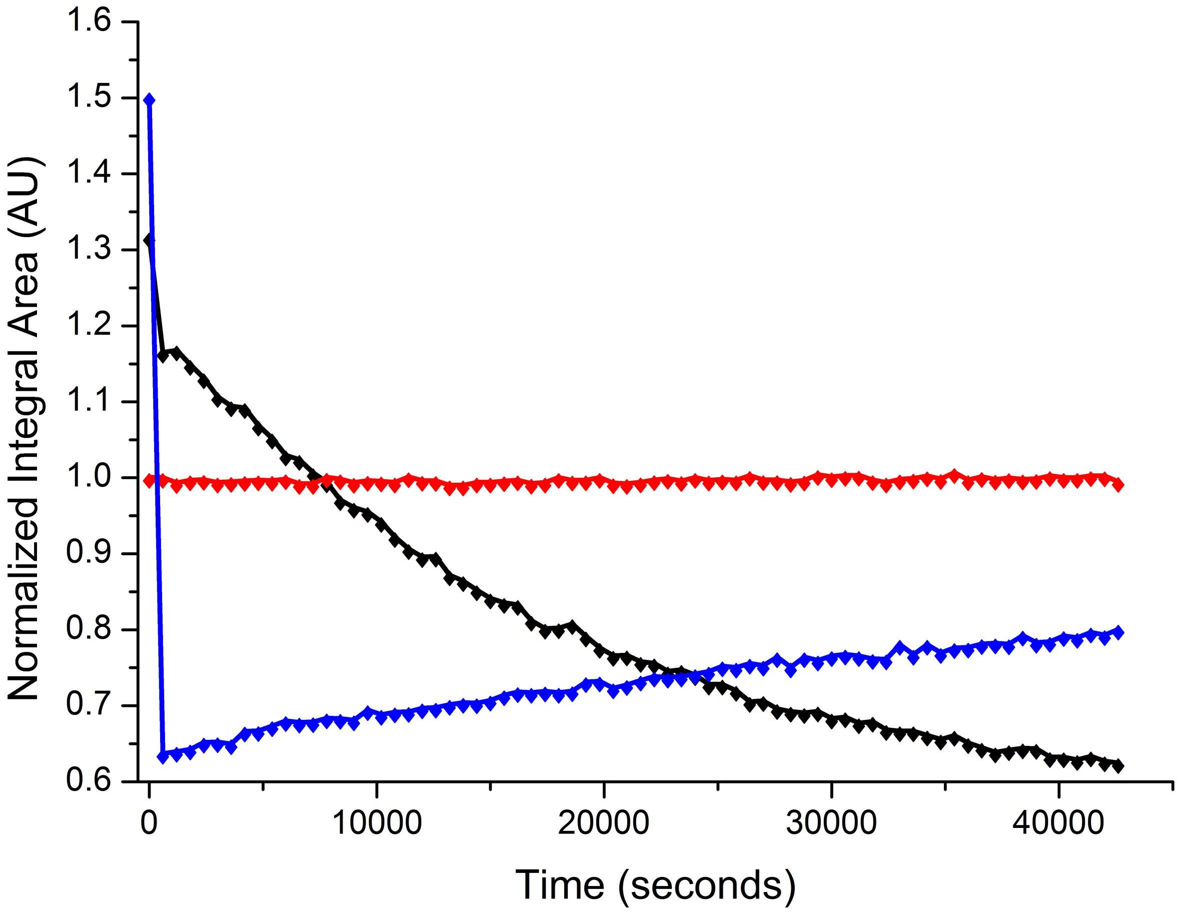

Hydrogen/Deuterium isotope exchange curves of the H2 proton (●), H12 proton (●) and H16 proton(●). The H16 proton, participating in a N-H···O hydrogen bond, exchanges more slowly than the H12 proton.

3.4 Variable Temperature ^{1}H and ^{19}F NMR experiments

3.4.1

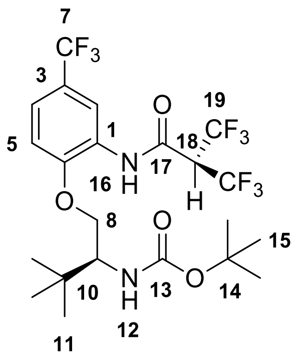

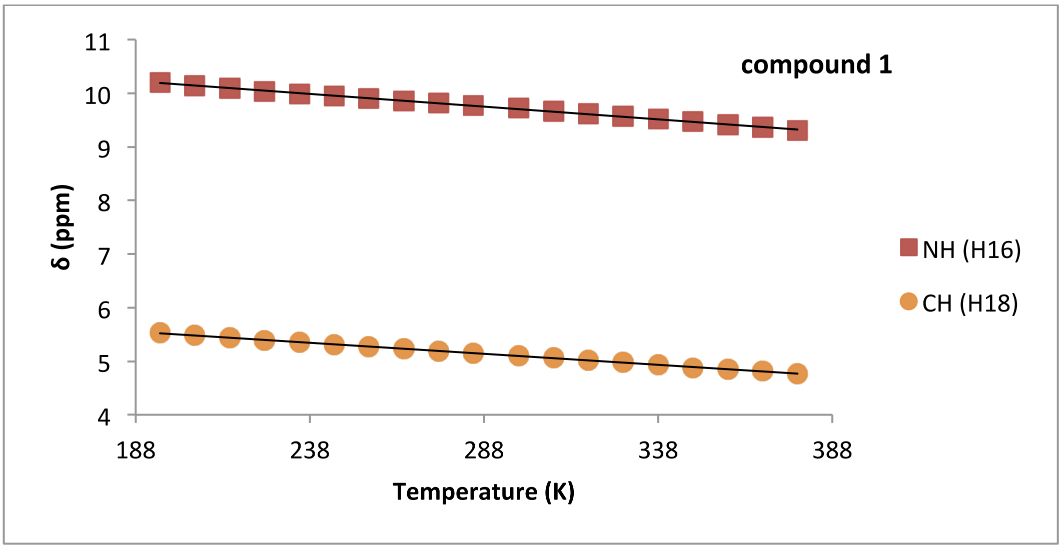

Variable Temperature plot for the ^{1}H resonances of **1** in Toluene-d_8

Temp (K)	NH (H16)	CH (H18)
298	9.72	5.10
308	9.67	5.06
318	9.62	5.02
328	9.57	4.98
338	9.52	4.93
348	9.47	4.88
358	9.41	4.85
368	9.36	4.81
	-5.1 ppb/K	-4.1 ppb/K

Variable temperature chemical shift data for **1**

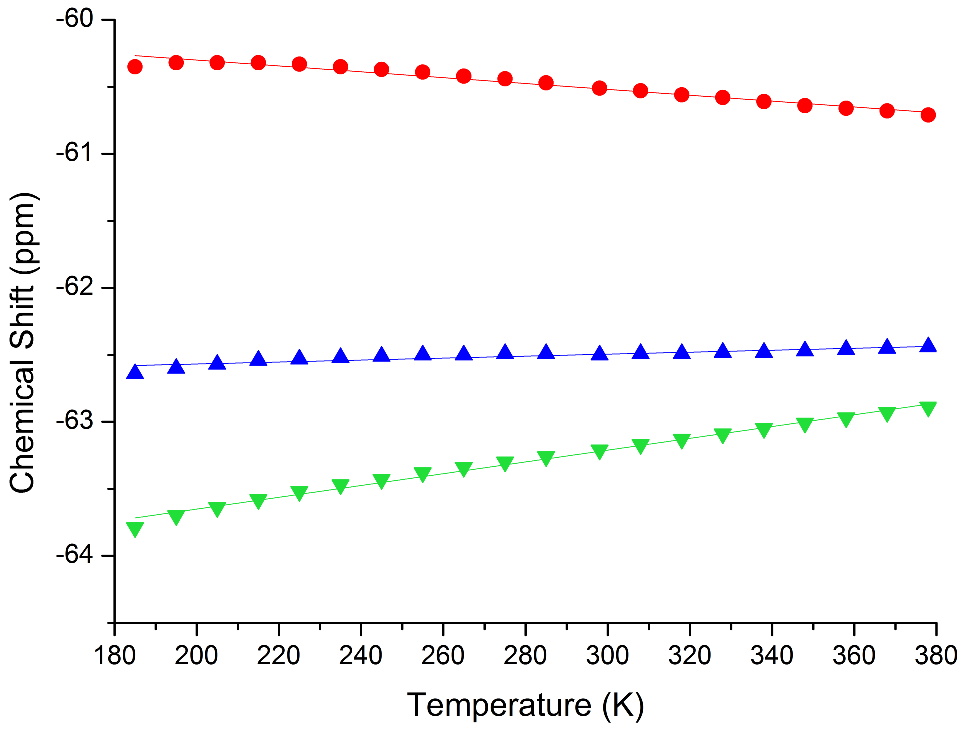

Variable Temperature plot for the ^{19}F resonances of **1** in Toluene-d_8 (● F7, Δ F19 and Δ F19') best fit lines.

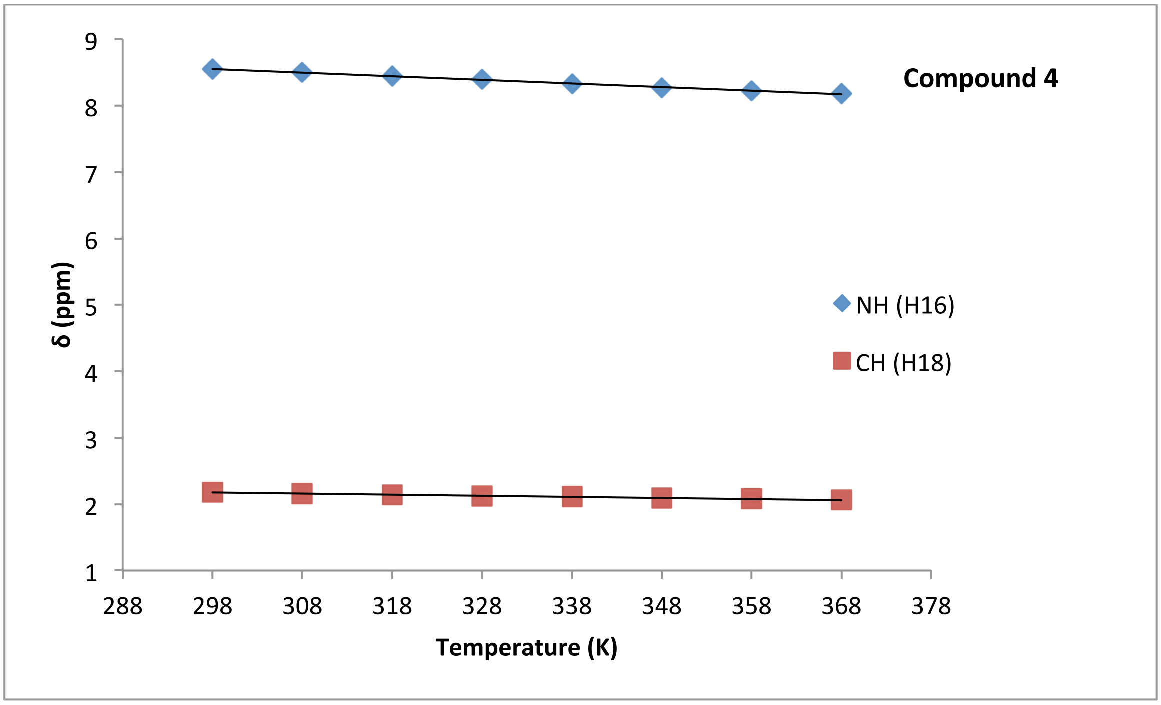

Variable Temperature plot for the ^{1}H resonances of **4** in Toluene-d_8

Temp (K)	NH (H16)	CH (H18)
298	8.55	2.18
308	8.5	2.16
318	8.44	2.14
328	8.39	2.12
338	8.33	2.11
348	8.27	2.09
358	8.22	2.08
368	8.18	2.06
	-5.2 ppb/K	-1.7 ppb/K

Variable temperature chemical shift data for **4**

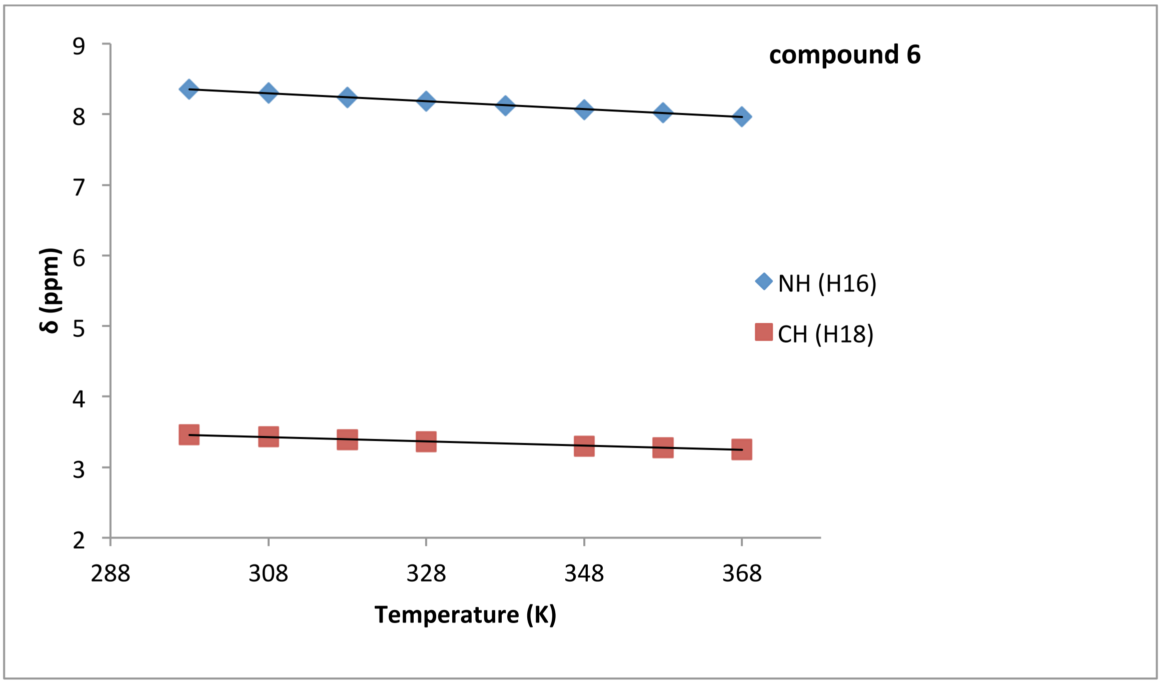

Variable Temperature plot for the ^{1}H resonances of **6** in Toluene-d_8

Temp (K)	NH (H16)	CH (H18)
298	8.36	3.46
308	8.3	3.43
318	8.24	3.39
328	8.18	3.36
338	8.12	-
348	8.07	3.3
358	8.02	3.28
368	7.97	3.25
	-5.5 ppb/K	-3.0 ppb/K

Variable temperature chemical shift data for **6**

3.4.2

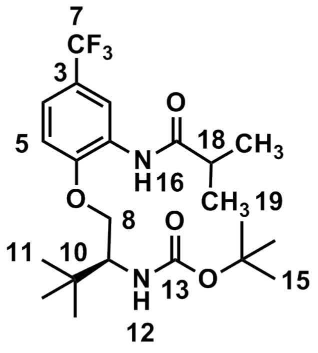

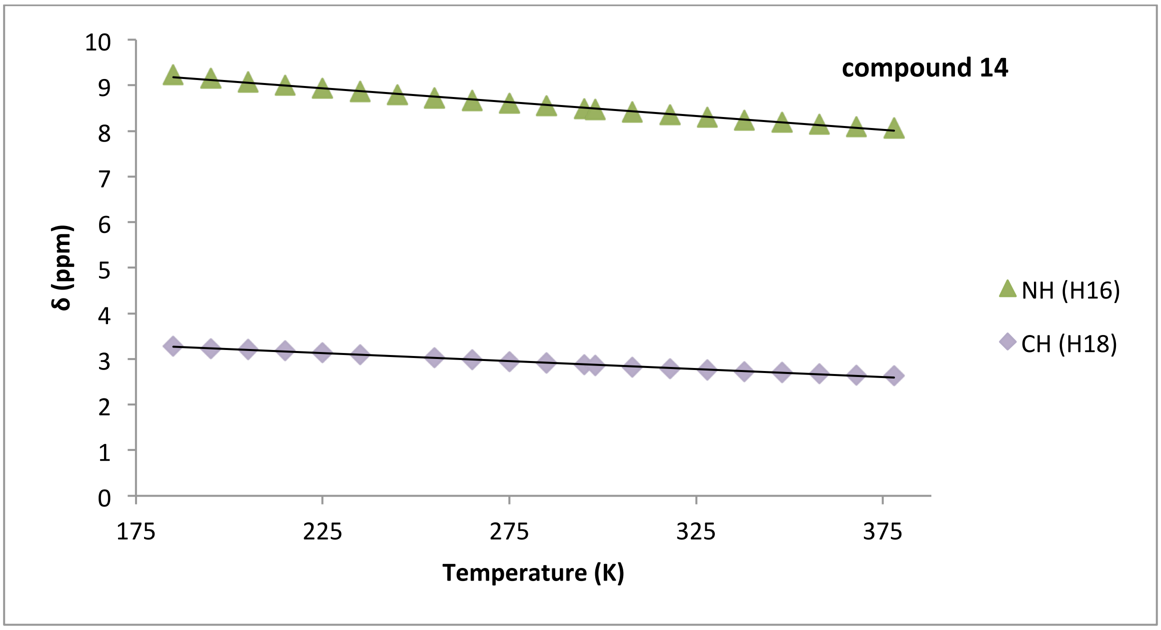

Variable Temperature plot for the ^{1}H resonances of **14** in Toluene-d_8

Temp (K)	NH (H16)	CH (H18)
298	8.47	2.86
308	8.42	2.82
318	8.36	2.79
328	8.30	2.76
338	8.24	2.72
348	8.19	2.70
358	8.15	2.67
368	7.10	2.65
	-5.2 ppb/K	-3.0 ppb/K

Variable temperature chemical shift data for **14**

3.5 Quantitative homonuclear NOE experiments

3.5.1

(S)-tert-butyl (3,3-dimethyl-1-(2-(3,3,3-trifluoro-2-(trifluoromethyl)propanamido)-4-(trifluoromethyl)phenoxy)butan-2-yl)carbamate

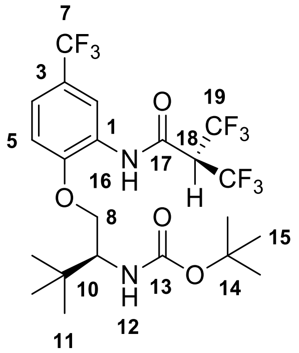

3.5.1.2 NOE experiments in C_6D_6

Summary

NOE buildup curves were obtained for the H18-H16 in C_6D_6 at 25C protons by irradiating the H18 proton and for the H8 geminal protons by irradiating the H8 proton farthest upfield.

The H18-H16 NOE build up curve is linear from 20ms to 500ms (**Figure 1**). Unfortunately, the H8 geminal proton NOE curve (**Figure 2**) shows two distinct regions (due to selective polarization transfer?): a steep slope with negative integrals at < 100ms (**Figure 4**) and a less steep slope with positive integrals at > 100ms (**Figure 3**).

X-ray Crystal Structure Distance H18-H16: **2.14 Å**.

NOE-calculated distance H18-H16 using global H8 slope: **1.83 Å**.

NOE-calculated distance H18-H16 using H8 slope > 100ms **1.84 Å**

NOE-calculated distance H18-H16 using H8 slope < 100 ms: **2.12 Å**.

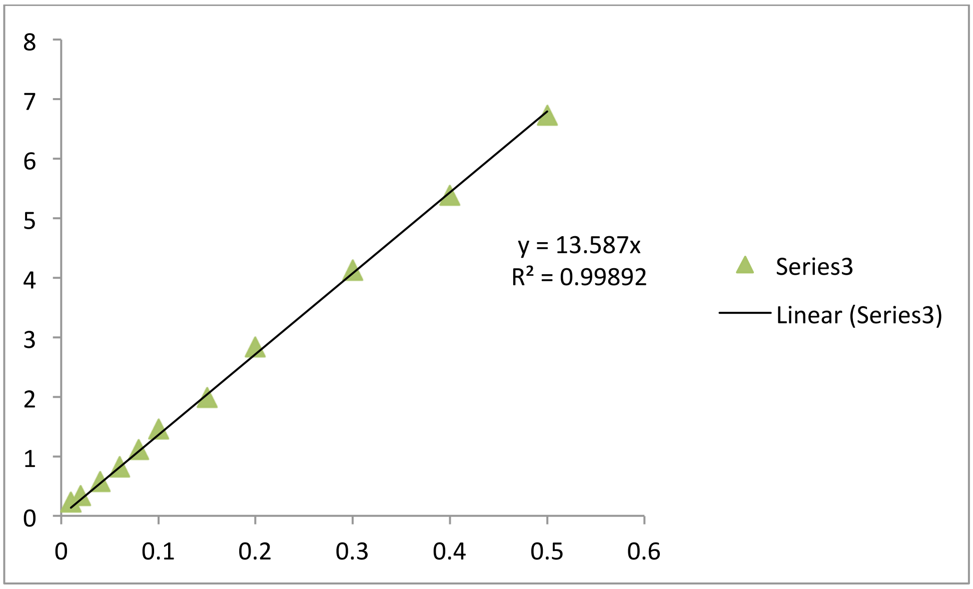

Figure 1. H18-H16 NOE buildup curve linear fit.

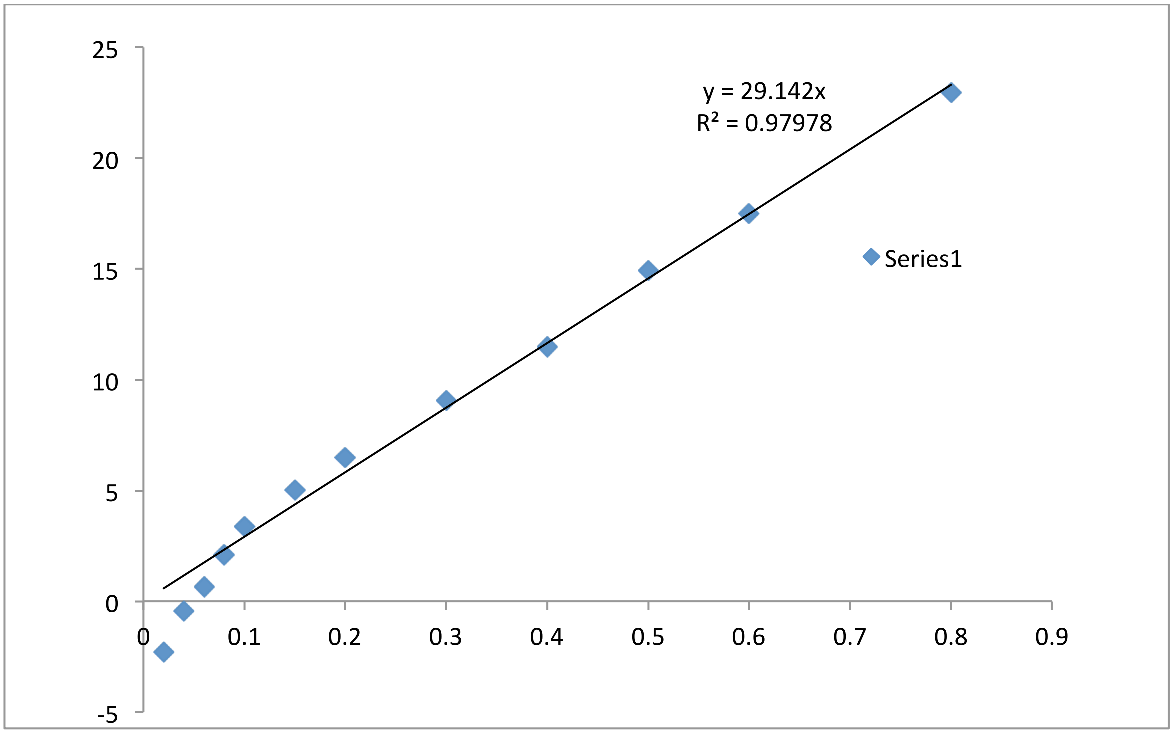

Figure 2. H8→H8' NOE buildup curve global linear fit.

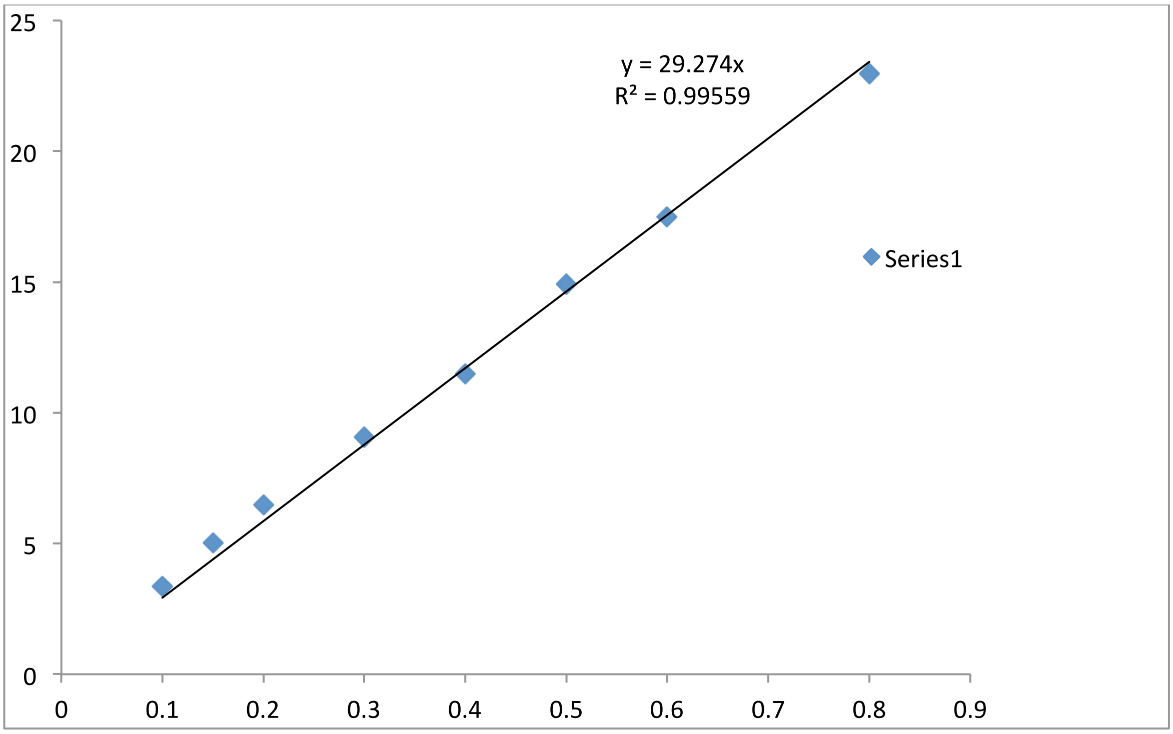

Figure 3. H8→H8' > 100msec NOE buildup curve linear fit.

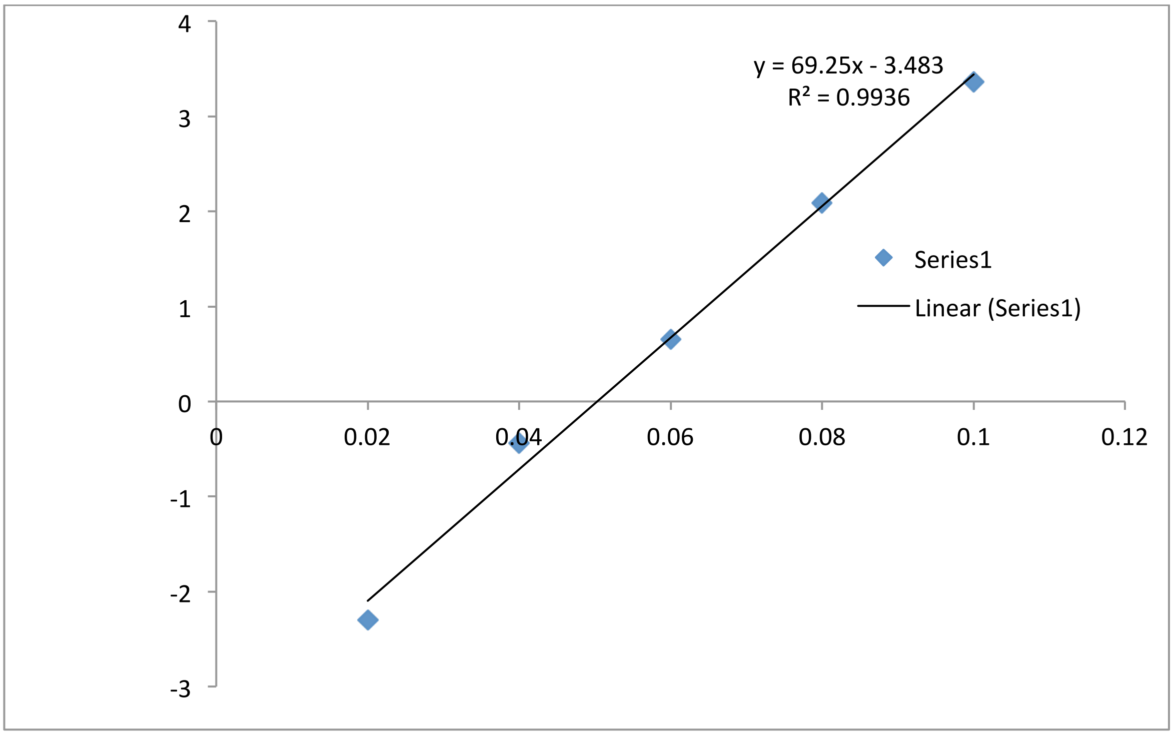

Figure 4. H8→H8’ < 100 msec NOE buildup curve fit.

3.5.1.3 NOE experiments in CCl_4

Summary

NOE buildup curves were obtained for the H18, H16 and H9 protons in CCl_4 at 25C. Two different NOE buildup curves were obtained for each of the H8/H8’ geminal protons.

The H16→H18 and H18→H16 NOE buildup curves look as expected (**Figure1**, **Figure 2**). Unfortunately, both the H8→H8’ and H8’→H8 NOE build up curves are negative from 10ms to 80ms (**Figure 3**), although the slopes are nearly identical for the global fit (**Figure 3**) and d8 > 100msec fit (**Figure 4**).

Calculated NOE distance H18→H16 in CCl_4 (average of two): **1.85 Å**. Xray Distance: **2.14 Å**.

Calculated NOE distance H9→H12 in CCl4: **2.71 Å**. Xray Distance: **2.70 Å.**

Calculated NOE distance H8→H12 in CCl4: **2.30 Å**. Xray Distance: **2.67 Å.**

Calculated NOE distance H8→H16 in CCl4: **3.30 Å**. Xray Distance: **3.61 Å.**

Calculated NOE distance H9→H16 in CCl4: **2.70 Å**. Xray Distance: **2.48 Å.**

Calculated NOE distance H9→H8 in CCl4: **2.28 Å**. Xray Distance: **2.45 Å.**

H8 →H12, H9→H18, H8 →H9 cannot be observed.

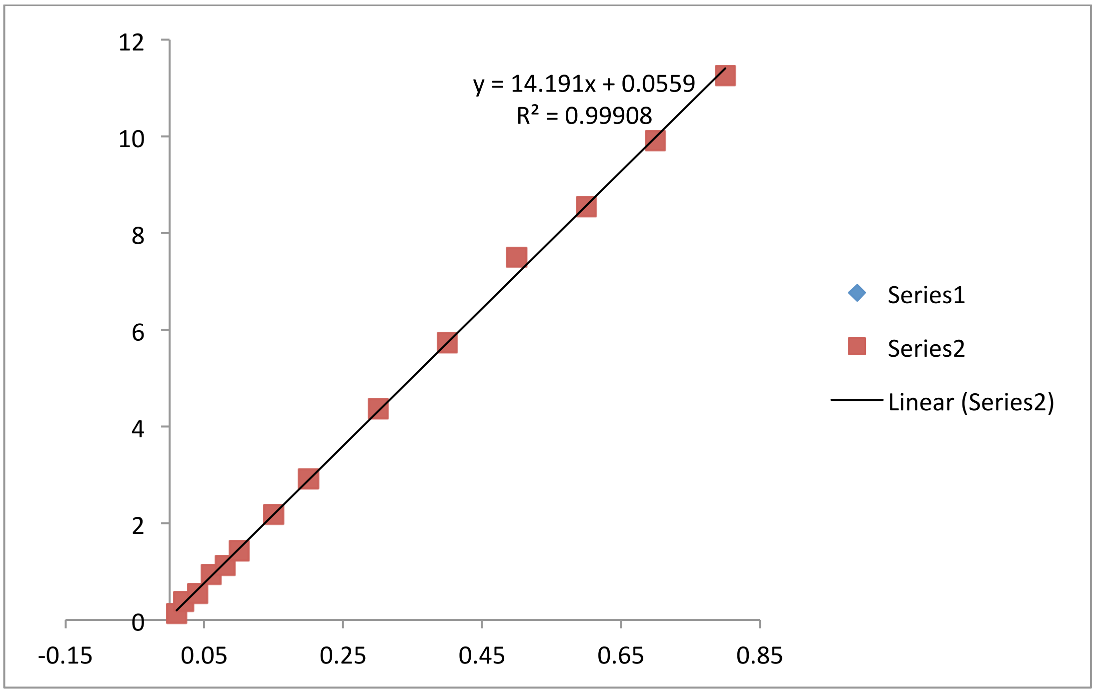

Figure 1. NOE buildup curve of H18→H16.

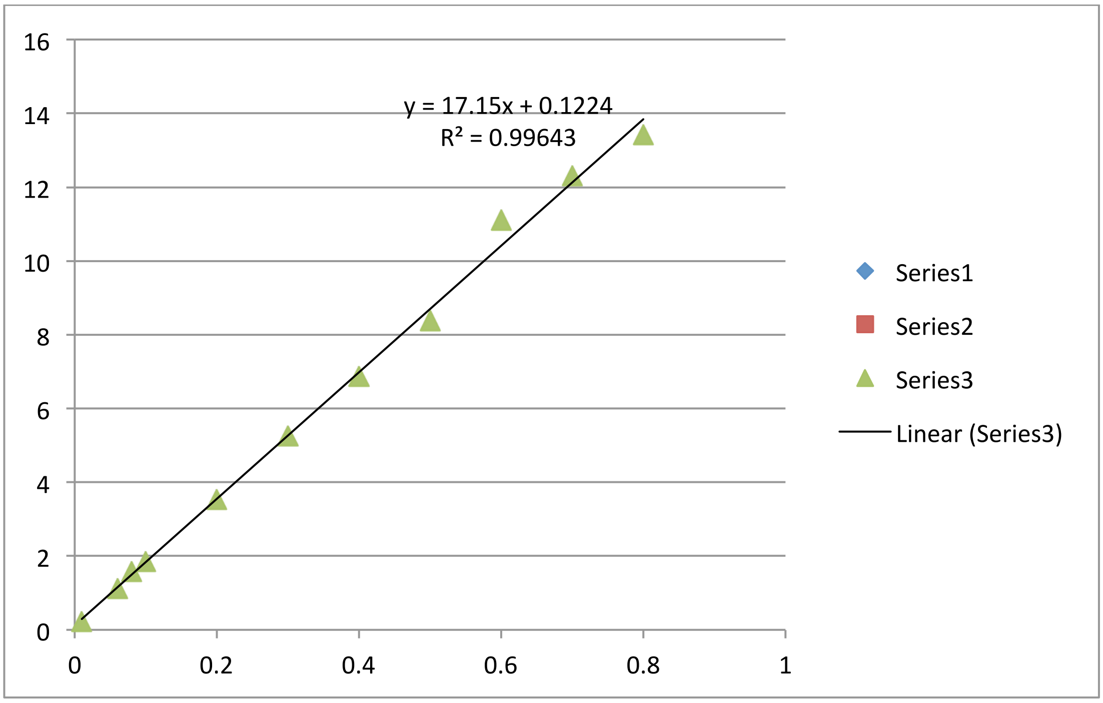

Figure 2. NOE buildup curve of H16→H18.

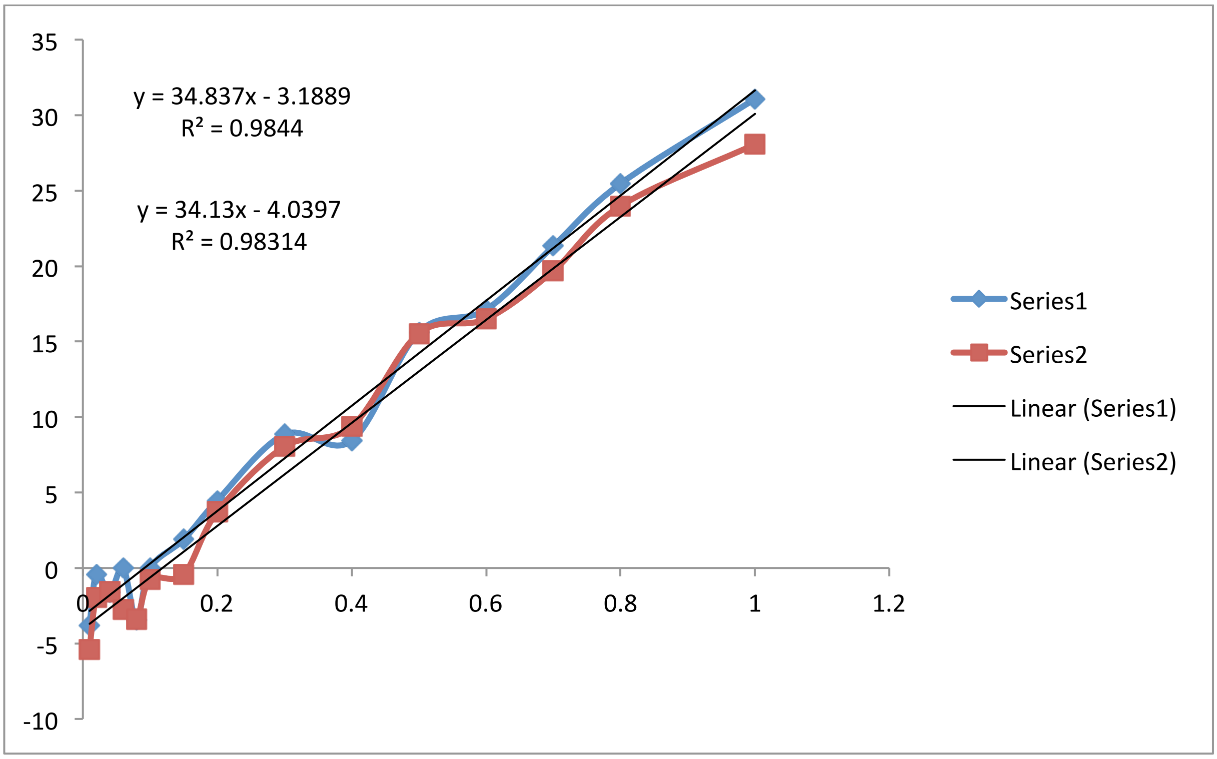

Figure 3. NOE buildup curve of H8→H8' (blue) and H8'→H8 (maroon).

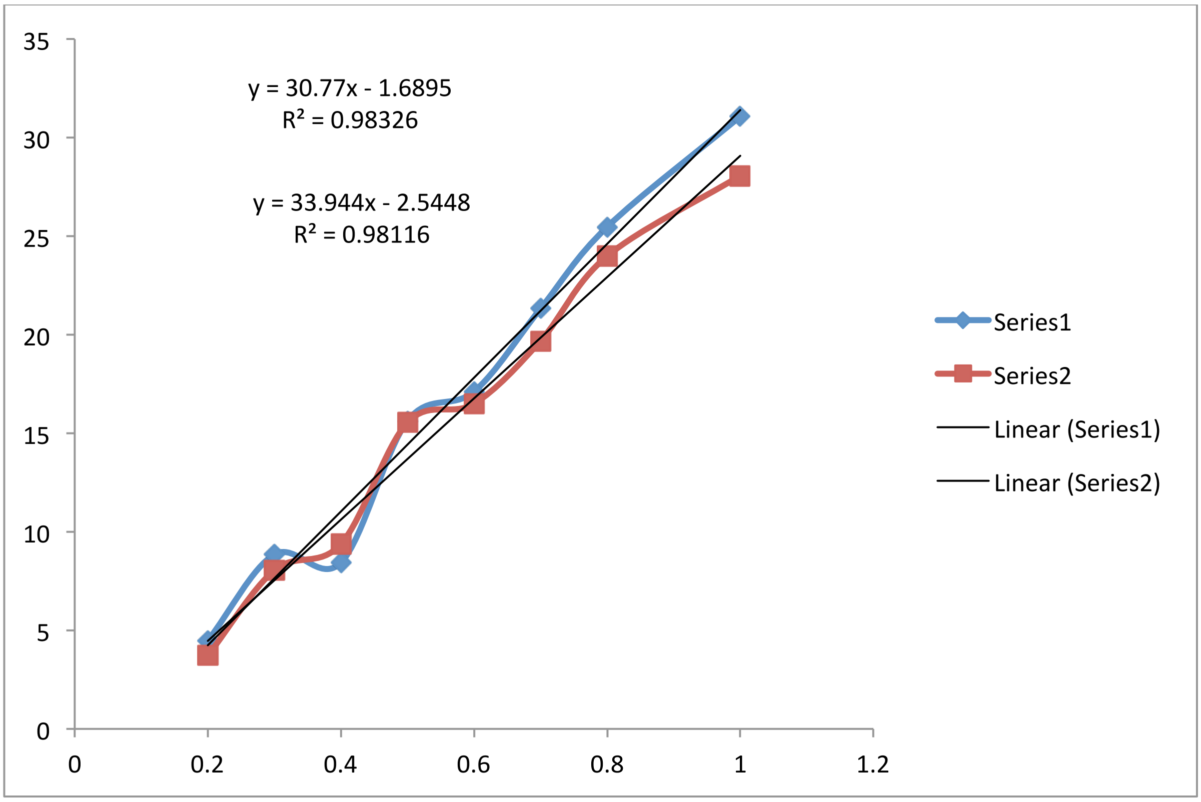

Figure 4. NOE buildup curve of H8→H8' (blue) and H8'→H8 (maroon) at d8 > 150ms.

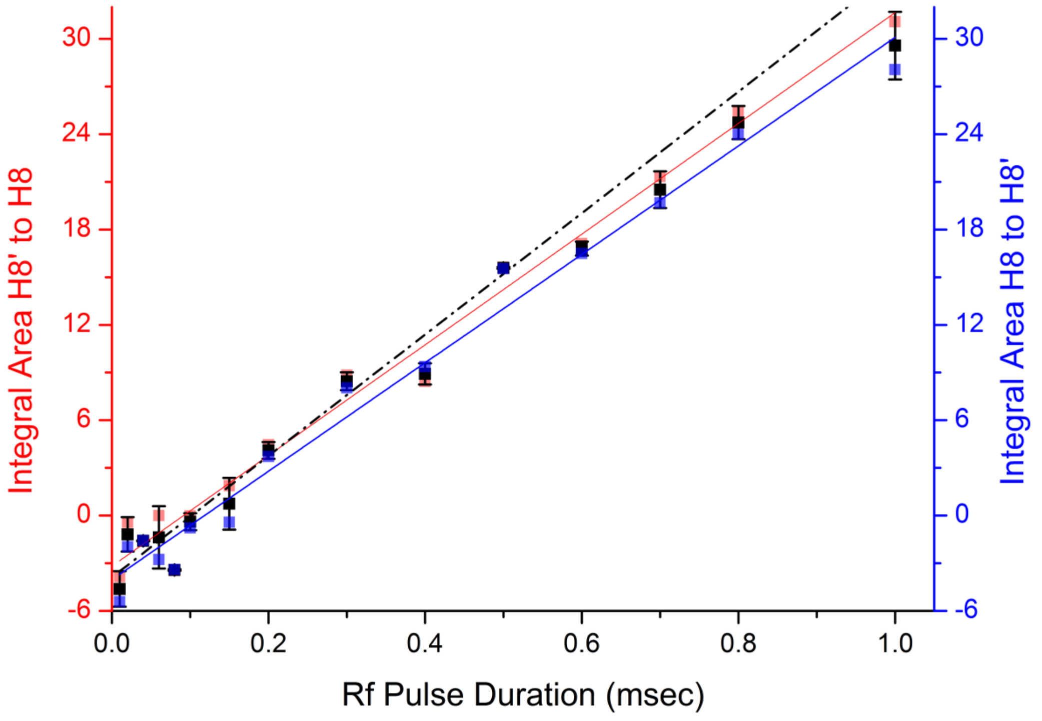

Figure

Figure 5. NOE buildup curve of H8→H8' (blue) and H8'→H8 (maroon) with best-fit lines.

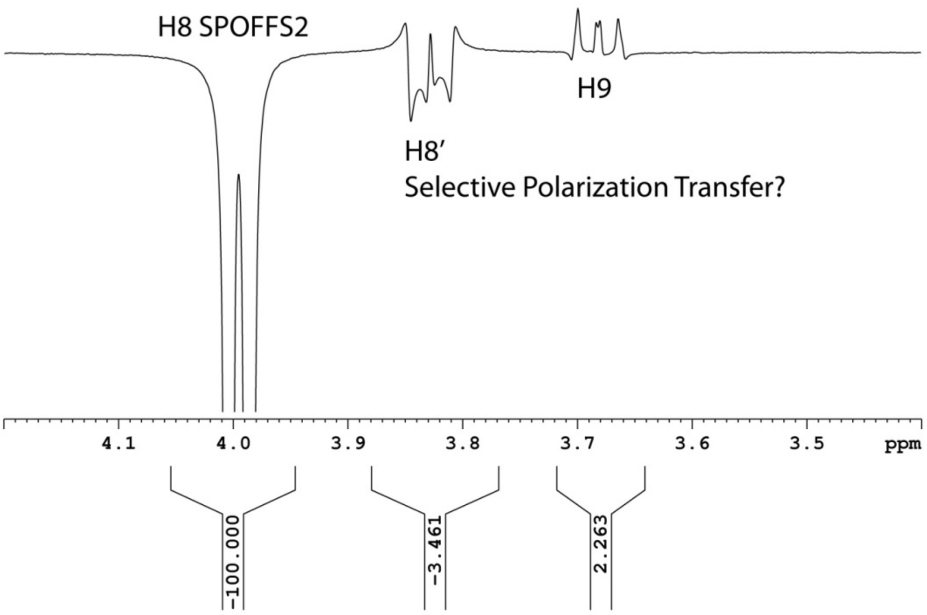

Figure 6. NOE buildup curve of H8→H8' at 80ms showing negative integral for H8'.
